# Changes in Muscle Cell Metabolism and Mechanotransduction Are Associated with Myopathic Phenotype in a Mouse Model of Collagen VI Deficiency

**DOI:** 10.1371/journal.pone.0056716

**Published:** 2013-02-20

**Authors:** Sara De Palma, Roberta Leone, Paolo Grumati, Michele Vasso, Roman Polishchuk, Daniele Capitanio, Paola Braghetta, Paolo Bernardi, Paolo Bonaldo, Cecilia Gelfi

**Affiliations:** 1 Department of Biomedical Sciences for Health, University of Milan, Segrate (MI), Italy; 2 Institute of Molecular Bioimaging and Physiology, National Research Council (CNR), Segrate (MI), Italy; 3 Department of Biomedical Sciences, University of Padova, Padova, Italy; 4 Telethon Institute of Genetics and Medicine, Institute of Protein Biochemistry, Italian National Research Council (CNR), Naples, Italy; University of Michigan, United States of America

## Abstract

This study identifies metabolic and protein phenotypic alterations in gastrocnemius, tibialis anterior and diaphragm muscles of *Col6a1^−/−^* mice, a model of human collagen VI myopathies. All three muscles of *Col6a1^−/−^* mice show some common changes in proteins involved in metabolism, resulting in decreased glycolysis and in changes of the TCA cycle fluxes. These changes lead to a different fate of α-ketoglutarate, with production of anabolic substrates in gastrocnemius and tibialis anterior, and with lipotoxicity in diaphragm. The metabolic changes are associated with changes of proteins involved in mechanotransduction at the myotendineous junction/costameric/sarcomeric level (TN-C, FAK, ROCK1, troponin I fast) and in energy metabolism (aldolase, enolase 3, triose phosphate isomerase, creatine kinase, adenylate kinase 1, parvalbumin, IDH1 and FASN). Together, these change may explain Ca^2+^ deregulation, impaired force development, increased muscle-relaxation-time and fiber damage found in the mouse model as well as in patients. The severity of these changes differs in the three muscles (gastrocnemius<tibialis anterior<diaphragm) and correlates to the mass-to-tendon (myotendineous junction) ratio and to muscle morphology.

## Introduction

Collagen VI is an extracellular matrix (ECM) protein that provides a structural link between ECM and the cell basement membranes [1]. In skeletal muscle, the ECM-basement membrane complex acts as a sensor that transfers the contractile force from the sarcomere to cells and ultimately to the tendon [2] translating extracellular mechanical stimuli into intracellular biochemical signals (mechanotransduction) [3]–[6]. Defects in the ECM can produce altered mechanosensing triggering the onset of diseases, like dystrophies [5], [7].

In humans, mutations in any of the three genes coding for collagen VI (*COL6A1*, *COL6A2*, *COL6A3*) result in defective ECM composition and cause a wide clinical spectrum of collagen VI myopathies including Ullrich congenital muscular dystrophy (UCMD), Bethlem myopathy (BM) [8]–[11] and myosclerosis myopathy [12]. Collagen VI myopathies are characterized by muscle weakness and contractures, associated with variable degrees of joint hyperlaxity. UCMD, the most severe form of collagen VI disorders, is characterized by early onset and proximal joint contractures associated with striking distal hyperlaxity. The orthopedic deformities and respiratory impairment, with diaphragm failure, generally develop within the first decade of life and are life-threatening in the most severe cases [13]–[16]. A milder form of human collagen VI myopathy is BM, characterized by early contractures of finger flexors, wrist, elbows and ankles. Respiratory failure and distal hyperlaxity are usually absent or are milder than in UCMD, although the latter may occur only in very young children with BM [8], [17]–[19].

Although the link between altered ECM and muscle fiber degeneration is not yet completely understood, the generation of a mutant mouse with targeted inactivation of the *Col6a1* gene (*Col6a1^−/−^*) provided a valuable tool for a better understanding of the pathophysiology of collagen VI myopathies [20]–[22]. *Col6a1^−/−^* mice carry a targeted inactivation of the α1 chain of collagen VI and exhibit an early onset myopathic phenotype that is reminiscent of features present in human BM and UCMD [20]. Studies in collagen VI null mice revealed that a cell pathway involved in these myopathies includes an increased opening of the permeability transition pore (PTP) within the mitochondrial inner membrane [22], [23]. This is associated with an impairment of autophagy that exacerbates the myofiber dysfunction, as defective mitochondria and proteins are not adequately cleared [21], [24]. Together, these defects lead to myofiber apoptosis and muscle wasting, which are characteristic also of human muscles affected by collagen VI mutations [22], [24]. Recent studies revealed that not only the muscles but also the tendon and osteotendineous junctions are affected in *Col6a1^−/−^* mice [25], [26]. Further studies suggested that alterations of the sarcolemma (the barrier and the link between ECM and intracellular environment) may be also involved in the myofiber phenotype caused by collagen VI deficiency [27].

Despite these major advances, several questions remain to be answered for a better understanding of the muscle-specific metabolic and phenotypic alterations characteristic of collagen VI myopathies. The aim of the study was to identify protein changes in the skeletal muscle of *Col6a1^−/−^* mice. We selected three different muscles (gastrocnemius, tibialis anterior and diaphragm) as these muscles are differently affected in the animal model [20] and in human collagen VI myopathies [8], [13]–[19].

This study for the first time identifies and correlates alterations in proteins involved in the metabolism and mechanotransduction in three muscles of *Col6a1^−/−^* mice, a model of human collagen VI myopathies. Our results identify cellular changes that explain the dysregulation of Ca^2+^, force development impairment, increased muscle-relaxation-time and fiber damage observed in patients. The severity of these changes differs in the three muscles (gastrocnemius<tibialis anterior<diaphragm) and correlates to the mass-to-tendon (myotendinous junction) ratio and to muscle morphology.

## Materials and Methods

### Ethics Statement

All the procedures involving animals and their care were conducted in conformity with the institutional procedures in compliance with national (D.L. No. 116, G.U. Suppl. 40, Feb. 18, 1992, Circolare No. 8, G.U., 14 Luglio 1994) and international regulations (EEC Council Directive 86/609, OJ L 358, 1 DEC.12, 1987; NIH Guide for the Care and use of Laboratory Animals, U.S. National Research Council, 1996).

Mouse procedures were approved by the Ethics Committee of the University of Padova and authorized by the Italian Ministry of Health. (Permit Number: 22675). The animals were anesthetized before sacrifice and all efforts were made to minimize suffering.

### Mice and Sample Collection

All experiments were performed in 6-month-old *Col6a1^−/−^* (collagen VI null) mice and *Col6a1^+/+^* (wild-type control) mice in the same inbred C57BL/6NCrl strain. Four male mice per group were used. Since previous studies demonstrated the absence of specific phenotypic differences between male and female collagen VI null mice [22], in the present study we have used only males.

Mice were housed in individual cages in an environmentally controlled room (23°C, 12-h light-dark cycle) and provided with food and water *ad libitum*. Animals were sacrified and gastrocnemius, tibialis anterior and diaphragm muscles were removed and immediately frozen in liquid nitrogen, ground in a dry ice-cooled mortar and stored at −80°C.

### Proteomic Analysis

#### Two-dimensional difference in gel electrophoresis (2D-DIGE)

Protein labeling, 2D-separation and analysis were performed exactly as previously described [28]. The adopted 3–10 non linear pH gradients enable separation of protein isoforms in the first dimension, providing a detailed pattern of the muscle proteome. The proteomic profiles of gastrocnemius, tibialis anterior and diaphragm muscles of *Col6a1^−/−^* mice were compared with their correspondent controls. Statistically significant differences of 2D-DIGE data were computed by Student’s t-test (*p*<0.01). False discovery rate (FDR) was applied as a multiple test correction in order to keep the overall error rate as low as possible. Power analysis was conducted on statistically changed spots, and only spots that reached a sensitivity threshold >0.8 were considered as differentially expressed.

#### Protein identification by MALDI-TOF and ESI mass spectrometry

For protein identification, semi preparative gels were loaded with unlabelled sample (400 µg per strip); electrophoretic conditions were the same as 2D-DIGE, and gels were stained with a total-protein fluorescent stain (Deep purple, GE Healthcare). Image acquisition was performed using a Typhoon 9200 laser scanner. Spots of interest were excised from gel using the Ettan spot picker robotic system (GE Healthcare), destained in 50% methanol/50 mM ammonium bicarbonate (AMBIC) and incubated with 30 µl of 6 ng/µl trypsin (Promega) dissolved in 10 mM AMBIC for 16 hours at 37°C. Released peptides were subjected to reverse phase chromatography (Zip-Tip C18 micro, Millipore), eluted with 50% acetonitrile/0,1% trifluoroacetic acid. Peptides mixture (1 µl) was diluted in an equal volume of 10 mg/ml α-cyano-4-hydroxycinnamic acid matrix dissolved in 70% acetonitrile/30% citric acid and processed on a Ultraflex III MALDI-ToF/ToF (Bruker Daltonics) mass spectrometer. Mass spectrometry was performed at an accelerating voltage of 20 kV and spectra were externally calibrated using Peptide Mix calibration mixture (Bruker Daltonics); 1000 laser shots were taken per spectrum. Spectra were processed by FlexAnalysis software v. 3.0 (Bruker Daltonics) setting the signal to noise threshold value to 6 and search was carried out by correlation of uninterpreted spectra to Rodentia entries (133347 sequences) in NCBInr 20100918 (11833178 sequences; 4040378175 residues). The significance threshold was set at p-value <0.05. No mass and pI constraints were applied and Trypsin was set as enzyme. One missed cleavage per peptide was allowed and carbamidomethylation was set as fixed modification while methionine oxidation as variable modification. Mass tolerance was set at 30 ppm for MS spectra. To confirm protein identification, a MS/MS spectrum was collected by Ultraflex III MALDI-ToF/ToF (Bruker Daltonics) mass spectrometer, as acceptance criterium. Spectra were searched against the database using BioTools v. 3.2 (Bruker Daltonics) interfaced to the on-line MASCOT software, which utilizes a robust probabilistic scoring algorithm. The significance threshold was set at p-value <0.05. One missed cleavage per peptide was allowed and carbamidomethylation was set as fixed modification while methionine oxidation as variable modification. Mass tolerance was set at 30 ppm and 0.5 Da for peptide and MS/MS fragment ion respectively.

In cases where this approach was unsuccessful, additional searches were performed using ESI-MS/MS. MS/MS spectra were recorded using a HCT Ultra mass spectrometer (Bruker Daltonics) interfaced to an EASY-nLC chromatograph (Proxeon). The samples were dissolved in 0.1% aqueous formic acid, injected onto a 0.075×100mm EASY-Column (Proxeon), and eluted with an acetonitrile/0.1% formic acid gradient (from 5 to 50% ACN). The capillary voltage was set to −1600 V, and data-dependent MS/MS acquisitions were performed on precursors with charge states of 2, 3 or 4 over a survey mass range of 300–1500. The collision gas was helium. Proteins were identified by correlation of uninterpreted MS/MS to Rodentia entries in NCBInr, using MASCOT software. No mass and pI constraints were applied and Trypsin was set as enzyme. One missed cleavage per peptide was allowed, and the precursor and fragment ion tolerance window was set to 0.3 Da. Carbamidomethylation of cysteine was set as fixed modification, whereas methionine oxidation as variable modification.

### Immunoblotting

Protein extracts (50 µg) from pooled *Col6a1^−/−^* and control muscles were loaded in triplicate and resolved on 6%, 10% and 12% polyacrylamide gels, according to protein molecular weight. Blots were incubated with rabbit or goat polyclonal primary antibodies (Cell Signaling Technology and Santa Cruz Biotechnology) as follows: anti-TN-C (1∶500), anti-ROCK1 (1∶500), anti PI3K (1∶1000), anti FAK (1∶1000), anti-PPARγ (1∶1000), anti-PPARα (1∶1000), anti-PPARβ (1∶1000), anti-Sirt1 (1∶1000), anti-Sirt3 (1∶1000), anti-FASN (1∶1000), anti-IDH1 (1∶1000), anti-IDH2 (1∶1000) and anti β-tubulin (1∶1000). To confirm 2D-DIGE data, 15% of differential expressed proteins were validated by Immunoblotting. Blots were incubated with polyclonal primary antibodies (as shown in Fig. S1) as follows: anti-PKM2 (1∶1000), anti-VDAC1 (1∶1000), anti-ALDOA (1∶1000) and anti-UQCRC1 (1∶1000) for gastrocnemius; anti-PKM2, anti-ACO2 (1∶1000), anti-SDHA (1∶1000) and anti-ATP5B (1∶1000) for tibialis anterior; anti-PKM2, anti-CA3 (1∶2000), anti-ALDOA and anti-ATP5B for diaphragm. After washing, membranes were incubated with anti-rabbit (GE Healthcare) or anti-goat (Santa Cruz Biotechnology) secondary antibodies conjugated with horseradish peroxidase. Signals were visualized by chemiluminescence using the ECL Plus detection kit. An image analysis system (Image Quant TL, Molecular Dynamics) was performed followed by statistical analysis (Student’s t-test, *p*<0.05).

### Pyruvate Assay

Pyruvate concentration was determined by a colorimetric assay measured at 570 nm (Sigma, cat.# MAK071) proportional to the pyruvate present.

### Enzymatic Assays

Citrate synthase (CS) was analyzed by a spectrophotometric assay (Sigma, cat.# CS0720). NADH dehydrogenase activity (complex I) was analyzed by a spectrophotometric assay as described previously [29] and dehydrogenase activity (complex II) was analyzed by a colorimetric-continuous method as described previously [30].

### Electron Microscopy

Samples were fixed with 2% glutaraldehyde in Hepes 0.2 M (pH 7.4), in 2% OsO4, buffered with 0.1 sodium cacodylate. The specimens were washed in distilled water, stained with 2% aqueous uranyl acetate, dehydrated in alcohol and embedded in Epon resin. Thin sections were cut with a Leica EM UC6 ultramicrotome (Leica Microsystems, Vienna, Austria). EM images were acquired from thin sections using a FEI Tecnai-12 electron microscope (FEI, Eindhoven, Netherlands) equipped with a VELETTA CCD digital camera (Soft Imaging Systems GmbH, Munster, Germany).

## Results

### Gastrocnemius, Tibialis Anterior and Diaphragm Muscles: Proteomic Analysis

**Figure 1:**
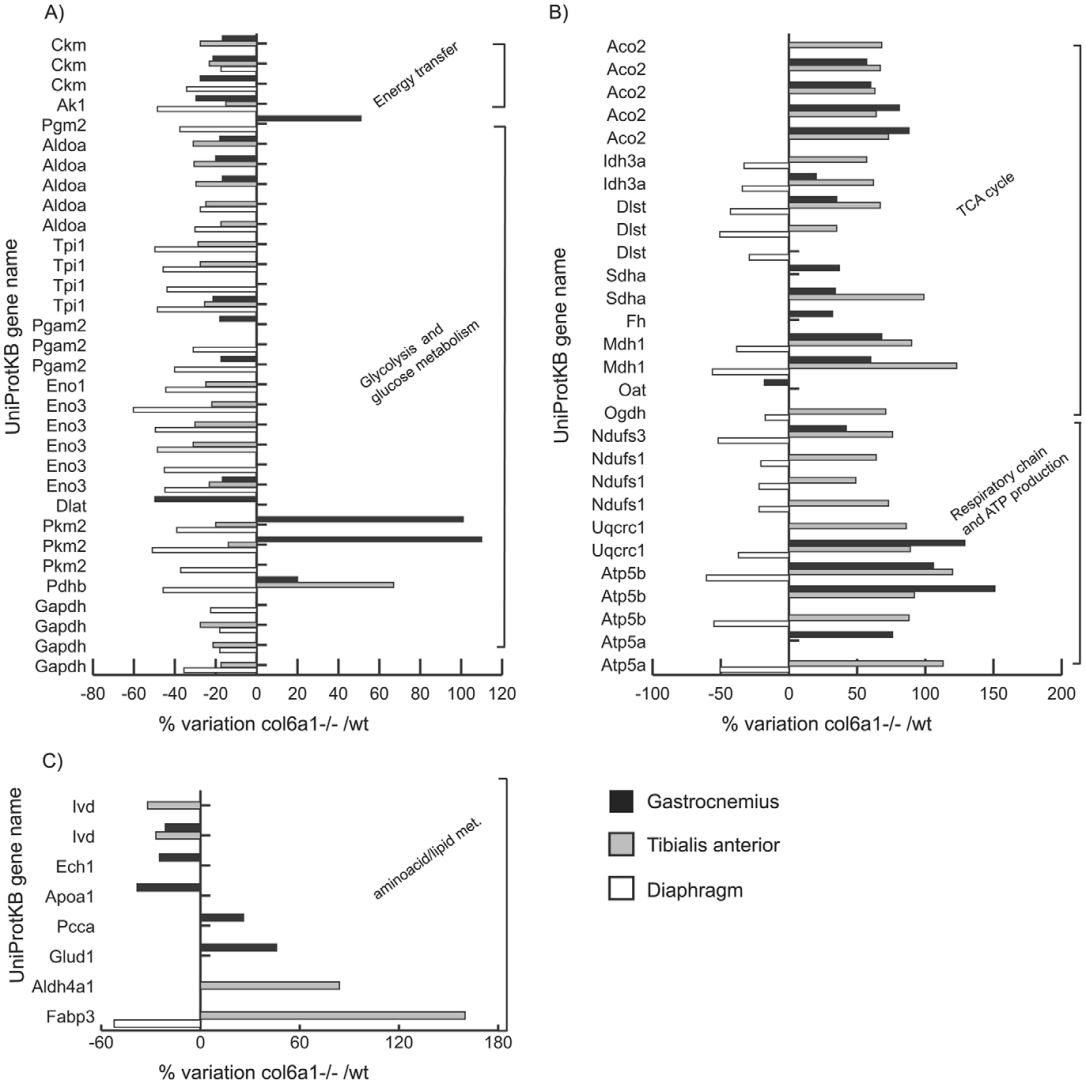
Histograms of differentially expressed metabolic proteins in muscles of *Col6a1* ^−/−^ *versus* wild-type controls. Histograms of differentially expressed proteins in gastrocnemius (black bars), tibialis anterior (grey bars) and diaphragm (white bars) muscles. Isoforms of proteins significantly altered (Student's T-test, p<0.01) are expressed as % of spot areas variation in *Col6a1* ^−/−^ *versus* wild-type controls. Panel A: energy transfer and glycolysis and glucose metabolism; panel B: TCA cycle, respiratory chain and ATP production; panel C: aminoacid and lipid metabolism. (Ckm: muscle creatine kinase; Ak1: adenylate kinase isoenzyme 1; Pgm2: phosphoglucomutase 2; Aldoa: fructose-bisphosphate aldolase A; Tpi1: triosephosphate isomerase 1; Pgam2: phosphoglycerate mutase 2; Eno1: enolase 1 (alpha); Eno3: enolase 3 (beta muscle); Dlat: dihydrolipoyllysine-residue acetyltransferase; Pkm2: pyruvate kinase isozymes M1/M2; Pdhb: pyruvate dehydrogenase (lipoamide) beta; Gapdh: glyceraldehyde-3-phosphate dehydrogenase; Aco2: aconitase 2; Idh3a: isocitrate dehydrogenase [NAD] subunit alpha; Dlst: dihydrolipoamide S-succinyltransferase; Sdha: succinate dehydrogenase complex, subunit A; Fh: fumarate hydratase 1; Mdh1: cytosolic malate dehydrogenase; Oat: ornithine aminotransferase; Ogdh: 2-oxoglutarate (α-ketoglutarate) dehydrogenase; Ndufs3: NADH dehydrogenase (ubiquinone) iron-sulfur protein 3; Ndufs1: NADH dehydrogenase (ubiquinone) Fe-S protein 1; Uqcrc1: ubiquinol-cytochrome-c reductase complex core protein I; Atp5b: ATP synthase, H+ transporting mitochondrial F1 complex, beta subunit; Atp5a: ATP synthase subunit alpha; Ivd: isovaleryl-CoA dehydrogenase; Ech1: delta (3,5)-delta(2,4)-dienoyl-CoA isomerase; Apoa1: Apolipoprotein A-I; Pcca: propionyl-CoA carboxylase alpha chain; Glud1: glutamate dehydrogenase 1; Aldh4a1: delta-1-pyrroline-5-carboxylate dehydrogenase; Fabp3: fatty acid-binding protein).

**Figure 2:**
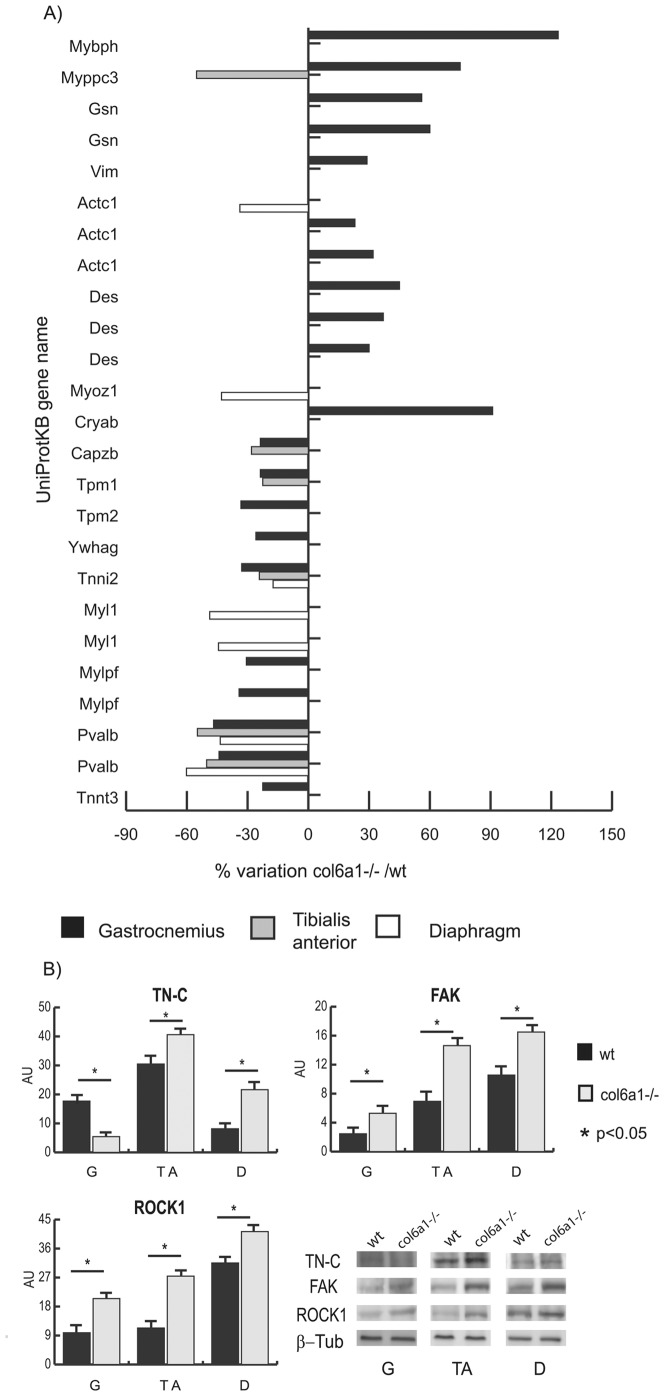
Histograms of proteomic results and Immunoblotting of cytoskeletal proteins. Panel A: histograms of structural and cytoskeletal proteins differentially expressed in gastrocnemius (black bars), tibialis anterior (grey bars) and diaphragm (white bars) muscles. Proteins significantly altered (Student’s t-test, *p*<0.01) are expressed as % of spot volume variation in *Col6a1* ^−/−^ versus wild-type. (Mybph: myosin binding protein H, Mybpc3: myosin binding protein c; Gsn: gelsolin; Vim: vimentin; Actc1: actin, alpha cardiac muscle 1; Des: desmin; Myoz1: myozenin1; Cryab: alpha-crystallin B chain; Capzb: capping protein (actin filament) muscle Z-line, beta isoform; Tpm1: tropomyosin 1 alpha chain; Tpm2: tropomyosin 2 beta chain; Ywhag: 14-3-3 protein gamma; Tnni2: troponin I, fast skeletal muscle; Myl1: myosin A1 catalytic light chain; Mylpf: myosin light chain, phosphorylatable, fast skeletal muscle; Pvalb: parvalbumin; Tnnt3: troponin T, fast skeletal muscle). Panel B: histograms and representative immunoblot images of TN-C, FAK and ROCK1 in gastrocnemius, tibialis anterior and diaphragm of *Col6a1* ^−/−^ and wild-type mice (n = 4; mean ± S.D.; Student’s T-test).

The proteomic profiles of the three muscles from *Col6a1^−/−^* mice were compared with the corresponding controls. Among the differentially expressed proteins, a total of 76, 66 and 65 changed spots were identified in the gastrocnemius, tibialis anterior and diaphragm, respectively. Histograms of differentially expressed proteins are shown in Fig. 1 (panels A–C), Fig. 2 (panel A) and Fig. S2. A representative proteomic map (see Fig. S3) and the identification UniProtKB accession (AC) numbers from the three muscles are shown in supplementary material together with peptide mass fingerprint (PMF) and Liquid Chromatography/Mass Spectrometry (LC-MS/MS) data (see Table S1 and Data S1). The main alterations identified can be grouped as metabolic or structural/contractile.

### Muscle Metabolism

The metabolic profile of each muscle was analyzed using proteomics, enzymatic assays and immunoblottings. The identified proteins are sub-grouped into energy transfer, glycolysis, anaerobic metabolism, oxidative metabolism, respiratory chain, and amino acid and lipid metabolism.

#### Energy transfer: proteomics (Fig. 1, panel A; Fig. 3)

Creatine kinase (M-CK) and adenylate kinase-1 (Ak1) are essential mediators for cellular energetic and metabolic signaling processes in tissues characterized by high and sudden increases of energy demand, such as skeletal muscle. M-CK and Ak1 were decreased in the three *Col6a1^−/−^* muscles, suggesting that the cellular ability to maintain ATP turnover, under muscle functional load, is reduced.

#### Glycolysis: proteomics (Fig. 1, panel A; Fig. 3)

**Figure 3:**
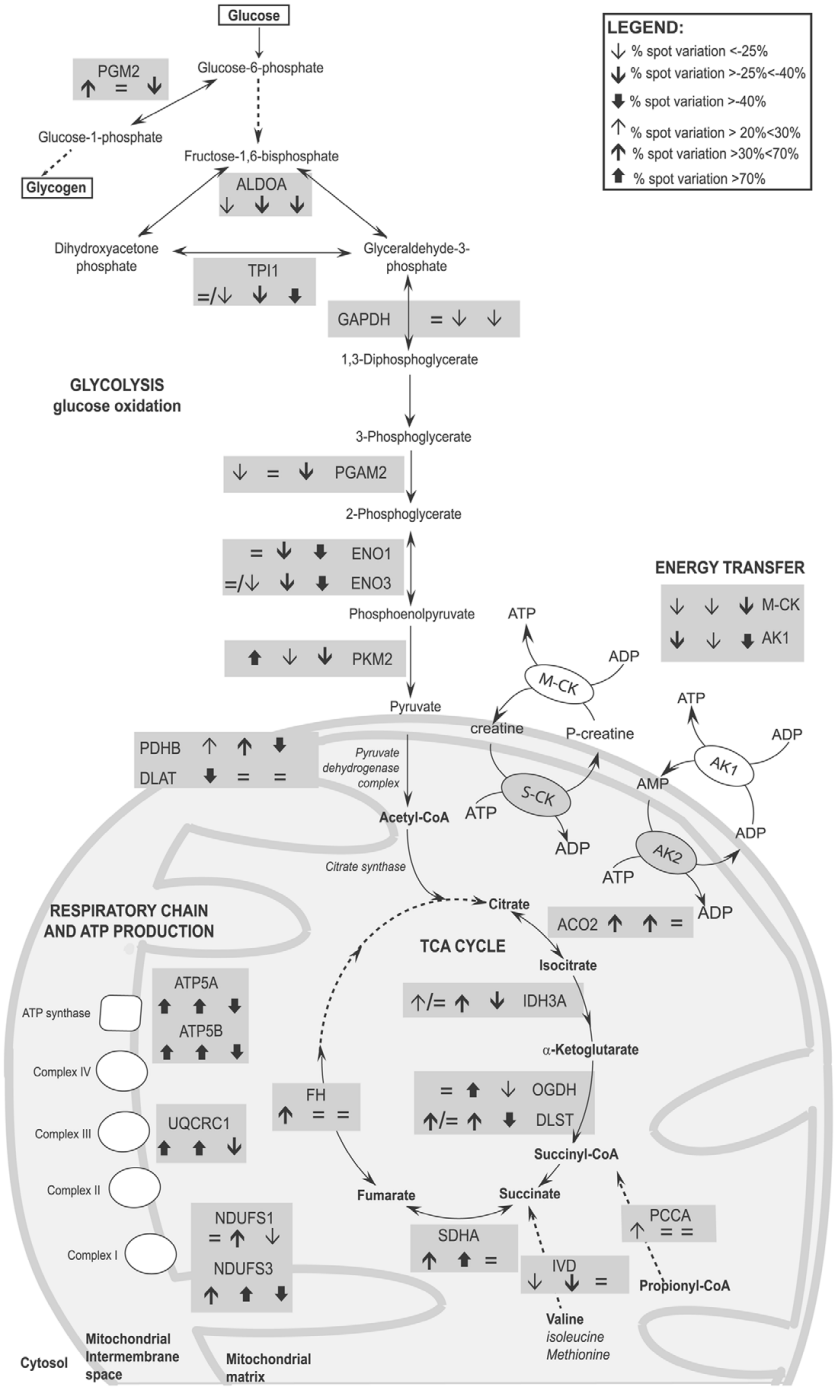
Schematic representation of metabolic enzymes deregulated in gastrocnemius, tibialis anterior and diaphragm muscles. Grey rectangles contain gene names and arrows indicate the protein trend in gastrocnemius, tibialis anterior and diaphragm, respectively. The arrows direction indicates up- and down-regulation, the thickness the % of spot variation.  = /↓ or  = /↑ indicate the differential expression of isoforms of the same protein in a given muscle (for details see Table S1).

In gastrocnemius, phosphoglucomutase 2 (Pgm2), which interconverts glucose-1-P to glucose-6-phosphate, was increased. Most of the proteins involved in the glycolytic pathway were decreased (Fig. 1, panel A and Fig. 3; fructose biphosphate aldolase A, Aldoa*;* triosephosphate isomerase, Tpi1; phosphoglycerate mutase, Pgam2; and enolase, Eno3), with the exception of pyruvate kinase M1/M2 isozymes (Pkm2) that were increased. Two subunits of pyruvate dehydrogenase complex were changed: dihydrolipoyllysine-residue acetyltransferase (Dlat) was decreased, while pyruvate dehydrogenase lipoamide beta (Pdhb*)* was increased.

In tibialis anterior, the glycolytic proteins were overall decreased (Fig. 1, panel A and Fig. 3; Aldoa; Tpi1; Gapdh; Eno1; Eno3 and Pkm2), with the exception of Pdhb that was increased.

In diaphragm, the glycolytic proteins were all decreased, except Dlat that was unchanged compared with its control.

#### TCA cycle and associated metabolic substrates: proteomics (Fig. 1, panel B; Fig. 3) and pyruvate assay (Fig. 4, panel A)

In gastrocnemius, proteins of the TCA cycle were overall increased (Aconitase 2, Aco2; and Isocytrate dehydrogenase, Idh3a - which together enhance the production of α-ketoglutarate from citrate; dihydrolipoamide S-succinyltranferase, Dlst - involved in the lipoic/dihydrolipoate antioxidant redox balance; the subunit A of the succinate dehydrogenase complex, Sdha; and fumarate hydratase 1, Fh). Also the cytosolic malate dehydrogenase Mdh1 (a protein involved in the malate-aspartate shuttle that transfers reducing equivalent, produced by glycolysis, from cytoplasm to mitochondria and *viceversa*) was increased. Conversely, ornithine aminotransferase (Oat), a mitochondrial enzyme that catalyzes the reversible inter-conversion of ornithine and proline, was decreased. In this reaction, glutamate acts as the amine donor for Oat, forming α-ketoglutarate and in the reverse direction, α-ketoglutarate accepts the amine group, forming glutamate and leading to the ultimate conversion of proline to ornithine. The decrease in Oat suggests an attempt of the gastrocnemius muscle to control energy balance.

In tibialis anterior, all the proteins of the TCA cycle were increased (Aco2; Idh3a; α-ketoglutarate dehydrogenase, Ogdh; Dlst and Sdha), as well as cytosolic malate dehydrogenase (Mdh1).

In diaphragm, a limited number of proteins of the TCA cycle (Idh3a, Ogdh, Dlst) and Mdh1 were decreased.

The proteomic analysis of gastrocnemius and tibialis anterior of *Col6a1^−/−^* mice (identified in the energy transfer, glycolysis and TCA cycles) suggests that the overall decrease of glycolysis is compensated by an increase of Pkm2 (specifically for gastrocnemius) and Pdhb (for both muscles) to sustain the activity of pyruvate dehydrogenase complex (PDH). The latter, associated with an increase in α-ketoglutarate complex (e.g. Ogdh, Dlst) and IDH3, could support the activity of the TCA cycle. These results also show that gastrocnemius appears less impaired at the metabolic level than tibialis anterior.

The proteomic results obtained in the diaphragm indicate a decreased flux in glycolysis, PDH complex, IDH3, and α-ketoglutarate dehydrogenase complex, suggesting a severe metabolic imbalance. The decrement of TCA cycle enzymes suggests that the excess of citrate not utilized by the TCA cycle could be exported to the cytosol to sustain the synthesis of lipids.

**Figure 4:**
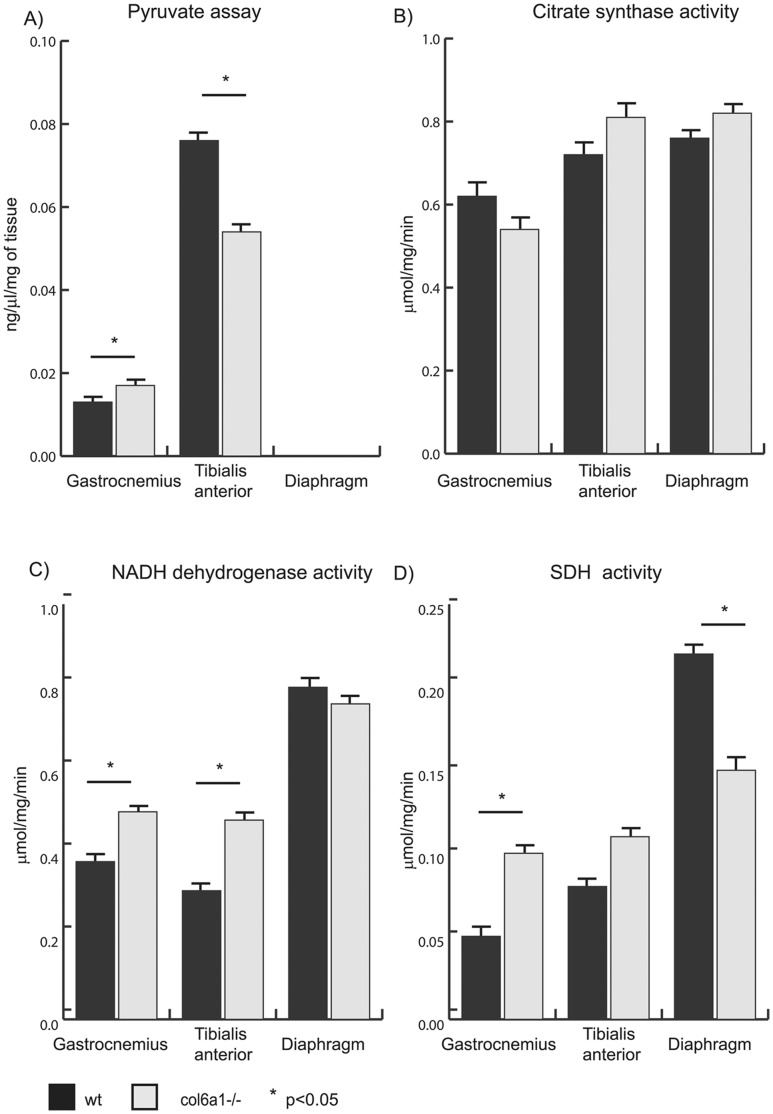
Results of enzymatic assays. Histograms showing pyruvate quantitation (panel A), citrate synthase enzymatic activity (panel B), NADH dehydrogenase (complex I) enzymatic activity (panel C) and succinate dehydrogenase (SDH, complex II) enzymatic activity (panel D) in gastrocnemius, tibialis anterior and diaphragm muscles from *Col6a1* ^−/−^ and wild-type (wt) mice.

The pyruvate concentrations detected in the three *Col6a1^−/−^* muscles support the proteomic results. The concentration of pyruvate was increased in gastrocnemius, decreased in tibialis anterior and not detectable in diaphragm compared with the corresponding wild-type control samples. The results suggest that in *Col6a1^−/−^* gastrocnemius, pyruvate was generated partially by glycolysis and by the malate/aspartate shuttle. In the *Col6a1^−/−^* tibialis anterior, where the downregulation of glycolysis was more pronounced, pyruvate production was reduced compared to control. In *Col6a1^−/−^* diaphragm, glycolysis was blunted and the malate/aspartate shuttle was not finalized to pyruvate production, resulting in undetectable pyruvate levels (Fig. 4, panel A).

#### Respiratory chain and ATP production: proteomics (Fig. 1, panel B and Fig. 3) and enzymatic activities (Fig. 4 and Fig. 3)

In gastrocnemius, an overall increase of respiratory chain proteins (NADH dehydrogenase ubiquinone iron sulfur 3 - Ndufs3 of complex I; ubiquinol-cytochrome *c* reductase core protein I, Uqcrc1 of complex III) and of ATP synthase (beta Atp5b and alpha Atp5a) were detected. In tibialis anterior and diaphragm, an overall increase in the proteins of the respiratory chain (Ndufs3 and Ndufs1; Uqcrc1; Atp5b and Atp5a) was detected.

The enzymatic activities of citrate synthase (CS), complex I and complex II in the three muscles of *Col6a1^−/−^* mice and of the corresponding wild-type controls (Fig. 4, panels B-D) were assessed. The CS activity was not significantly changed in the three muscles, whereas NADH dehydrogenase activity (complex I) and succinate dehydrogenase activity (complex II) were increased in gastrocnemius and tibialis anterior, although complex II did not reach statistical significance in the latter case. These results confirm the proteomic results and further suggest that gastrocnemius and tibialis anterior of *Col6a1^−/−^* mice are attempting to maintain the energetic homeostasis by increasing complexes I and II activities. On the other hand, in the *Col6a1^−/−^* diaphragm, the activity of complex I was similar to wild-type, while the activity of complex II was decreased. These results support the proteomic results and suggest that an energetic imbalance is occurring in this muscle.

#### Aminoacid and lipid metabolism: proteomics (Fig. 1, panel C; and Fig. 3) and immunoblotting (Fig. 5)

In gastrocnemius, three enzymes involved in the aminoacid and lipid metabolism (isovaleryl-CoA dehydrogenase, Ivd; δ(3,5)-dienoyl CoA isomerase, Ech1-a mitochondrial NAD^+^ dependent dehydrogenase involved in fatty acid metabolism; and apolipoprotein A-I, Apoa1) were decreased. Two enzymes mainly involved in catabolism (propionyl-CoA carboxylase alpha chain, Pcca - essential for the catabolism of valine and isoleucin aminoacids and odd-chain fatty acids; and glutamate dehydrogenase 1, Glud1, which functions in both the synthesis and the catabolism of glutamate) were increased.

In tibialis anterior, a decrease in Ivd and an increase of δ-1-pyrroline-5-carboxylate dehydrogenase (Aldh4a1, an enzyme which converts pyrroline-5-carboxylate to glutamate) and of fatty acid binding protein 3 (Fabp3, a fatty acid transporter and scavenger of fatty acids peroxides) [31]–[33] were observed.

In diaphragm, the only proteomic change was a decrease in Fabp3, suggesting a decrease in the lipid transport and scavenging of lipid peroxides.

**Figure 5:**
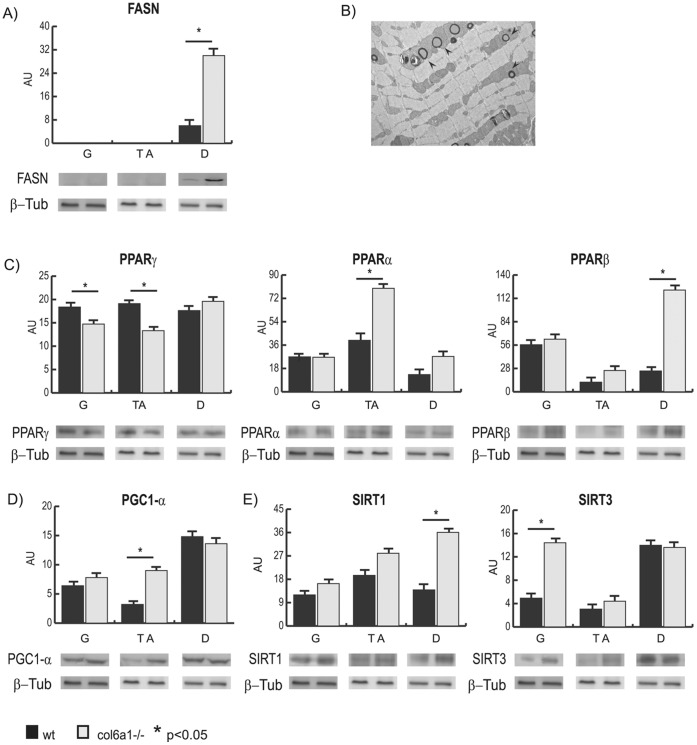
Immunoblotting of proteins involved in lipid metabolism. Histograms and representative immunoblot images of FASN (panel A), PPARγ, α and β (panel C), PGC1-α (panel D), SIRT1 and SIRT3 (panel E) in gastrocnemius, tibialis anterior and diaphragm from *Col6a1* ^−/−^ and wild-type mice (n = 4; mean ± S.D.; Student’s T-test). Panel B: electron microscopy image showing lipid accumulation in the diaphragm of *Col6a1* ^−/−^ mice. Black arrows show lipid droplets.

To further support the results observed by proteomics, the profile of the fatty acid synthase (FASN) protein was monitored in the three muscles of the *Col6a1^−/−^* and compared with the corresponding wild-type controls (Fig. 5, panel A).

In gastrocnemius and tibialis anterior, FASN was undetectable both in *Col6a1^−/−^* and in wild-type controls, whereas in the diaphragm the FASN level was significantly increased compared with the corresponding control (Fig. 5, panel A). This increase was associated with an accumulation of lipid droplets in the tissue (Fig. 5, panel B).

The peroxisome proliferator-activated receptors (PPARs) and peroxisome proliferator-activated receptor gamma coactivator-1-alpha (PGC1α), two master regulators of mitochondrial biogenesis, respiration and adipocyte differentiation, were analyzed in the three muscles of *Col6a1^−/−^* mice and compared with the corresponding wild-type muscles. PPARγ, a regulator of adipocyte differentiation [34], was decreased in the gastrocnemius and tibialis anterior and unchanged in diaphragm (Fig. 5, panel C). PPARβ, involved in fatty acid metabolism and inflammation [35], [36], was significantly increased in the diaphragm only (Fig. 5, panel C). PPARα, a key player in fatty acid oxidation, lipid and lipoprotein metabolism and oxidative stress [37], and PGC1α were unchanged in gastrocnemius and diaphragm, whereas they were increased in tibialis anterior (Fig. 5, panel C and D).

Two de-acetylase enzymes, sirtuin 1 (SIRT1) and sirtuin 3 (SIRT3), were analyzed to better characterize the potential dysmetabolism observed in the three muscles. Sirtuins were quantified in the three muscles of *Col6a1^−/−^* mice and compared with correspondent wild-type muscles. SIRT1, induced by NAD^+^, regulates metabolism by repressing glycolytic genes [38], [39] and induces DNA repair [40]. In gastrocnemius, SIRT1 did not change, in the tibialis anterior it was slightly increased (but not to statistically significant levels), while in diaphragm it was significantly increased (Fig. 5, panel E). These results suggest that the decrease in Gapdh (observed in tibialis anterior and diaphragm) and the decrease of Pdhb (observed in diaphragm) may modulate the NAD^+^/NADH ratio, resulting in SIRT1 overexpression.

SIRT3 acts as a positive modulator of fatty acid oxidation [41] and TCA cycle [42], [43] and displays a protective role against oxidative damage [44]–[46]. SIRT3 was increased in gastrocnemius, while it did not change in tibialis anterior and diaphragm (Fig. 5, panel E).

The expression of FASN, PPARs, PGC1α and sirtuins in the three muscles confirms the proteomic results.

#### Metabolic (citrate) refueling (Fig. 3 and 6): immunoblottings

In the absence of glucose, glutamine is the major substrate available to the cells [47]. In skeletal muscle, glutamine can act as a nitrogen donor in the synthesis of proteins and nucleosides [48]. It can also be converted to α-ketoglutarate, which can anaplerotically support the tricarboxylic acid (TCA) cycle or be reductively transformed by cytosolic IDH1 to generate citrate [49]–[51].

**Figure 6:**
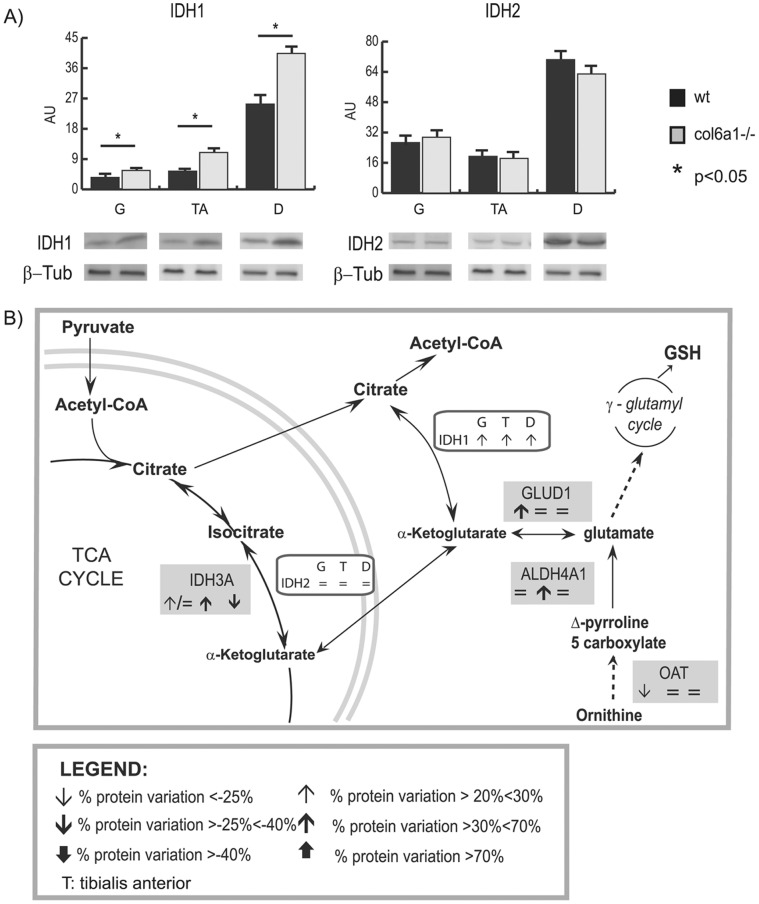
Immunoblotting and 2D-DIGE results on proteins involved in glutamate metabolism. Panel A: Histograms and representative immunoblot images of IDH1 and IDH2 in gastrocnemius, tibialis anterior and diaphragm from *Col6a1* ^−/−^ and wild-type mice (n = 4; mean ± S.D.; Student’s T-test). Panel B: schematic representation of IDHs pathways. Grey and empty rectangles contain gene names of deregulated proteins observed by 2D-DIGE and western blotting, respectively. Arrows indicate the protein trend in gastrocnemius (G), tibialis anterior (T) and diaphragm (D). For 2D-DIGE, the arrows direction indicates up- and down-regulation, the thickness the % of spot variation.  = /↓ or  = /↑ indicate the differential expression of isoforms of the same protein in a given muscle. For western blotting, the arrows direction indicates the up- and down-regulation of relative variation of *Col6a1* ^−/−^ *versus* control wild-type muscle.

Isocitrate dehydrogenases (IDHs) reversibly catalyze oxidative decarboxylation of isocitrate to α-ketoglutarate (α-KG) [47], [52] (Fig. 6 panel B). IDH1 (NADP^+^-dependent) functions in the cytosol and peroxisomes, and its activity becomes predominant under hypoxia for *de novo* lipogenesis [47]. IDH2 (NADP^+^-dependent) and IDH3 (NAD^+^-dependent) localize in the mitochondria, where they are positively modulated by the mitochondrial matrix Ca^2+^ concentration. IDH3 is allosterically regulated and operates in the oxidative direction. Proteomic results indicated an increase of IDH3 in gastrocnemius and tibialis anterior and a decrease in diaphragm, whereas IDH1 and IDH2 were undetectable in *Col6a1^−/−^* compared to wild-type muscles.

Quantitative immunoblottings show that IDH1 was overexpressed, while IDH2 remained unchanged in the three muscles (Fig. 6, panel A).

These results suggest that IDH1 sustains the synthesis of glutamine through α-ketoglutarate conversion in gastrocnemius and tibialis anterior of *Col6a1^−/−^* mice. In diaphragm, reductive carboxylation of α-ketoglutarate sustains, *via* IDH1, the lipogenic citrate synthesis. Furthermore, the observed increase in FASN confirms that citrate is finalized to *de novo* lipogenesis [47], [53], [54].

#### Muscle metabolism: summary of changes

In *Col6a1^−/−^* gastrocnemius, the glycolytic enzymes are overall decreased, whereas the TCA cycle enzymes are increased. Furthermore, the increase of Glud1 and IDH1, which generates α-ketoglutarate, is finalized to glutamine production. The TCA cycle activity is associated with an increased respiratory chain activity and SIRT3, resulting in an increased energy production and possibly in at least partial activation of defense mechanisms against oxidative stress.

In *Col6a1^−/−^* tibialis anterior, glycolytic enzymes are decreased, but energy production is still sustained by the TCA cycle. Conversely, IDH1 and Aldh4a1, which sustain glutamine production, are increased. Furthermore, also in tibialis anterior the respiratory chain activity is overall increased. However, the specific increases in PPARα and PGC1α suggest that tibialis anterior promotes cellular metabolism and muscle lipid oxidation.

In *Col6a1^−/−^* diaphragm, glycolysis is blunted, differently from gastrocnemius and tibialis anterior. TCA cycle and respiratory chain are also decreased, forcing IDH1 to reductively generate lipogenic citrate. The maintenance of CS activity and the decrease of cytosolic Mdh1 suggest that citrate is exported to the cytosol, possibly due to the TCA cycle impairment. Furthermore, due to Mdh1 decrease, oxaloacetate, which cannot re-enter into mitochondria, is reduced to α-ketoglutarate. The latter acts as IDH1 substrate (see Fig. S4), resulting in abnormal fatty acid synthesis, which is consistent with the increase in FASN and lipid droplets. The overexpression of SIRT1 and the increase of PPARβ (associated with the decrease in Fabp3) appear unable to counteract the detrimental lipid signaling leading to cell death.

#### Structural and contractile proteins: proteomics (Fig. 2A; Fig. 7) and immunoblottings (Fig. 2B; Fig. 7)

In *Col6a1^−/−^* gastrocnemius, proteins involved in cytoskeletal stabilization were increased (fetal myosin binding protein H, Mybph; myosin binding protein C, Mybpc3; gelsolin, Gsn; vimentin, Vim; alpha cardiac actin, Actc1; desmin, Des; myozenin 1, Myoz1; and alpha crystallin B chain, Cryab). Conversely, other structural and contractile proteins were decreased (actin capping protein A, Capzb; tropomyosin 1 alpha and beta chains, Tpm1 and Tpm2; 14-3-3 protein gamma, Ywhag; troponinin I fast, Tnni2; myosin A1 catalytic light chain, Myl1; myosin light chain phosphorylatable fast isoforms, Mylpf), suggesting instability of actin filament, muscle fiber damage, force development impairment, deregulation of Ca^2+^ handling, and increased muscle relaxation time (decrease of parvalbumin, Pvalb).

In *Col6a1^−/−^* tibialis anterior, several proteins were decreased (Mybpc3; Capzb; Myoz1; Tpm1; Tnni2 and Pvalb), suggesting an alteration in actin filaments polymerization, muscle fiber damage, deregulation of Ca^2+^ handling, and increased muscle relaxation time (decrease of parvalbumin, Pvalb).

In *Col6a1^−/−^* diaphragm, only Mybpc3, a regulator of actin-myosin interaction and remodeling, was increased and several proteins mainly involved in the Ca^2+^ binding were decreased (Capzb; Actc1; troponin T fast, Tnnt3; Myl1 and Pvalb). These results suggest that deregulation of Ca^2+^ handling and instability of the actin filament may play a predominant role in the diaphragm impairment.

**Figure 7:**
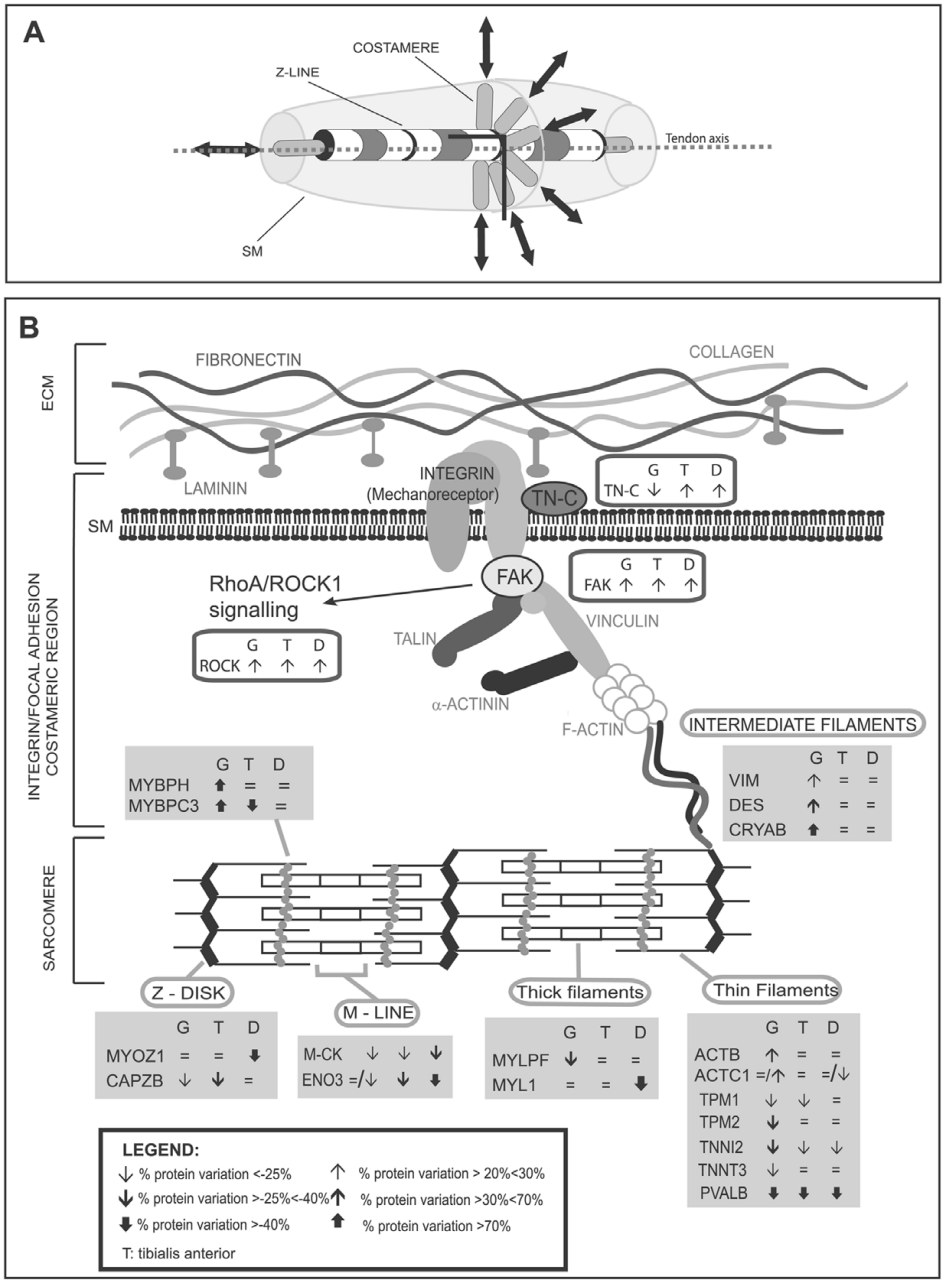
Costameric schematization, immunoblotting and proteomic results of proteins involved in mechanotransduction. Panel A: schematic representation of costameres and force transmission by ECM. The junction between cell matrix to the muscle cell is represented by costameres which consist of a complex protein network that forms a physical attachment of the underlying, outer Z discs of the muscle cell to its surrounding, stress-tolerant, ECM. These specialized sites occur over the entire sarcolemma, where the transmission of force is concentrated (black arrows) from cells to ECM, along and perpendicularly to the tendon axis (dotted grey line) with the role of keeping sarcolemma aligned with nearby contractile structures [79], [80]. The absence of collagen VI would alter the signaling from the interstitial connective tissue to the muscle fiber and tendon [1], [2], [4], [81]. Panel B: Graphical representation of the muscle unit involved in mechanotransduction in which deregulated proteins are indicated. For details see Table S1. Grey and empty rectangles contain gene names of deregulated proteins observed by 2D-DIGE and western blotting, respectively. Arrows indicate the protein trend in gastrocnemius (G), tibialis (T) and diaphragm (D). For 2D-DIGE results, the arrows direction indicates the up and down regulation, the thickness the % of spot variation, as described in “legend”.  = /↓ or  = /↑ indicate the differential expression of isoforms of the same protein in a given muscle. For western blotting, the arrows direction indicates the up and down regulation of relative variation of Col6a1−/− versus control muscle.

The identified changes in structural and contractile proteins suggest that signaling from the sarcomeres to muscle cell is altered, albeit at a different extent, in the three muscles. Specific immunoblotting assays were performed to further analyze muscle changes induced by altered mechanotransduction (Fig. 2, panel B and Fig. 7).

Tenascin C (TN-C) is a glycoprotein located in the area where high mechanical forces are transmitted (e.g. at the myotendineous junction) [55]; it provides de-adhesive properties and plays a critical role in muscle injury repair [56]–[58]. In *Col6a1^−/−^* gastrocnemius, TN-C was decreased resulting in a possible increase in matrix adhesion. Mybpc3, a regulator of actin-myosin interaction and remodeling [59] and intermediate filament proteins (Vim, Des, Cryab) were increased, suggesting a compensatory pathway in the sarcomere. In *Col6a1^−/−^* tibialis anterior and diaphragm, TN-C was increased resulting in a possible decrease of matrix adhesion.

Focal adhesion kinase (FAK) regulates focal adhesion turnover and adhesion strength [60] and is essential for costamerogenesis [61]. Costameres promote the alignment of myofibrils and anchor myofibril Z-bands to the sarcolemma. Furthermore, Rho/ROCK1 pathway promotes myosin contractility and mediates reorganization of the actin cytoskeleton [62], [63]. Focal adhesion kinase (FAK) and Rho-associated protein kinase 1 (ROCK1) were increased in the three *Col6a1^−/−^* muscles, further supporting alterations of cytoskeletal tension and of mechanotransduction (Fig. 7).

#### Muscle phenotype: summary of changes

In *Col6a1^−/−^* mice, gastrocnemius tends to stabilize the Z-line and the costameric structure (described in Fig. 7, panel A and B) with increase in Gsn, Vim, Actc1, Des, Myoz1 and Cryab), in an attempt to adapt to the physical surrounding. However, despite these compensatory mechanisms, *Col6a1^−/−^* gastrocnemius is characterized by instability of actin filament, fiber damage, impairment of force development, deregulation of Ca^2+^ handling (e.g. decrease in Tnni2, Mylpf and Myl1 and increase in Mybph), increased muscle relaxation time (decrease of Pvalb), abnormal muscle elasticity (i.e. decrease in TN-C; increase in Mybpc3) and altered cytoskeletal tension (i.e. increase in FAK and ROCK1*Col6a1^−/−^* tibialis anterior is characterized by changes in actin filaments polymerization, presence of muscle fiber damage, deregulation of Ca^2+^ handling (e.g. decrease in Capzb and Tpm1), increase in muscle relaxation time (decrease of Pvalb), abnormal muscle elasticity (i.e. increase in TN-C; decrease in Mybpc3) and altered cytoskeletal tension (i.e. increase in FAK and ROCK1).

*Col6a1^−/−^* diaphragm is characterized by more severe phenotypic changes in comparison with gastrocnemius and tibialis anterior, with instability of actin filaments, deregulation of Ca^2+^ handling (e.g. decrease in Myoz1, Actc1, Myl1 and Pvalb), increased muscle relaxation time (decrease of Pvalb), abnormal muscle elasticity (i.e. increase in TN-C and Mybpc3) and altered cytoskeletal tension.

Besides those discussed here, other proteins were altered in the three *Col6a1^−/−^* muscles and the results are summarised in the Supplementary Fig. S2.

## Discussion

This study investigated protein alterations in three muscles (gastrocnemius, tibialis anterior and diaphragm) of *Col6a1^−/−^* mice, a model of human collagen VI myopathies. These muscles were selected since they are differently affected in *Col6a1^−/−^* mice [20], [21] and in human collagen VI myopathies [8], [13]–[19]. The *Col6a1^−/−^* mouse model is widely studied, as it resembles human Bethlem myopathy and UCMD. *Col6a1^−/−^* mice display a myopathic phenotype characterized by an early onset phenotype and very slow progression [20]. Therefore, a single time-point (i.e. 6-month-old mice) was used, as this represents the most characterized time-point for these mice [21], [22]. A limitation of the proteomic analysis is the inability to identify proteins in the range of pico- to femtomoles. We have, at least partially, overcome this limitation by performing immunoblotting and enzymatic assays on specific proteins. This careful design enabled the characterization of metabolic and structural protein alterations in the three muscles, thus providing a broader understanding of the pathological changes and of their connections to the pathophysiology of the collagen VI myopathies.

Based on the results of this study, the gastrocnemius muscle of *Col6a1^−/−^* mice is metabolically characterized by a slight decrement of the glycolytic flux, compensated by an increase of the TCA cycle, respiratory chain and SIRT3, resulting in an increased energy production and at least a partial activation of defense mechanisms against oxidative stress. The tibialis anterior muscle of *Col6a1^−/−^* mice has an overall similar metabolic profile to the gastrocnemius (except for the increase of SIRT3). However, the specific increases of PPARα and PGC1α in *Col6a1^−/−^* tibialis anterior suggest that this muscle compensates collagen VI deficiency (and its concomitant mitochondrial deficit) with increased biogenesis. At variance from gastrocnemius and tibialis anterior, diaphragm is characterized by a blunted glycolytic flux, TCA cycle and respiratory chain activity. This profile is associated to an increased α-ketoglutarate production by IDH1, which generates lipogenic citrate. Lipotoxicity and the resulting cell death are further sustained by an increment of PPARβ, triggering detrimental lipid signaling, and of SIRT1, sensing NADH imbalance and inhibiting glycolytic genes. Interestingly, metabolic changes are not accompanied by a fiber type switch, as indicated by the lack of changes in the myosin heavy chain (MyHC) isoforms composition (see supplementary Fig. S5). These results are in agreement with those of Irwin et al. [22] and suggest that, in collagen VI deficiency, metabolic changes are caused by an altered signal transduction and are not due to a change in fiber type distribution which occurs in ageing or unloading processes [64], [65].

We have provided the first analysis of the proteome of *Col6a1^−/−^* mice, which documents a set of changes in proteins that are involved in energy transformation, mitochondrial biogenesis and Ca^2+^ homeostasis (troponin I fast, aldolase, enolase 3, triose phosphate isomerase, creatine kinase, adenylate kinase 1, parvalbumin, PPARs, PGC1α),associated with changes of proteins involved in mechanotransduction at the myotendineous junction/costameric/sarcomeric level (TN-C, FAK, ROCK1). The observed changes are specific for each muscle type, suggesting that diaphragm is more impaired than tibialis anterior and gastrocnemius even before the onset of respiratory insufficiency.

Our results are in agreement with previous *in vivo* studies, reporting that diaphragm presents a higher frequency of degenerating fibers compared to other muscles in the *Col6a1^−/−^* animal model [20], [21] and with respiratory insufficiency phenotype observed in UCMD and BM patients [19].

The phenotypic changes of the three *Col6a1^−/−^* muscles demonstrate that the structural changes do compromise biochemical signaling (altered mechanotransduction), albeit to a different extent. In the gastrocnemius muscle, the structural changes at the thin filament level are balanced by the counteracting of de-adhesion activities, the increment of z-line proteins and the turnover of damaged fibers (Mybph increment). The energy demand is sustained by an increased capacity of the TCA cycle and by the oxidative production of α-ketoglutarate through IDH1. In contrast, the tibialis anterior muscle and even more the diaphragm muscle are unable to compensate for the specific structural changes, resulting in a more severe phenotype, with activation of lipid oxidation in tibialis anterior and activation of FASN and cell death process in the diaphragm. Our results are in agreement with the muscular changes reported in UCDM and BM patients [14], [15], [18]. Previous studies have reported that changes in mechanotransduction could result in (or at least contribute to) various human muscular diseases [66]. However, as far as we are aware, this is the first study describing alterations in the mechanotransduction, which triggers reductive TCA cycle leading in turn to lipogenic citrate production in a model of muscular dystrophy. Changes in the ECM-integrin-costameric system [67], a primary mechanical sensor of collagen VI deficiency, transduce altered signals to muscle fibers resulting in sarco/endoplasmic reticulum stress [22], [68], Ca^2+^ deregulation [69] and activation of signaling pathways including ROCK1 and FAK [60], [70], [71]. Altered mechanotransduction impacts on the localization of the protein-complexes and their correct functions, resulting in energy impairment. The changes in muscle phenotypes are strictly associated with the metabolic impairment (i.e. a decrease in glycolytic enzymes and altered TCA cycle, with diaphragm>tibialis anterior>gastrocnemius), as the precise subcellular localization of metabolic proteins (e.g. glycolytic enzymes and M-CK in the thin filament and Z-line) is critical for energy production and utilization [72]–[74].

Our results also suggest that muscle mass and tendon (myotendineous junction) ratio and the muscle morphology play a critical role in the selective response of individual muscles. Gastrocnemius, characterized by a high muscle mass and tendon ratio, is metabolically and structurally less impaired in *Col6a1^−/−^* mice than tibialis anterior and diaphragm. Tibialis anterior has a lower muscle mass and tendon ratio compared with gastrocnemius (but higher than diaphragm) and in *Col6a1^−/−^* mice is metabolically and structurally more impaired than gastrocnemius and less impaired than diaphragm. Furthermore, the mass to tendon ratio appears to be correlated with the incidence of centrally nuclei fibers in different muscles (triceps<femoralis<external respiratory muscles<tibialis<diaphragm) [20], [21], the diaphragm being the most affected muscle.

Finally, diaphragm is the most impaired muscle in *Col6a1^−/−^* mice. Importantly, tendons were reported to actively participate in mechanotransduction [75], [76] and to be dysfunctional in *Col6a1^−/−^* mice [25].

Gastrocnemius and tibialis anterior appear to counteract the biochemical changes induced by improper myofibril alignment, by increasing their capacity of energy transformation, thus balancing the reduction of the glycolytic flux and Ca^2+^ overload, and counteracting lipid signaling by recruiting all possible energy sources to produce NADH and ATP. This may result in buffering Ca^2+^ that, otherwise, cannot be taken up properly by the sarcoplasmic reticulum, as indicated by the decrement of parvalbumin [77], [78]. The oxidative production of energy, observed in gastrocnemius and tibialis anterior, results in the generation of intermediates that sustain the anabolic substrates production (Fig. S4, panel A). By contrast, in diaphragm, the myofibrils distribution and the higher contribution of the tendon, which expresses TN-C, exacerbates the mechanotransduction defects, leading to lipid signaling. The resulting lipotoxicity become predominant suggesting that the absence of collagen VI causes ECM structural changes, generating an improper alignment of myofibrils that impacts both on cell metabolism and mitochondria as final targets (Fig. S4, panel B).

### Overall Conclusions

Our study analyzed the muscle protein alterations in a mouse model of human collagen VI myopathies. Our study for the first time demonstrates the following points 1) In *Col6a1^−/−^* mouse model, the main changes identified can be grouped as metabolic and structural/contractile. 2) The metabolic changes occur at a cellular, not only at mitochondrial level. The gastrocnemius and tibialis anterior muscles of *Col6a1^−/−^* mice are characterized by an increased TCA cycle, respiratory chain and IDH1, whereas *Col6a1^−/−^* diaphragm is characterized by an increased FASN and activation of lipogenic citrate production through IDH1. 3) Overall, the metabolic changes are associated with the changes in muscle phenotype, generating an altered mechanotransduction. 4) Alterations in the mechanotransduction reveal that only a limited number of proteins (FAK, troponin I fast, aldolase, enolase 3, triose phosphate isomerase, creatine kinase, adenylate kinase 1, parvalbumin and IDH1) are similarly affected in all muscles suggesting that they may represent the potential markers and targets for pharmacological treatment. 5) The mass and tendon (myotendineous junction) ratio and the muscle morphology play a critical role in the severity of the muscle impairments.

## Supporting Information

Figure S1 **Validation of LC/MS/MS identified proteins by immunoblotting.** Immunoblotting of selected proteins (identified with proteomic) in gastrocnemius (A), tibialis anterior (B) and diaphragm (C) muscles from *Col6a1* ^−/−^ *versus* wild-type pooled samples, normalized against β-tubulin (β-Tub). Pkm2: pyruvate kinase isozymes M1/M2; Vdac1: voltage-dependent anion-selective channel protein 1; Aldoa: fructose-bisphosphate aldolase A; Uqcrc1: ubiquinol-cytochrome-c reductase complex core protein I; Aco2: aconitase 2; Sdha: succinate dehydrogenase complex, subunit A; Atp5b: ATP synthase, H+ transporting mitochondrial F1 complex, beta subunit; Ca3: carbonic anhydrase 3.
(TIF)

Figure S2 **Histograms of proteomics results of stress proteins and others.** Histograms of stress proteins and others differentially expressed in gastrocnemius (black bars), tibialis anterior (grey bars) and diaphragm (white bars) muscles. Isoforms of proteins significantly altered (Student’s t-test, *p*<0.01) are expressed as percent of spot volume variation in *Col6a1* ^−/−^ *versus* wild-type. (Fgg: fibrinogen, gamma polypeptide; Mb: myoglobin; Immt: inner membrane protein, mitochondrial; Phb: prohibitin; Vdac1: voltage-dependent anion-selective channel protein 1; Eif4a2: protein synthesis initiation factor 4; Selenbp1: Selenium-binding protein 1; Prdx3: peroxiredoxin-3; Hspd1: heat shock protein 1; Ca2: carbonic anhydrase 2; Ca3: carbonic anhydrase 3; Prdx1: peroxiredoxin-1; Sod1: superoxide dismutase [Cu-Zn]; Hspa8: heat shock protein 8; Hspb1: heat shock protein beta-1; Prdx6: peroxiredoxin-6; Psma6: proteasome subunit, alpha type 6; Pdia3: protein disulfide-isomerase A3).
(TIF)

Figure S3 **Representative skeletal muscle 2D map.** Proteins separation was performed on pH 3–10 NL 24 cm IPG strips in the first dimension and on 12%T-2.5%C PAGE gels in the second dimension. The identified and statistically changed spots in gastrocnemius, tibialis anterior and diaphragm of *Col6a1* ^−/−^ *vs.* wild-type mice are indicated by numbers. The protein names, the gene name and the AC number together with MS data are listed in table 1S.
(TIF)

Figure S4 **Schematic representation of the different α-ketoglutarate fate in muscles from** ***Col6a1*** **^−/−^ mice.** The direction of IDH1 reaction is controlled by alterations in TCA cycle fluxes leading to the production of anabolic substrates for gastrocnemius and tibialis anterior muscles (panel A), and lipotoxicity for diaphragm muscle (panel B).
(TIF)

Figure S5 **Myosin heavy chain isoforms composition.** SDS electrophoresis was performed on muscle samples using a discontinuous buffer system with a 4% stacking gel (pH 6.8) and a 37% glycerol, 6%T constant concentration running gel (pH 8.8). Samples were separated at 100 V, overnight. Gels were stained with SYPRO Orange (Molecular Probe) and scanned using a 570 nm emission filter on Typhoon laser scanner. Protein band quantification was achieved using Image Quant (Molecular Dynamics) software. Individual samples were run in duplicate; 2 mg protein extract was loaded per lane. Differences between groups were computed by Student’s t-test, the significativity level being set at p<0.01. A two-tail F-test was applied in order to verify the homoscedasticity of variances.
(TIF)

Table S1 **Mass spectrometry data. Protein identification in muscle tissue by PMF and MS/MS.** The spot number, the protein names, the gene name, the % of spot variation and the AC number together with MS data are listed.
(PDF)

Data S1 **Supplementary MS data.** Representative MALDI-ToF PMF spectra and MSMS sequence analysis from the fragmentation of a precursor ion of identified spots by MALDI-ToF/ToF mass spectrometer.
(PDF)
