# Supplementary figures and images for: Changes in Muscle Cell Metabolism and Mechanotransduction Are Associated with Myopathic Phenotype in a Mouse Model of Collagen VI Deficiency
Source: PLoS One. 2013 Feb 20;8(2):e56716. doi: 10.1371/journal.pone.0056716 (PMC3577731; doi:10.1371/journal.pone.0056716)

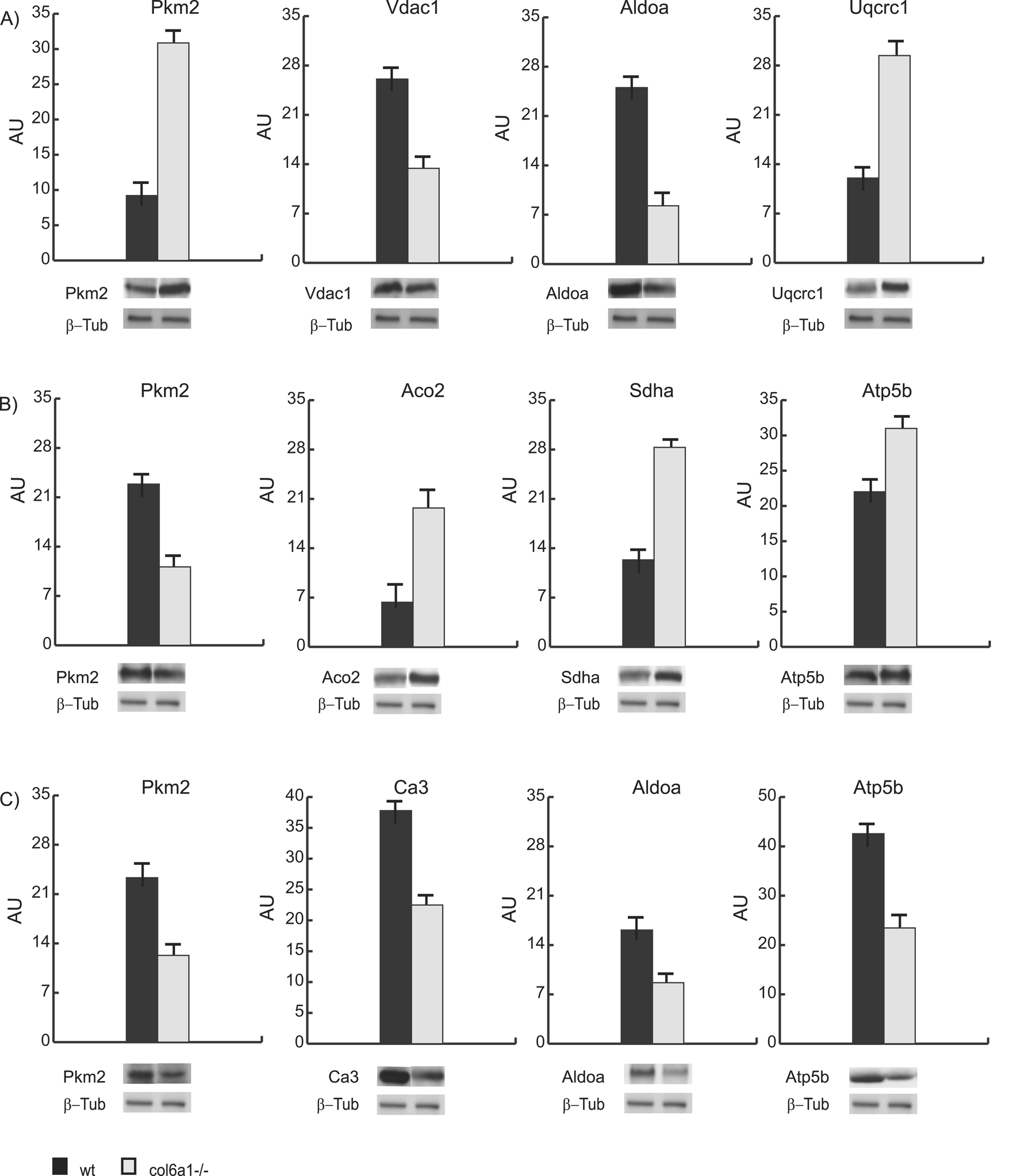

Supplement: Figure S1 — Validation of LC/MS/MS identified proteins by immunoblotting. Immunoblotting of selected proteins (identified with proteomic) in gastrocnemius (A), tibialis anterior (B) and diaphragm (C) muscles from Col6a1 −/− versus wild-type pooled samples, normalized against β-tubulin (β-Tub). Pkm2: pyruvate kinase isozymes M1/M2; Vdac1: voltage-dependent anion-selective channel protein 1; Aldoa: fructose-bisphosphate aldolase A; Uqcrc1: ubiquinol-cytochrome-c reductase complex core protein I; Aco2: aconitase 2; Sdha: succinate dehydrogenase complex, subunit A; Atp5b: ATP synthase, H+ transporting mitochondrial F1 complex, beta subunit; Ca3: carbonic anhydrase 3. (TIF) [file pone.0056716.s001.tif]

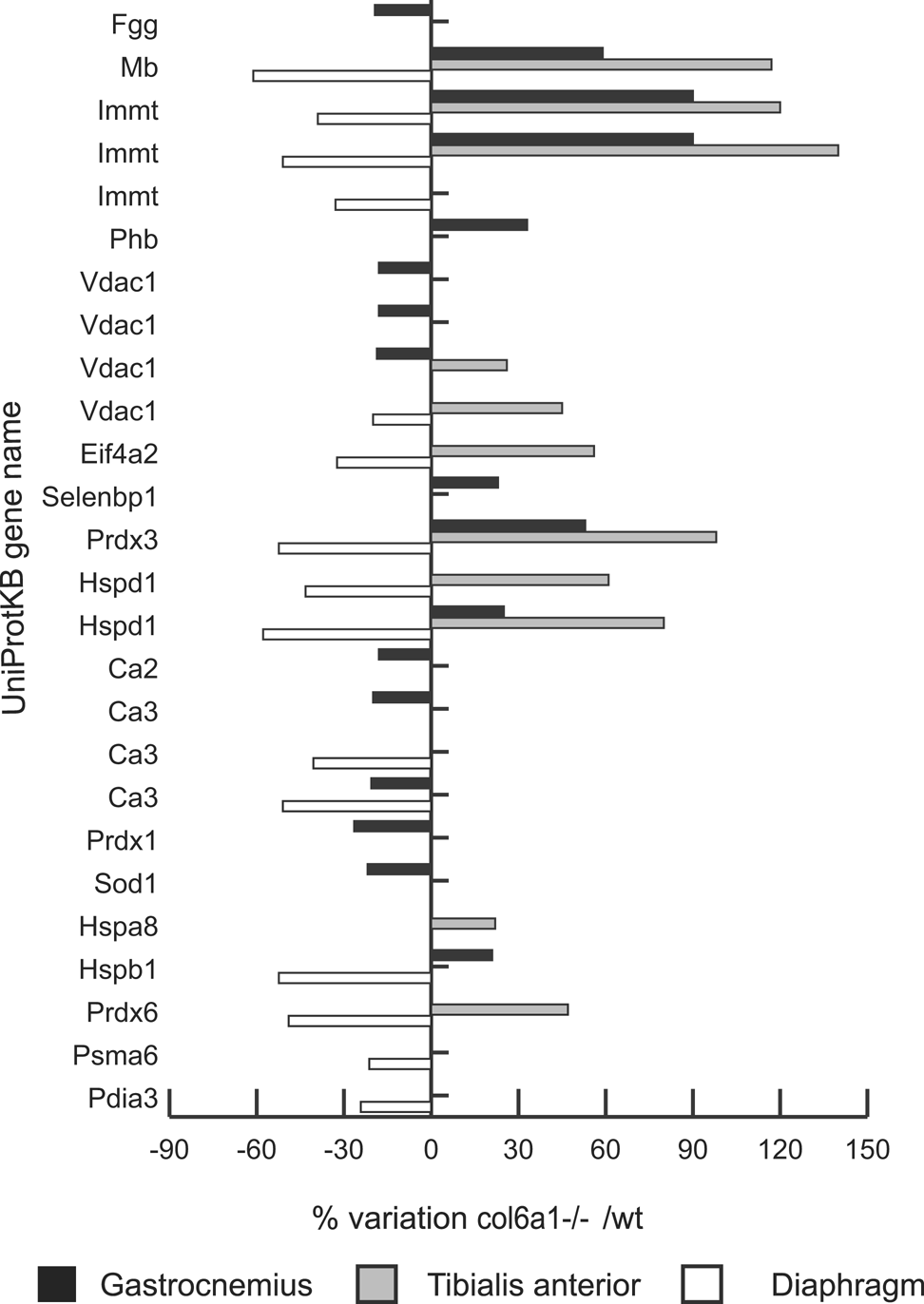

Supplement: Figure S2 — Histograms of proteomics results of stress proteins and others. Histograms of stress proteins and others differentially expressed in gastrocnemius (black bars), tibialis anterior (grey bars) and diaphragm (white bars) muscles. Isoforms of proteins significantly altered (Student’s t-test, p<0.01) are expressed as percent of spot volume variation in Col6a1 −/− versus wild-type. (Fgg: fibrinogen, gamma polypeptide; Mb: myoglobin; Immt: inner membrane protein, mitochondrial; Phb: prohibitin; Vdac1: voltage-dependent anion-selective channel protein 1; Eif4a2: protein synthesis initiation factor 4; Selenbp1: Selenium-binding protein 1; Prdx3: peroxiredoxin-3; Hspd1: heat shock protein 1; Ca2: carbonic anhydrase 2; Ca3: carbonic anhydrase 3; Prdx1: peroxiredoxin-1; Sod1: superoxide dismutase [Cu-Zn]; Hspa8: heat shock protein 8; Hspb1: heat shock protein beta-1; Prdx6: peroxiredoxin-6; Psma6: proteasome subunit, alpha type 6; Pdia3: protein disulfide-isomerase A3). (TIF) [file pone.0056716.s002.tif]

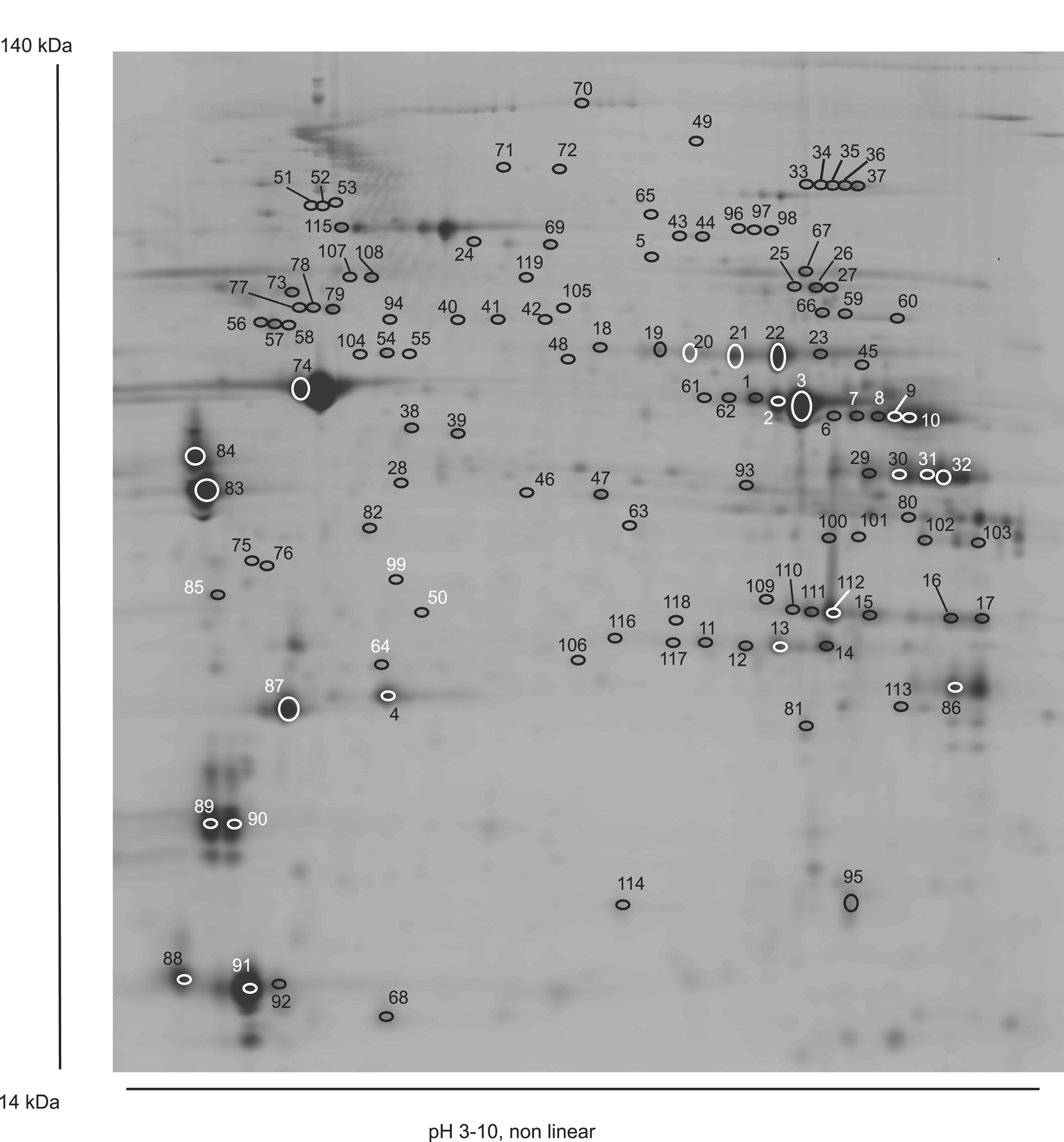

Supplement: Figure S3 — Representative skeletal muscle 2D map. Proteins separation was performed on pH 3–10 NL 24 cm IPG strips in the first dimension and on 12%T-2.5%C PAGE gels in the second dimension. The identified and statistically changed spots in gastrocnemius, tibialis anterior and diaphragm of Col6a1 −/− vs. wild-type mice are indicated by numbers. The protein names, the gene name and the AC number together with MS data are listed in table 1S. (TIF) [file pone.0056716.s003.tif]

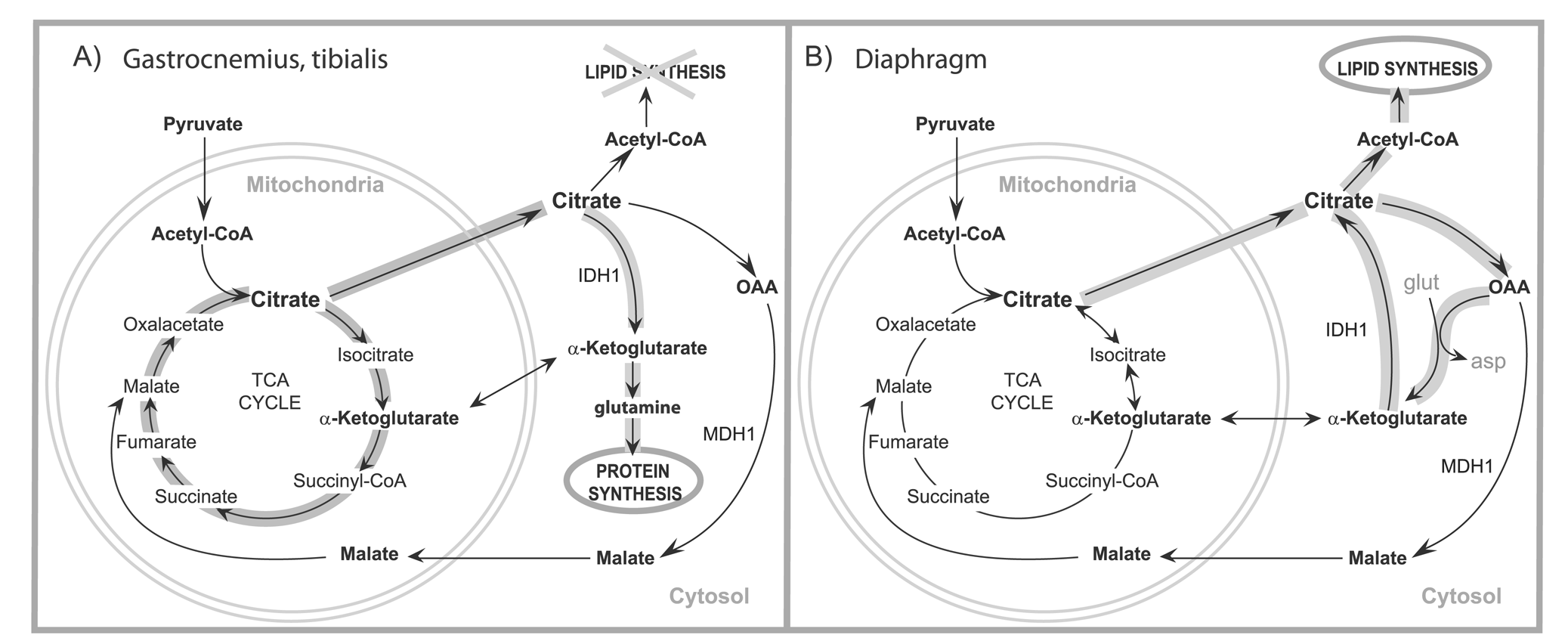

Supplement: Figure S4 — Schematic representation of the different α-ketoglutarate fate in muscles from Col6a1 −/− mice. The direction of IDH1 reaction is controlled by alterations in TCA cycle fluxes leading to the production of anabolic substrates for gastrocnemius and tibialis anterior muscles (panel A), and lipotoxicity for diaphragm muscle (panel B). (TIF) [file pone.0056716.s004.tif]

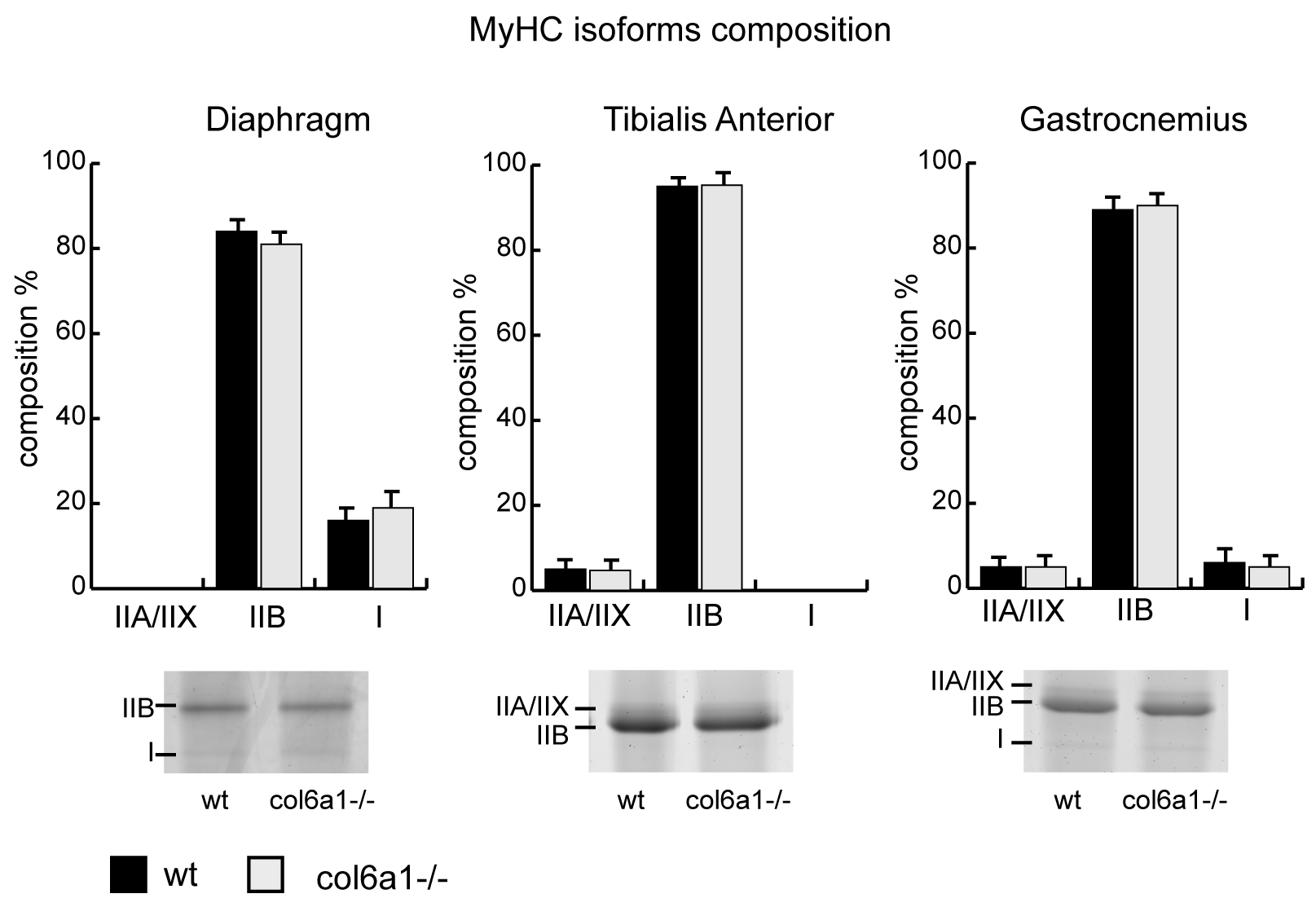

Supplement: Figure S5 — Myosin heavy chain isoforms composition. SDS electrophoresis was performed on muscle samples using a discontinuous buffer system with a 4% stacking gel (pH 6.8) and a 37% glycerol, 6%T constant concentration running gel (pH 8.8). Samples were separated at 100 V, overnight. Gels were stained with SYPRO Orange (Molecular Probe) and scanned using a 570 nm emission filter on Typhoon laser scanner. Protein band quantification was achieved using Image Quant (Molecular Dynamics) software. Individual samples were run in duplicate; 2 mg protein extract was loaded per lane. Differences between groups were computed by Student’s t-test, the significativity level being set at p<0.01. A two-tail F-test was applied in order to verify the homoscedasticity of variances. (TIF) [file pone.0056716.s005.tif]
