# Supplementary material for: Changes in Muscle Cell Metabolism and Mechanotransduction Are Associated with Myopathic Phenotype in a Mouse Model of Collagen VI Deficiency
Source: PLoS One. 2013 Feb 20;8(2):e56716. doi: 10.1371/journal.pone.0056716 (PMC3577731; doi:10.1371/journal.pone.0056716)
Supplement: Table S1 — Mass spectrometry data. Protein identification in muscle tissue by PMF and MS/MS. The spot number, the protein names, the gene name, the % of spot variation and the AC number together with MS data are listed. (PDF) [file pone.0056716.s006.pdf]

Table 1S. Protein identification in tissues by PMF and MS/MS.

Spots were identified by MALDI/MS. To confirm identification, a MS/MS spectrum per protein was collected by MALDI ToF/ToF as acceptance criterium.
* Indicates proteins identified by ESI-Ion trap mass spectrometer.

Spot n.	Protein name	Gene symbol	% variation gastrocnemius	% variation tibialis	% variation diaphragm	UniProt KB entry	Calculated MW	Calculated pI	MASCOT protein score	Coverage	Matched/searched peaks	MS/MS	MS/MS score	m/z	z
	**Energy transfer**														
1	Muscle creatine kinase	Ckm	-16.7	-27.5		P07310	43044.97	6.58	207	49.6	19/37	$_{224}$SFLVWVNEEDHLR$_{236}$	98	1643.856	1
2	Muscle creatine kinase	Ckm	-21.3	-23.1	-17.4	P07310	43044.97	6.58	175	36.5	14/24	$_{87}$DLFDPIIQDR$_{96}$	53	1231.642	1
3	Muscle creatine kinase	Ckm	-27.5		-34.2	P07310	43044.97	6.58	281	48.8	20/24	$_{87}$DLFDPIIQDR$_{96}$	41	1231.642	1
4	Adenylate kinase isoenzyme 1	Ak1	-29.6	-15	-48.5	Q9R0Y5	21539.60	5.67	115	58.1	11/29	$_{48}$YGYTHLSTGDLLR$_{60}$	106	1495.774	1
	**Glycolysis and glucose metabolism**														
5	Phosphoglucomutase 2	Pgm2	51.0		-37.5	Q7TNU0	63454.38	6.02	173	31.7	17/29	$_{31}$TQAYPDQKPGTSGLR$_{45}$	46	1618.821	1
6	Fructose-bisphosphate aldolase A	Aldoa	-18.0	-31.0		P05064	39224.74	8.40	131	36.8	13/37	$_{244}$FSNEEIAMATVTALR$_{258}$	105	1652.855	1
7	Fructose-bisphosphate aldolase A	Aldoa	-20.0	-30.6		P05064	39224.74	8.40	202	56.0	18/34	$_{244}$FSNEEIAMATVTALR$_{258}$	91	1652.836	1
8	Fructose-bisphosphate aldolase A	Aldoa	-16.7	-29.6		P05064	39224.74	8.40	122	31.3	12/26	$_{88}$ADDGRPFPQVIK$_{99}$	71	1342.703	1
9	Fructose-bisphosphate aldolase A	Aldoa		-24.8	-27.5	P05064	39224.74	8.40	275	58.0	21/29	$_{88}$ADDGRPFPQVIK$_{99}$	76	1342.696	1
10	Fructose-bisphosphate aldolase A	Aldoa		-17.4	-30.1	P05064	39224.74	8.40	188	56.6	17/34	$_{88}$ADDGRPFPQVIK$_{99}$	60	1342.714	1
11	Triosephosphate isomerase	Tpi		-28.6	-49.7	Q64513	26581.43	7.09	182	49.8	13/27	$_{7}$FFVGGNWK$_{14}$	40	954.479	1
12	Triosephosphate isomerase	Tpi		-27.5	-45.7	Q64513	26581.43	7.09	127	38.2	9/19	$_{176}$TATPQQAQEVHEK$_{188}$	43	1466.730	1
13	Triosephosphate isomerase	Tpi			-43.8	Q64513	26581.43	7.09	129	44.6	11/36	$_{7}$FFVGGNWK$_{14}$	60	954.486	1
14	Triosephosphate isomerase	Tpi	-21.3	-25.4	-48.5	Q64513	26581.43	7.09	116	38.6	10/33	$_{7}$FFVGGNWK$_{14}$	44	954.477	1
15	Phosphoglycerate mutase 2	Pgam2	-18.0			O70250	28695.91	8.65	83	43.9	8/29	$_{11}$HGESLWNQENR$_{21}$	87	1369.634	1
16	Phosphoglycerate mutase 2	Pgam2			-31.0	O70250	28695.91	8.65	100	34.4	10/28	$_{11}$HGESLWNQENR$_{21}$	83	1369.642	1
17	Phosphoglycerate mutase 2	Pgam2	-17.4		-40.1	O70250	28695.91	8.65	107	34.4	10/33	$_{11}$HGESLWNQENR$_{21}$	93	1369.639	1
18	Enolase 1 (alpha) *	Eno1		-24.8	-44.4	Q5XKE1	47140.82	6.37	705	34.6	12	$_{413}$IEEELGSK$_{420}$	39	452.7466	2
												$_{82}$VNVVEQEK$_{89}$	43	472.7881	2
												$_{336}$SCNCLLLK$_{343}$	35	504.2518	2
												$_{184}$IGAEVYHNLK$_{193}$	45	572.2824	2
												$_{61}$GVSQAVEHINK$_{71}$	29	591.3211	2
												$_{93}$LMIEMDGTENK$_{103}$	73	640.7659	2
												$_{82}$VNVVEQEKIDK$_{92}$	55	650.8480	2
												$_{16}$GNPTVEVDLYTAK$_{28}$	61	703.8650	2
												$_{359}$LAQSNGWGVMVSHR$_{372}$ + oxidation	28	519.9584	3
												$_{344}$VNQIGSVTESLQACK$_{358}$	90	817.4344	2
												$_{90}$IDKLMIEMDGTENK$_{103}$ + oxidation	26	557.2452	3
												$_{203}$DATNVGDEGGFAPNILENK$_{221}$	77	980.9988	2
19	Enolase 3 (beta, muscle)	Eno3		-21.9	-60.2	Q4FK59	46997.77	6.29	80	23.0	8/24	$_{359}$LAQSNGWGVMVSHR$_{372}$	87	1541.810	1
20	Enolase 3 (beta, muscle)	Eno3		-30.1	-49.5	Q4FK59	46997.77	6.29	191	36.2	14/18	$_{359}$LAQSNGWGVMVSHR$_{372}$	83	1541.784	1
21	Enolase 3 (beta, muscle)	Eno3		-31.0	-48.5	Q4FK59	46997.77	6.29	201	40.3	14/17	$_{359}$LAQSNGWGVMVSHR$_{372}$	88	1541.790	1
22	Enolase 3 (beta, muscle)	Eno3			-45.1	Q4FK59	46997.77	6.29	89	24.0	9/27	$_{359}$LAQSNGWGVMVSHR$_{372}$	67	1541.795	1
23	Enolase 3 (beta, muscle)	Eno3	-16.7	-23.1	-44.8	Q4FK59	46997.77	6.29	102	24.2	9/16	$_{359}$LAQSNGWGVMVSHR$_{372}$	49	1541.762	1
24	Dihydrolipoyllysine-residue acetyltransferase *	Dlat	-49.7			Q8BMF4	58778.08	5.70	761	34.1	12	$_{478}$ASALACLK.$_{485}$	36	417.2314	2
												$_{364}$GIDLTQVK$_{371}$	48	437.2714	2
												$_{383}$DIDSFVPSK$_{391}$	49	504.2949	2
												$_{469}$ISVNDFIIK$_{477}$	56	524.8334	2
												$_{633}$YLEKPITMLL.$_{642}$	47	610.9254	2
												$_{392}$AAPAAAAAMAPPGPR$_{406}$ + oxidation	54	668.3647	2
												$_{528}$GLETIASDVVSLASK$_{542}$	95	745.4792	2
												$_{282}$DVPLGAPLCIIVEK$_{295}$	38	762.4977	2
												$_{486}$VPEANSSWMDTVIR$_{499}$	97	802.9353	2
												$_{93}$VPLPSLSPTMQAGTIAR$_{109}$	70	870.0609	2
												$_{407}$VAPAPAGVFTDIPISNIR$_{424}$	33	613.3989	3
												$_{570}$NFSAIINPPQACILAIGASEDK$_{591}$	38	777.1493	3
25	Pyruvate kinase isozymes M1/M2	Pkm2	101.0	-20.0	-39.0	P52480	57713.70	7.42	110	32.0	13/39	$_{476}$DAVLNAWAEDVDLR$_{489}$	90	1586.822	1
26	Pyruvate kinase isozymes M1/M2	Pkm2	110.0	-13.8	-51.0	P52480	57713.70	7.42	161	43.8	15/29	$_{476}$DAVLNAWAEDVDLR$_{489}$	87	1586.776	1
27	Pyruvate kinase isozymes M1/M2	Pkm2			-37.1	P52480	57713.70	7.42	108	33.7	15/52	$_{476}$DAVLNAWAEDVDLR$_{489}$	80	1586.780	1
28	Pyruvate dehydrogenase (lipoamide) beta	Pdhb	20.0	67.0	-45.7	Q9D051	35768.29	5.39	128	42.3	16/30	$_{259}$EGIECEVINLR$_{269}$	75	1331.685	1
29	Glyceraldehyde-3-phosphate dehydrogenase	Gapdh			-22.5	Q569X2	35678.82	8.45	127	47,1	11/27	$_{308}$LISWYDNEYGYSNR$_{321}$	101	1779.828	1
30	Glyceraldehyde-3-phosphate dehydrogenase	Gapdh		-27.5	-18.0	Q569X2	35678.82	8.45	102	41.1	11/34	$_{308}$LISWYDNEYGYSNR$_{321}$	106	1779.842	1
31	Glyceraldehyde-3-phosphate dehydrogenase	Gapdh		-21.3	-18.0	Q569X2	35678.82	8.45	130	37.5	10/23	$_{308}$LISWYDNEYGYSNR$_{321}$	106	1779.836	1
32	Glyceraldehyde-3-phosphate dehydrogenase	Gapdh		-17.4	-35.5	Q569X2	35678.82	8.45	64	27,3	9/30	$_{308}$LISWYDNEYGYSNR$_{321}$	97	1779.836	1
	**TCA cycle**														
33	Aconitase 2, mitochondrial	Aco2		68.0		Q505P4	82463.88	7.40	298	36.4	25/33	$_{634}$NAVTQEFGPVPDTAR$_{648}$	131	1601.813	1
34	Aconitase 2, mitochondrial	Aco2	57.0	67.0		Q505P4	82463.88	7.40	315	43.3	30/47	$_{634}$NAVTQEFGPVPDTAR$_{648}$	111	1601.813	1
35	Aconitase 2, mitochondrial	Aco2	60.0	63.0		Q505P4	82463.88	7.40	287	35.1	26/36	$_{634}$NAVTQEFGPVPDTAR$_{648}$	120	1601.796	1

36	Aconitase 2, mitochondrial	Aco2	81.0	64.0		Q505P4	82463.88	7.40	179	28.6	19/32	634NAVTQEFGPVPDTAR648	104	1601.809	1
37	Aconitase 2, mitochondrial	Aco2	88.0	73.0		Q505P4	82463.88	7.40	246	39.4	25/44	634NAVTQEFGPVPDTAR648	110	1601.826	1
38	Isocitrate dehydrogenase [NAD] subunit alpha, mit prec	Idh3a		57.0	-32.9	Q9D6R2	36707.29	5.60	108	25.7	11/26	179IAEFAFEYAR188	77	1216.613	1
39	Isocitrate dehydrogenase [NAD] subunit alpha, mit prec	Idh3a	20.0	62.0	-34.2	Q9D6R2	36707.29	5.60	156	39.9	16/35	179IAEFAFEYAR188	70	1216.612	1
40	Dlst protein dihydrolipoamide S-succinyltransferase (E2 component of 2-oxo-glutarate complex)	Dlst	35.0	67.0	-42.9	Q8CIE8	41469.71	5.98	72	17.2	10/21	335NVETMNYADIER346	83	1454.662	1
41	Dlst protein dihydrolipoamide S-succinyltransferase (E2 component of 2-oxo-glutarate complex)	Dlst		35.0	-50.7	Q8CIE8	41469.71	5.98	96	17.2	9/12	335NVETMNYADIER346	70	1454.675	1
42	Dlst protein dihydrolipoamide S-succinyltransferase (E2 component of 2-oxo-glutarate complex)	Dlst			-29.1	Q8CIE8	41469.71	5.98	79	24.4	13/36	335NVETMNYADIER346	89	1454.672	1
43	Succinate dehydrogenase complex, subunit A	Sdha	37.0			Q921P5	68032.07	6.32	228	29.7	19/26	634VTLEYRPVIDK644	40	1332.767	1
44	Succinate dehydrogenase complex, subunit A	Sdha	34.0	99.0		Q921P5	68032.07	6.32	215	32.1	18/25	634VTLEYRPVIDK644	42	1332.764	1
45	Fumarate hydratase 1	Fh	32.0			Q3UIA9	54356.73	9.12	100	24.8	11/26	197GYSFVTTAER206	55	1130.556	1
46	Malate dehydrogenase, cytoplasmic	Mdh1	68.0	90.0	-38.3	P14152	36379.97	6.16	91	22.2	6/8	221GEFITTVQQR230	64	1178.620	1
47	Malate dehydrogenase, cytoplasmic	Mdh1	60.0	123.0	-56.1	P14152	36379.97	6.16	99	35.3	10/25	299FVEGLPINDFSR310	87	1393.706	1
48	Ornithine aminotransferase *	Oat	-18.0			P29758	45790.52	5.73	848	31.6	12	427ESVEIINK434	49	466.2799	2
												375GLLNAIVIR383	67	484.8339	2
												363LPSDVVTSVR372	60	536.8054	2
												67GIYMWDVEGR76 + oxidation	57	621.2719	2
												103SQVDKLTLTSR113	71	624.3806	2
												170IVFADGNFWGR180	61	641.3225	2
												359ELMKLPSDVVTSVR372 + oxidation	46	530.6417	3
												33TEQGPPSSEYIFER46	84	820.4159	2
												136VLPMNTGVEAGETACK151	92	838.9425	2
												32KTEQGPPSSEYIFER46	48	589.9675	3
												256HQVLFIADEIQTGLAR271	41	604.3498	3
												332IAIAALEVLEEENLAENADK351	102	1078.1261	2
49	Oxoglutarate dehydrogenase (lipoamide)	Ogdh		71.0	-17.4	Q5SVX9	111839.51	6.05	247	24.2	25/34	257STRFEEFLQR266	40	1312.666	1
	**Respiratory chain and ATP production**														
50	NADH dehydrogenase [ubiquinone] iron-sulfur protein 3, mit prec	Ndufs3	42.0	76.0	-51.9	Q9DCT2	26479.00	5.45	156	25.5	9/11	218VVAEPVELAQEFR230	99	1486.798	1
51	NADH dehydrogenase (ubiquinone) Fe-S protein 1	Ndufs1		64.0	-20.6	Q91VD9	77182.59	5.24	282	36.7	25/31	409FEAPLFNAR655	73	1064.564	1
52	NADH dehydrogenase (ubiquinone) Fe-S protein 1	Ndufs1		49.0	-21.9	Q91VD9	77182.59	5.24	330	35.4	30/36	292FAYDGLKR299	34	969.529	1
53	NADH dehydrogenase (ubiquinone) Fe-S protein 1 *	Ndufs1		73.0	-21.9	Q91VD9	77182.59	5.24	675	20.5	9	69LSVAGNCR76	36	438.7373	2
												451ILQDIASGR459	51	486.7840	2
												593SATYVNTEGR602	48	549.2812	2
												646LEEVSPNLVR655	50	578.3566	2
												88VVAACAMPVMK98 + oxidation	49	604.8396	2
												201GNDMQVGTYIEK212	67	677.8719	2
												429VALIGSPVDLTYR441	70	702.4741	2
												185FASEIAGVDDLGTTGR200	128	804.9834	2
												277MHEDINEEWISDK289	30	549.2786	3
54	Ubiquinol-cytochrome-c reductase complex core protein I, mit prec	Uqcrc1		86.0		Q9CZ13	49219.41	5.28	211	41.3	19/33	423RIPLAEWESR432	77	1256.666	1
55	Ubiquinol-cytochrome-c reductase complex core protein I, mit prec	Uqcrc1	129.0	89.0	-37.1	Q9CZ13	49219.41	5.28	154	35.0	15/33	423RIPLAEWESR432	53	1256.659	1
56	ATP synthase, H+ transporting mitochondrial F1 complex, beta subunit	Atp5b	106.0	120.0	-60.5	Q8CI65	51749.20	4.99	295	51.0	24/29	226AHGGYSVFAGVGER239	116	1406.668	1
57	ATP synthase, H+ transporting mitochondrial F1 complex, beta subunit	Atp5b	151.0	92.0		Q8CI65	51749.20	4.99	275	44.0	23/26	226AHGGYSVFAGVGER239	120	1406.692	1
58	ATP synthase, H+ transporting mitochondrial F1 complex, beta subunit	Atp5b		88.0	-55.0	Q8CI65	51749.20	4.99	212	43.7	22/30	226AHGGYSVFAGVGER239	111	1406.688	1
59	ATP synthase subunit alpha, mit prec	Atp5a	76.0			Q03265	55310.38	8.28	251	42.5	23/32	335EAYPGDVFYLHSR347	107	1553.748	1
60	ATP synthase subunit alpha, mit prec	Atp5a		113.0	-50.5	Q03265	55310.38	8.28	155	27.5	13/17	335EAYPGDVFYLHSR347	84	1553.757	1
	**Aminoacid/ lipid metabolism**														
61	Isovaleryl-CoA dehydrogenase, mitochondrial [Precursor]	Ivd		-32.0		Q9JHI5	42971.36	6.29	133	34.0	11/20	273GVYVLMSGLDLER285	73	1451.755	1
62	Isovaleryl-CoA dehydrogenase, mitochondrial [Precursor]	Ivd	-21.3	-27.0		Q9JHI5	42971.36	6.29	147	42.0	14/29	273GVYVLMSGLDLER285	73	1451.756	1
63	Delta(3,5)-Delta(2,4)-dienoyl-CoA isomerase, mitochondrial precursor	Ech1	-24.8			O35459	32433.09	6.01	89	28.7	9/22	65HVLHVQLNRPEK76	47	1469.863	1
64	Apolipoprotein A-I *	Apoa1	-38.3			Q58EV2	23022.10	6.99	481	37.1	7	157LQELQGR163	30	422.2635	2
												248TQVQSVIDK256	41	509.2901	2
												36DFANVYVDAVK46	61	620.8291	2
												184TQLAPHSEQMR194 + oxidation	45	657.3192	2
												206SNPTLNEYHTR216	58	666.3053	2
												142VAPLGAELQESAR154	87	670.8702	2
												34VKDFANVYVDAVK46	75	734.4286	2
65	Propionyl-CoA carboxylase alpha chain, mitochondrial precursor	Pcca	26.0			Q91ZA3	74429.33	6.04	184	28.0	17/25	413LSQYQEPIHLPGVR426	49	1636.884	1
66	Glutamate dehydrogenase 1, mitochondrial precursor	Glud1	46.0			P26443	55912.55	6.71	202	39.8	24/44	481HGGTIPVVPTAEFQDR496	101	1723.895	1
67	Delta-1-pyrroline-5-carboxylate dehydrogenase, mit prec.	Aldh4a1		84.0		Q8CHT0	59124.30	7.70	119	22.1	12/25	509STGSVVGQQPFGGAR523	76	1447.739	1
68	Fatty acid-binding protein, heart	Fabp3		160.0	-52.2	P11404	14687.66	6.15	115	62.9	9/23	67LGVEFDEITADDRK80	96	1607.791	1
	**Structural and contractile proteins**														
69	Myosin binding protein H	Mybph	123.4			P70402	52588.38	5.66	98	28.6	10/30	316TGQWFTVLER325	48	1236.651	1
70	Myosin binding protein C	Mybpc3	75.0	-55.2		A2AGQ1	141298.44	6.18	209	24.7	21/29	1169ALDFSEAPSFTQPLANR1185	75	1863.943	1
71	Gelsolin	Gsn	56.0			Q6PAC1	80762.59	5.52	80	15.5	8/18	127HVVPNEVVVQR137	42	1275.719	1
72	Gelsolin	Gsn	60.0			Q6PAC1	80762.59	5.52	70	12.2	7/15	176HVVPNEVVVQR186	56	1275.723	1
73	Vimentin	Vim	29.0			P20152	53556.48	5.06	330	57.4	27/36	94FANYIDKVR102	34	1125.606	1
74	Actin, alpha cardiac muscle 1	Actc1			-33.8	P68033	41784.64	5.23	91	26.4	9/22	197GYSFVTTAER206	70	1130.553	1

75	Actin, alpha cardiac muscle 1	Actc1	23.0			P68033	41784.64	5.23	92	48.3	7/16	31AVFPSIVGRPR41	34	1198.730	1
76	Actin, alpha cardiac muscle 1	Actc1	32.0			A1E281	41784.64	5.23	80	17.3	6/11	75IWHHTFYNELR85	67	1515.781	1
77	Desmin	Des	45.0			P31001	53366.59	5.21	293	59,3	23/33	355FASEANGYQDNIAR368	76	1555.685	1
78	Desmin	Des	37.0			P31001	53366.59	5.21	330	51.6	28/38	355FASEANGYQDNIAR368	102	1555.735	1
79	Desmin	Des	30.0			P31001	53366.59	5.21	291	61.0	25/34	150VAELYEEEMR159	49	1268.594	1
80	Myozenin 1 *	Myoz1			-42.9	Q9JK37	31457.28	8.57	747	54.7	10	235MTFQMPK241	36	441.7267	2
												28ESSGLNLGK36	34	452.7562	2
												18LIMELTGGGR27	65	523.8253	2
												221TAMPYGGYEK230 + oxidation	36	566.8028	2
												2PLSGTPAPNKR12	35	379.9136	3
												197VELGIDLLAYGAK209	86	681.4300	2
												43DVMLEELSLLTNR55	81	766.9457	2
												110GSSGGQAGSSGSAGQYGSDR129	87	886.9364	2
												90FLPTVGGQLETAGQGFSYGK109	122	1029.1402	2
												70FIYENHPDVFSDSSMDHFQK89	32	815.0896	3
81	Alpha-crystallin B chain	Cryab	91.0			P23927	20068.83	6.76	155	53.7	12/31	11RPFFPFHSPSR21	63	1374.727	1
82	Capping protein (actin filament) muscle Z-line, beta isoform a *	Capzb	-23.7	-28.1		A2AMV5	31345.47	5.47	444	26.7	8	216LVEDMENK223	43	489.2831	2
												261ELSQVLTQR269	41	537.3051	2
												15RLPPQQIEK23	45	370.2328	3
												226STLNEIYFGK235	52	586.3368	2
												58DYLLCDYNR66	58	616.3219	2
												216LVEDMENKIR225 + oxidation	27	631.9057	2
												182SGSGTMNLGGSLTR195	87	669.3455	2
												96LEVEANNAFDQYR108	93	784.9379	2
83	Tropomyosin 1 alpha chain	Tpm1	-23.7	-22.5		P58771	32680.56	4.69	167	36.3	15/30	92IQLVEEELDRAQER105	64	1727.886	1
84	Tropomyosin 2 beta chain	Tpm2	-33.3			P58774	32836.70	4.66	295	57.0	22/30	168KLVILEGELER178	93	1298.772	1
85	14-3-3 protein gamma	Ywhag	-25.9			P61982	28302.59	4.80	142	30.8	14/17	29NVTELNEPLSNEER42	117	1643.801	1
86	Troponin I, fast skeletal muscle	Tnni2	-32.9	-24.2	-17.4	P13412	21226.32	8.66	82	36.8	7/13	20SVMLQIAATELEKEESR36	140	1934.003	1
87	Myosin A1 catalytic light chain, skeletal muscle	Myl1			-48.7	P05977	20463.32	4.98	82	40.7	7/19	81DQGGYEDFVEGLR93	107	1484.666	1
88	Myosin A1 catalytic light chain, skeletal muscle	Myl1			-44.4	P05977	20463.32	4.98	91	52.7	8/25	81DQGGYEDFVEGLR93	115	1484.655	1
89	Myosin light chain, phosphorylatable, fast skeletal muscle	Mylpf	-30.6			P97457	18824.26	4.82	138	53.3	11/24	32EAFTVIDQNR41	78	1192.591	1
90	Myosin light chain, phosphorylatable, fast skeletal muscle	Mylpf	-34.2			P97457	18824.26	4.82	226	79.9	17/26	32EAFTVIDQNR41	78	1192.596	1
91	Parvalbumin alpha	Pvalb	-46.8	-54.8	-43.5	P32848	11799.33	5.02	110	48.2	8/19	15AIGAFAAADSFDHKK29	125	1548.775	1
92	Parvalbumin alpha	Pvalb	-44.1	-50.2	-60.2	P32848	11799.33	5.02	100	48.2	7/14	15AIGAFAAADSFDHKK29	125	1548.772	1
93	Troponin T, fast skeletal muscle *	Tnnt3	-22.5			Q9QZ47	32109.66	5.26	492	19.7	5	227YDITTLR233	37	441.2518	2
												55VDFDDIQK62	55	490.2648	2
												86EEEELIALK94	80	537.3056	2
												147ALSSMGANYSSYLAK161	93	781.9446	2
												49IPEGEKVDFDDIQK62	66	816.9321	2
	**Others**														
94	Fibrinogen, gamma polypeptide	Fgg	-19.4			Q8VCM7	46671.09	5.55	130	40.8	13/34	188ESGLYFIRPLK198	44	1322.752	1
95	Myoglobin	Mb	59.0	117.0	-61.2	P04247	16938.49	7.23	119	55.2	9/22	18VEADLAGHGQEVLIGLFK35	121	1896.025	1
96	Inner membrane protein, mitochondrial	Immt	90.0	120.0	-39.0	Q8CAQ8	83900.08	6.18	307	45.5	22/26	391FVNQLKGESR400	44	1177.636	1
97	Inner membrane protein, mitochondrial	Immt	90.0	140.0	-51.0	Q8CAQ8	83900.08	6.18	167	32.7	14/22	391FVNQLKGESR400	39	1177.628	1
98	Inner membrane protein, mitochondrial	Immt			-32.9	Q8CAQ8	83900.08	6.18	113	39.5	13/39	36VVSQYHELVVQAR48	61	1527.837	1
99	Prohibitin	Phb	33.0			P67778	29820.10	5.57	117	30.9	8/14	134FDAGELITQR143	52	1149.599	1
100	Voltage-dependent anion-selective channel protein 1	Vdac1	-18.0			Q60932	32351.49	8.55	159	51.6	12/30	225YQVDPDACFSAK236	97	1400.625	1
101	Voltage-dependent anion-selective channel protein 1	Vdac1	-18.0			Q60932	32351.49	8.55	132	47.3	10/26	225YQVDPDACFSAK236	73	1400.632	1
102	Voltage-dependent anion-selective channel protein 1	Vdac1	-18.7	26.0		Q60932	32351.49	8.55	106	32.9	8/20	225YQVDPDACFSAK236	56	1400.635	1
103	Voltage-dependent anion-selective channel protein 1 *	Vdac1		45.0	-20.0	Q60932	32351.49	8.55	442	30.4	5	257LTLSALLDGK266	70	515.8408	2
												164VTQSNFAVGYK174	84	607.3147	2
												225YQVDPDACFSAK236	85	700.8662	2
												97LTFDSSFSPNTGK109	80	700.9062	2
												33TKSENGLEFTSSGSANTETTK53	55	730.4079	3
104	Protein synthesis initiation factor 4A	Eif4a2		56.0	-32.4	P10630	46402.27	5.33	160	37.5	17/33	148LQAEAPHIVVGTPGR162	75	1544.888	1
105	Selenium-binding protein 1 *	Selenbp1	23.0			P17563	52514.02	5.87	1083	43.6	15	94LILPGLISSR103	77	534.8751	2
												121VIEASEIQAK130	87	544.3334	2
												280NAEGTWSVEK289	39	560.7905	2
												104IYVVDVGSEPR114	93	617.3664	2
												345LAGQIFLGGSIVR357	90	665.9549	2
												8CGPGYSTPLEAMK20	52	706.3631	2
												24EEIVYLPCIYR34	47	727.9347	2
												399LYATTSLYSAWDK411	81	759.9580	2
												161GSFVLLDGETFEVK174	72	770.9607	2
												383IPGGPQMIQLSLDGK397	81	777.4962	2
												212DGFNPAHVEAGLYGSR227	83	563.9719	3
												383IPGGPQMIQLSLDGKR398 + oxidation	31	576.0056	3
												35NTGTEAPDYLATVDVDPK52	101	953.5416	2
												255FLHDPSATQGFVGCALSSNIQR276	72	802.4705	3
												358GGSVQVLEDQELTCQPEPLVVK379	63	809.1486	3

106	Thioredoxin-dependent peroxide reductase, mitochondrial precursor	Prdx3	53.0	98.0	-52.4	P20108	21564.58	5.73	108	32.3	8/19	198HLSVNDLPVGR208	70	1206.666	1
107	Heat shock protein 1 (chaperonin)	Hspd1		61.0	-43.2	Q8C2C7	57925.78	5.35	104	28.6	11/29	61TVIIEQSWGSPK72	74	1344.730	1
108	Heat shock protein 1 (chaperonin)	Hspd1	25.0	80.0	-57.8	Q8C2C7	57925.78	5.35	83	18.7	8/21	61TVIIEQSWGSPK72	68	1344.720	1
109	Carbonic anhydrase 2	Ca2	-18.0			P00920	28901.36	6.48	128	51.5	12/32	213EPITVSSEQMSHFR226	125	1647.796	1
110	Carbonic anhydrase 3	Ca3	-20.0			P16015	29235.08	6.97	165	44.6	10/15	68VVFDDTYDR76	65	1129.522	1
111	Carbonic anhydrase 3	Ca3			-40.5	P16015	29235.08	6.97	92	30.0	7/17	68VVFDDTYDR76	58	1129.545	1
112	Carbonic anhydrase 3	Ca3	-20.6		-51.0	P16015	29235.08	6.97	118	53,1	10/36	68VVFDDTYDR76	69	1129.526	1
113	Peroxiredoxin-1	Prdx1	-26.5			P35700	22176.50	8.26	153	51.3	10/15	8IGYPAPNFK16	45	1006.563	1
114	Superoxide dismutase [Cu-Zn]	Sod1	-21.9			P08228	15811.48	6.03	81	27.9	5/12	11GDGPVQGTIHFEQK24	95	1512.744	1
115	Hspa8 protein	Hspa8		22.0		Q504P4	82463.88	7.40	229	36.2	26/42	300ARFEELNADLFR311	41	1480.785	1
116	Heat shock protein beta-1	Hspb1	21.0		-52.4	P14602	23013.85	6.12	139	43.5	9/17	29LFDQAFGVPR38	68	1149.610	1
117	Peroxiredoxin 6	Prdx6		47.0	-49.0	O08709	24739.45	5.72	206	44.2	13/14	42DFTPVCTTELGR53	87	1395.665	1
118	Proteasome (Prosome, macropain) subunit, alpha type 6 *	Psma6			-21.3	Q0VGS3	27372.43	6.35	434	29.3	6	12HITIFSPEGR21	57	578.8371	2
												22LYQVEYAFK30	67	580.8531	2
												44GKDCAVIVTQK54	65	609.8722	2
												31AINQGGLTSVAVR43	107	643.3854	2
												105YGYEIPVDMLCK116 + oxidation	46	752.4204	2
												72ITESIGCVMTGMTADSR88 + oxidation	84	923.0270	2
119	Protein disulfide-isomerase A3 *	PDIA3			-24.2	P27773	54267.36	5.69	657	28.3	9	297LNFAVASR304	67	439.2706	2
												153DASVVGFFR161	48	499.3167	2
												95YGVSGYPTLK104	53	542.8251	2
												336FVMQEEFSR344	49	586.8332	2
												108DGEEAGAYDGPR119	87	618.7702	2
												184FAHTNIESLVK194	35	420.2534	3
												449GFPTIYFSPANK460	89	671.4003	2
												162DLFSDGHSEFLK173	45	697.9181	2
												259DLLTAYYDVDYEK271	88	804.4553	2
