# Supplementary material for: Changes in Muscle Cell Metabolism and Mechanotransduction Are Associated with Myopathic Phenotype in a Mouse Model of Collagen VI Deficiency
Source: PLoS One. 2013 Feb 20;8(2):e56716. doi: 10.1371/journal.pone.0056716 (PMC3577731; doi:10.1371/journal.pone.0056716)
Supplement: Data S1 — Supplementary MS data. Representative MALDI-ToF PMF spectra and MSMS sequence analysis from the fragmentation of a precursor ion of identified spots by MALDI-ToF/ToF mass spectrometer. (PDF) [file pone.0056716.s007.pdf]

SPOT 1

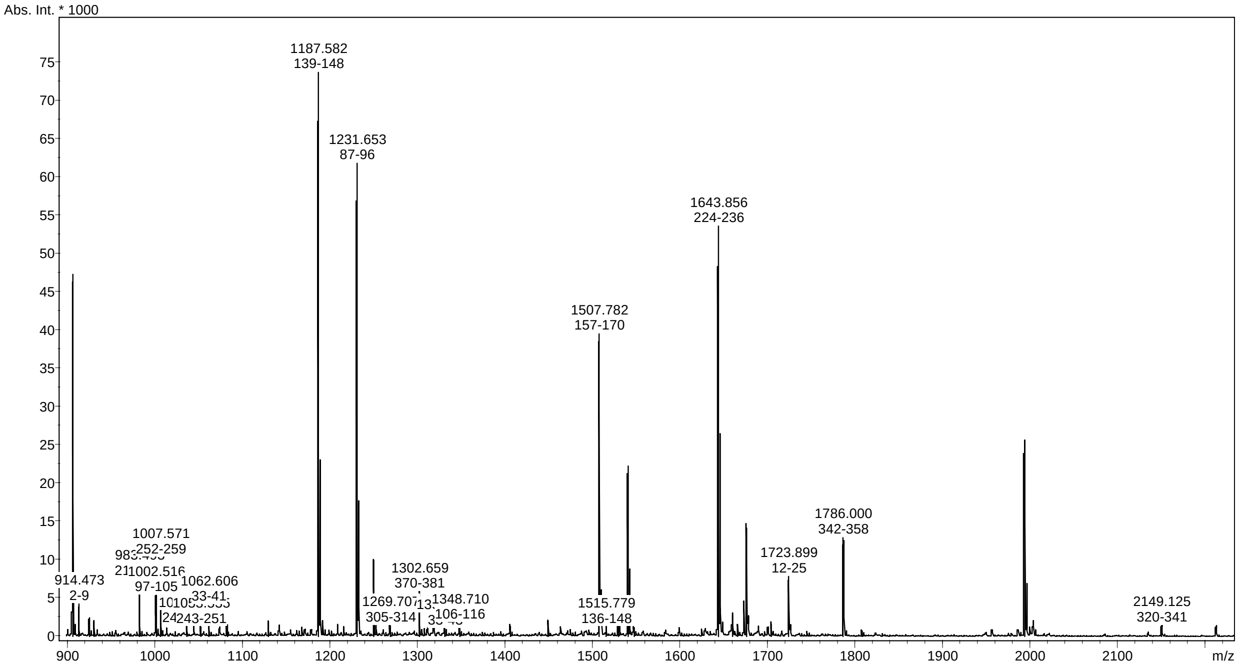

A representative MALDI-ToF PMF spectrum of spot 1

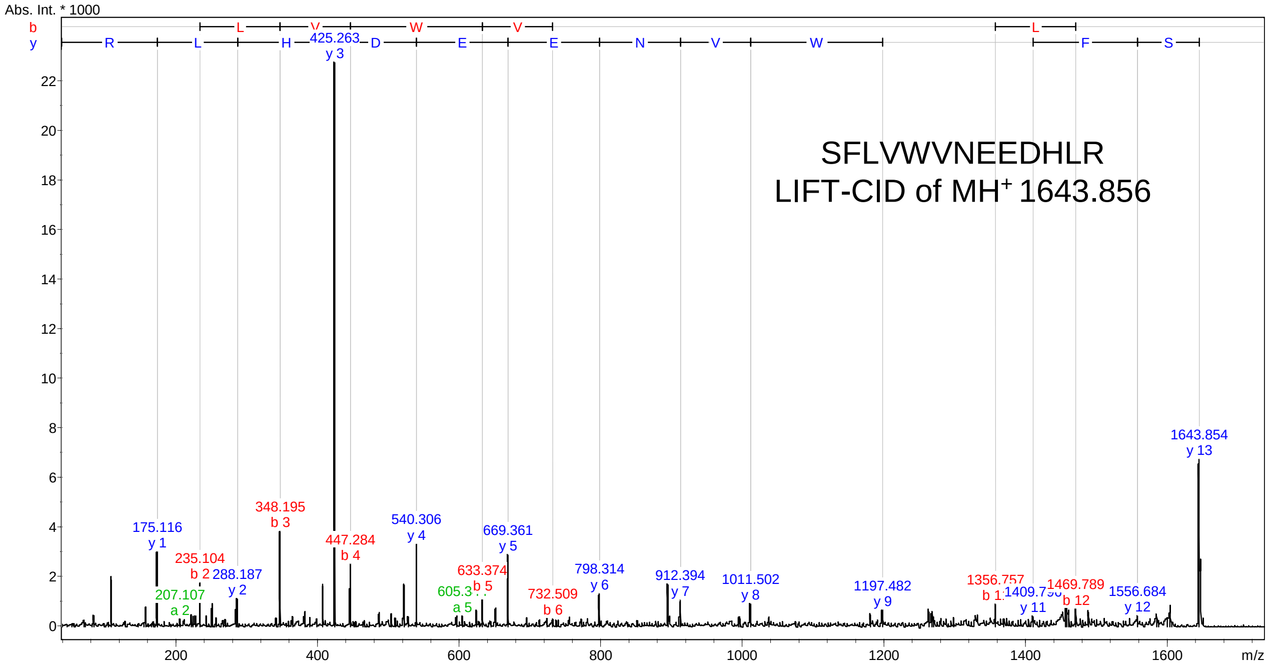

MSMS sequence analysis from the fragmentation of a precursor ion m/z 1643.856 by MALDI-ToF/ToF mass spectrometer

SPOT 2

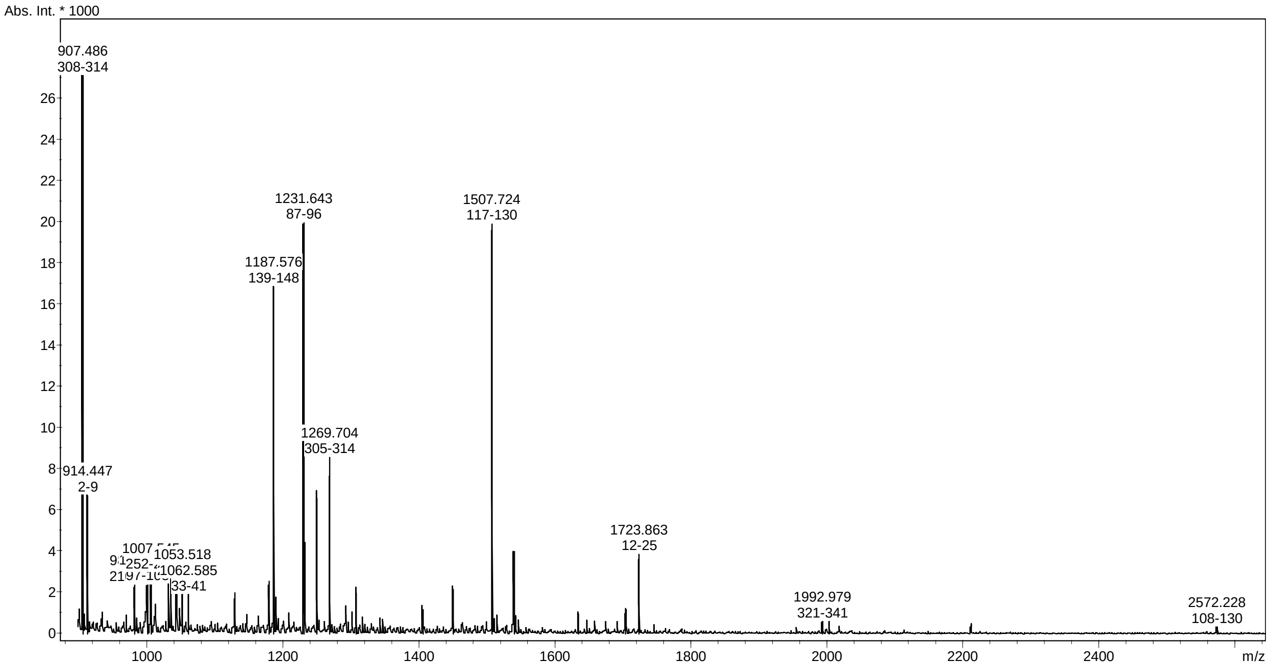

A representative MALDI-ToF PMF spectrum of spot 2

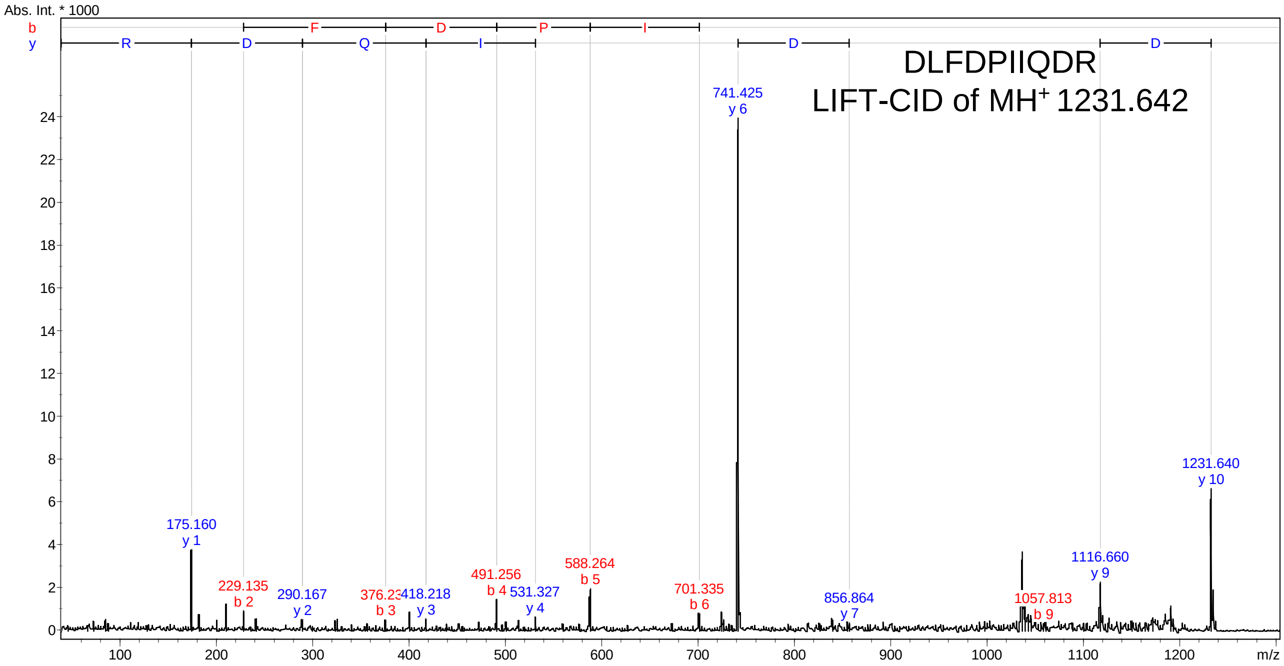

MSMS sequence analysis from the fragmentation of a precursor ion m/z 1231.642 by MALDI-ToF/ToF mass spectrometer

SPOT 3

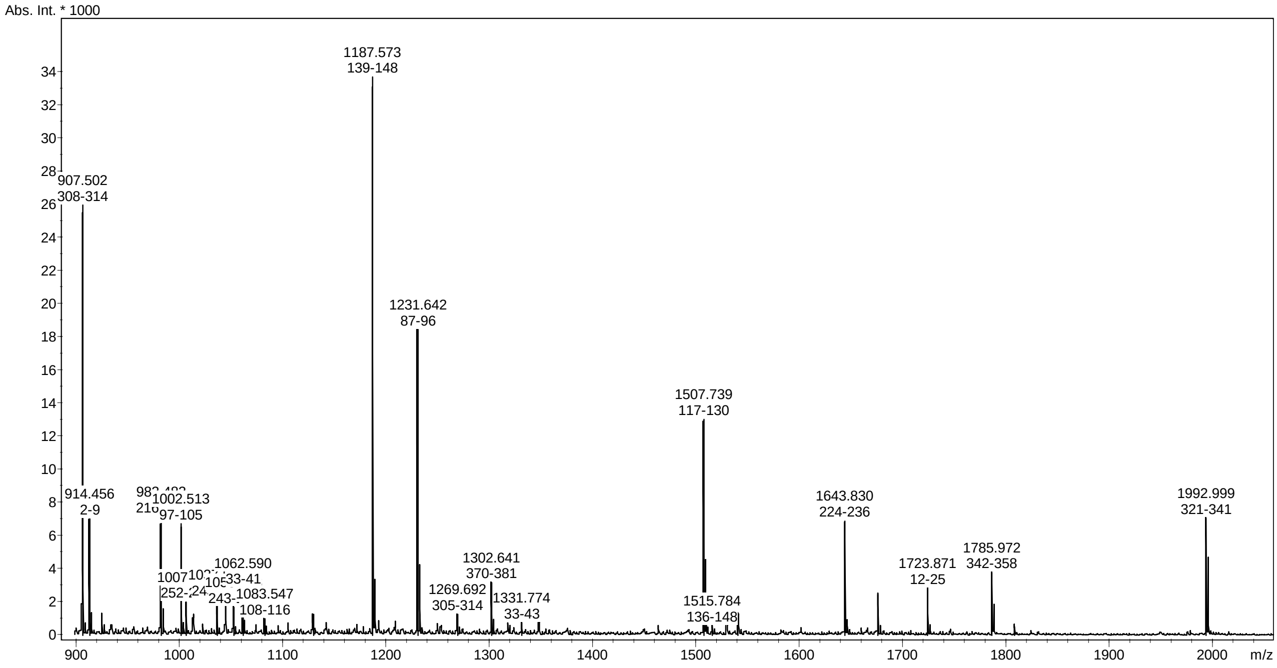

A representative MALDI-ToF PMF spectrum of spot 3

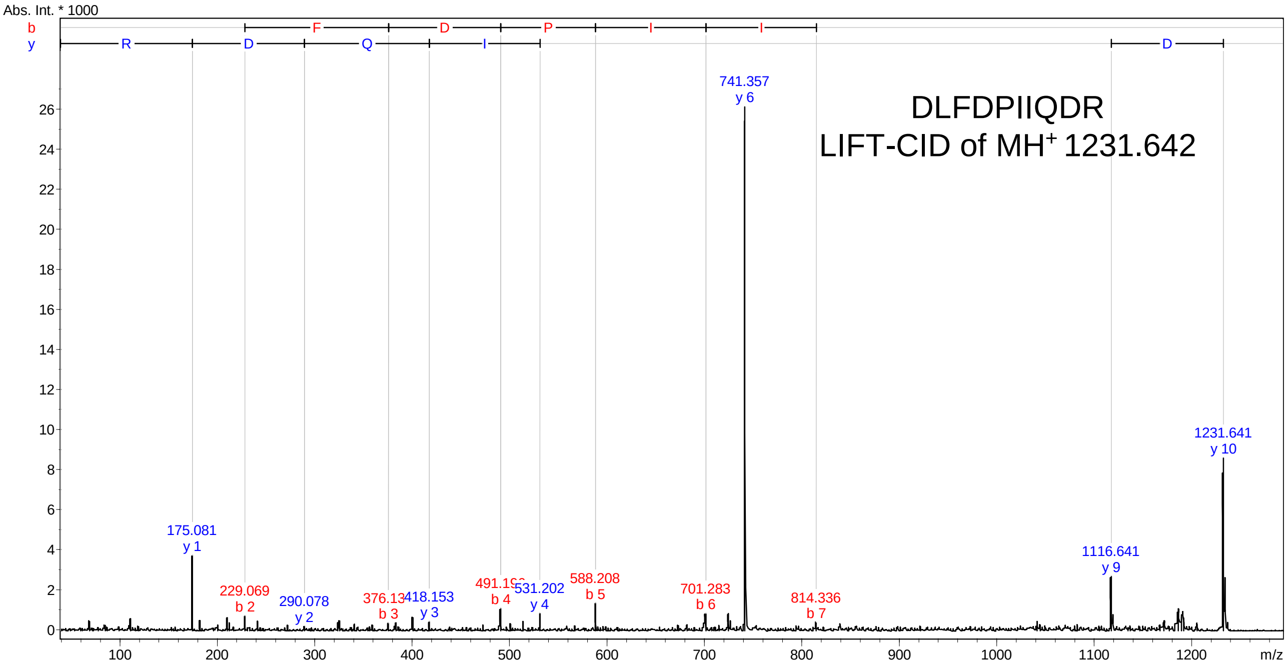

MSMS sequence analysis from the fragmentation of a precursor ion m/z 1231.642 by MALDI-ToF/ToF mass spectrometer

SPOT 4

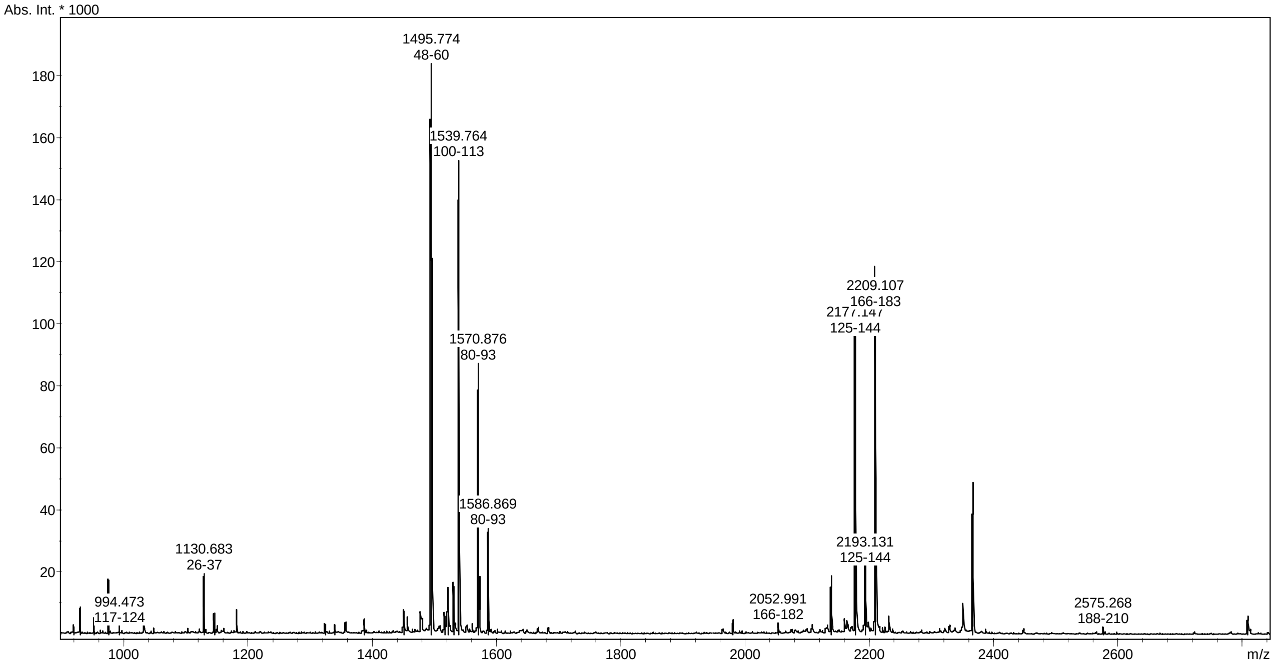

A representative MALDI-ToF PMF spectrum of spot 4

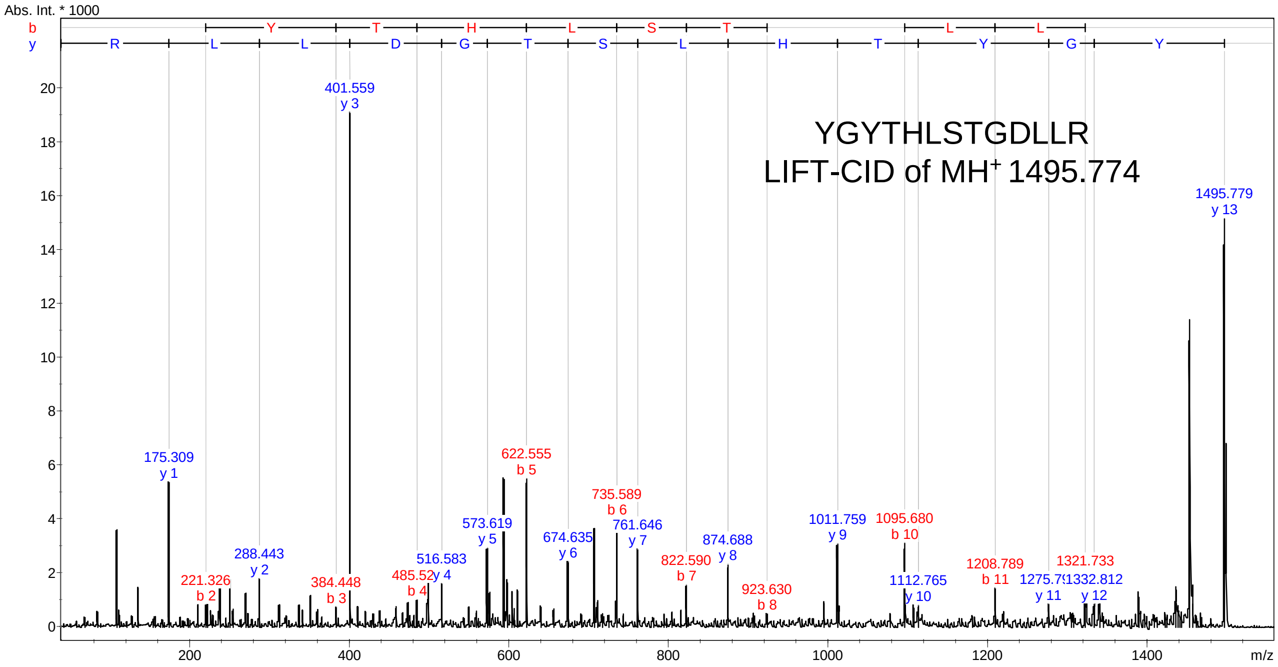

MSMS sequence analysis from the fragmentation of a precursor ion m/z 1495.774 by MALDI-ToF/ToF mass spectrometer

SPOT 5

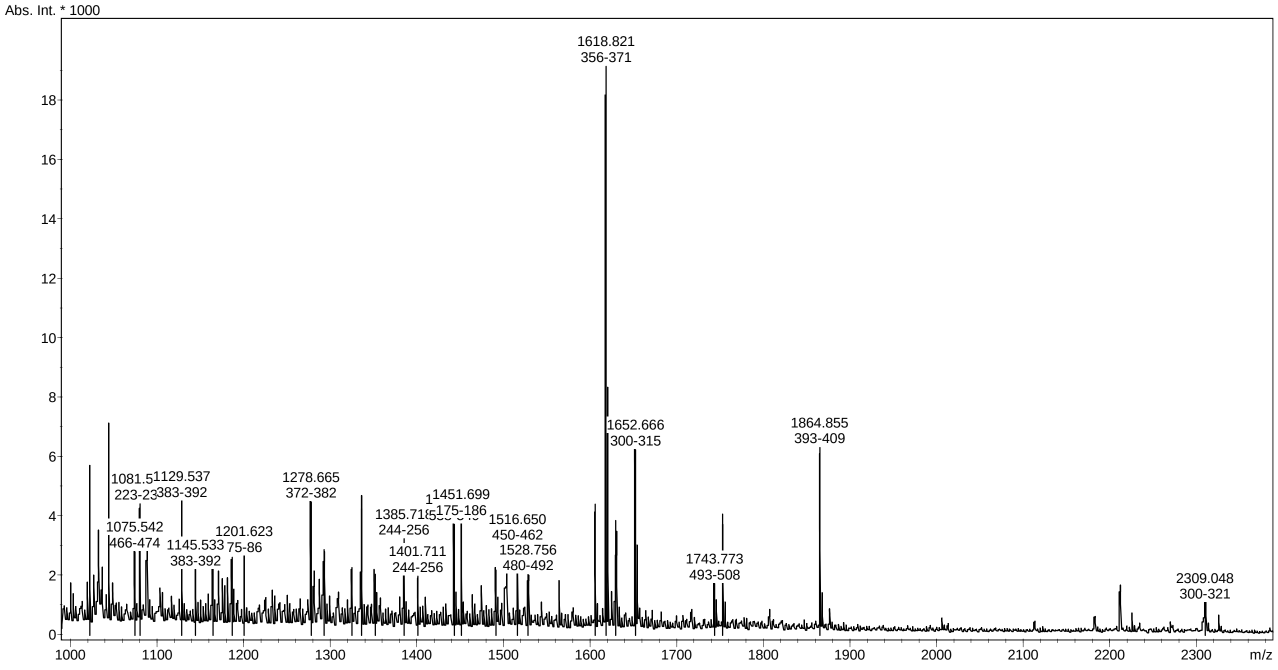

A representative MALDI-ToF PMF spectrum of spot 5

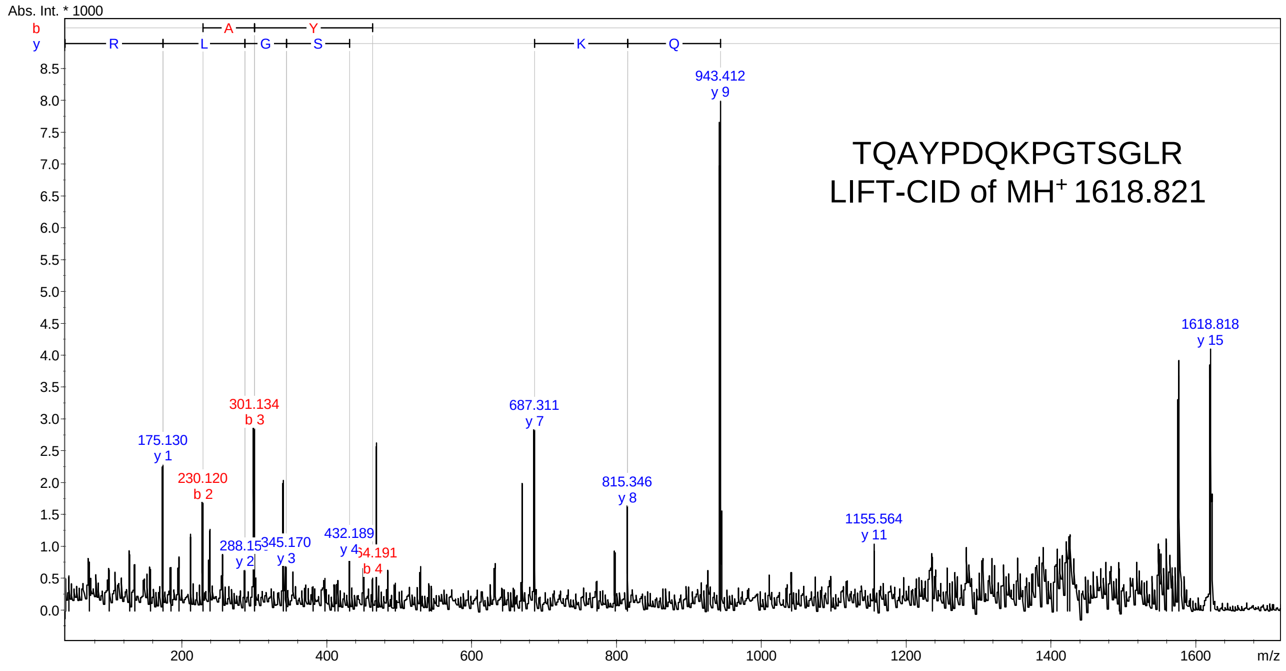

MSMS sequence analysis from the fragmentation of a precursor ion m/z 1618.821 by MALDI-ToF/ToF mass spectrometer

SPOT 6

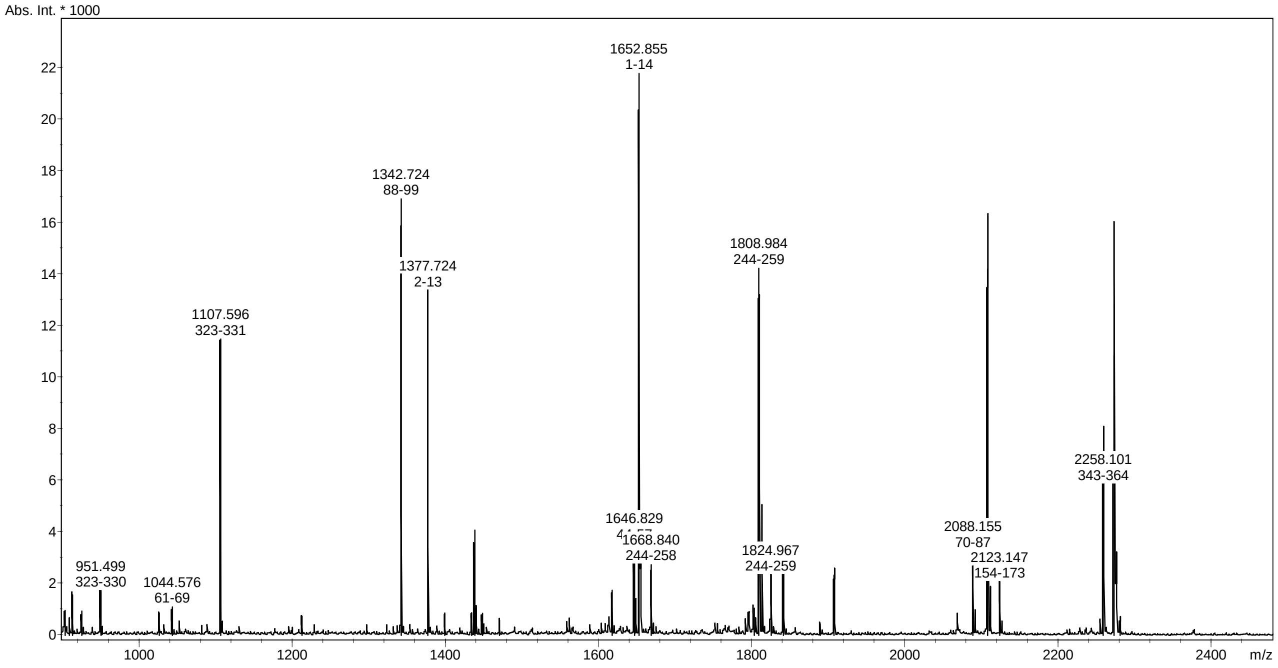

A representative MALDI-ToF PMF spectrum of spot 6

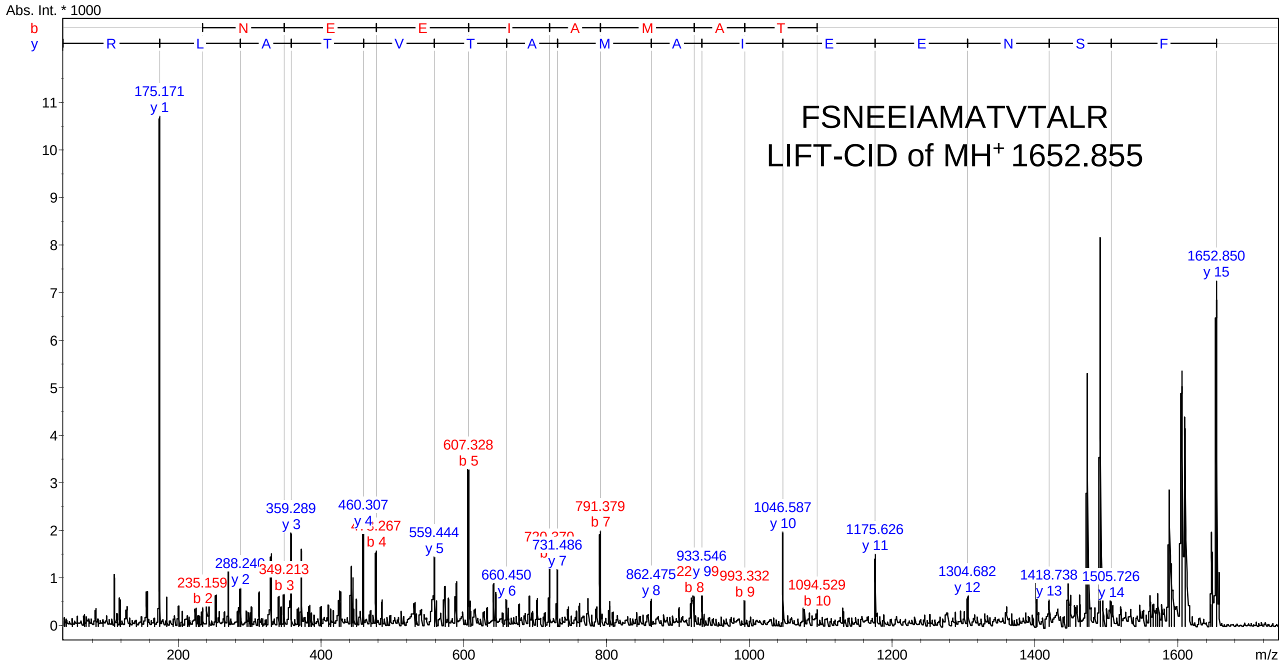

MSMS sequence analysis from the fragmentation of a precursor ion m/z 1652.855 by MALDI-ToF/ToF mass spectrometer

SPOT 7

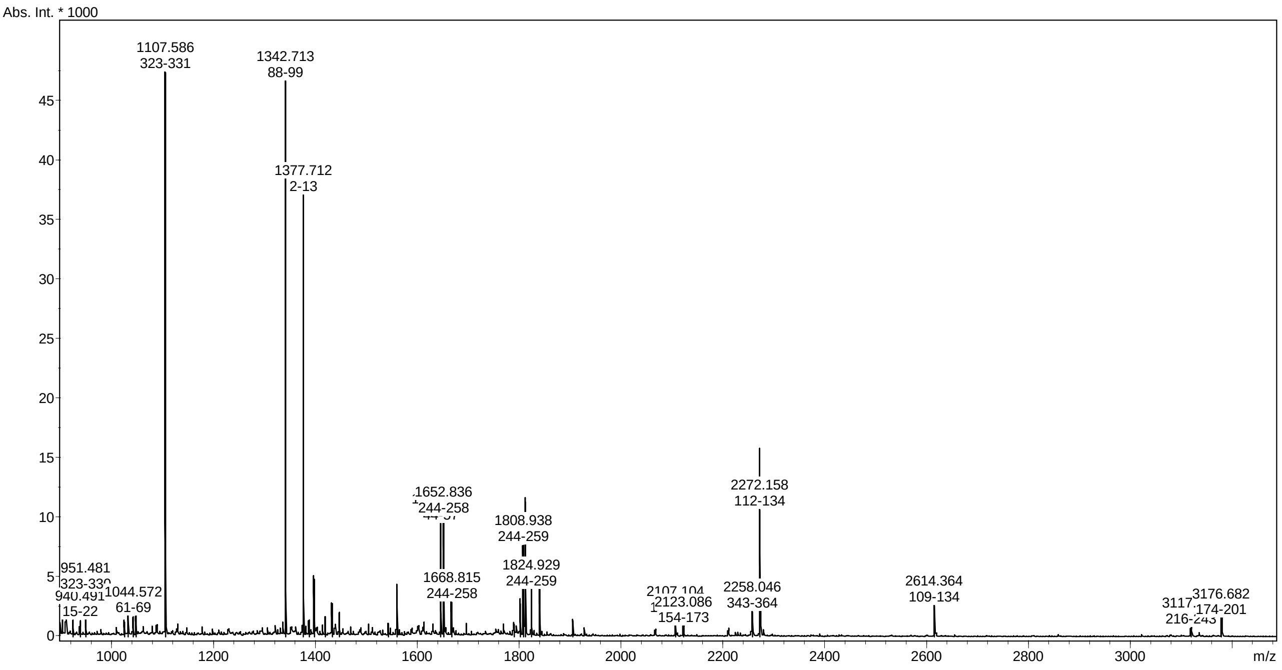

A representative MALDI-ToF PMF spectrum of spot 7

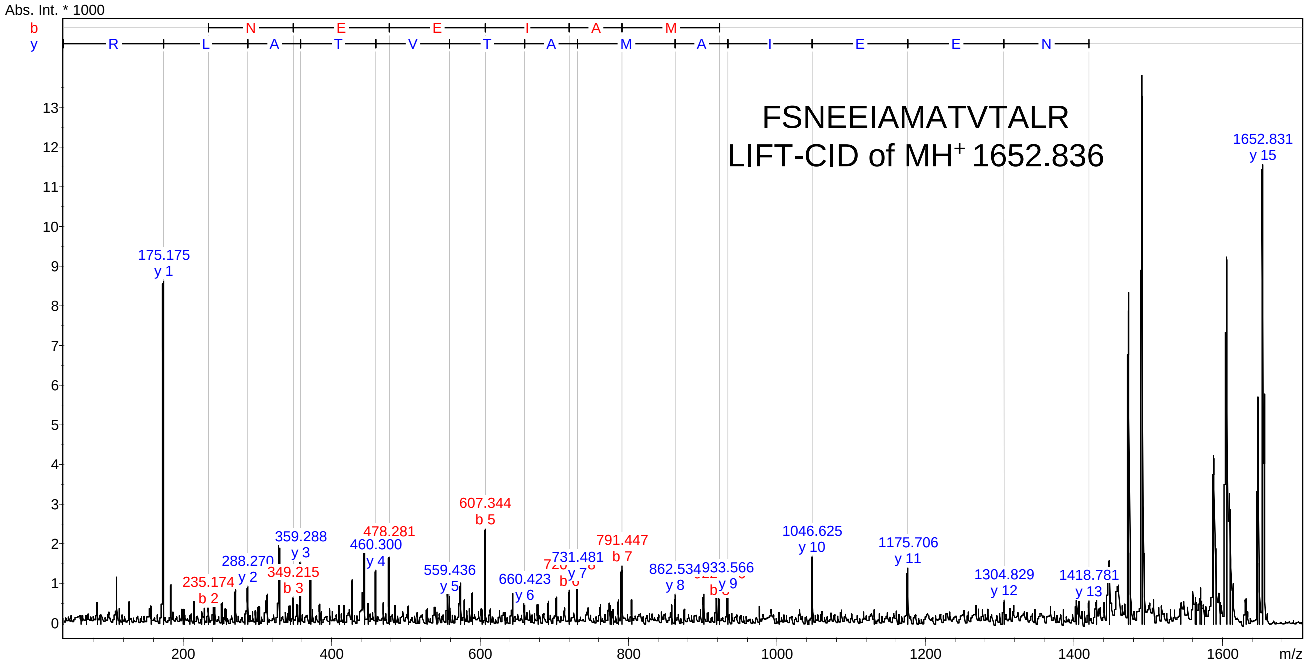

MSMS sequence analysis from the fragmentation of a precursor ion m/z 1652.836 by MALDI-ToF/ToF mass spectrometer

SPOT 8

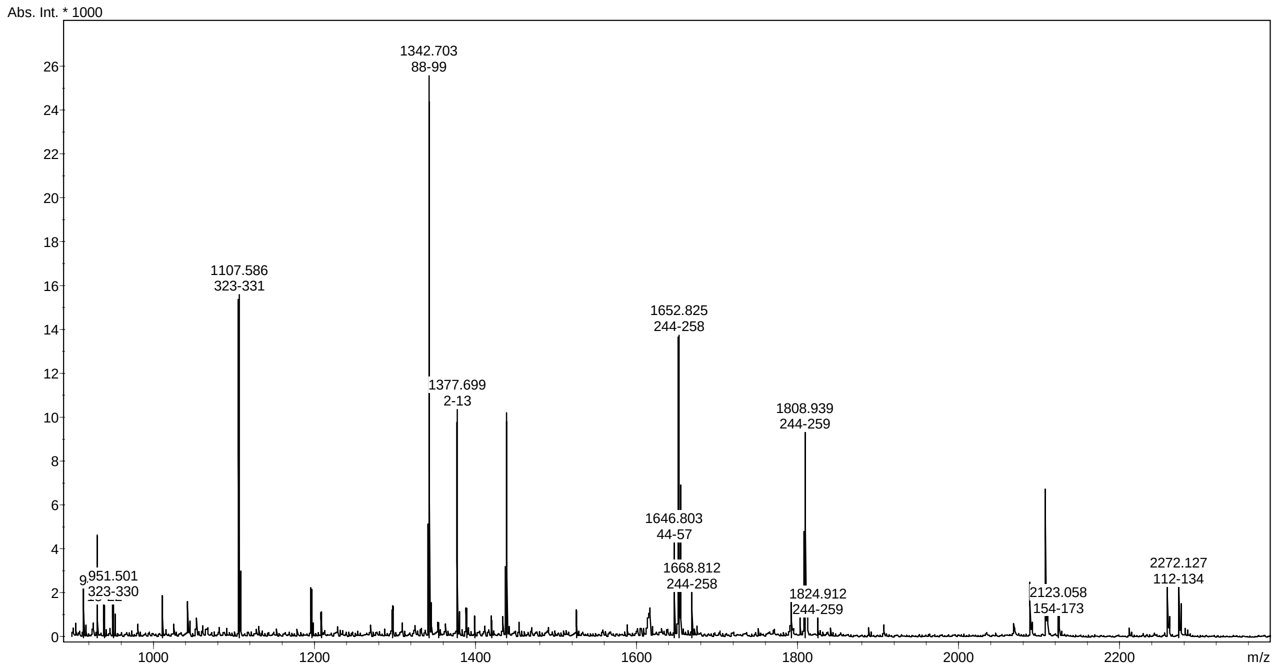

A representative MALDI-ToF PMF spectrum of spot 8

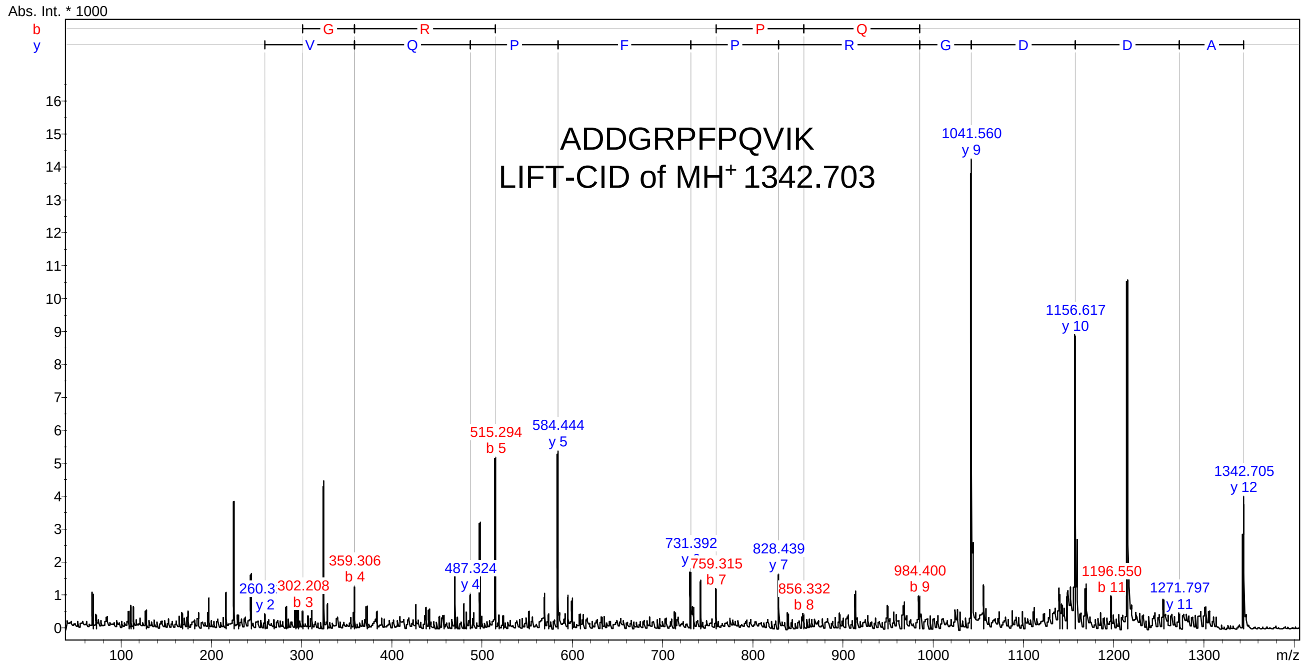

MSMS sequence analysis from the fragmentation of a precursor ion m/z 1342.703 by MALDI-ToF/ToF mass spectrometer

SPOT 9

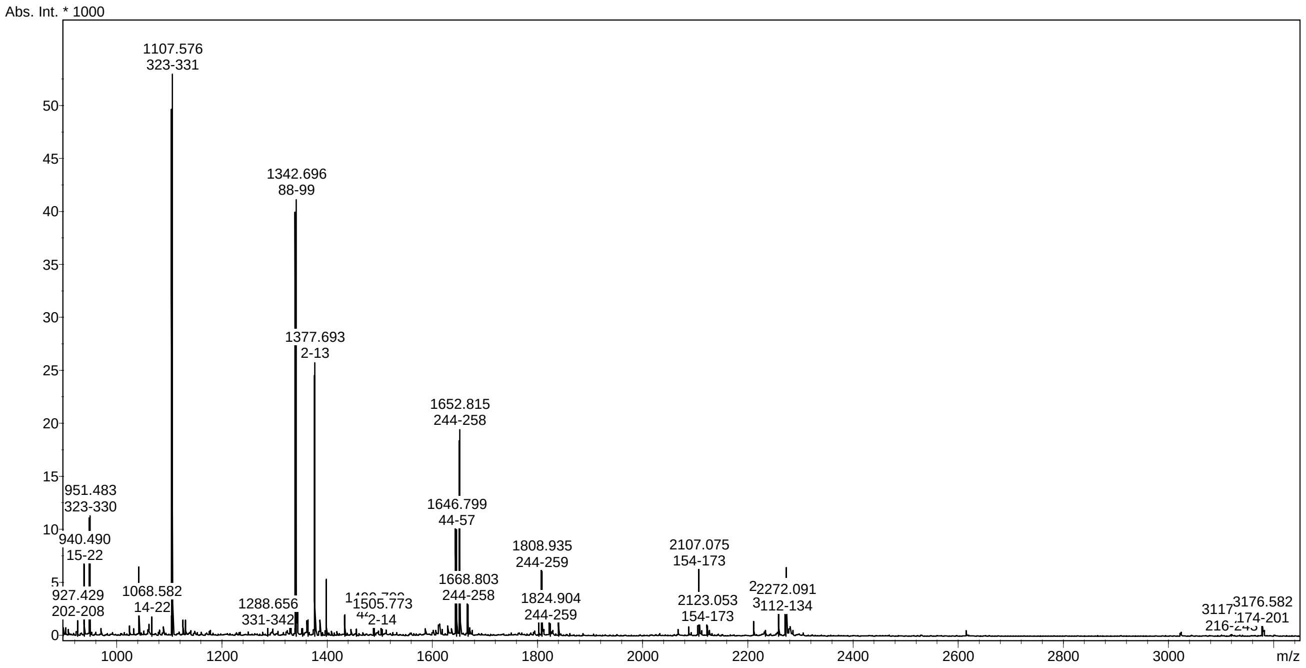

A representative MALDI-ToF PMF spectrum of spot 9

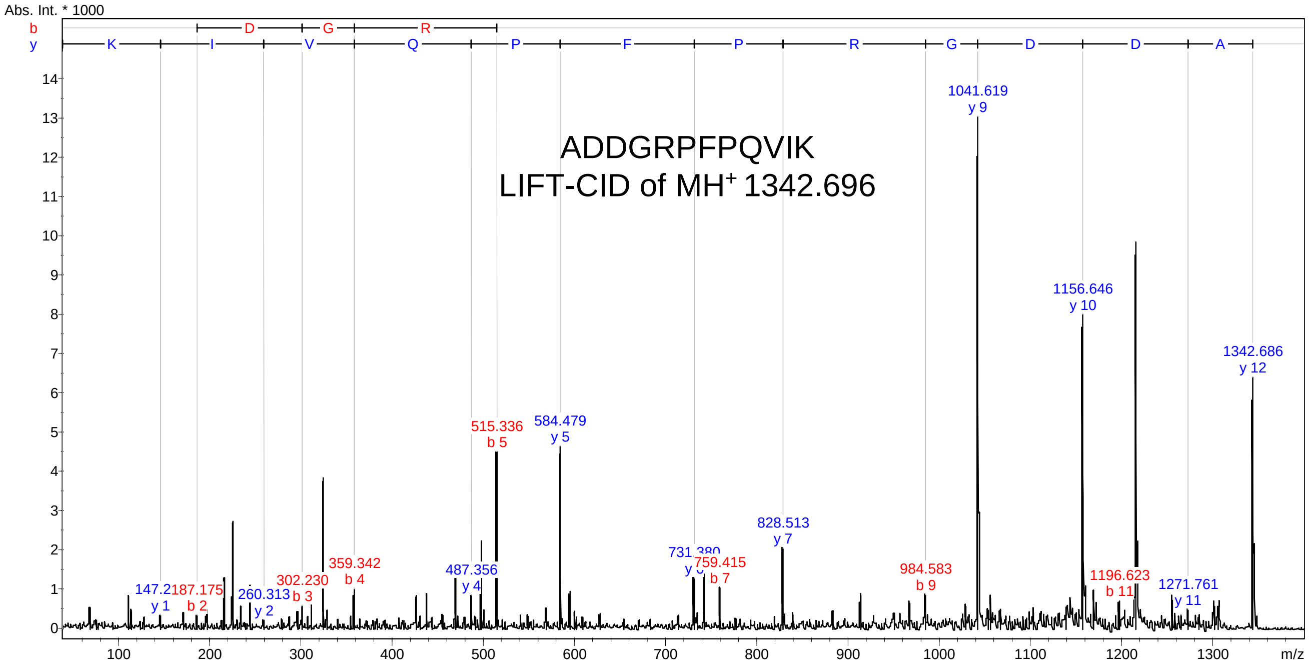

MSMS sequence analysis from the fragmentation of a precursor ion m/z 1342.696 by MALDI-ToF/ToF mass spectrometer

SPOT 10

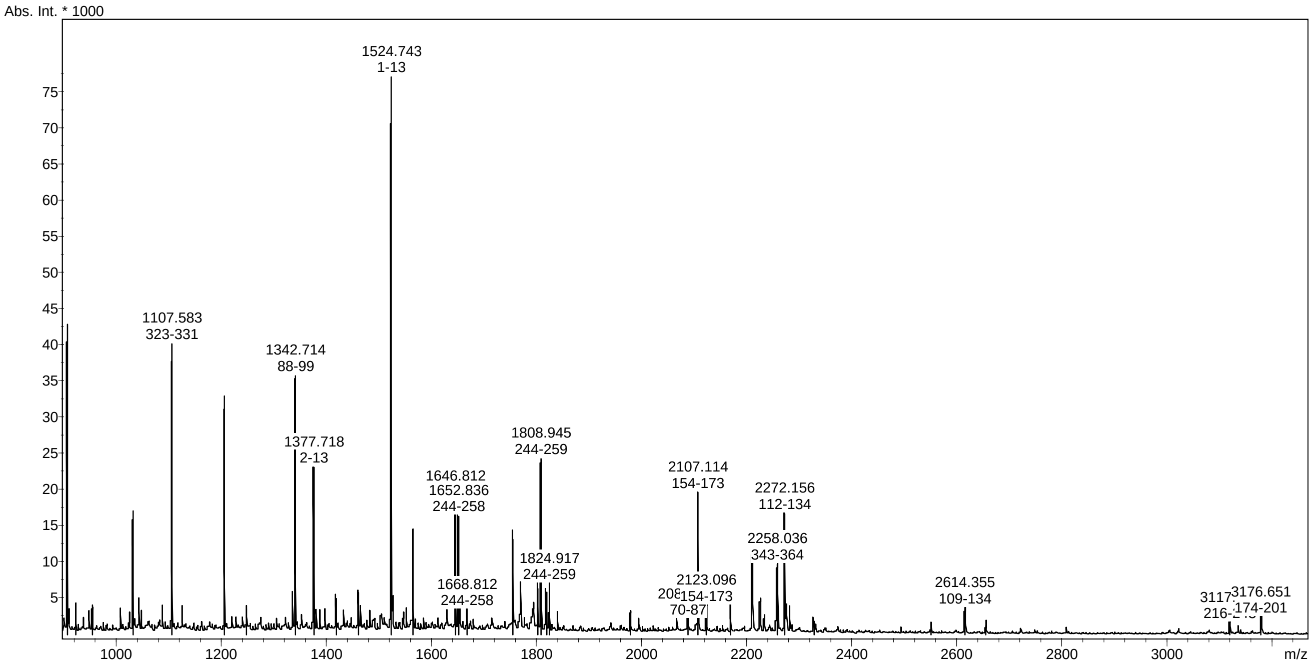

A representative MALDI-ToF PMF spectrum of spot 10

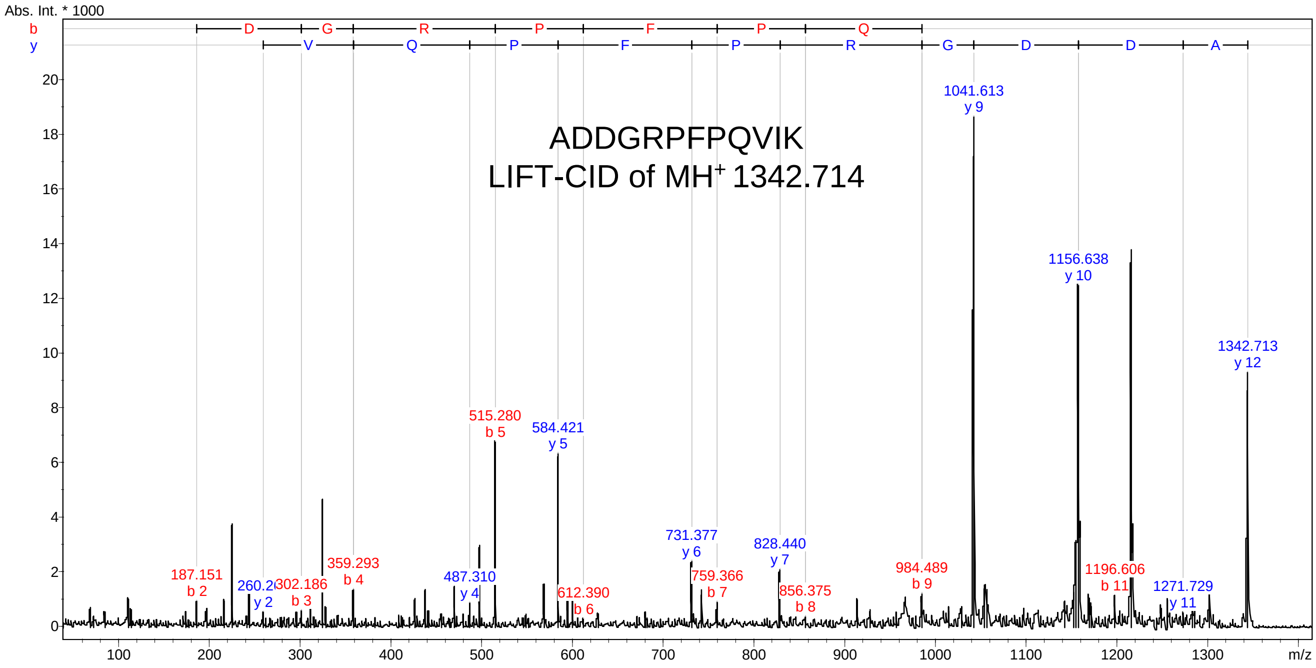

MSMS sequence analysis from the fragmentation of a precursor ion m/z 1342.714 by MALDI-ToF/ToF mass spectrometer

SPOT 11

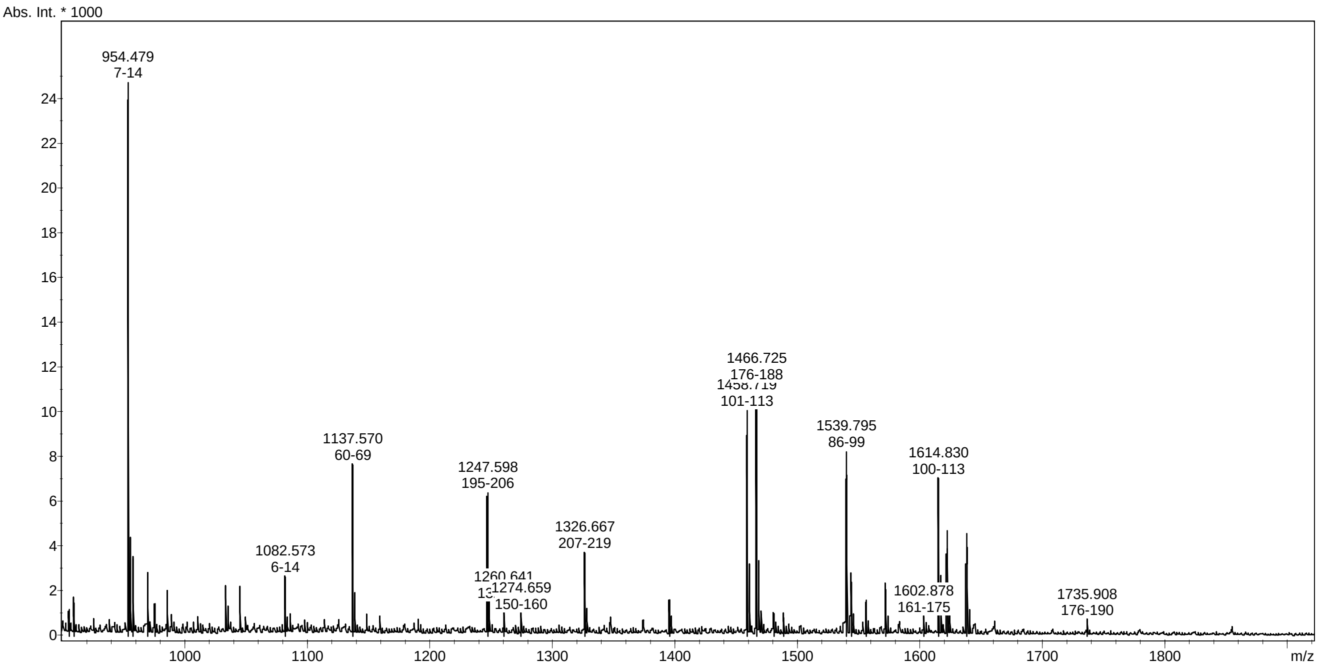

A representative MALDI-ToF PMF spectrum of spot 11

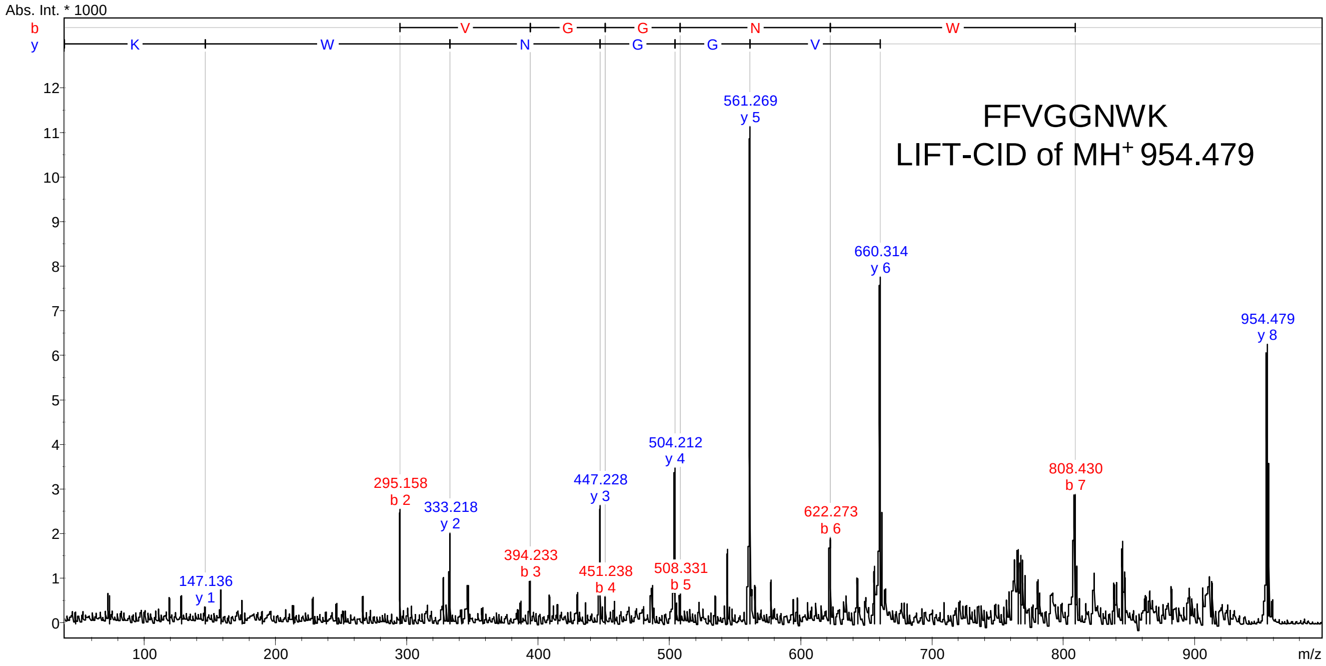

MSMS sequence analysis from the fragmentation of a precursor ion m/z 954.479 by MALDI-ToF/ToF mass spectrometer

SPOT 12

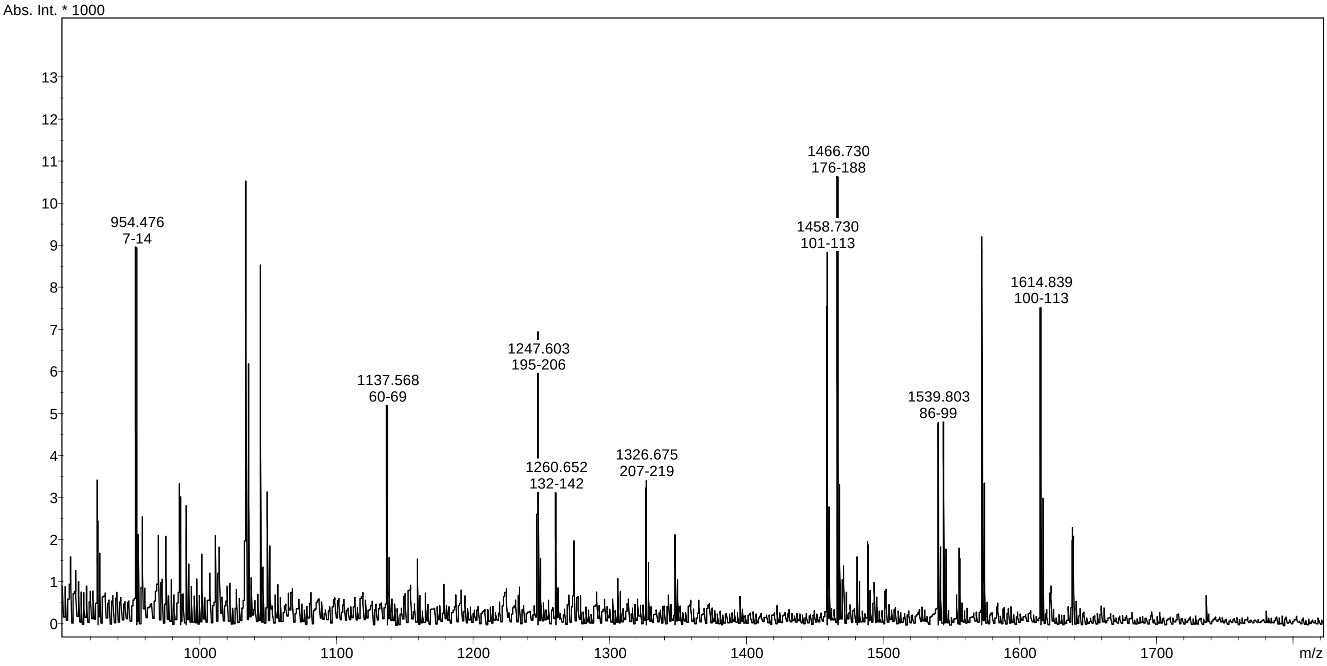

A representative MALDI-ToF PMF spectrum of spot 12

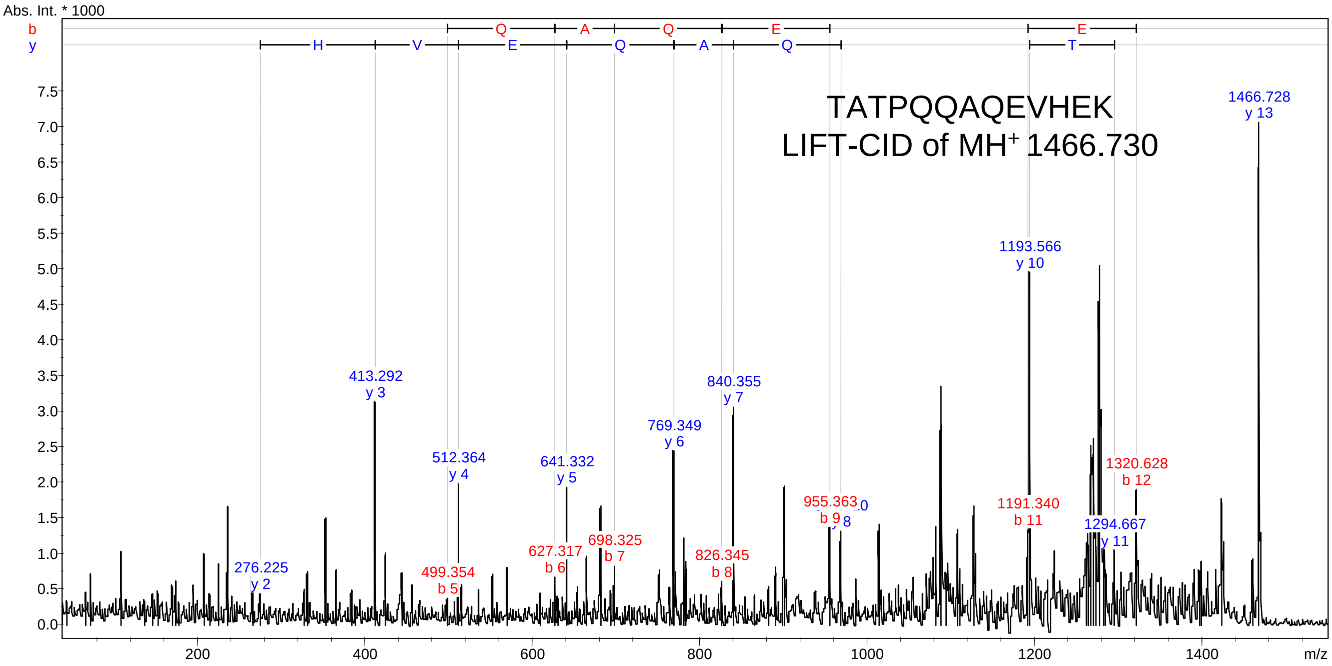

MSMS sequence analysis from the fragmentation of a precursor ion m/z 1466.730 by MALDI-ToF/ToF mass spectrometer

SPOT 13

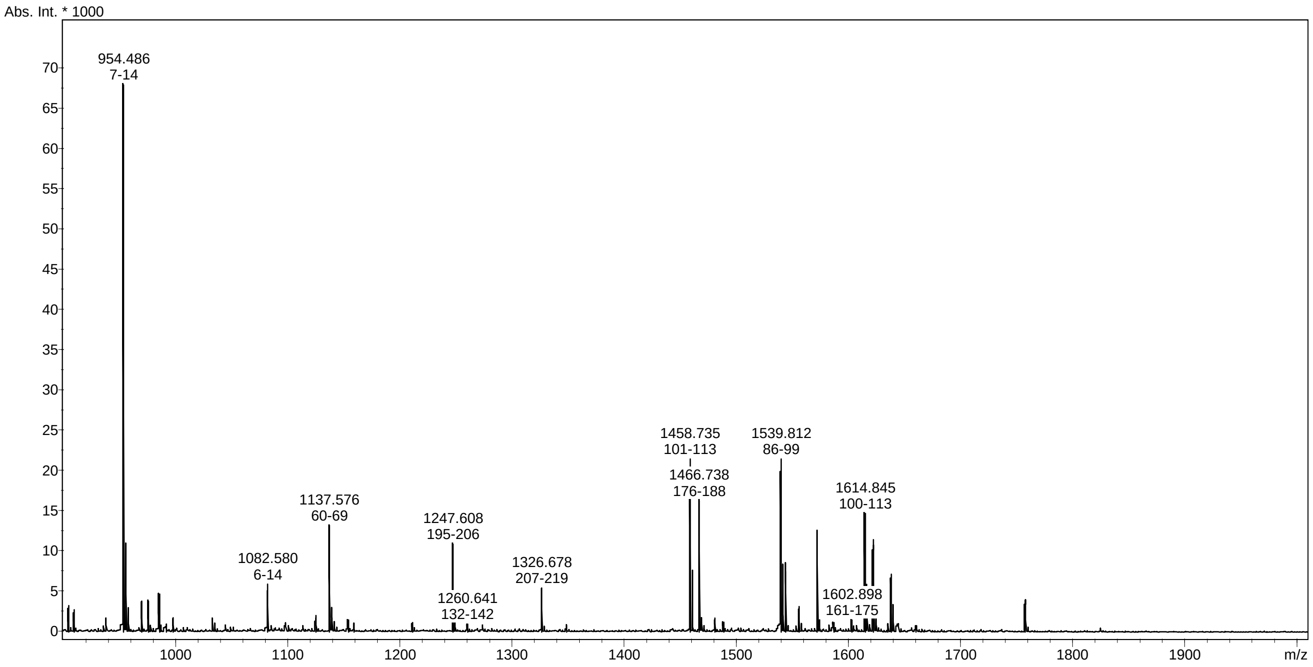

A representative MALDI-ToF PMF spectrum of spot 13

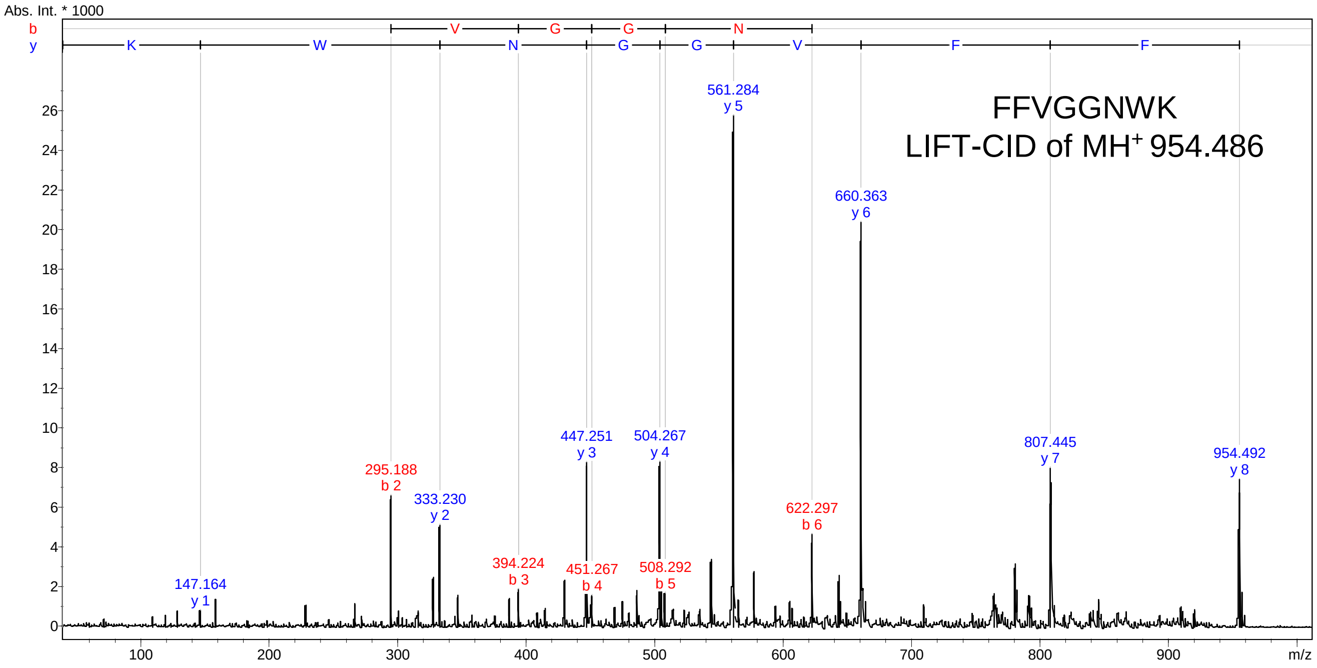

MSMS sequence analysis from the fragmentation of a precursor ion m/z 954.486 by MALDI-ToF/ToF mass spectrometer

SPOT 14

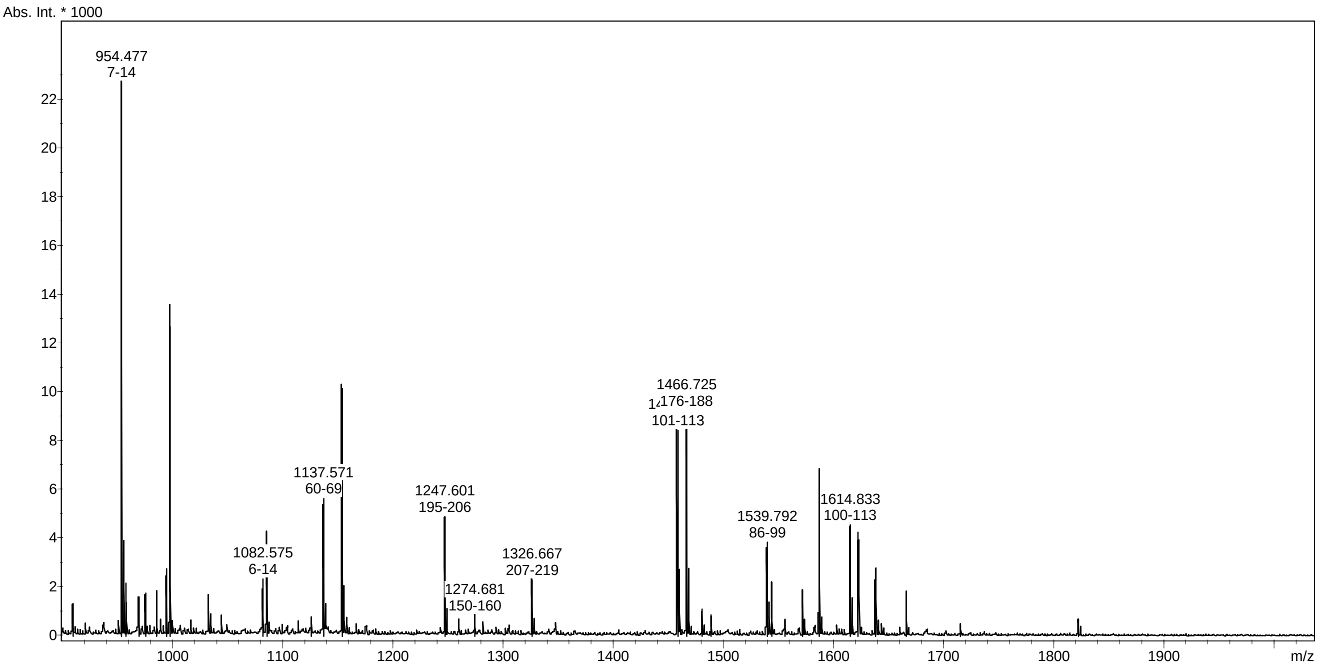

A representative MALDI-ToF PMF spectrum of spot 14

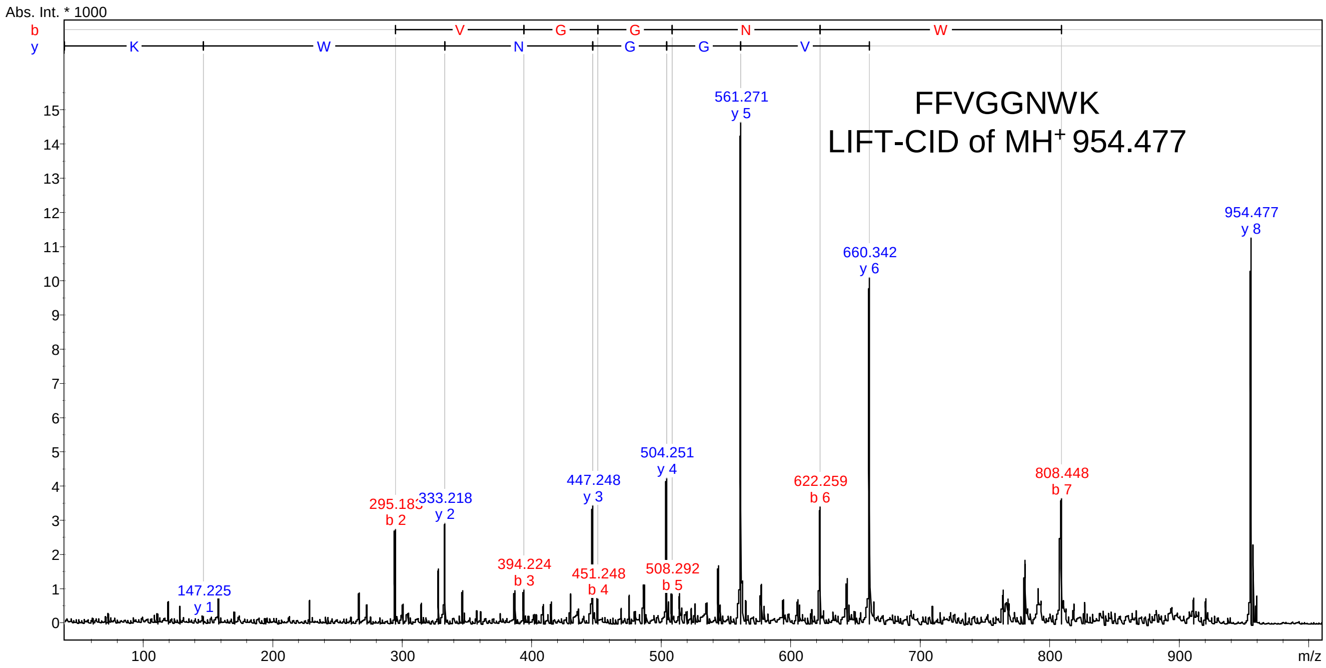

MSMS sequence analysis from the fragmentation of a precursor ion m/z 954.477 by MALDI-ToF/ToF mass spectrometer

SPOT 15

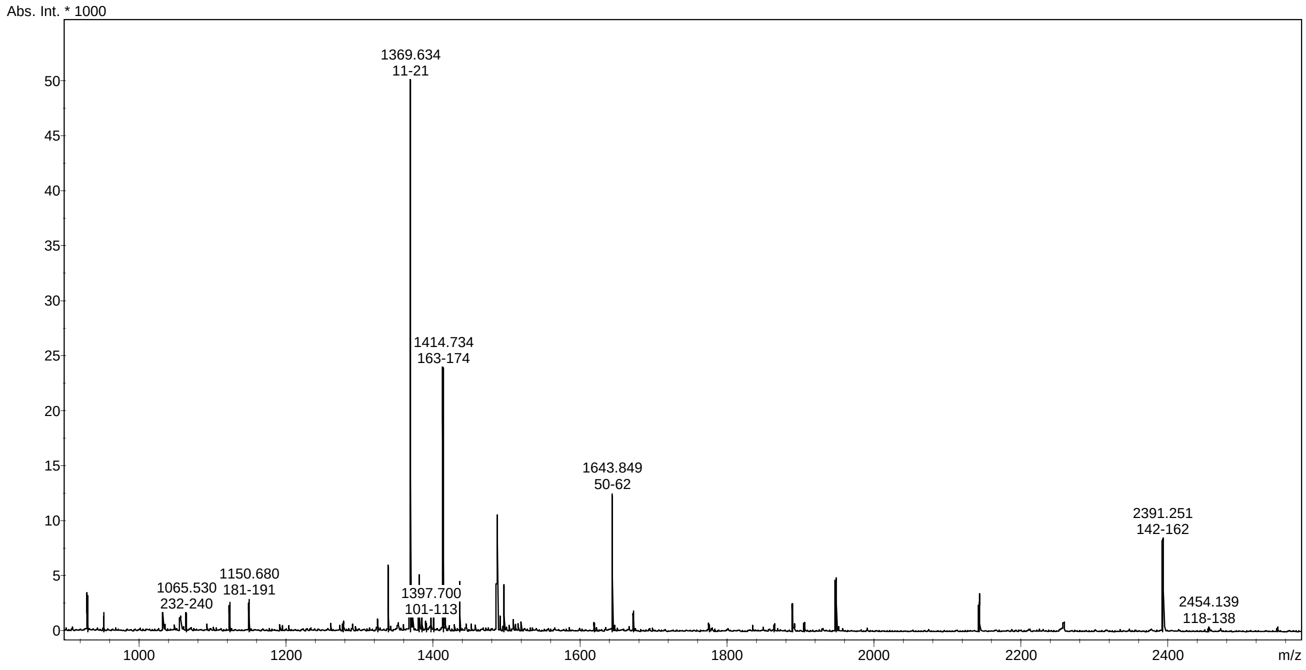

A representative MALDI-ToF PMF spectrum of spot 15

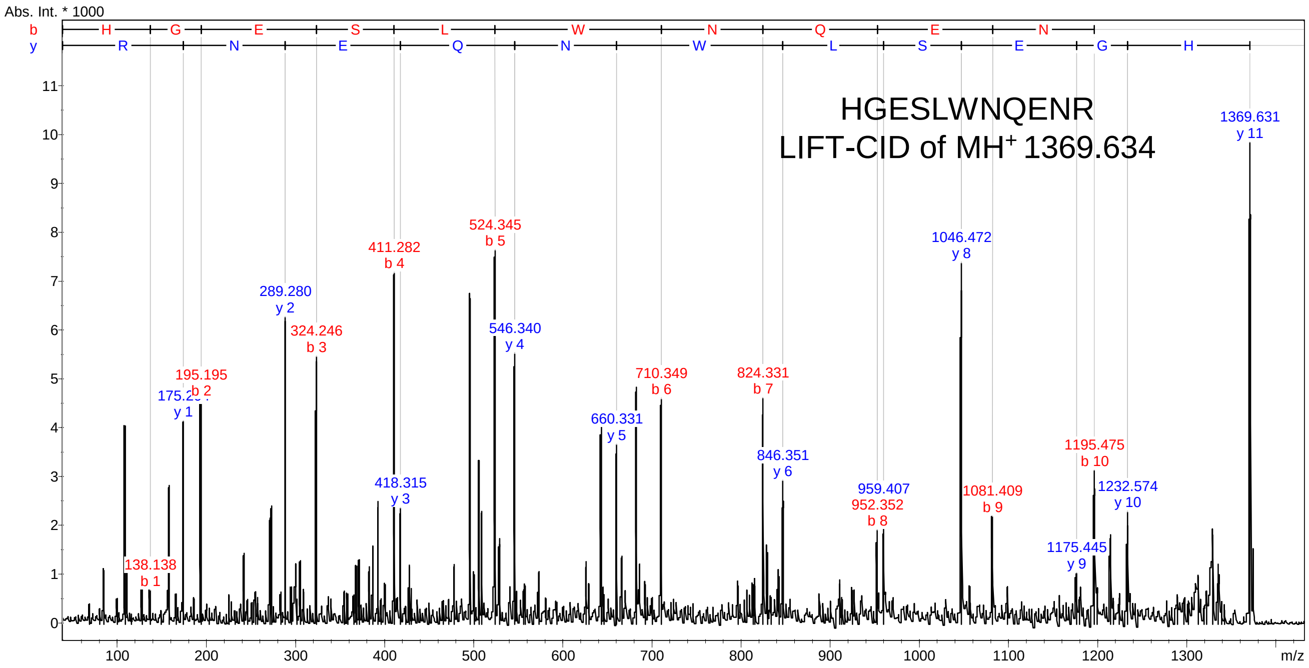

MSMS sequence analysis from the fragmentation of a precursor ion m/z 1369.634 by MALDI-ToF/ToF mass spectrometer

SPOT 16

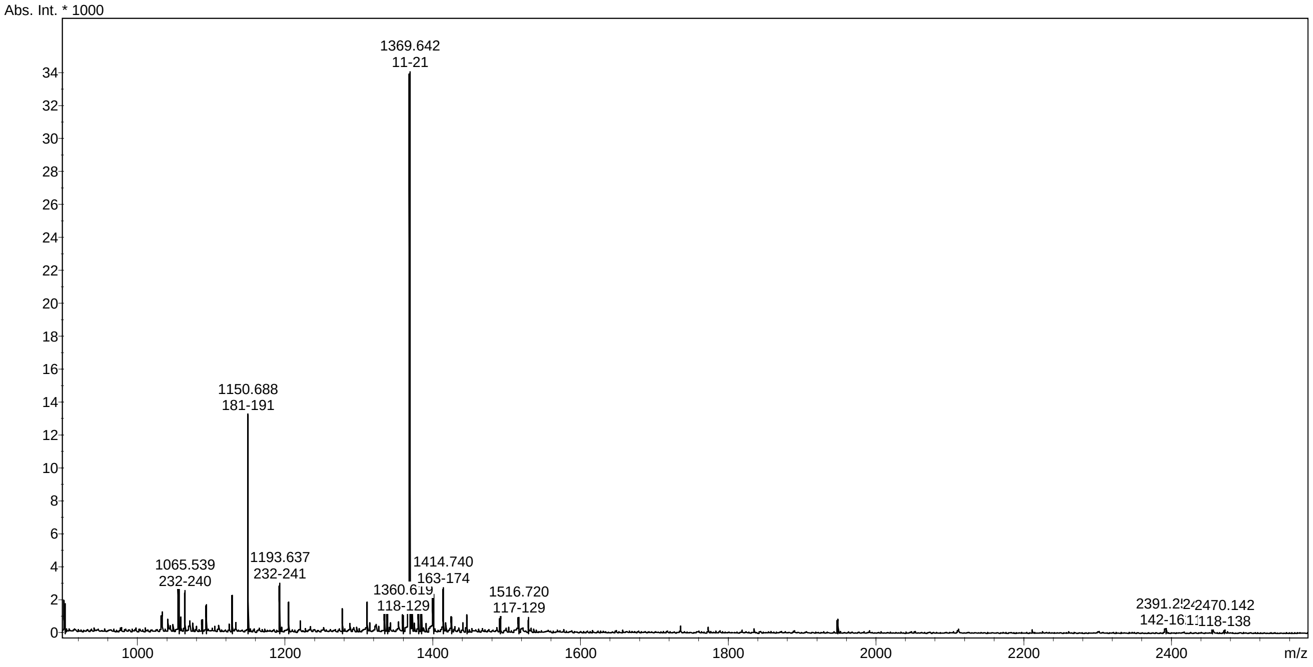

A representative MALDI-ToF PMF spectrum of spot 16

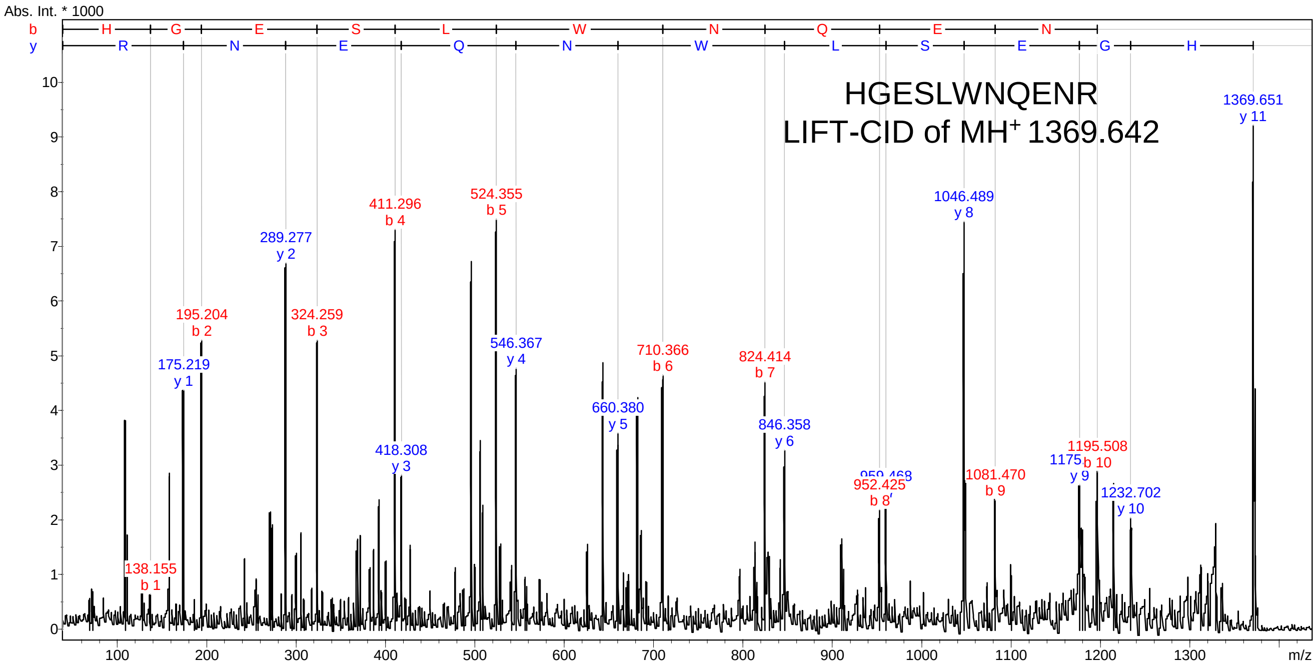

MSMS sequence analysis from the fragmentation of a precursor ion m/z 1369.642 by MALDI-ToF/ToF mass spectrometer

SPOT 17

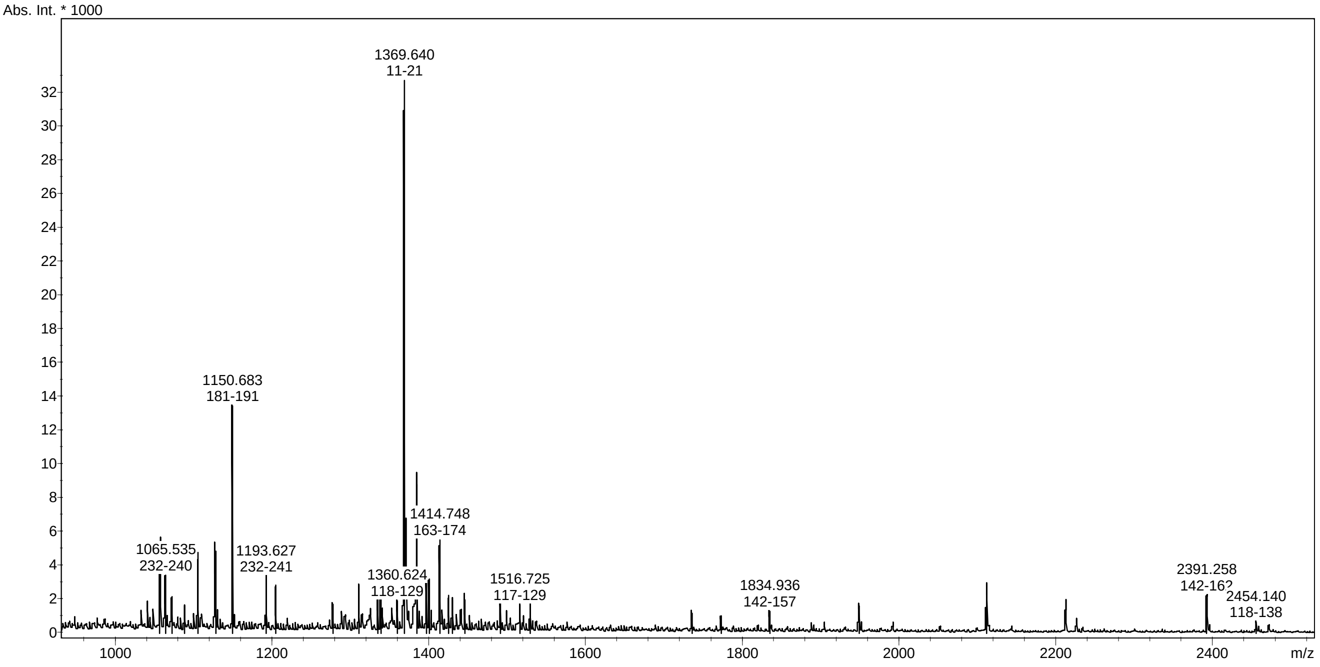

A representative MALDI-ToF PMF spectrum of spot 17

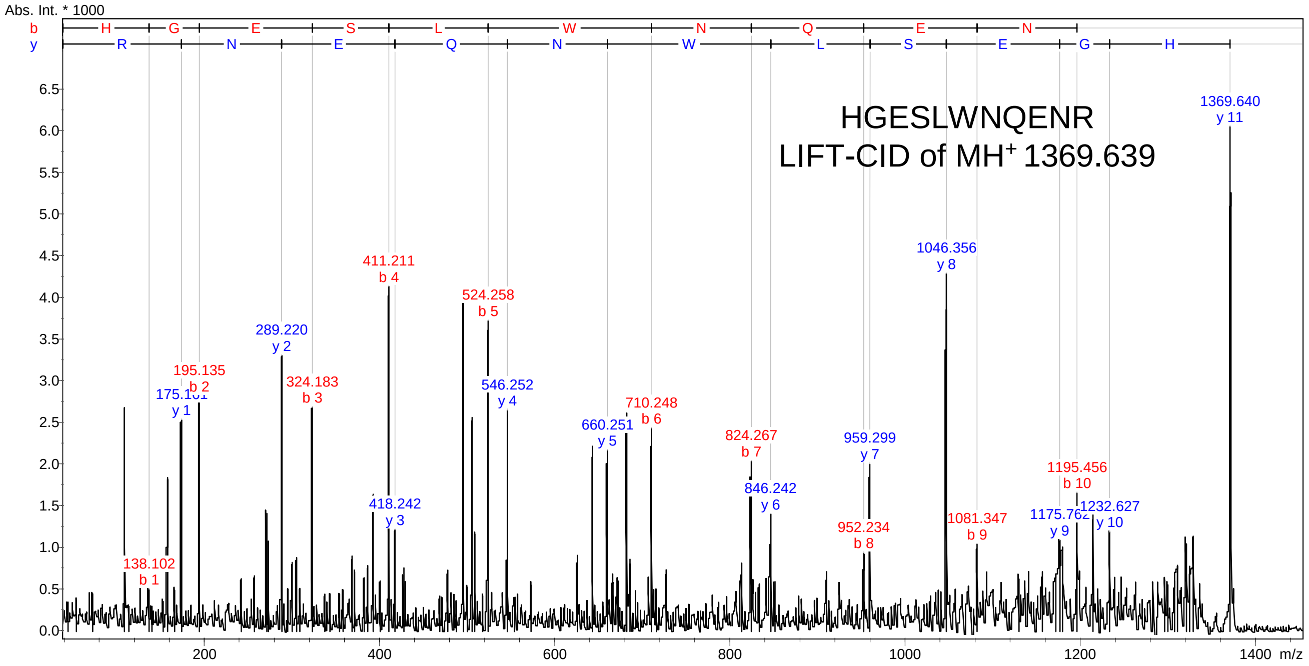

MSMS sequence analysis from the fragmentation of a precursor ion m/z 1369.639 by MALDI-ToF/ToF mass spectrometer

SPOT 19

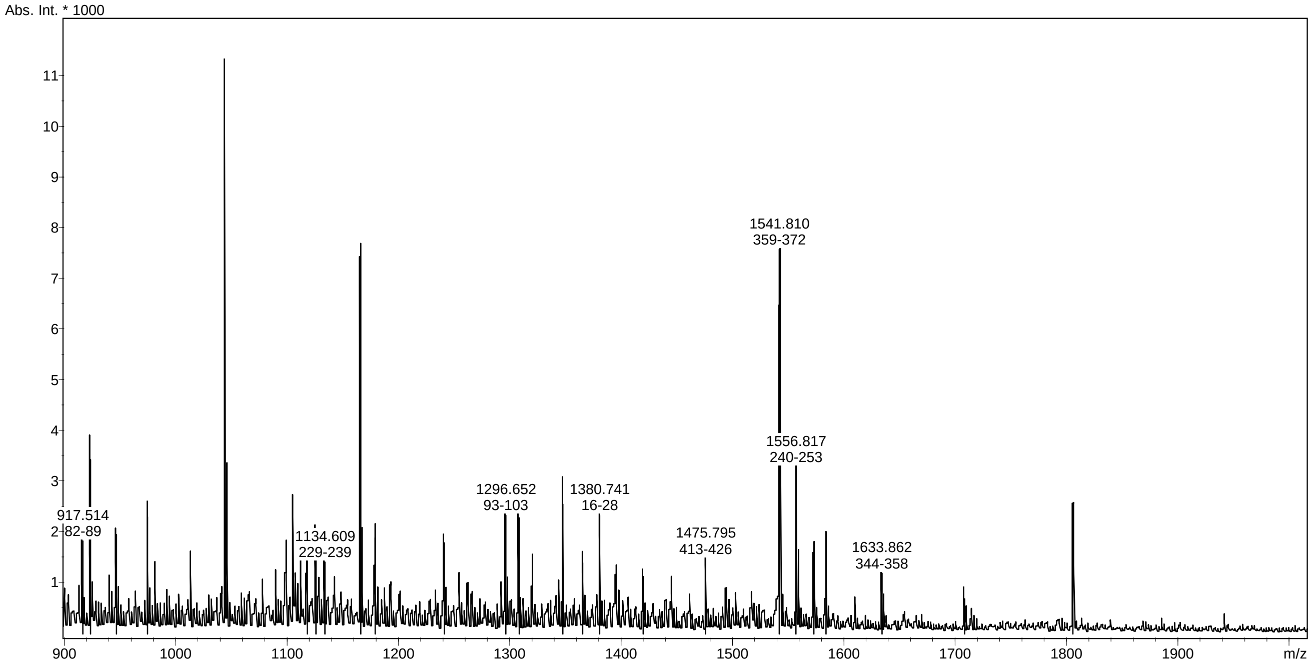

A representative MALDI-ToF PMF spectrum of spot 19

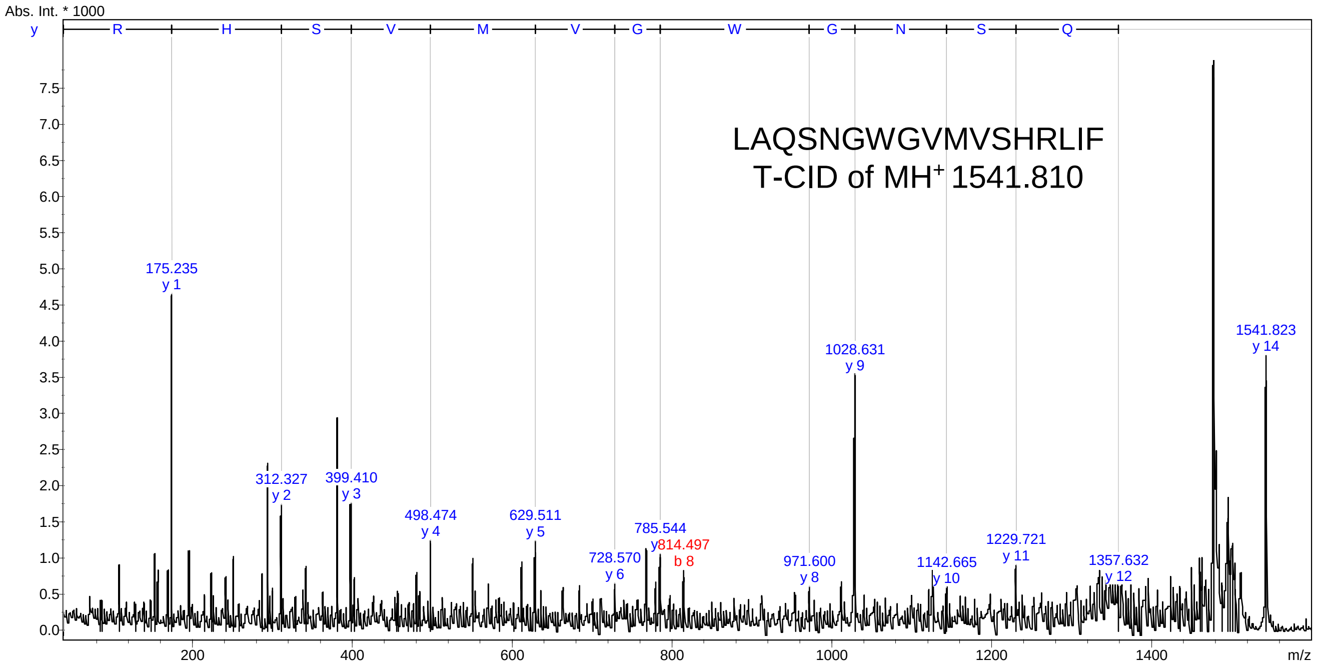

MSMS sequence analysis from the fragmentation of a precursor ion m/z 1541.810 by MALDI-ToF/ToF mass spectrometer

SPOT 20

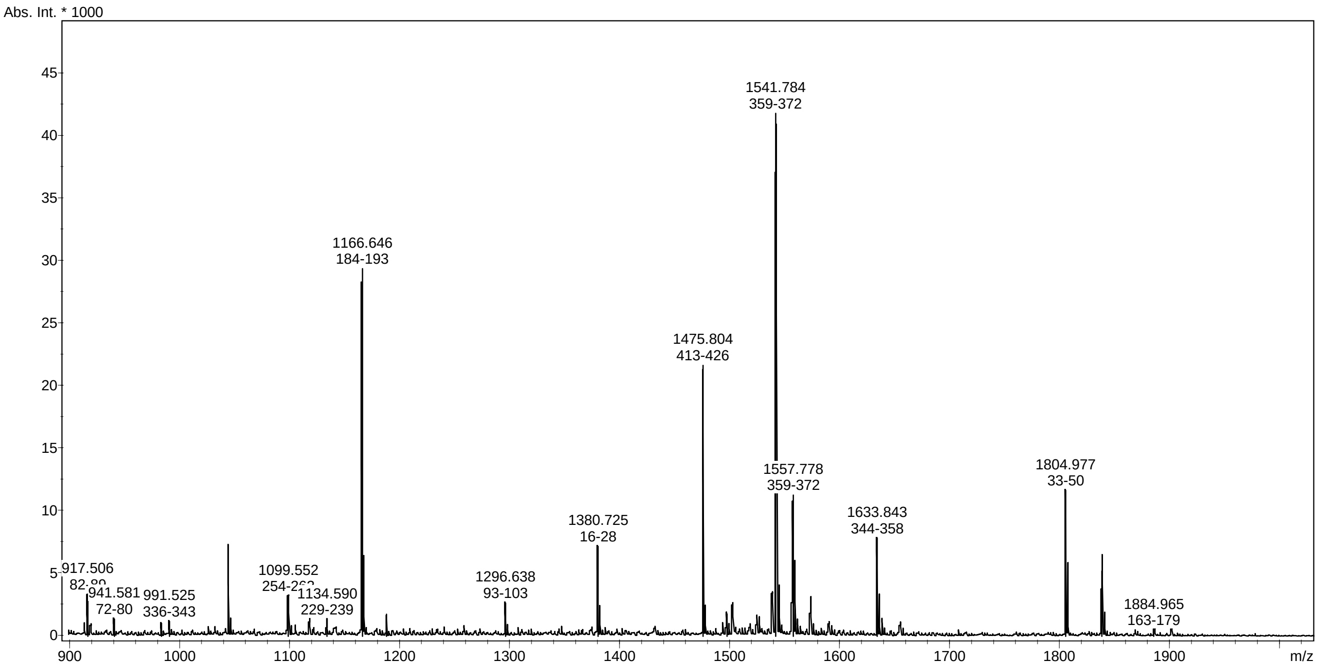

A representative MALDI-ToF PMF spectrum of spot 20

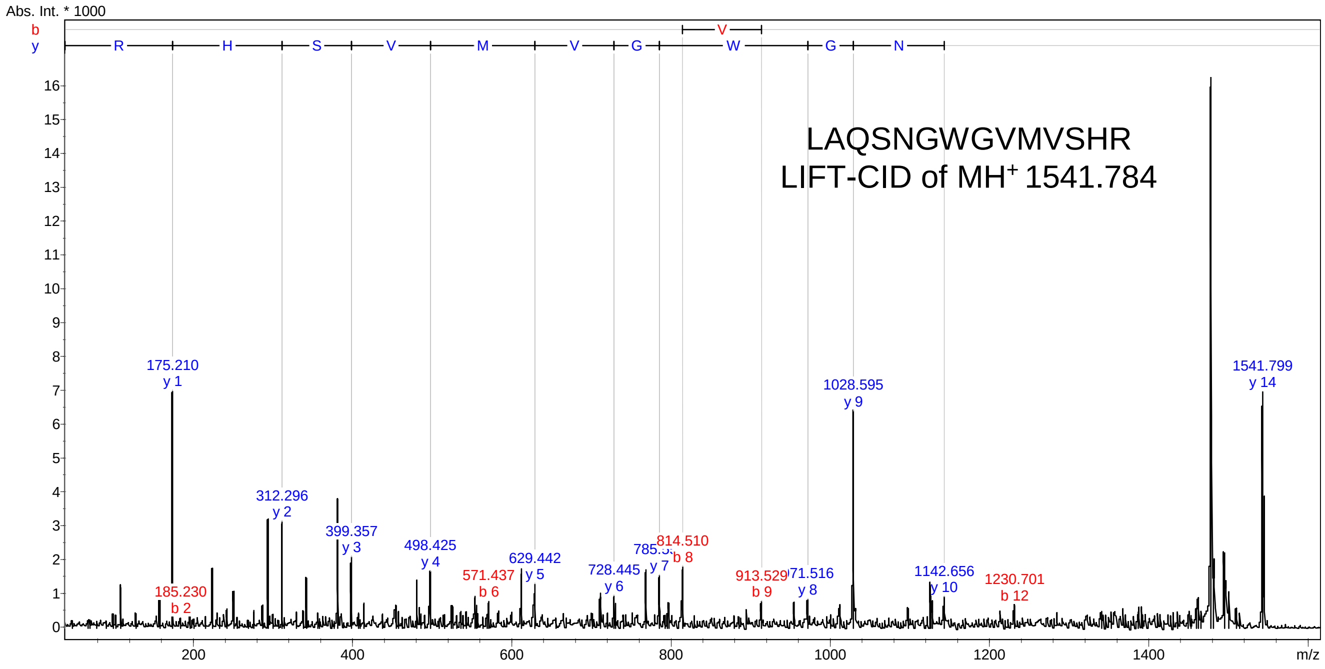

MSMS sequence analysis from the fragmentation of a precursor ion m/z 1541.784 by MALDI-ToF/ToF mass spectrometer

SPOT 21

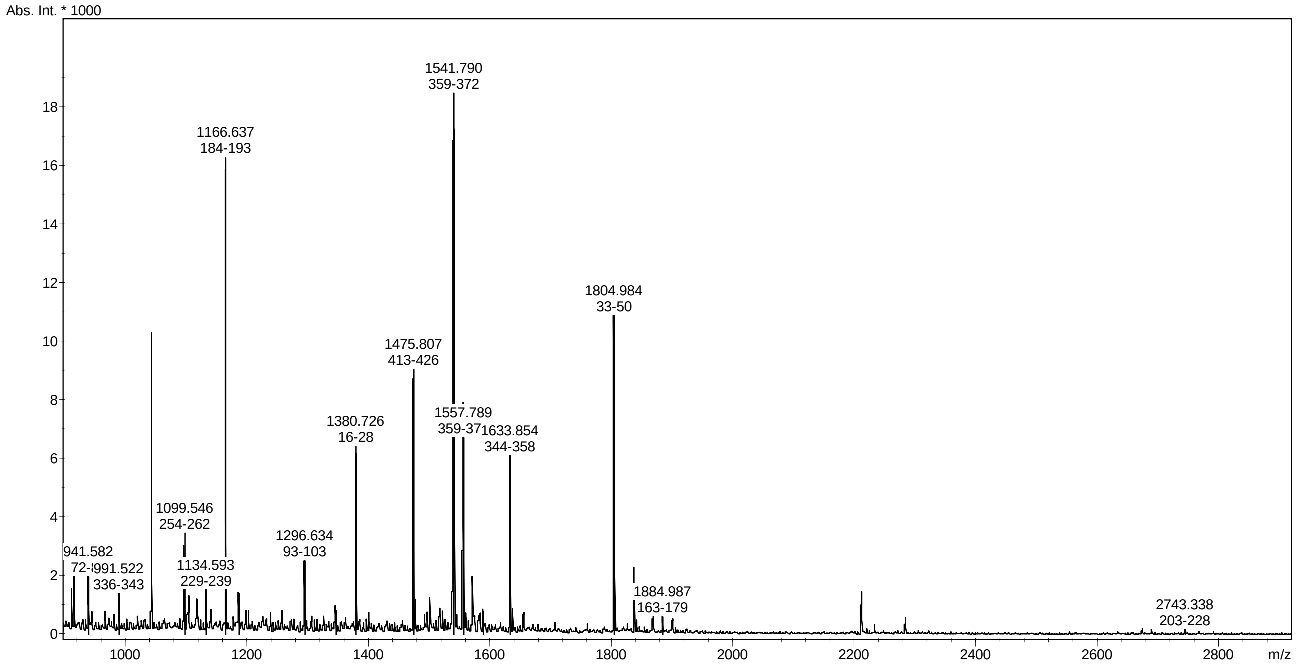

A representative MALDI-ToF PMF spectrum of spot 21

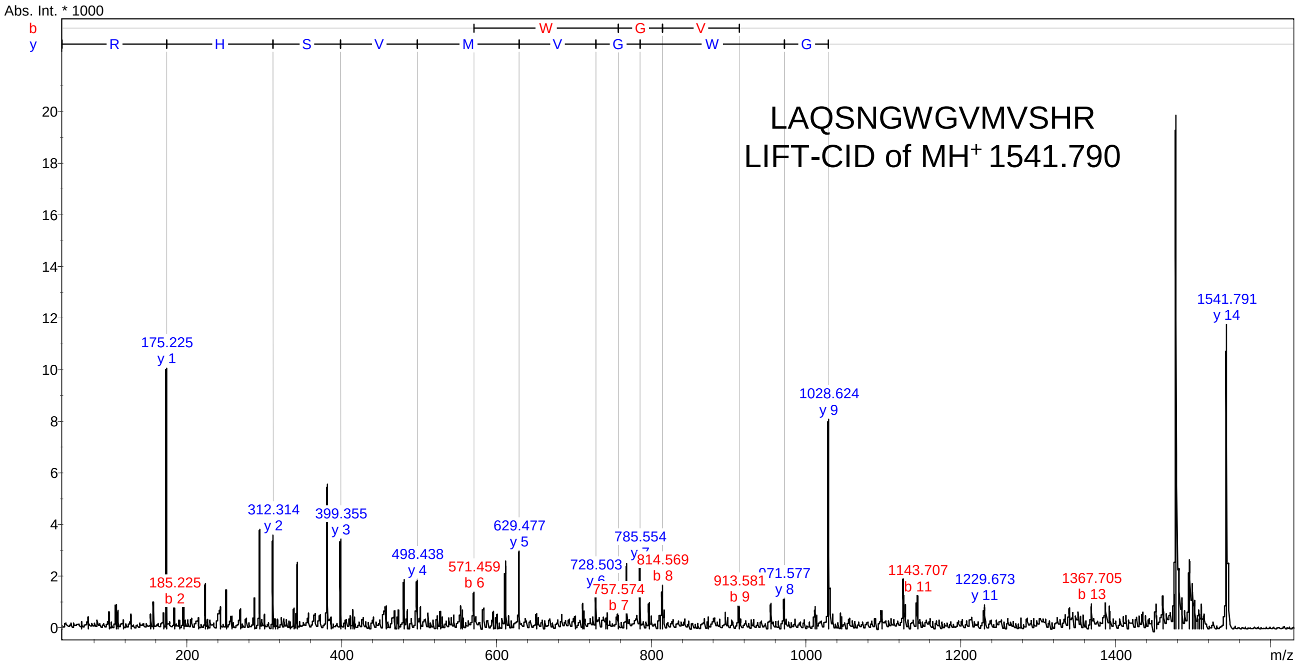

MSMS sequence analysis from the fragmentation of a precursor ion m/z 1541.790 by MALDI-ToF/ToF mass spectrometer

SPOT 22

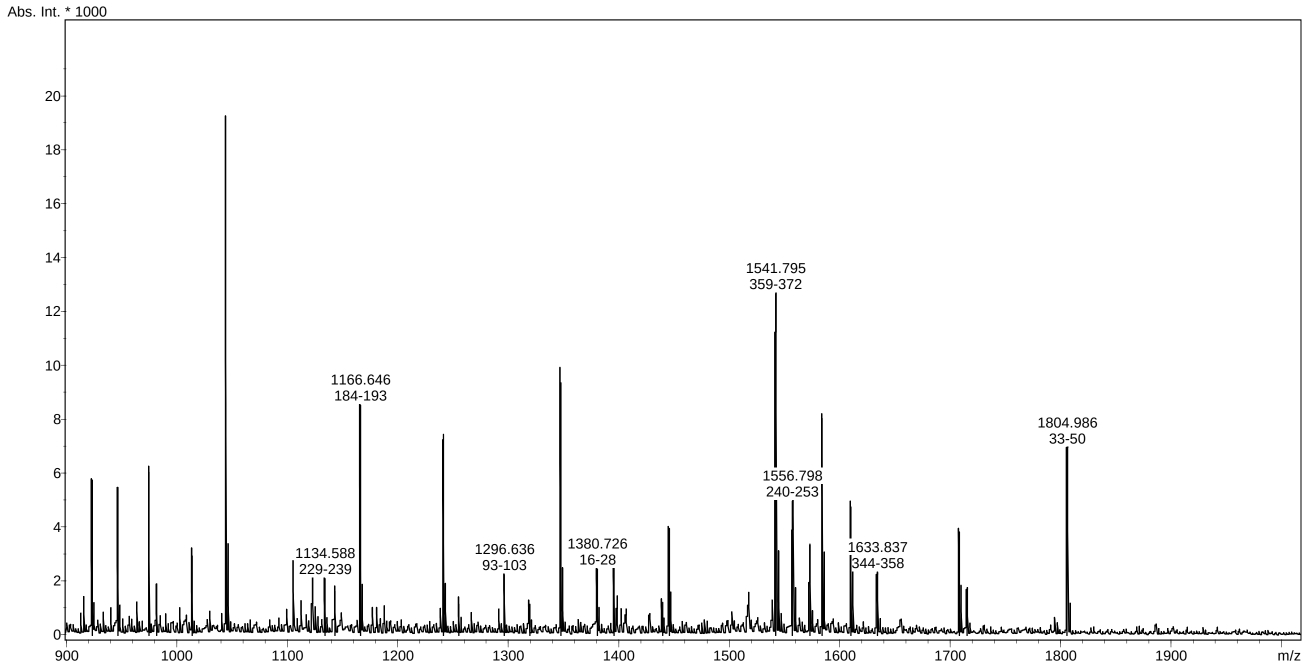

A representative MALDI-ToF PMF spectrum of spot 22

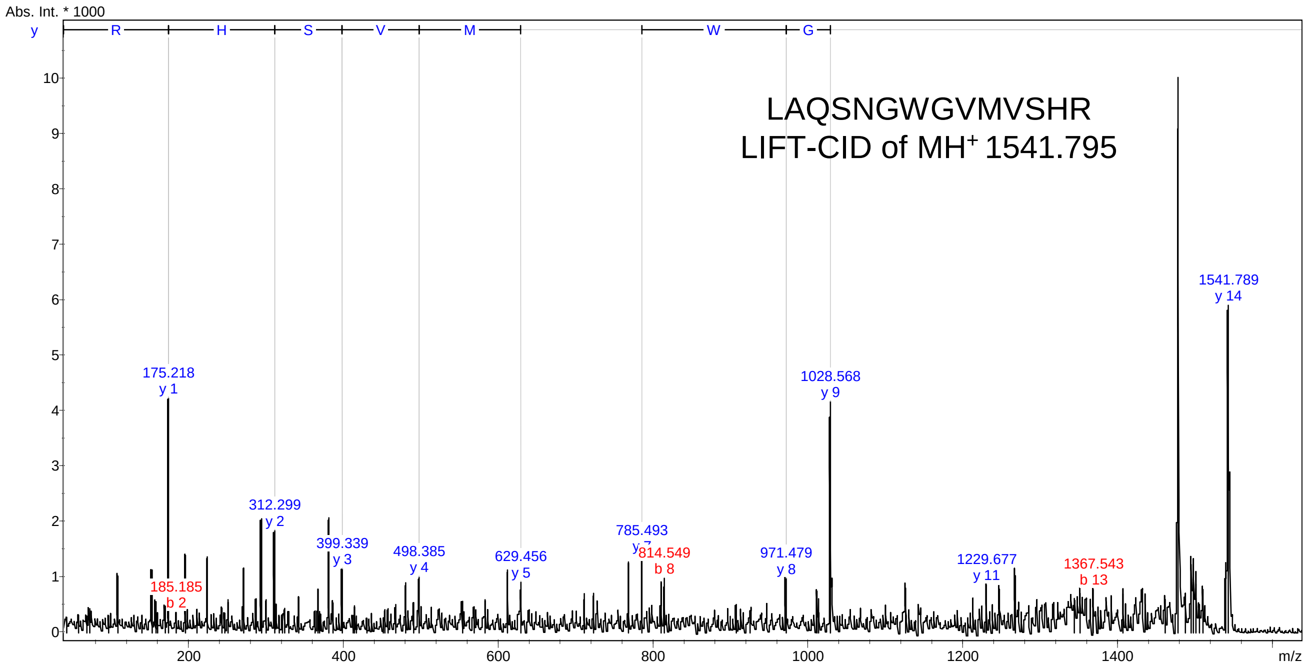

MSMS sequence analysis from the fragmentation of a precursor ion m/z 1541.795 by MALDI-ToF/ToF mass spectrometer

SPOT 23

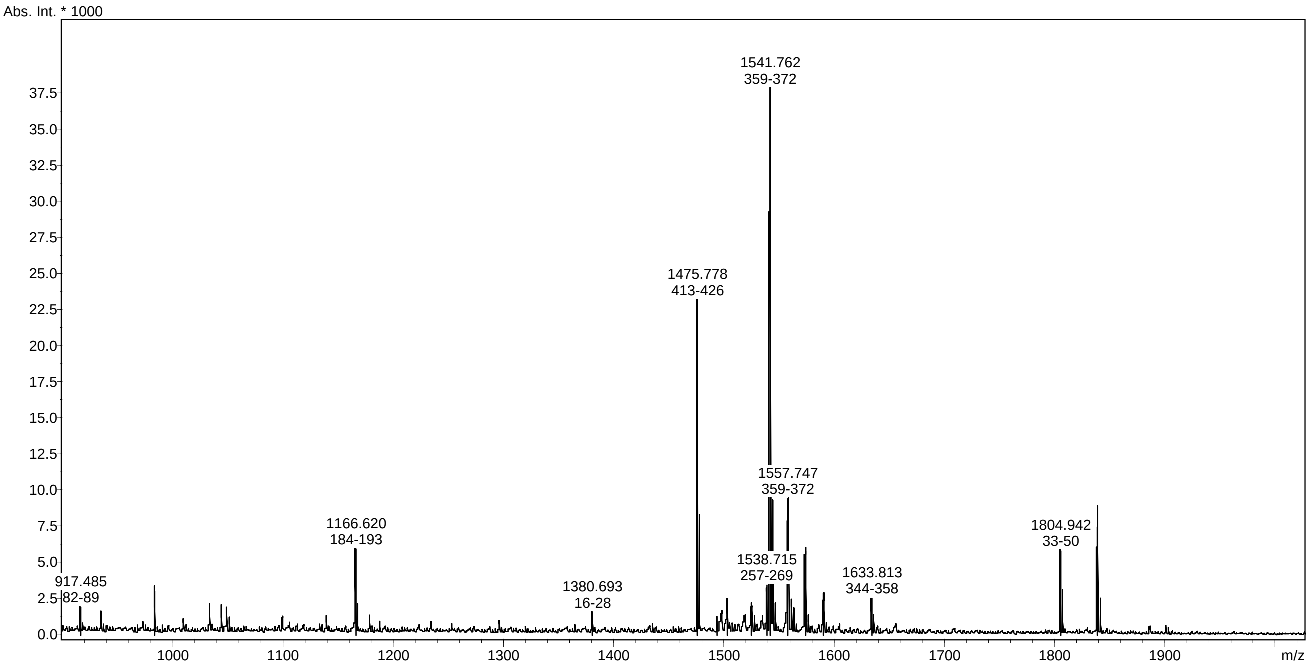

A representative MALDI-ToF PMF spectrum of spot 23

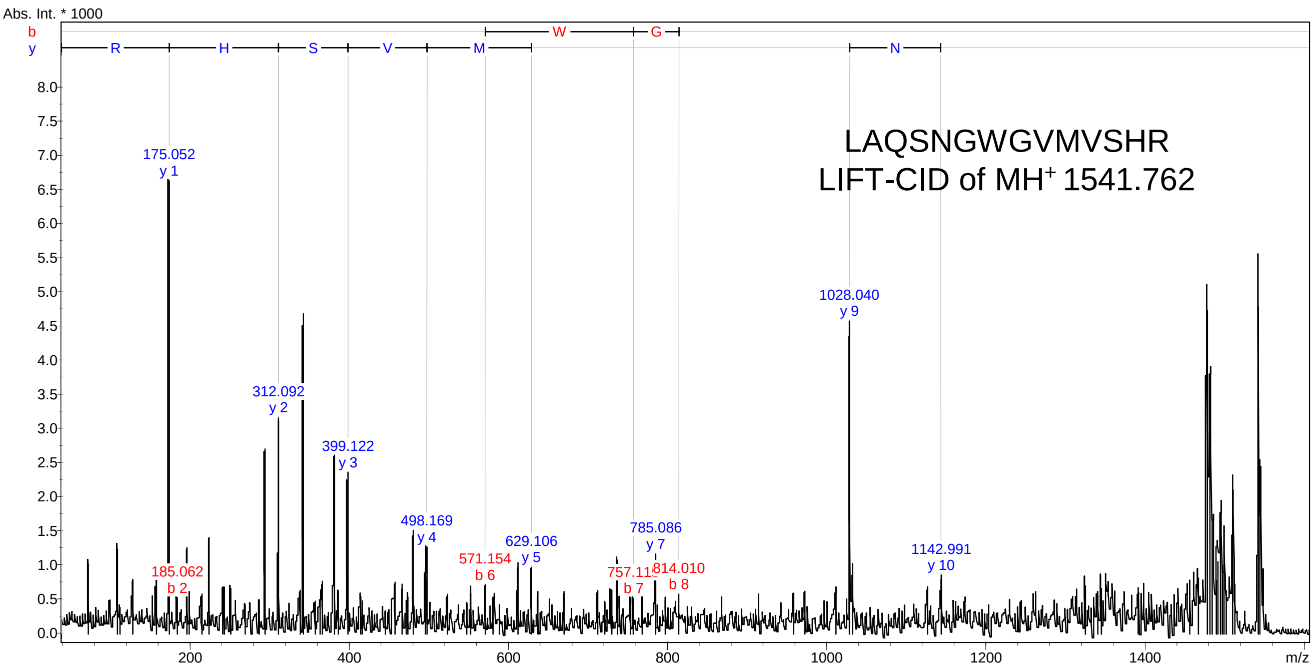

MSMS sequence analysis from the fragmentation of a precursor ion m/z 1541.762 by MALDI-ToF/ToF mass spectrometer

SPOT 25

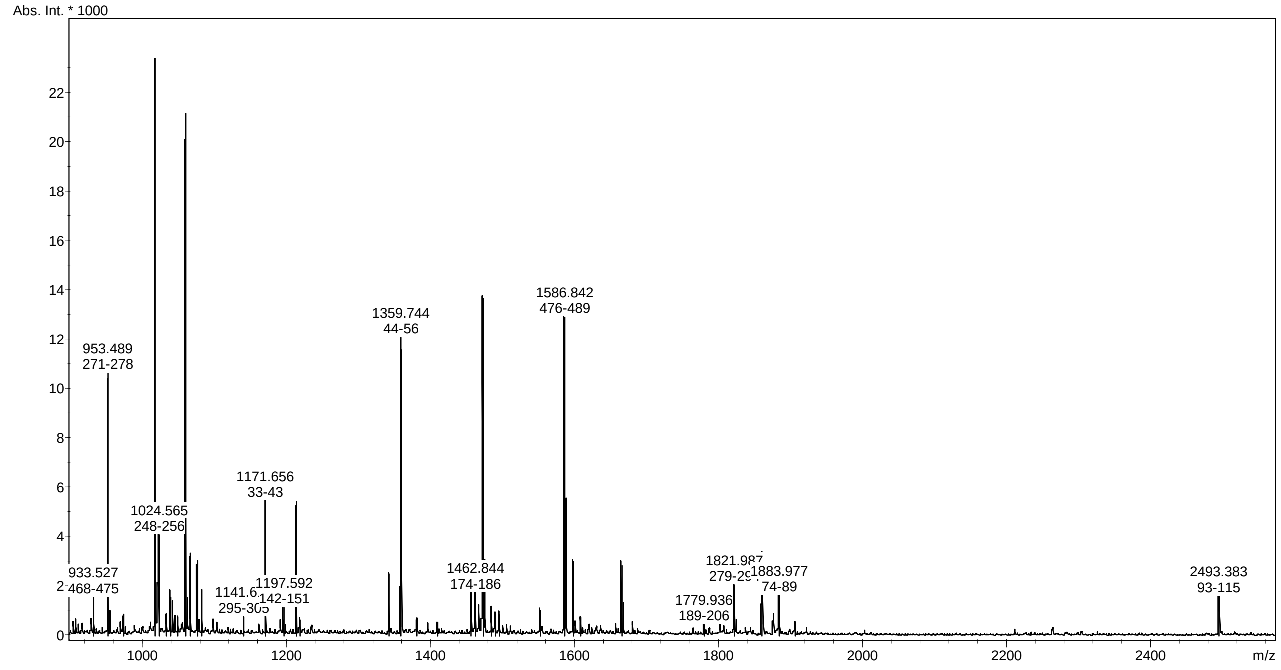

A representative MALDI-ToF PMF spectrum of spot 25

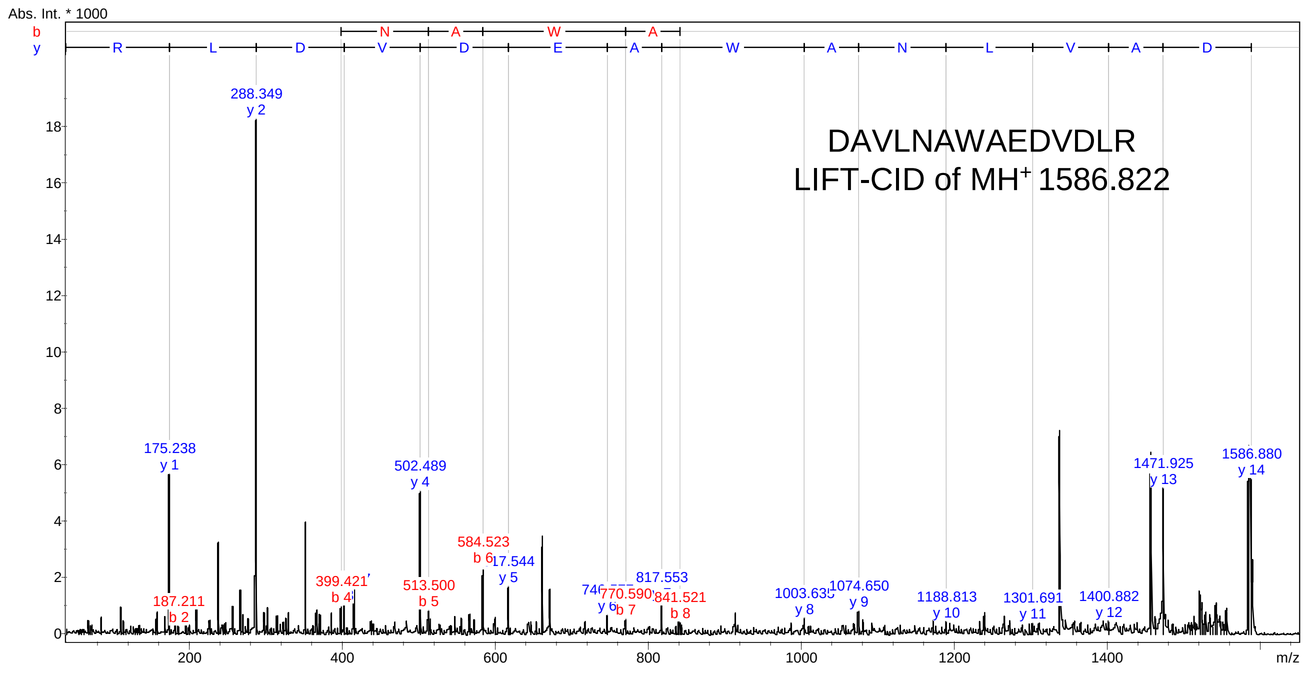

MSMS sequence analysis from the fragmentation of a precursor ion m/z 1586.822 by MALDI-ToF/ToF mass spectrometer

SPOT 26

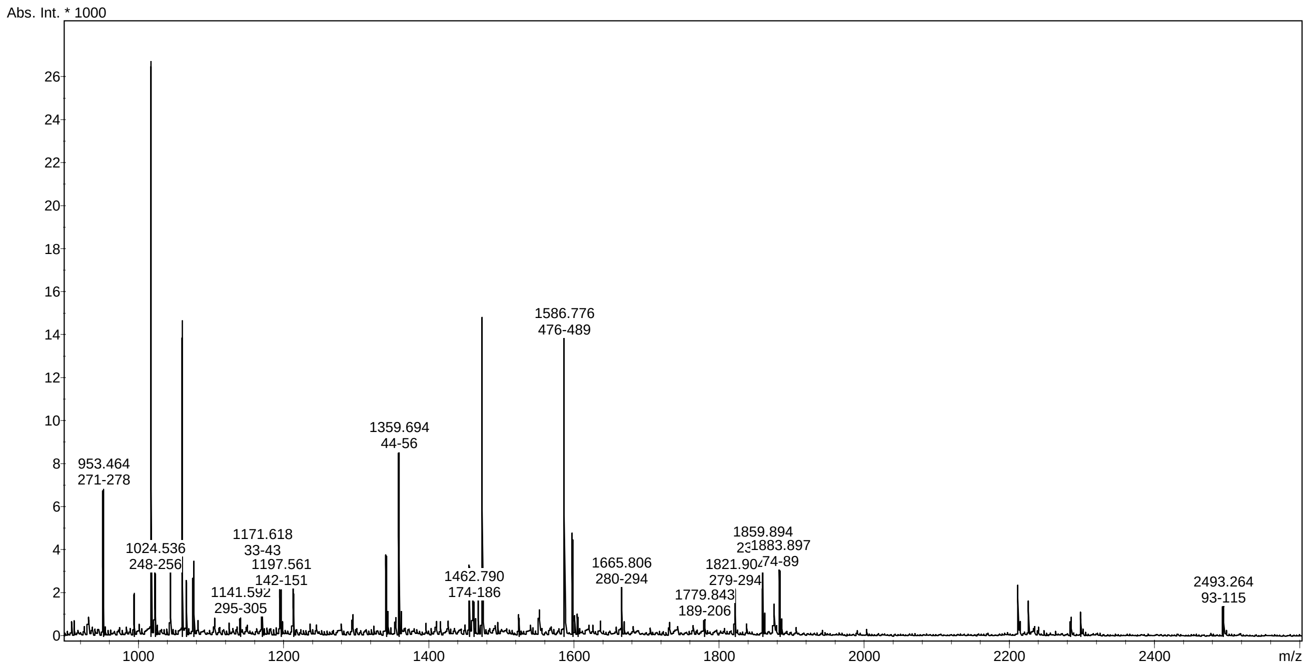

A representative MALDI-ToF PMF spectrum of spot 26

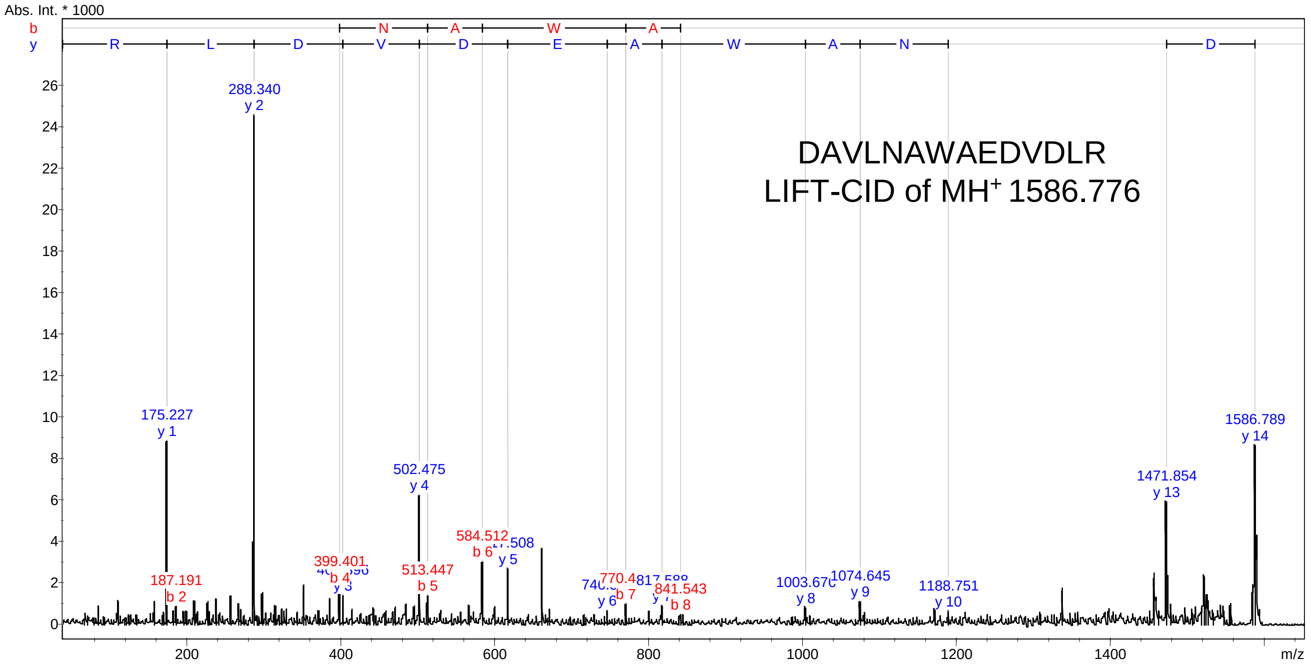

MSMS sequence analysis from the fragmentation of a precursor ion m/z 1586.776 by MALDI-ToF/ToF mass spectrometer

SPOT 27

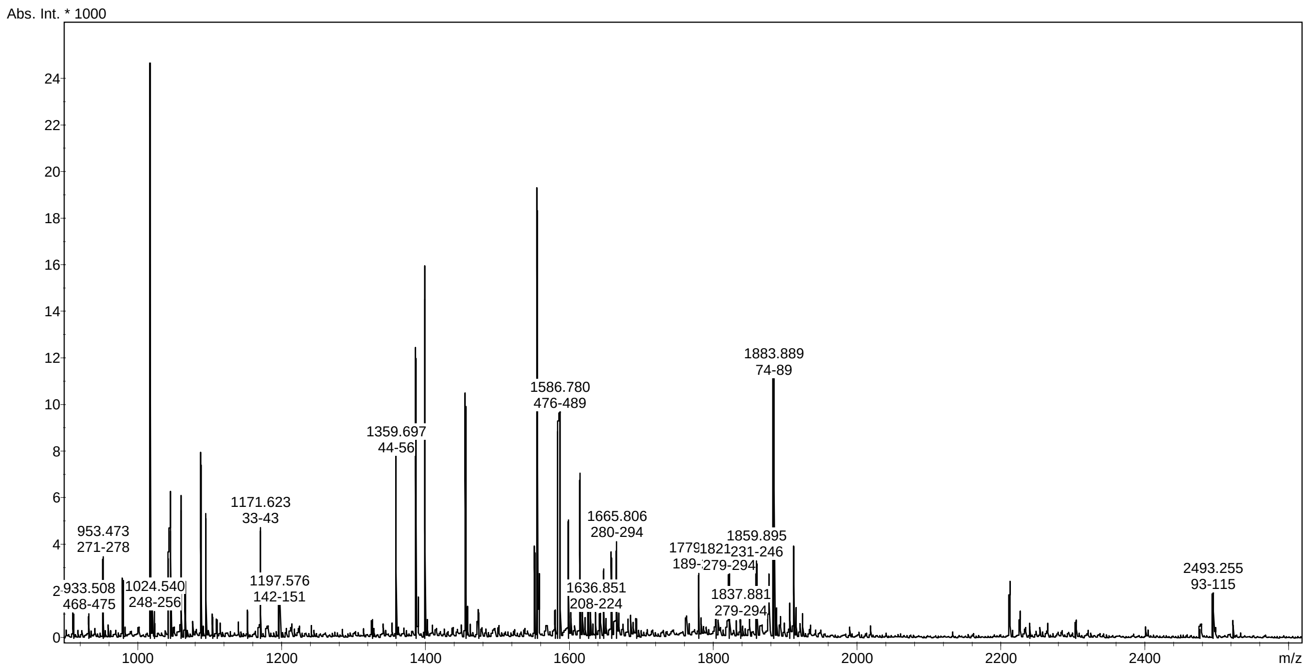

A representative MALDI-ToF PMF spectrum of spot 27

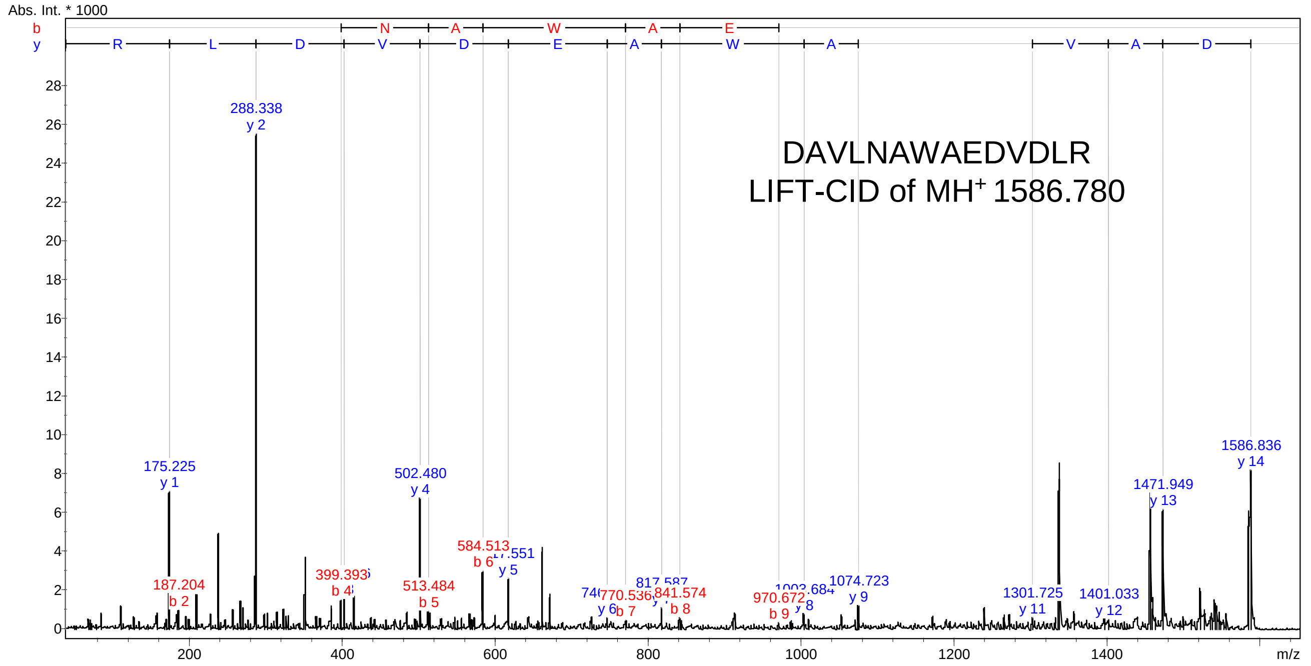

MSMS sequence analysis from the fragmentation of a precursor ion m/z 1586.780 by MALDI-ToF/ToF mass spectrometer

SPOT 28

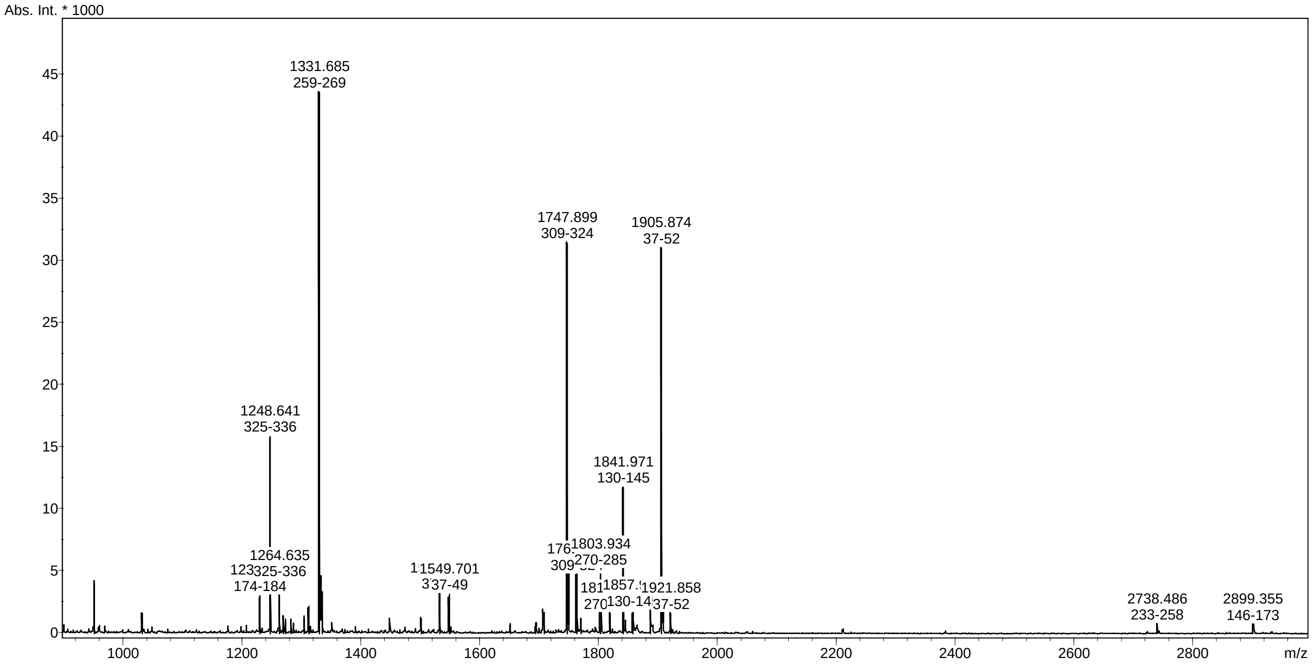

A representative MALDI-ToF PMF spectrum of spot 28

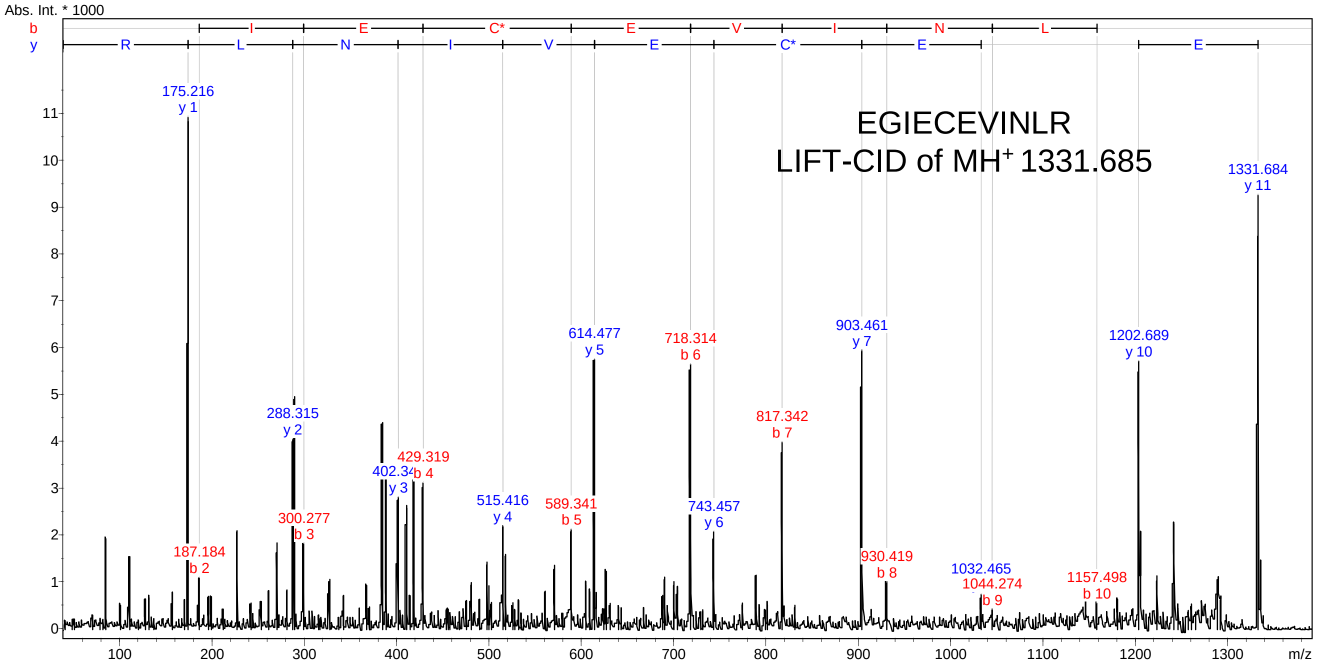

MSMS sequence analysis from the fragmentation of a precursor ion m/z 1331.685 by MALDI-ToF/ToF mass spectrometer

SPOT 29

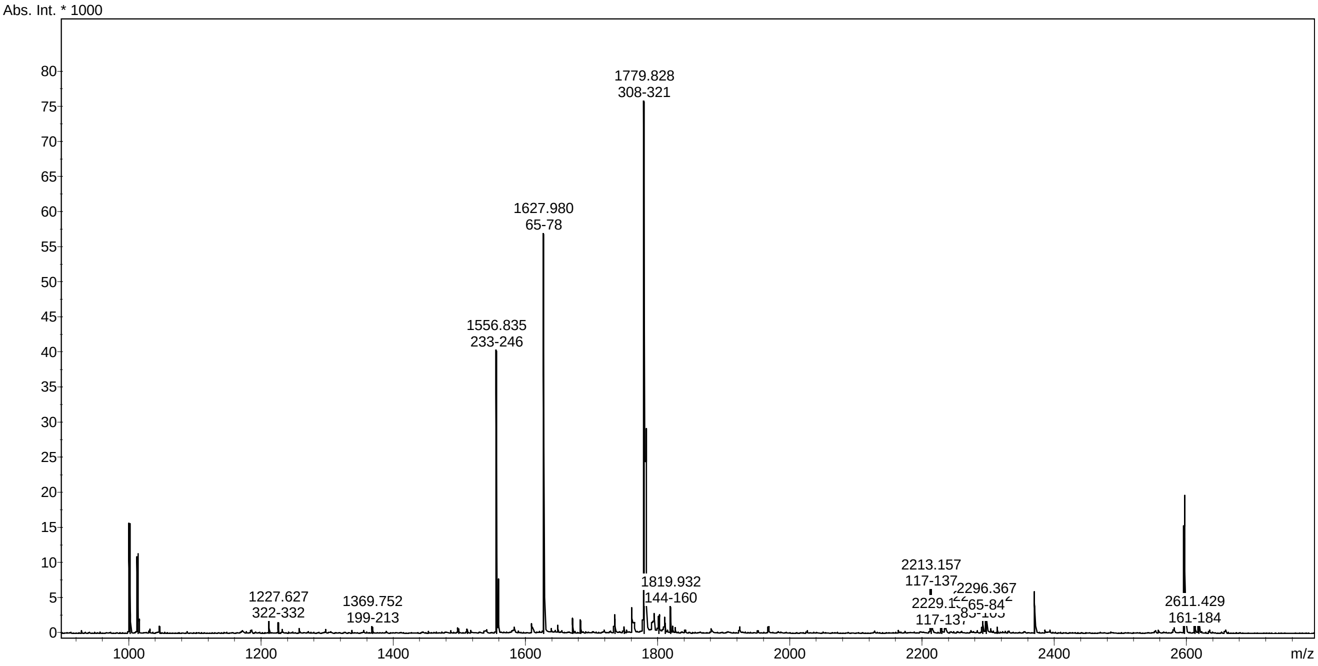

A representative MALDI-ToF PMF spectrum of spot 29

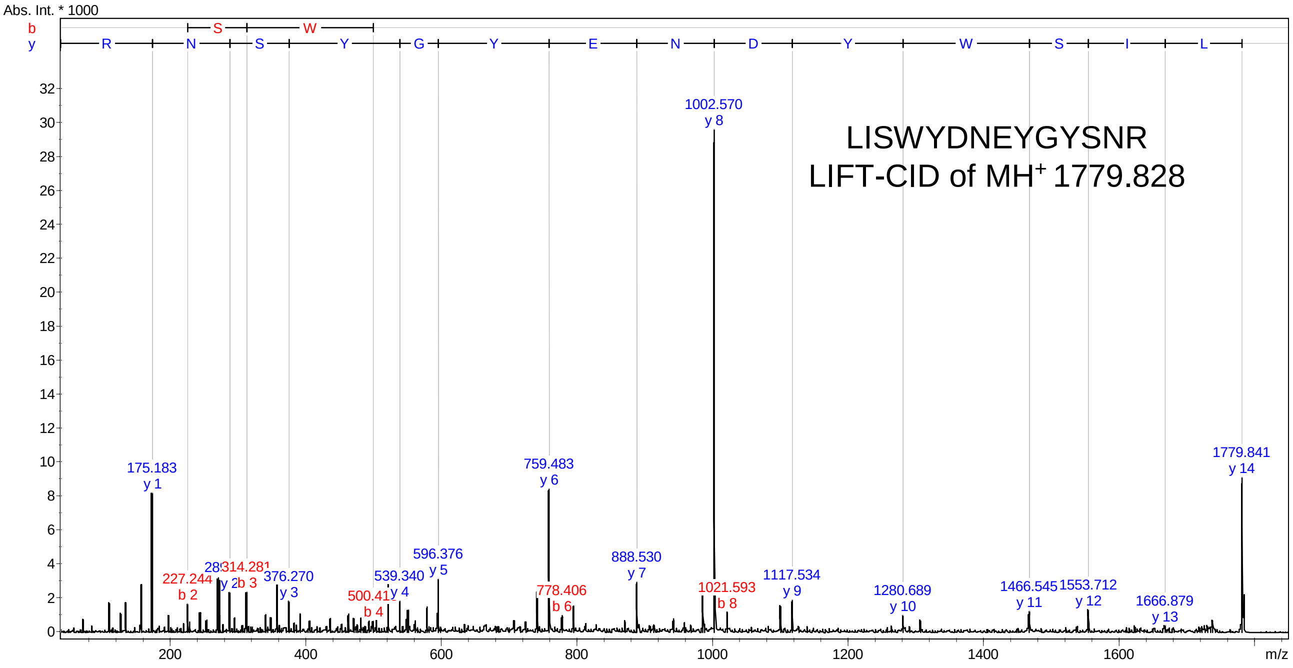

MSMS sequence analysis from the fragmentation of a precursor ion m/z 1779.828 by MALDI-ToF/ToF mass spectrometer

SPOT 30

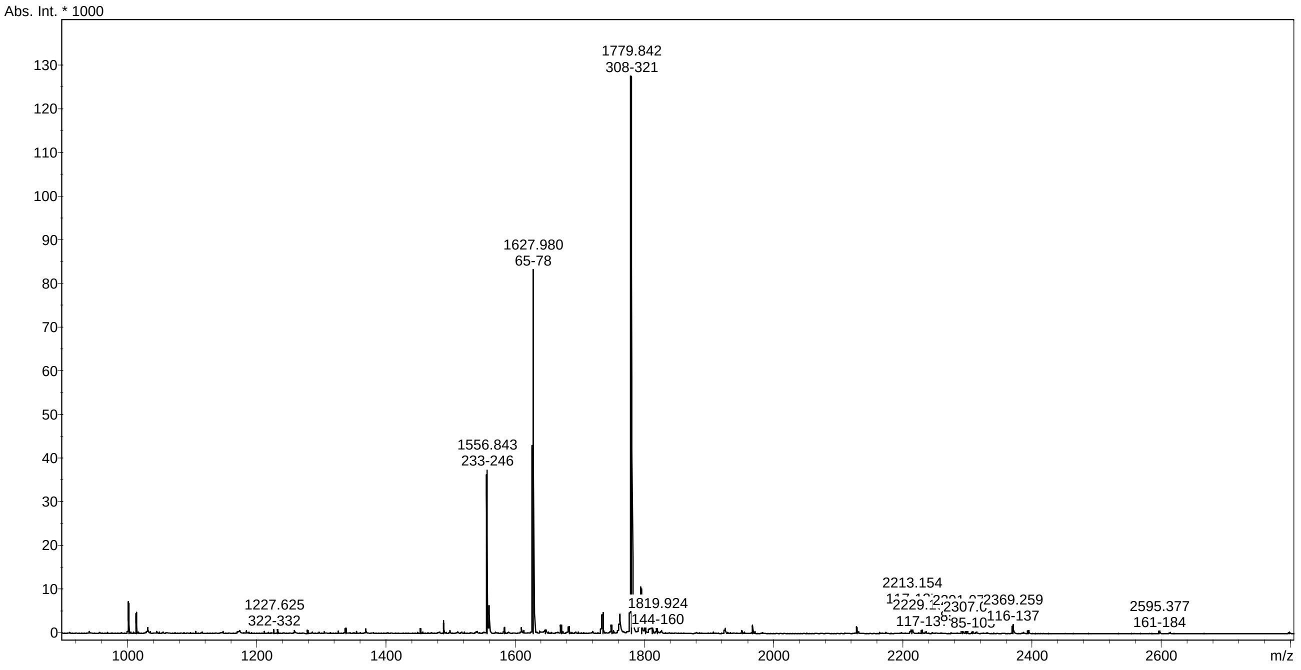

A representative MALDI-ToF PMF spectrum of spot 30

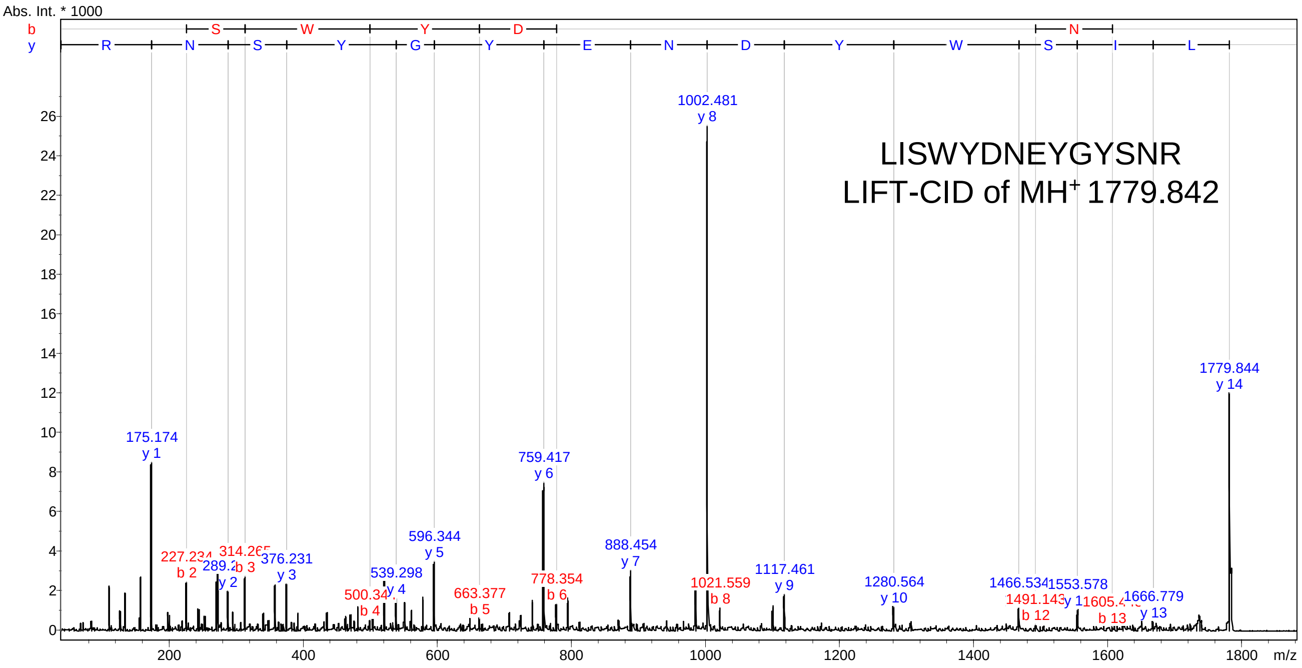

MSMS sequence analysis from the fragmentation of a precursor ion m/z 1779.842 by MALDI-ToF/ToF mass spectrometer

SPOT 31

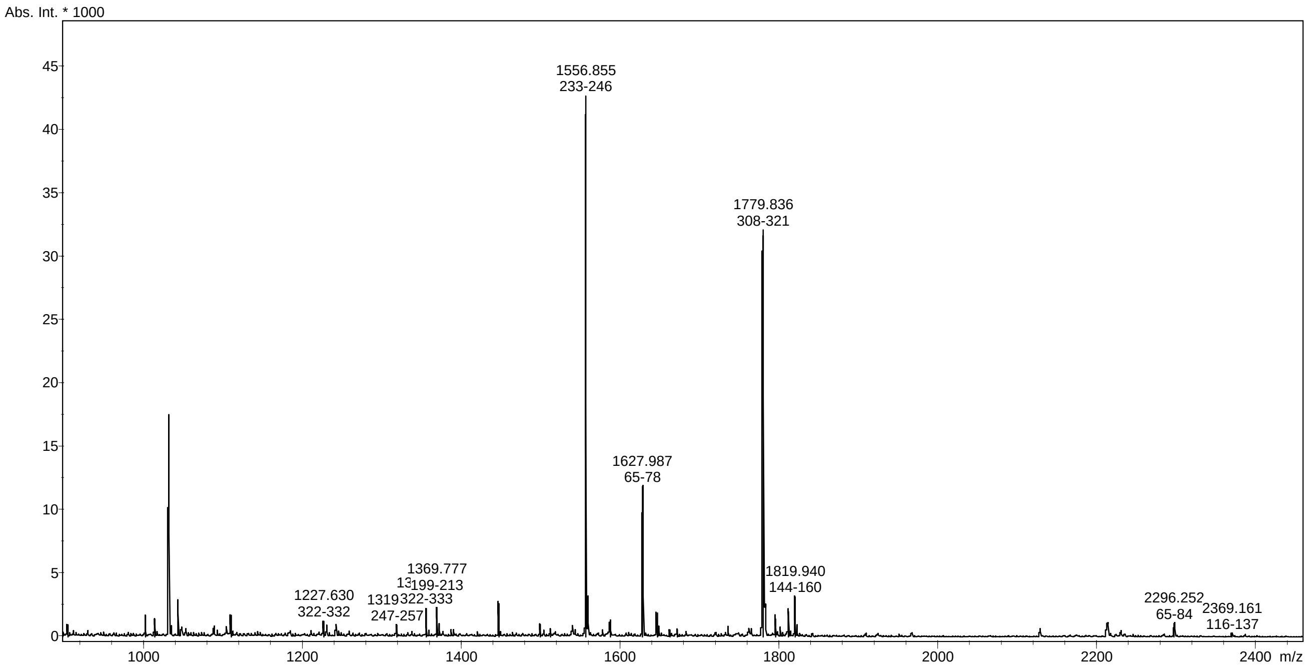

A representative MALDI-ToF PMF spectrum of spot 31

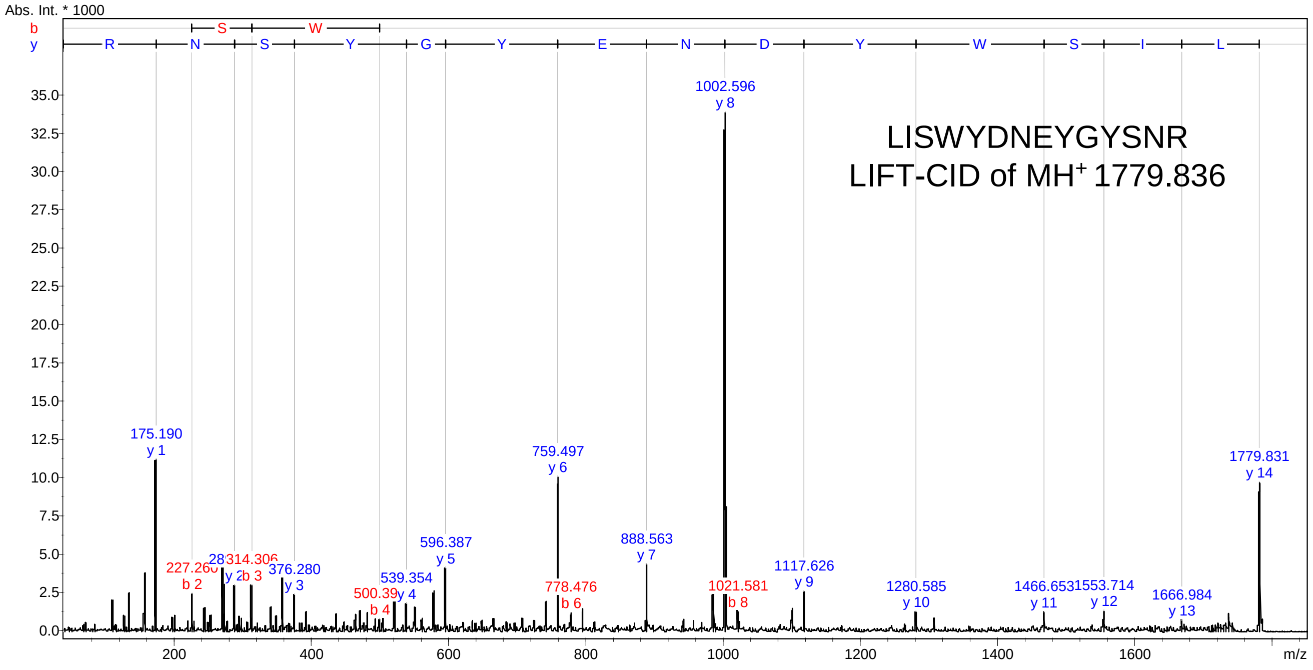

MSMS sequence analysis from the fragmentation of a precursor ion m/z 1779.836 by MALDI-ToF/ToF mass spectrometer

SPOT 32

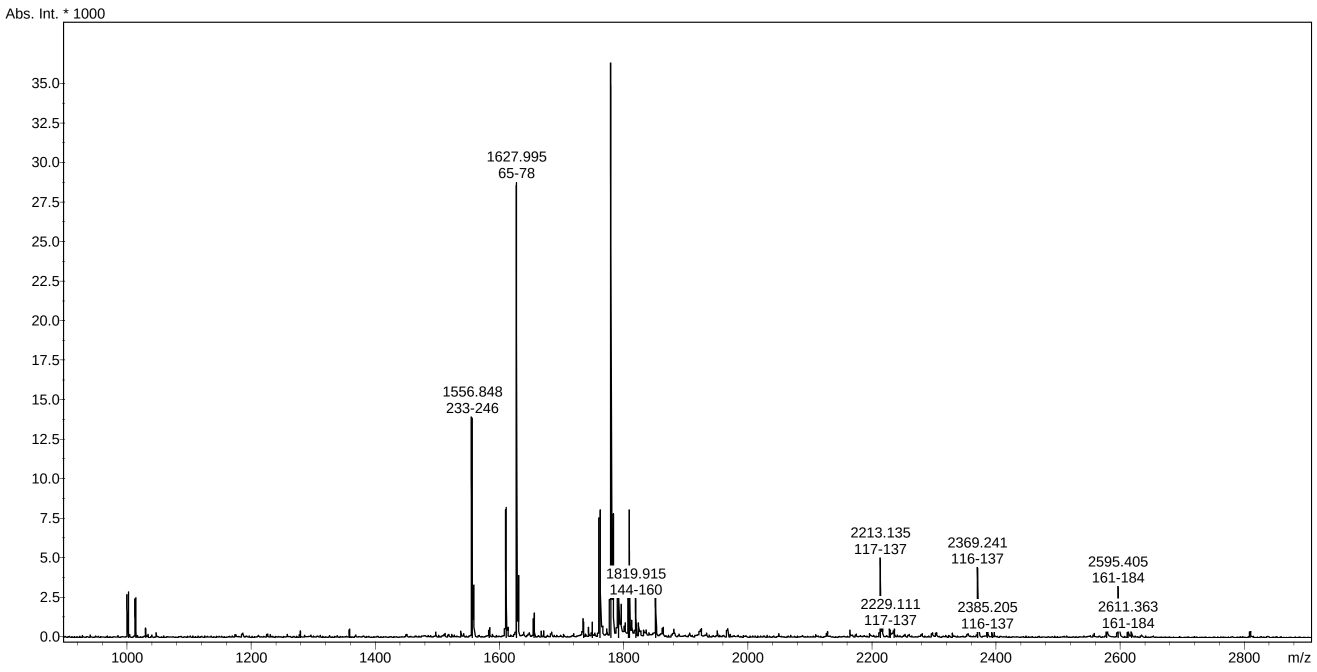

A representative MALDI-ToF PMF spectrum of spot 32

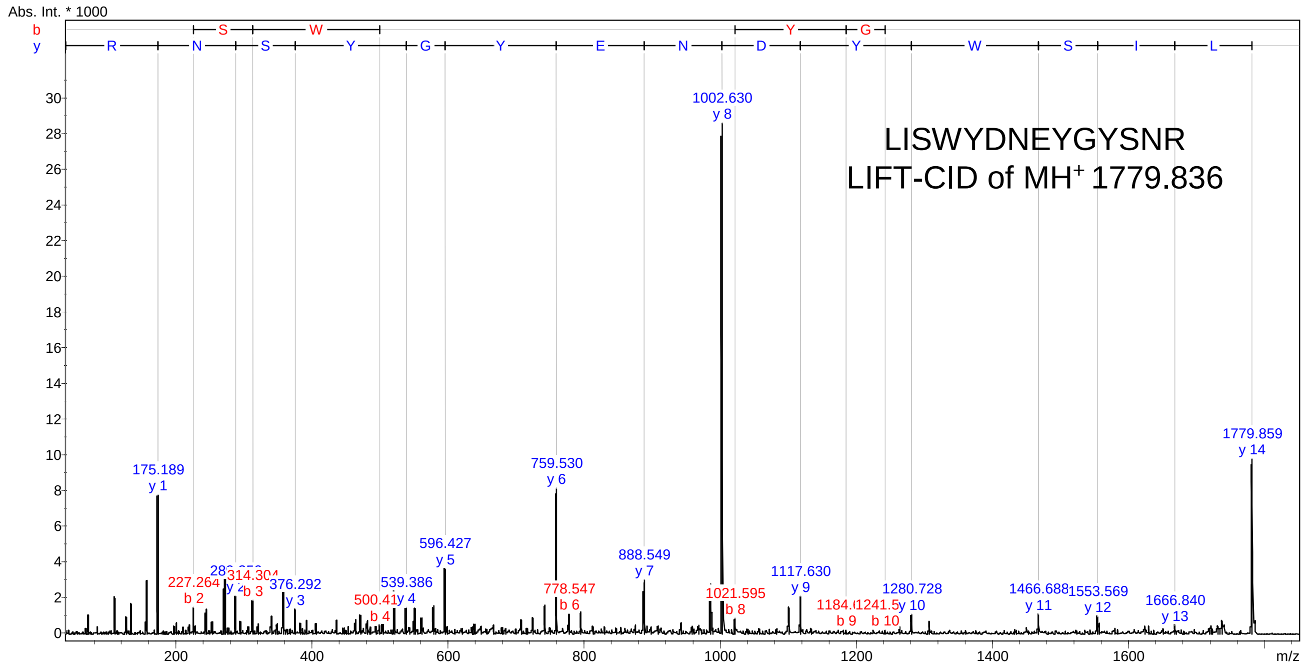

MSMS sequence analysis from the fragmentation of a precursor ion m/z 1779.836 by MALDI-ToF/ToF mass spectrometer

SPOT 33

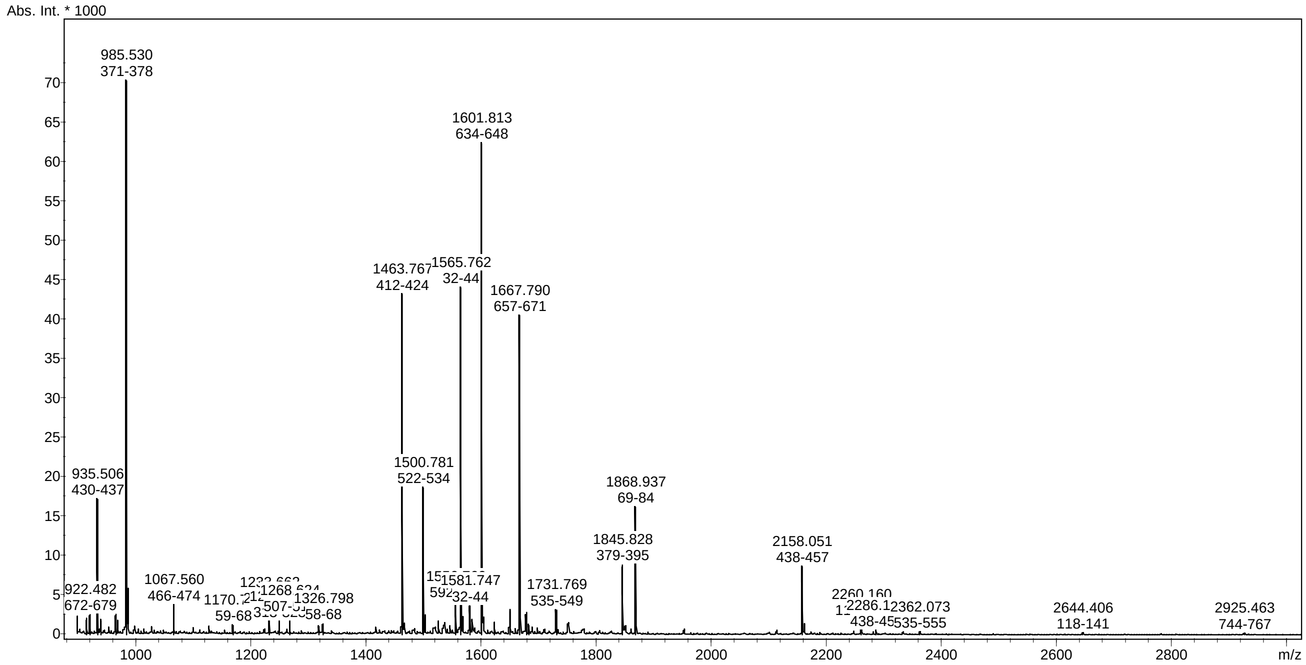

A representative MALDI-ToF PMF spectrum of spot 33

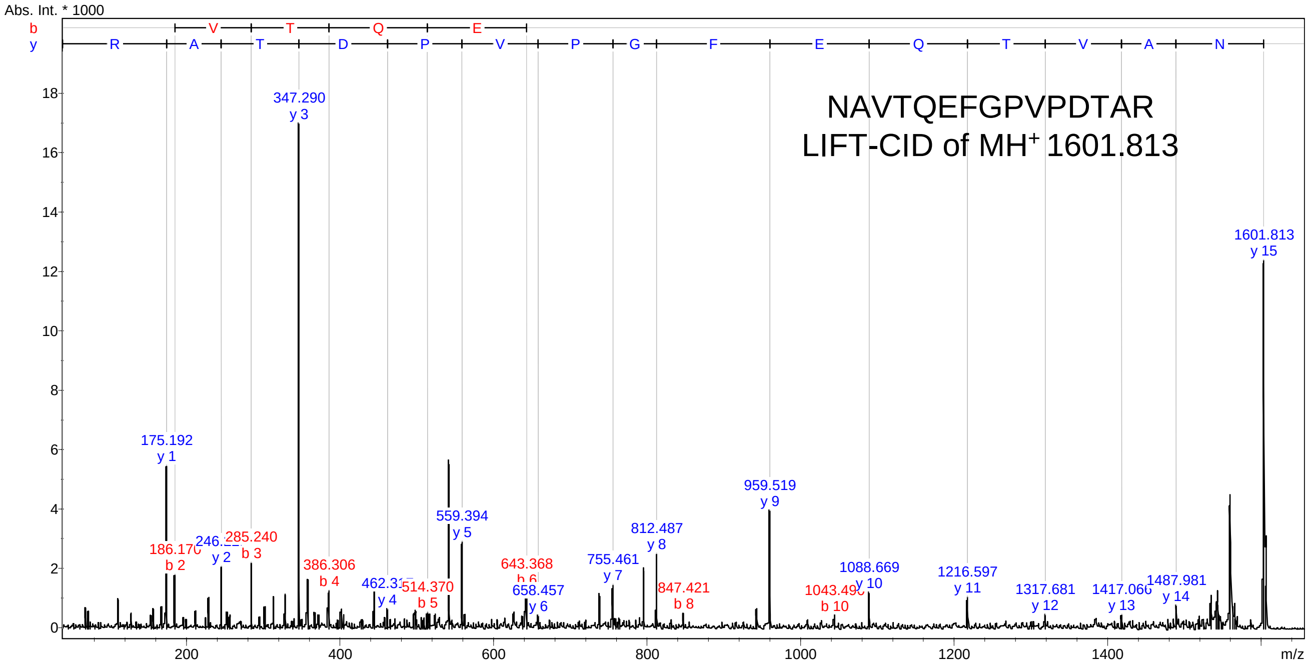

MSMS sequence analysis from the fragmentation of a precursor ion m/z 1601.813 by MALDI-ToF/ToF mass spectrometer

SPOT 34

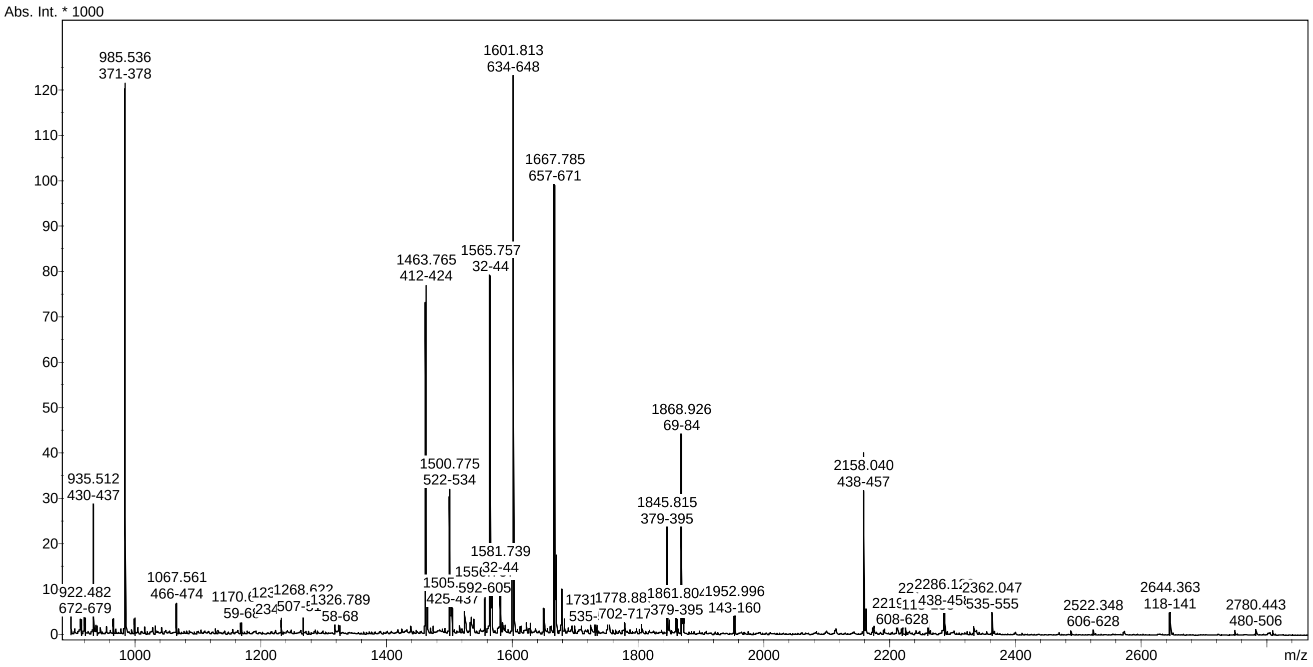

A representative MALDI-ToF PMF spectrum of spot 34

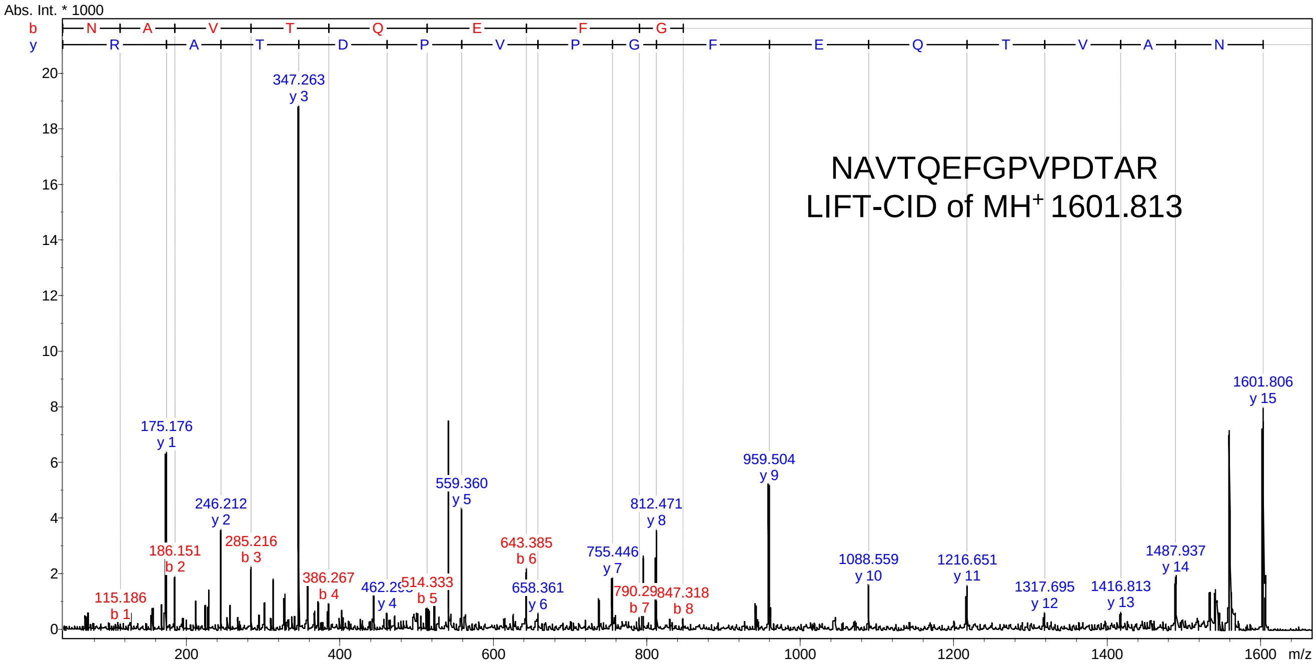

MSMS sequence analysis from the fragmentation of a precursor ion m/z 1601.813 by MALDI-ToF/ToF mass spectrometer

SPOT 35

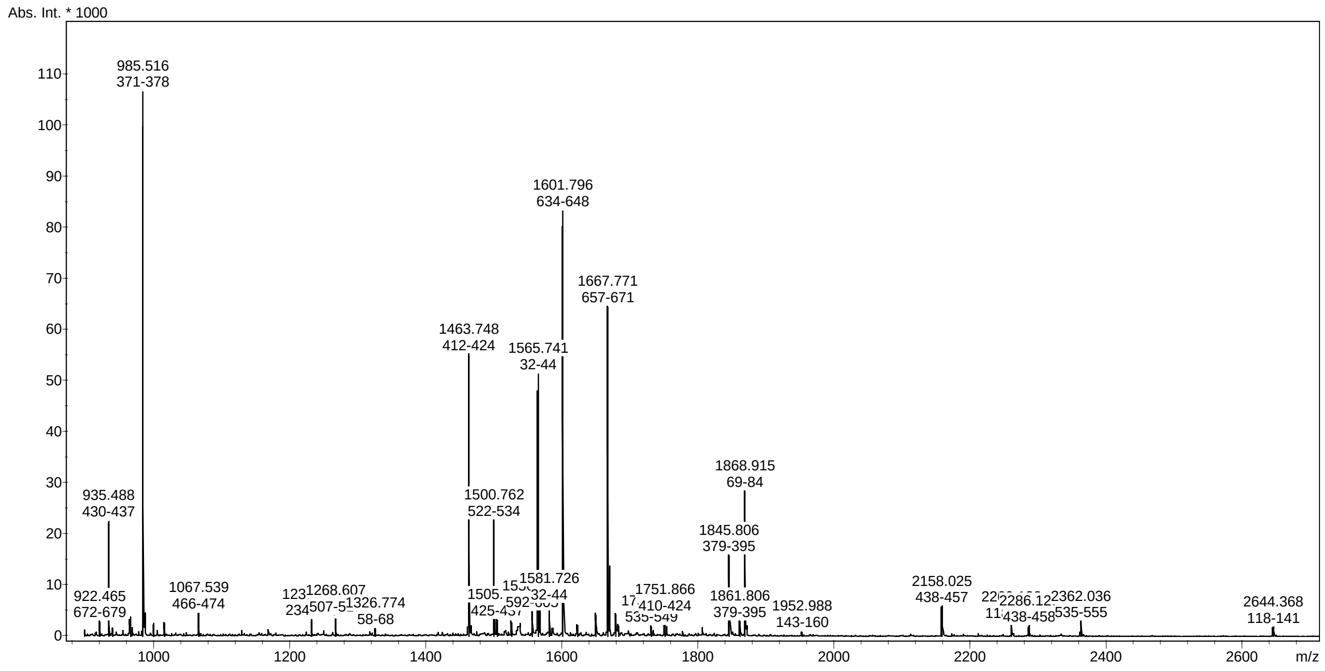

A representative MALDI-ToF PMF spectrum of spot 35

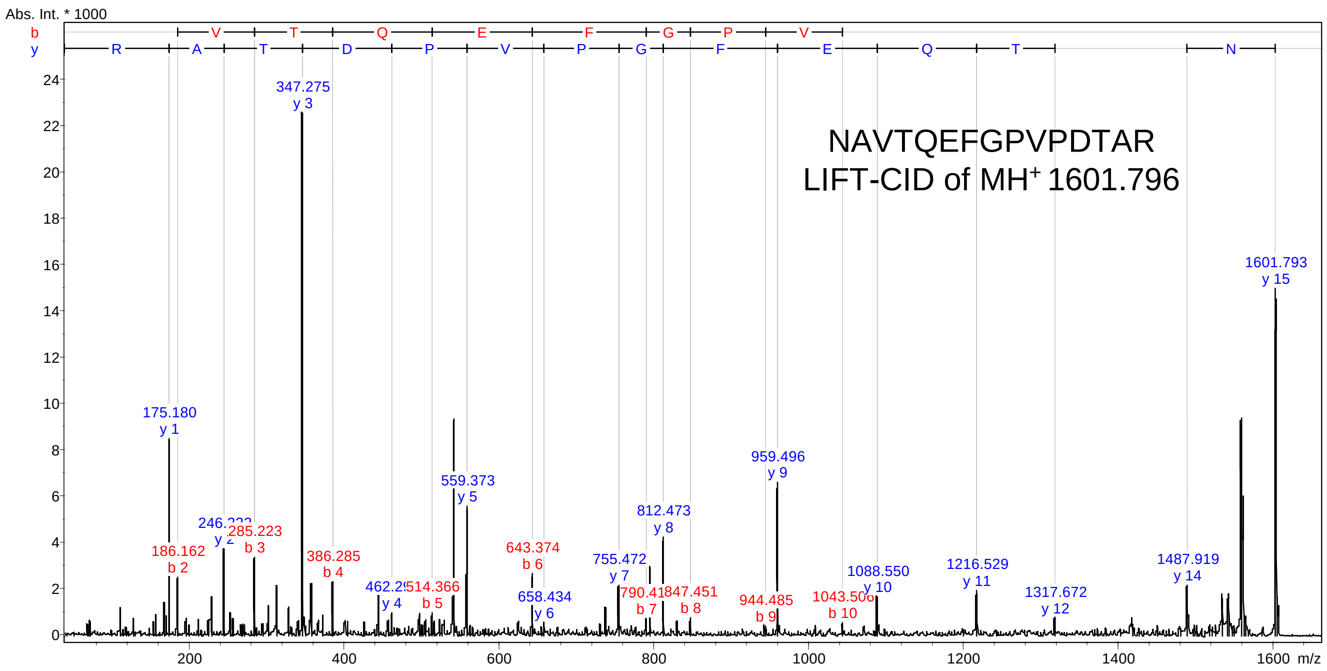

MSMS sequence analysis from the fragmentation of a precursor ion m/z 1601.796 by MALDI-ToF/ToF mass spectrometer

SPOT 36

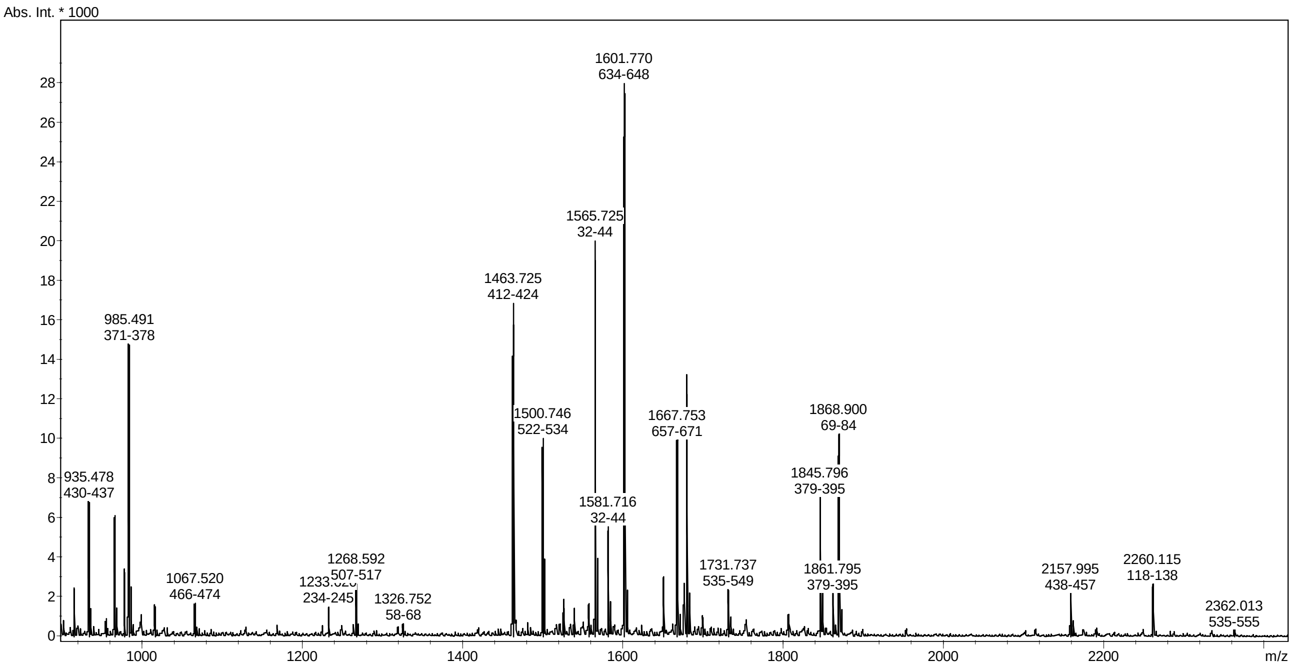

A representative MALDI-ToF PMF spectrum of spot 36

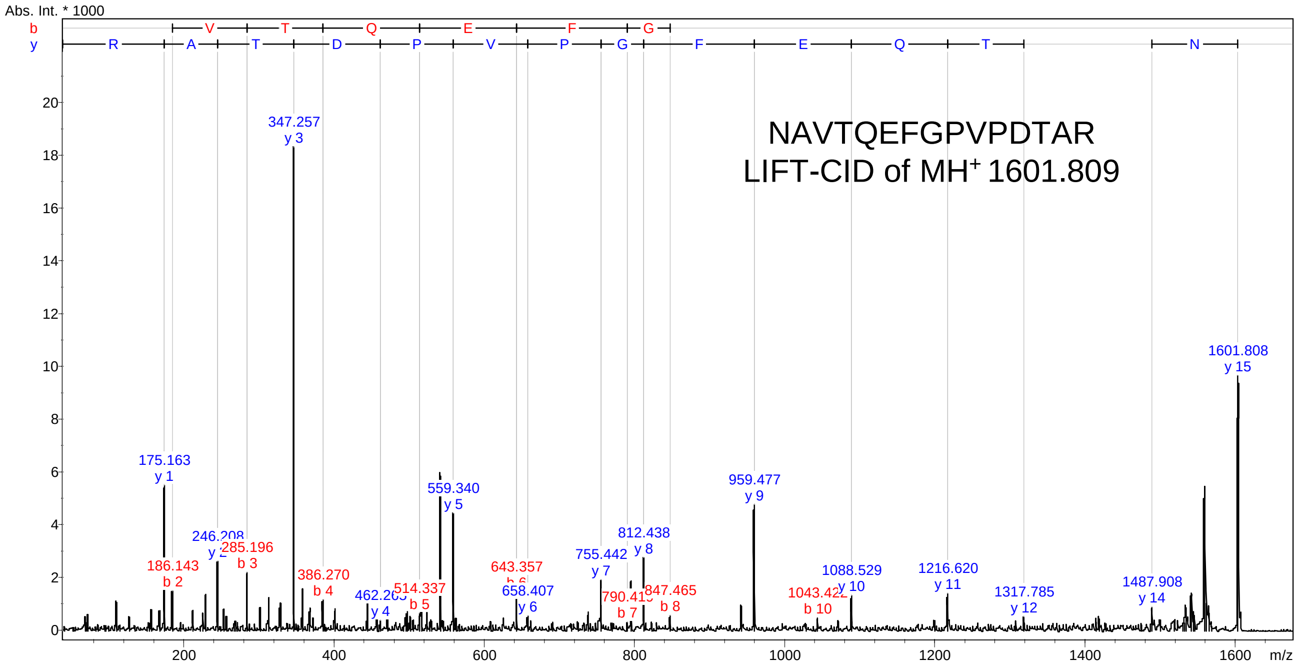

MSMS sequence analysis from the fragmentation of a precursor ion m/z 1601.809 by MALDI-ToF/ToF mass spectrometer

SPOT 37

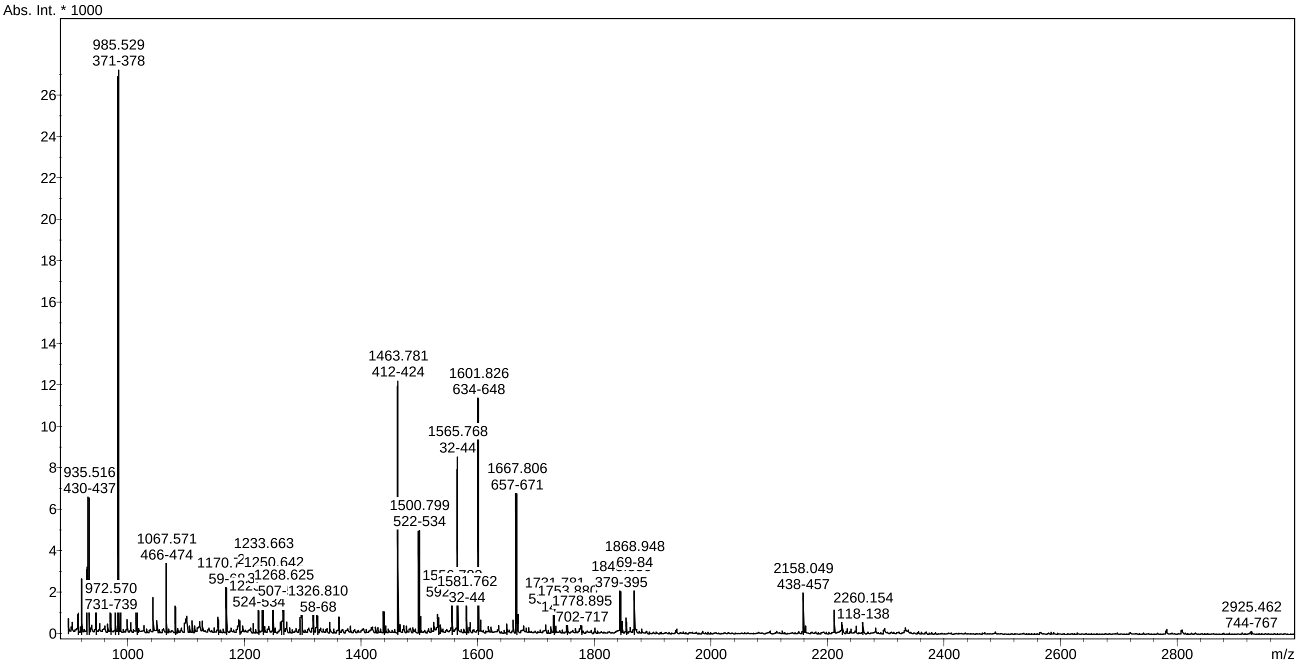

A representative MALDI-ToF PMF spectrum of spot 37

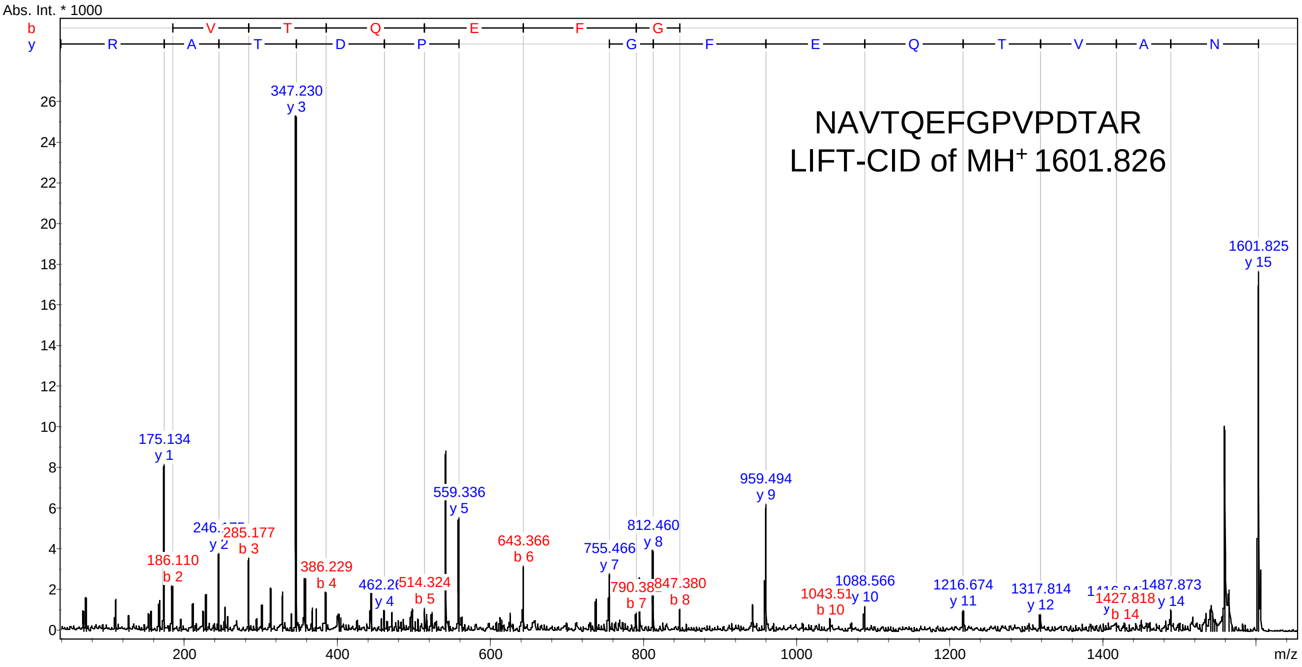

MSMS sequence analysis from the fragmentation of a precursor ion m/z 1601.826 by MALDI-ToF/ToF mass spectrometer

SPOT 38

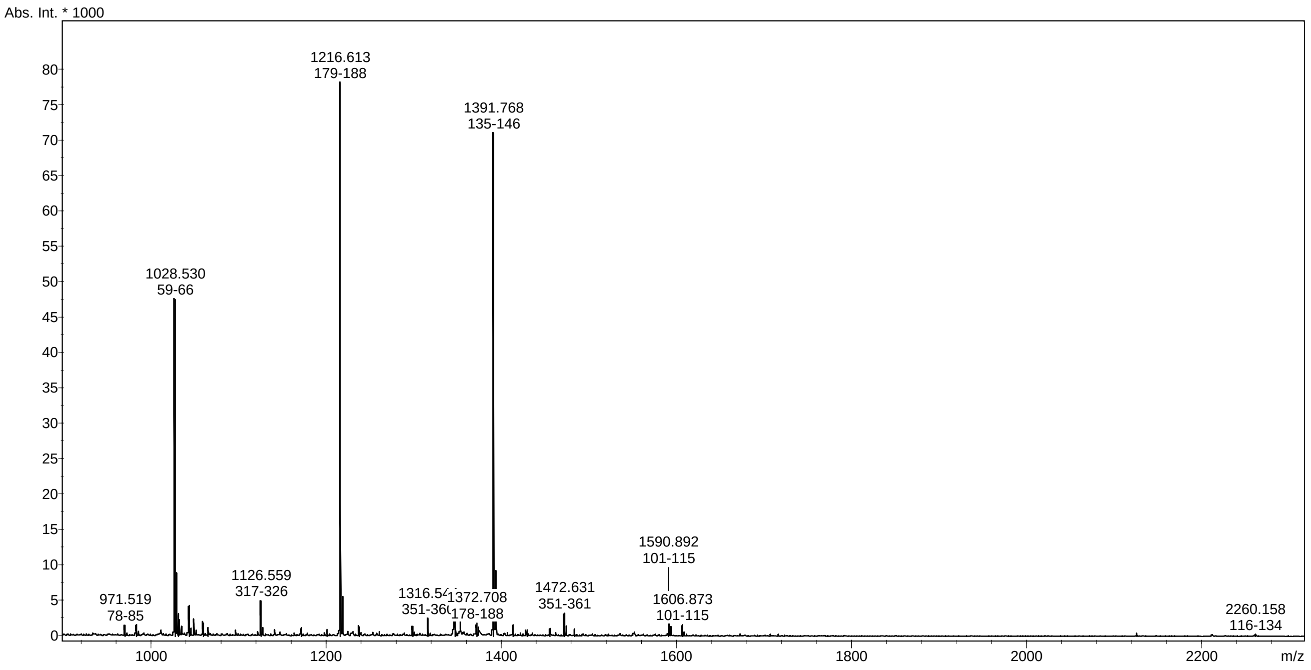

A representative MALDI-ToF PMF spectrum of spot 38

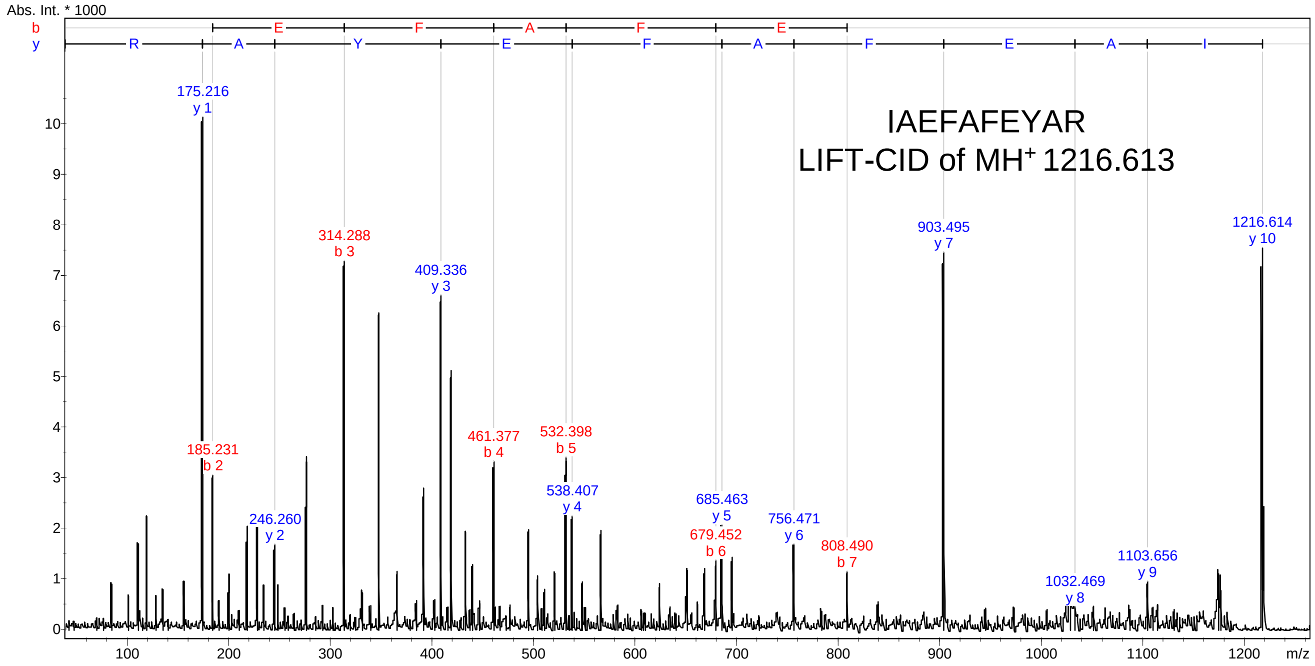

MSMS sequence analysis from the fragmentation of a precursor ion m/z 1216.613 by MALDI-ToF/ToF mass spectrometer

SPOT 39

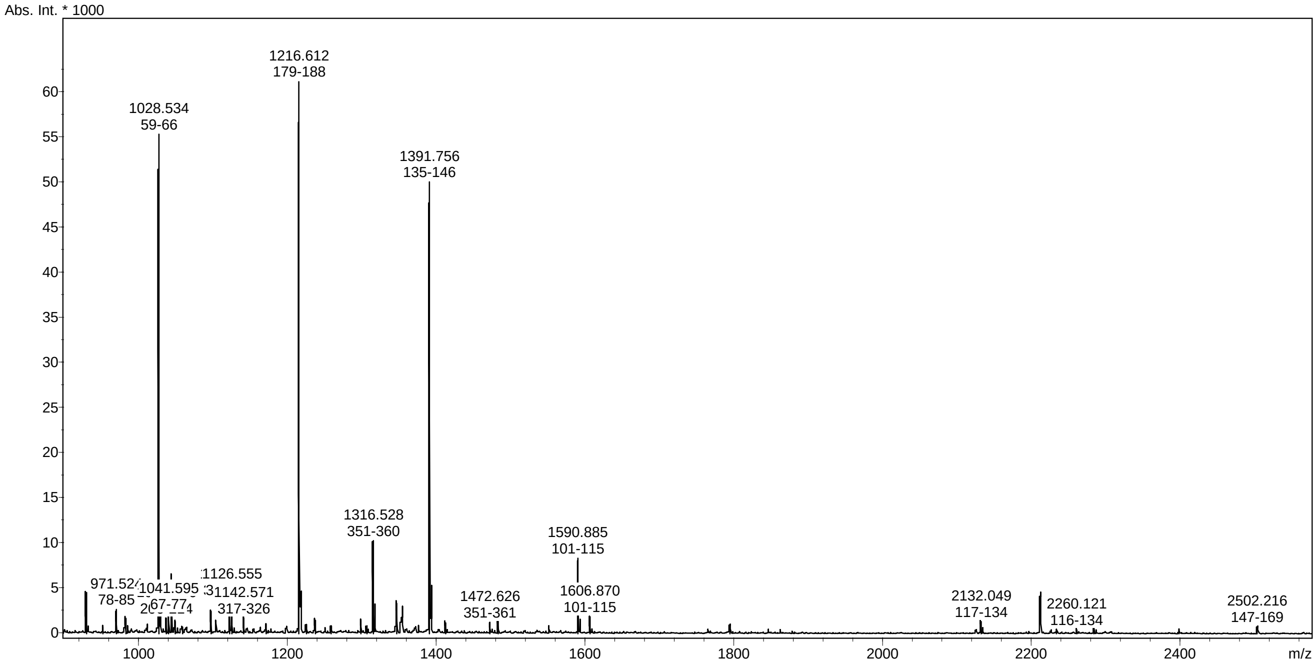

A representative MALDI-ToF PMF spectrum of spot 39

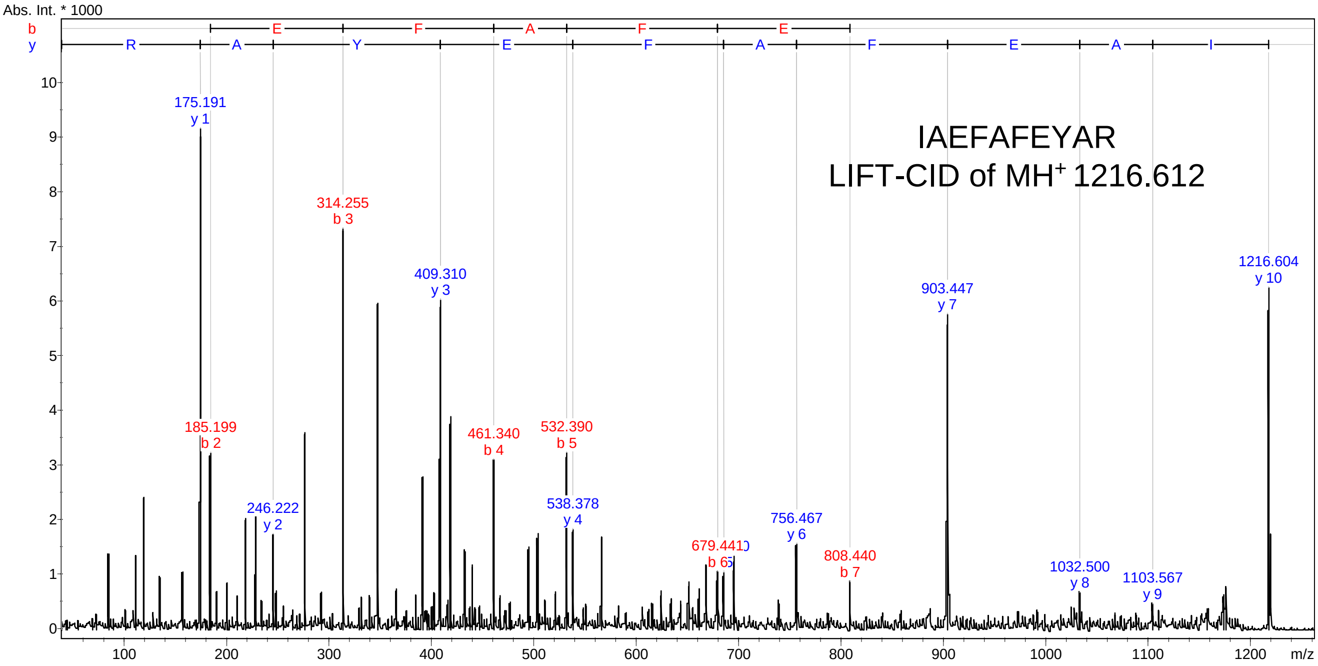

MSMS sequence analysis from the fragmentation of a precursor ion m/z 1216.612 by MALDI-ToF/ToF mass spectrometer

SPOT 40

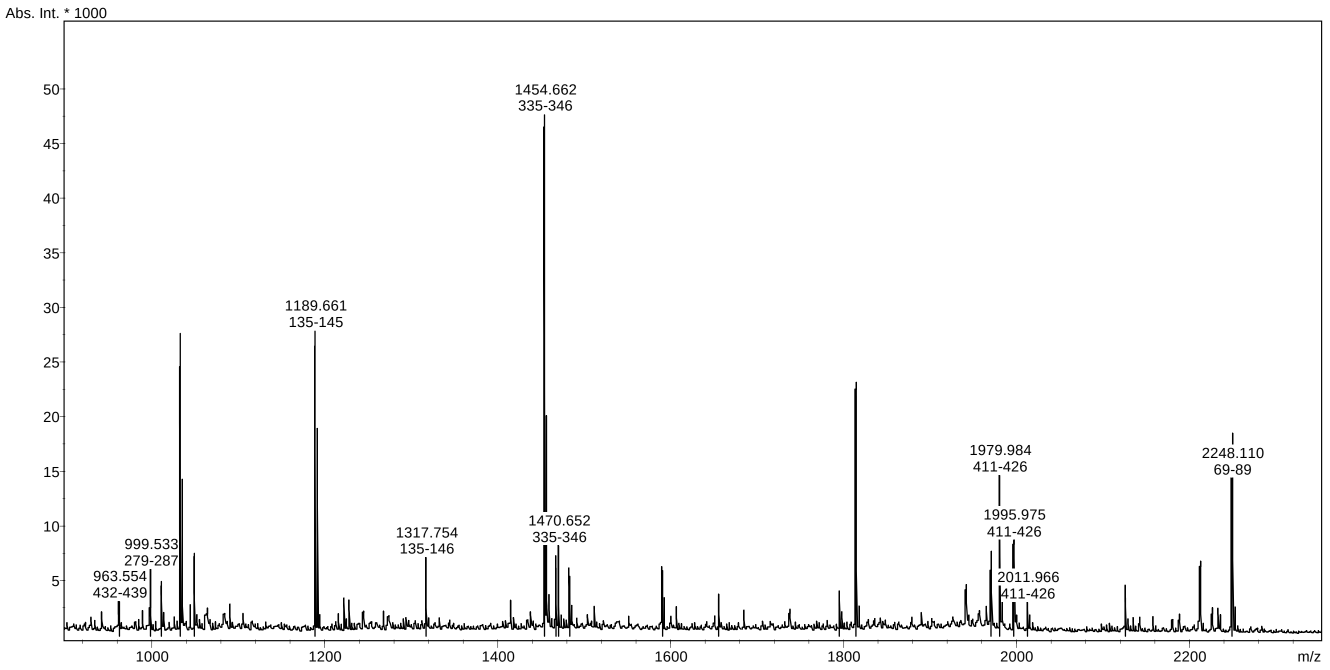

A representative MALDI-ToF PMF spectrum of spot 40

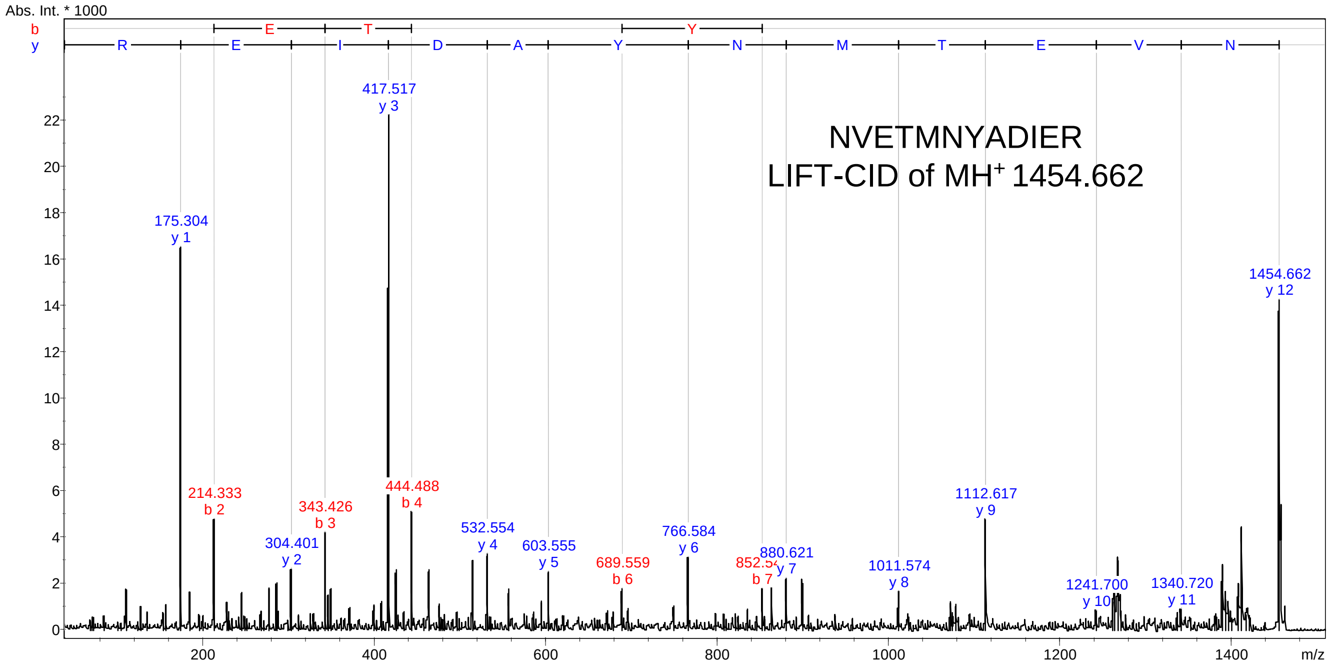

MSMS sequence analysis from the fragmentation of a precursor ion m/z 1454.662 by MALDI-ToF/ToF mass spectrometer

SPOT 41

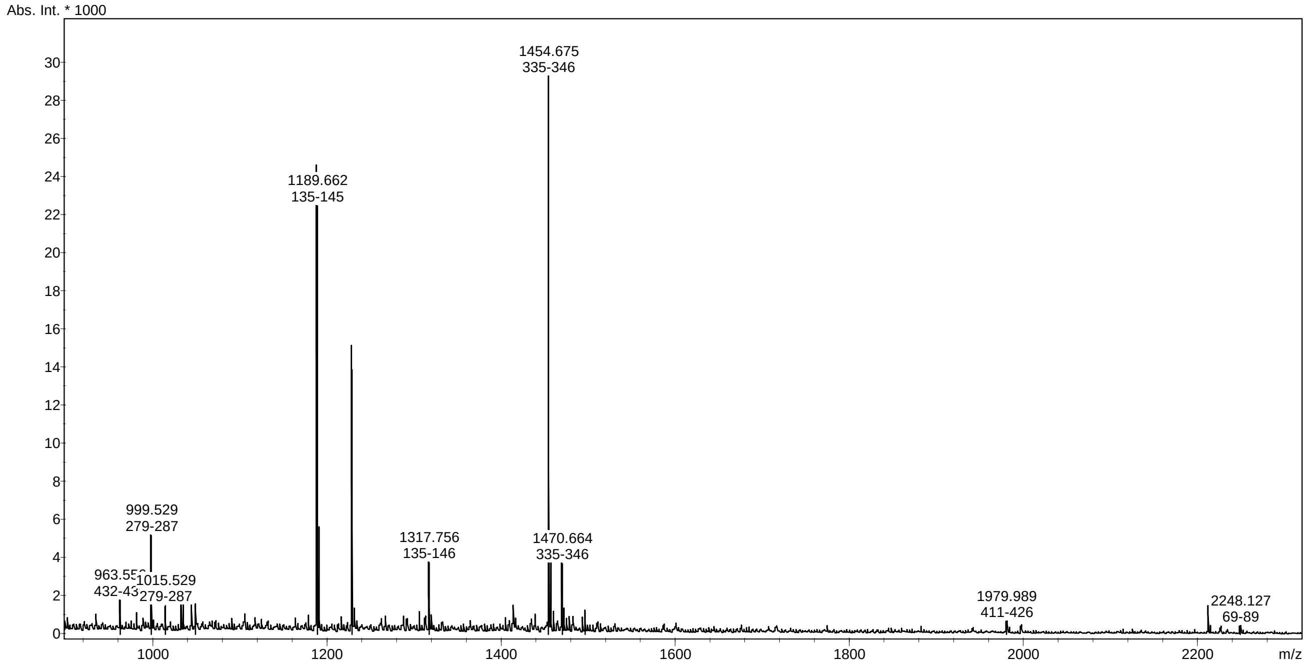

A representative MALDI-ToF PMF spectrum of spot 41

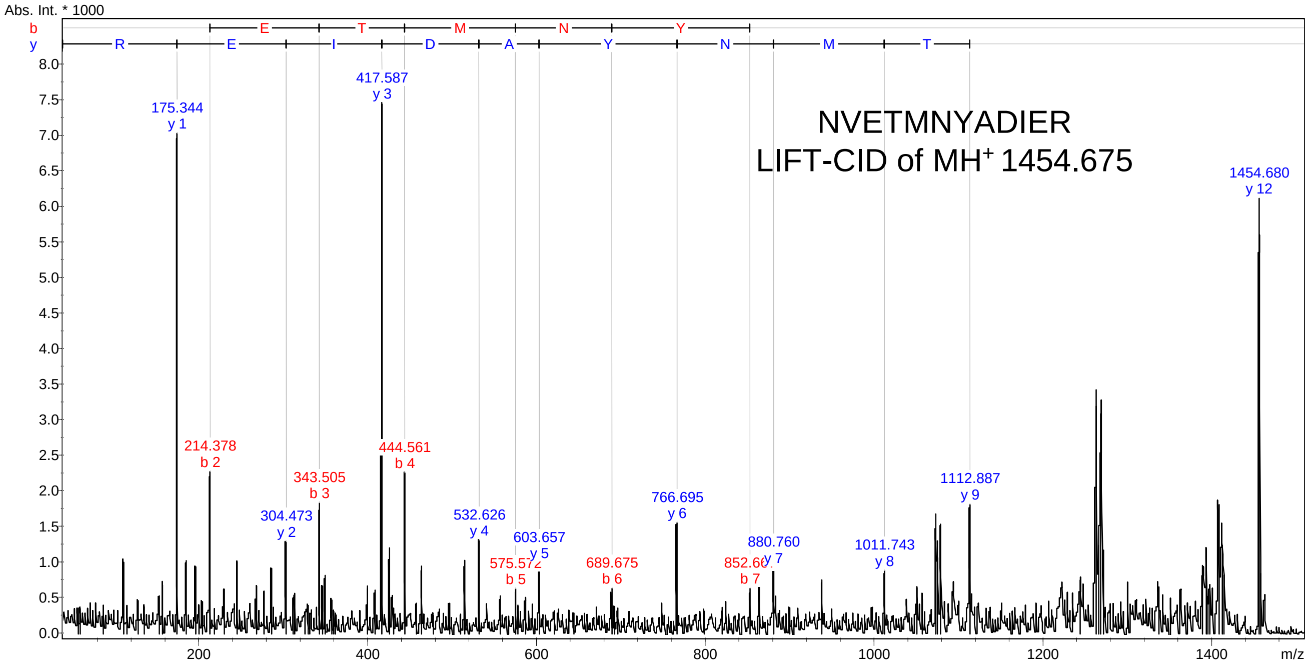

MSMS sequence analysis from the fragmentation of a precursor ion m/z 1454.675 by MALDI-ToF/ToF mass spectrometer

SPOT 42

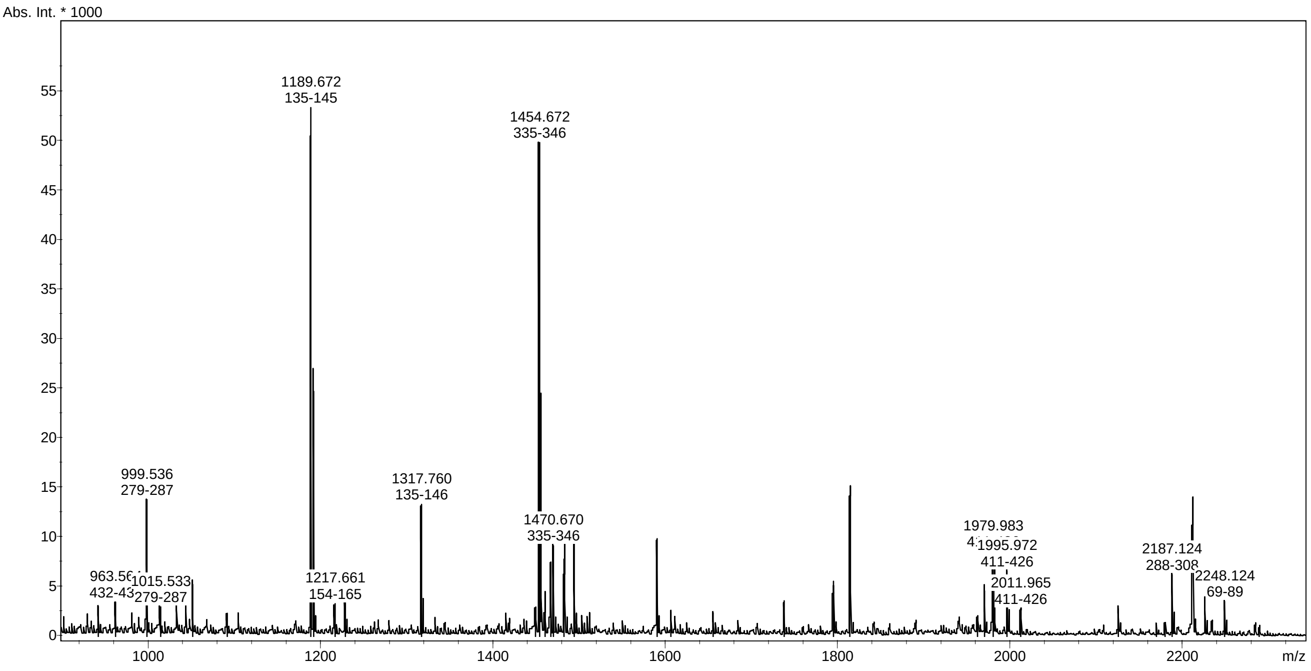

A representative MALDI-ToF PMF spectrum of spot 42

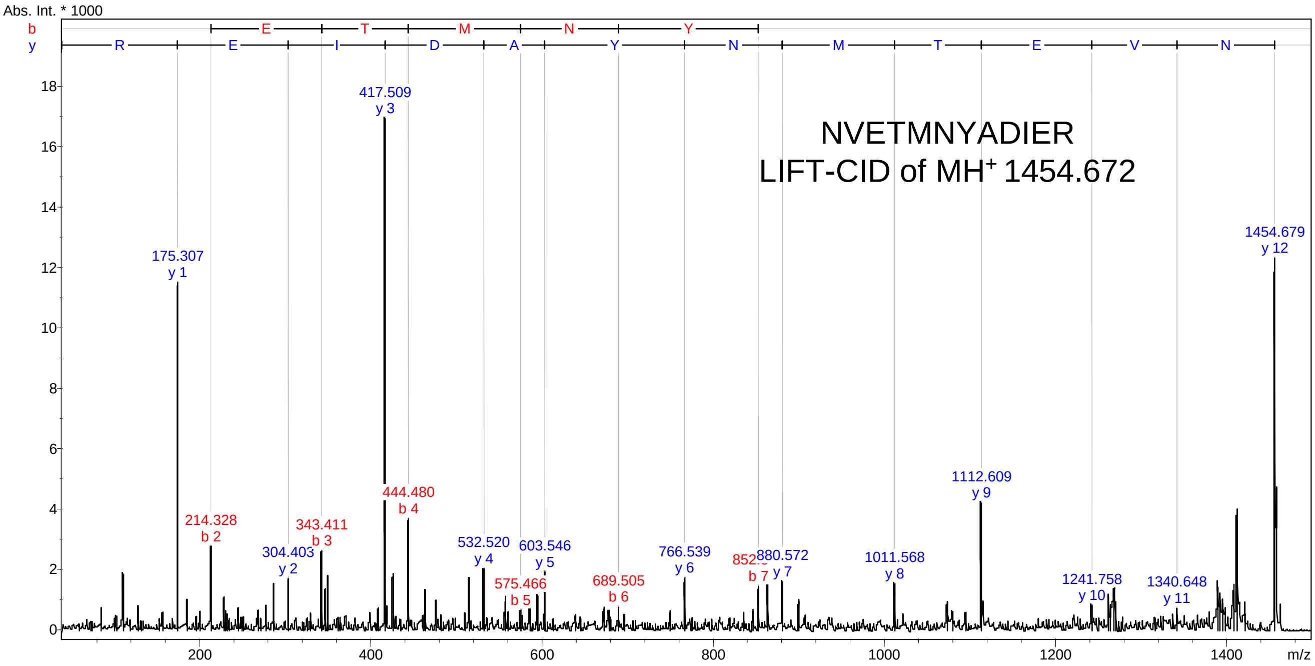

MSMS sequence analysis from the fragmentation of a precursor ion m/z 1454.672 by MALDI-ToF/ToF mass spectrometer

SPOT 43

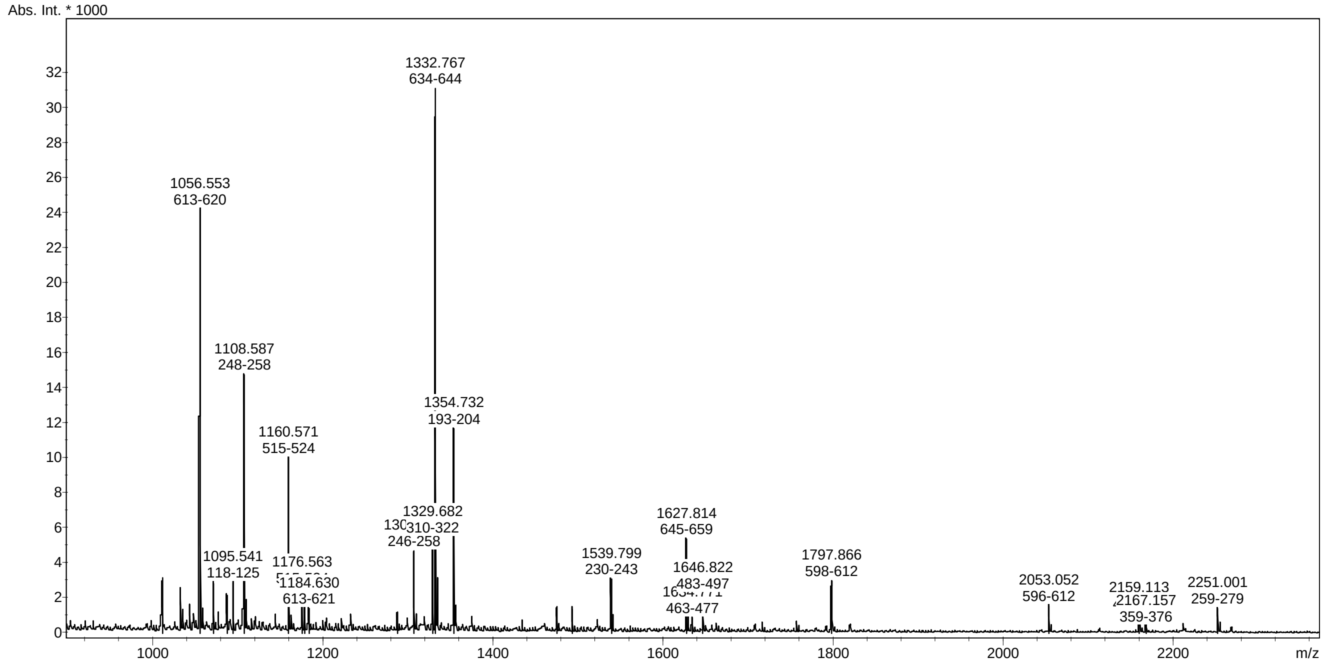

A representative MALDI-ToF PMF spectrum of spot 43

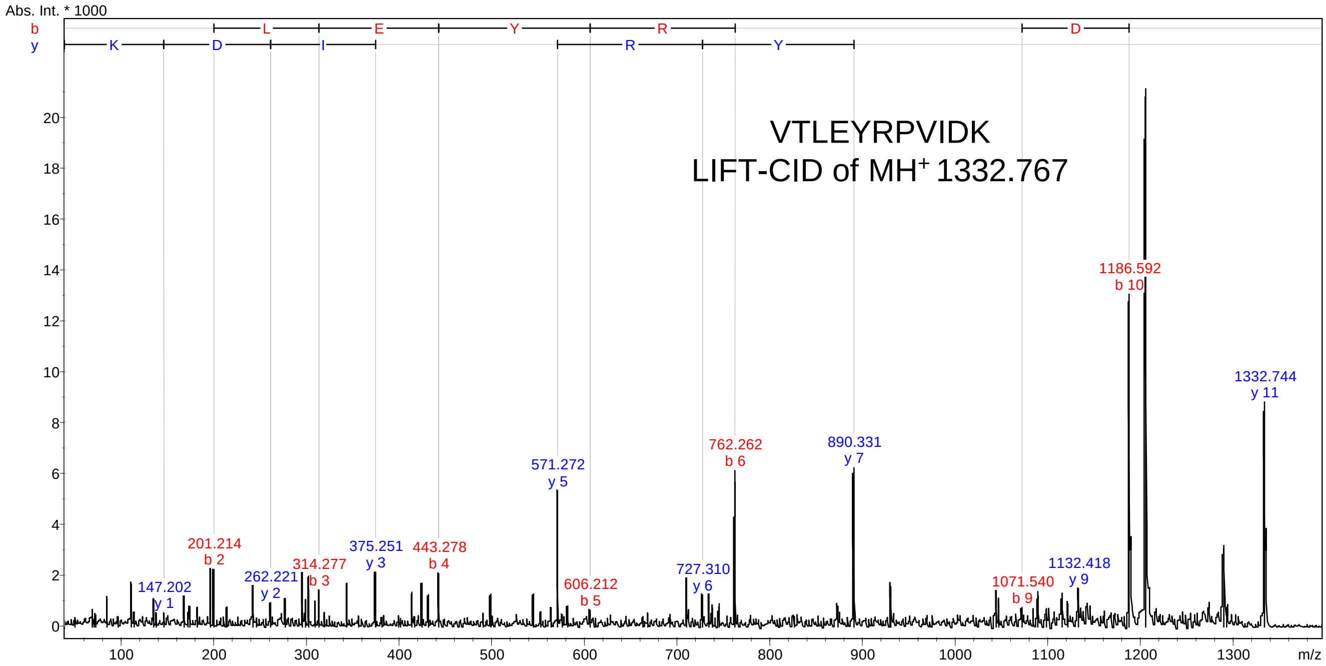

MSMS sequence analysis from the fragmentation of a precursor ion m/z 1332.767 by MALDI-ToF/ToF mass spectrometer

SPOT 44

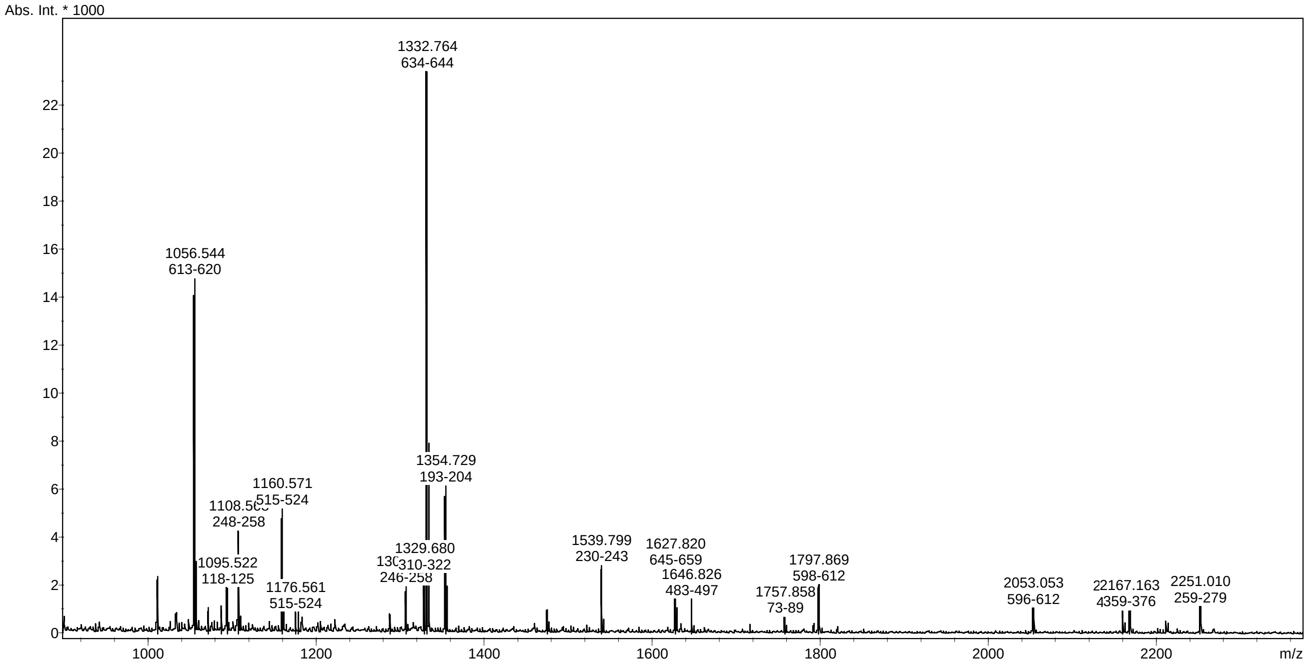

A representative MALDI-ToF PMF spectrum of spot 44

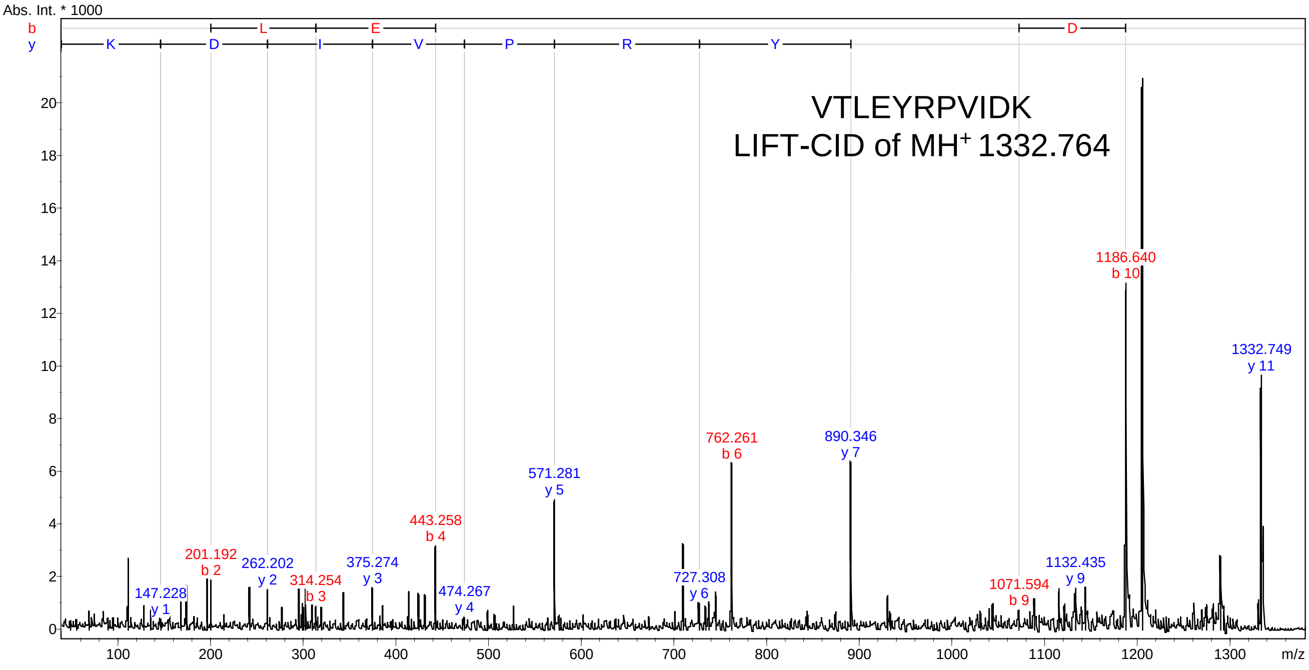

MSMS sequence analysis from the fragmentation of a precursor ion m/z 1332.764 by MALDI-ToF/ToF mass spectrometer

SPOT 45

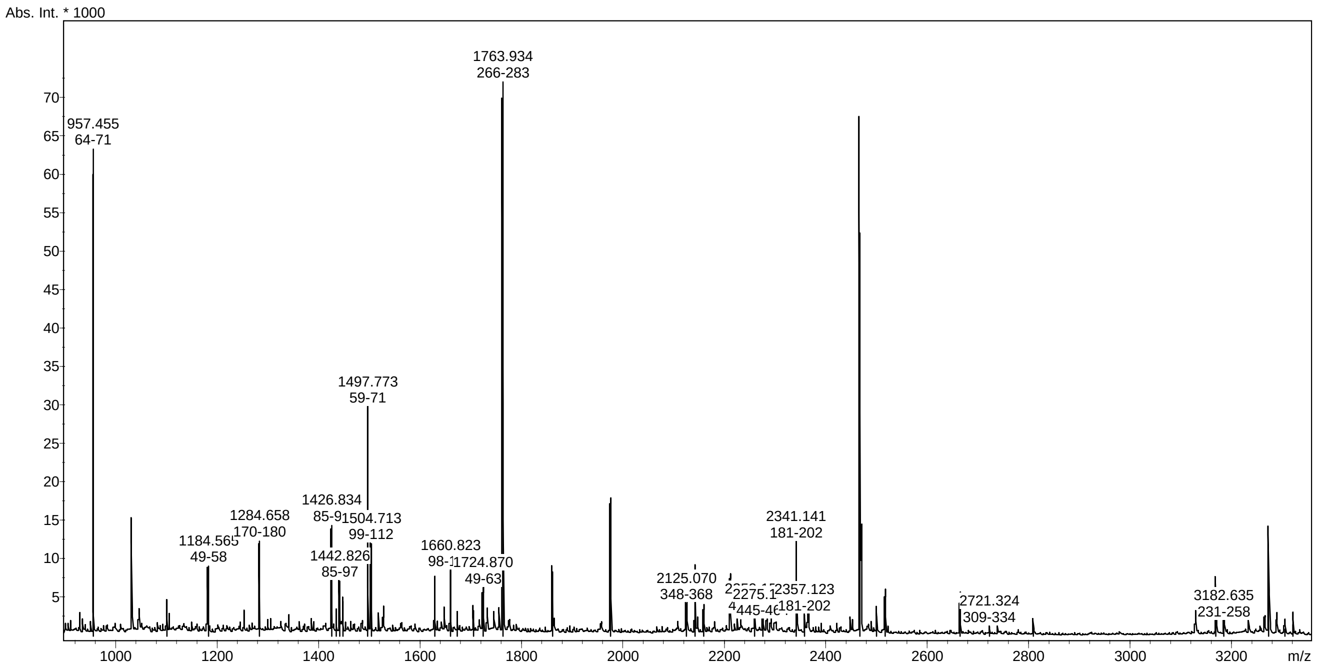

A representative MALDI-ToF PMF spectrum of spot 45

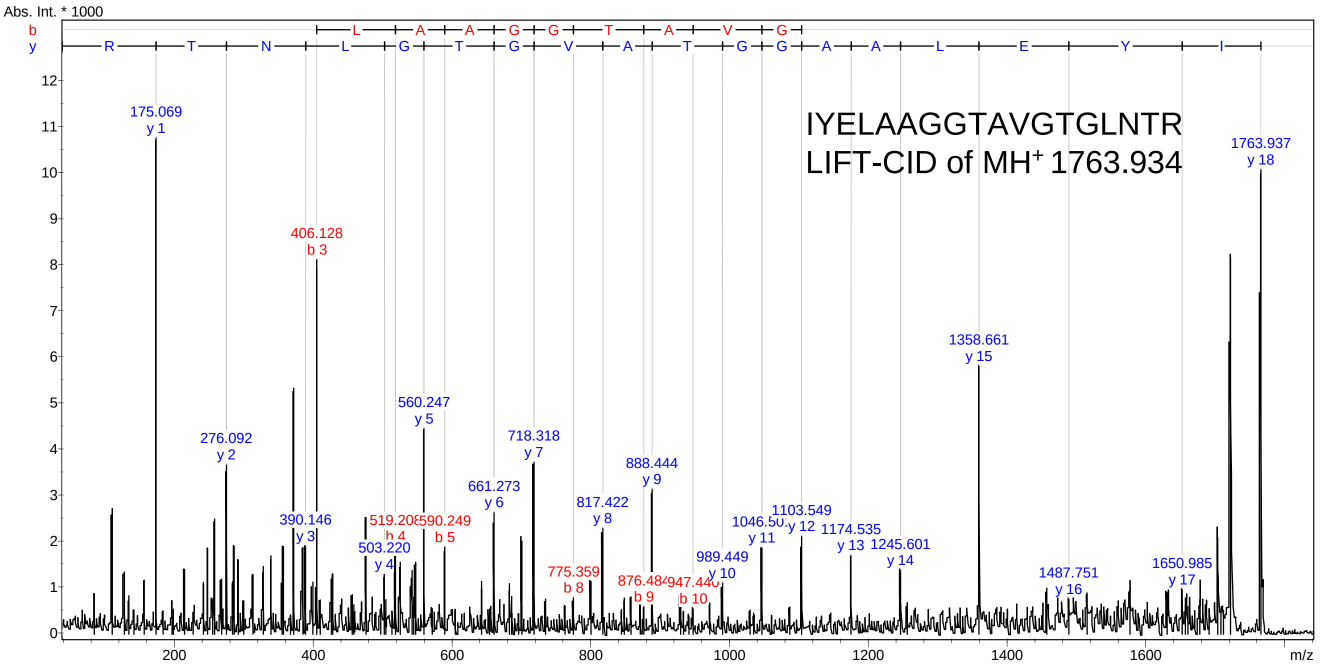

MSMS sequence analysis from the fragmentation of a precursor ion m/z 1763.934 by MALDI-ToF/ToF mass spectrometer

SPOT 46

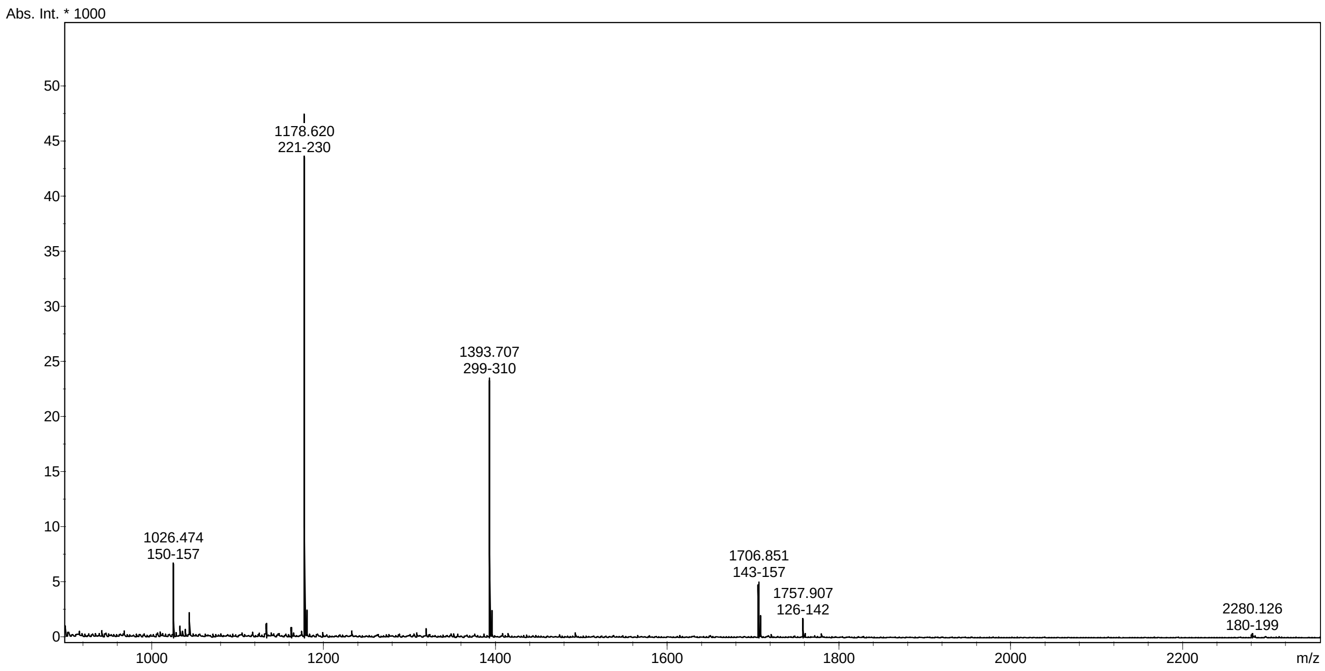

A representative MALDI-ToF PMF spectrum of spot 46

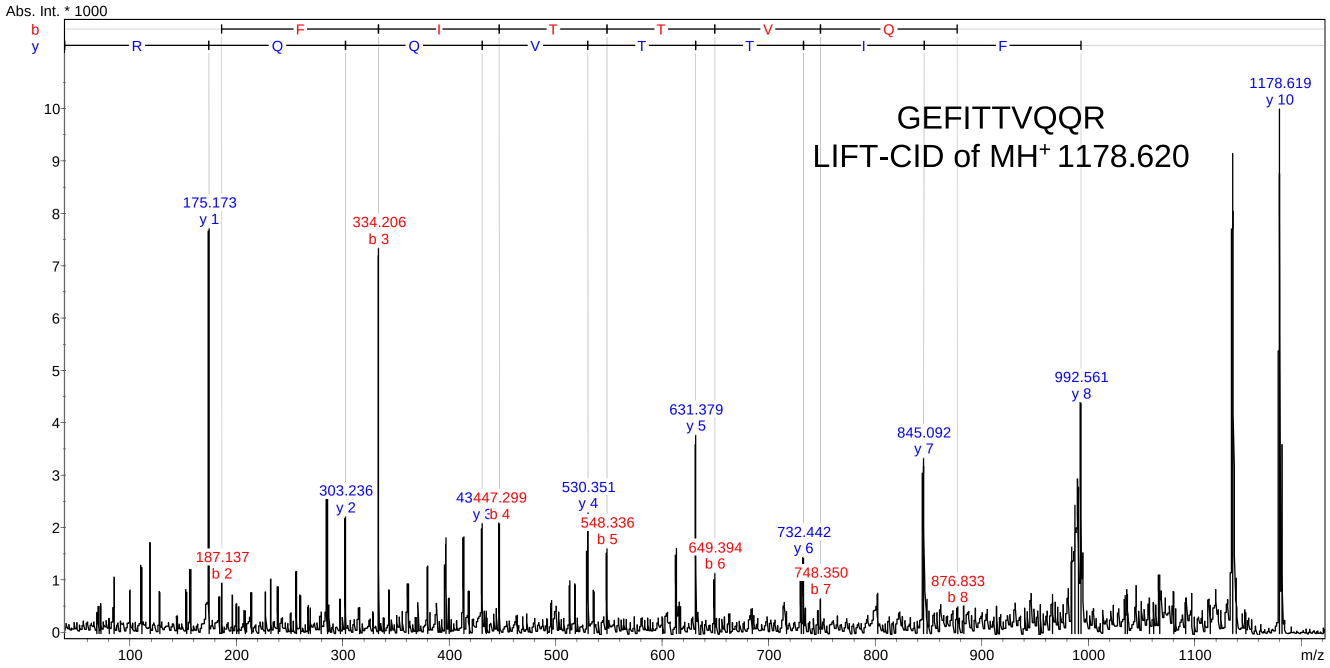

MSMS sequence analysis from the fragmentation of a precursor ion m/z 1178.620 by MALDI-ToF/ToF mass spectrometer

SPOT 47

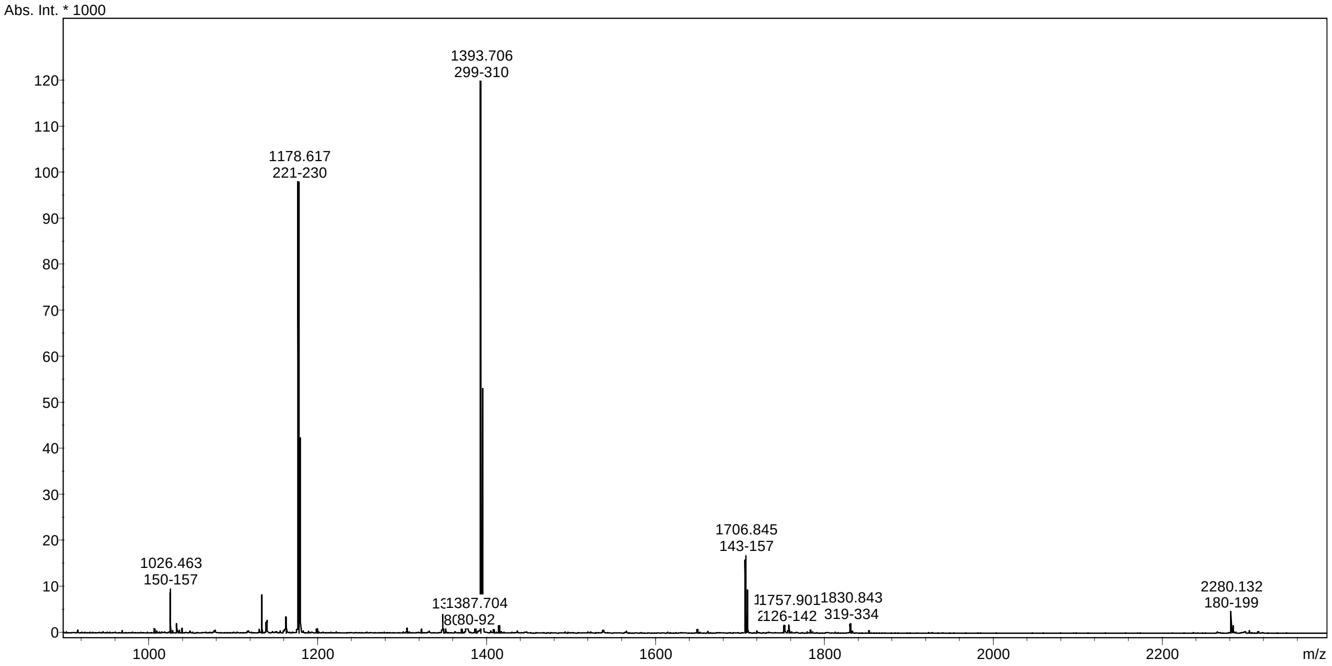

A representative MALDI-ToF PMF spectrum of spot 47

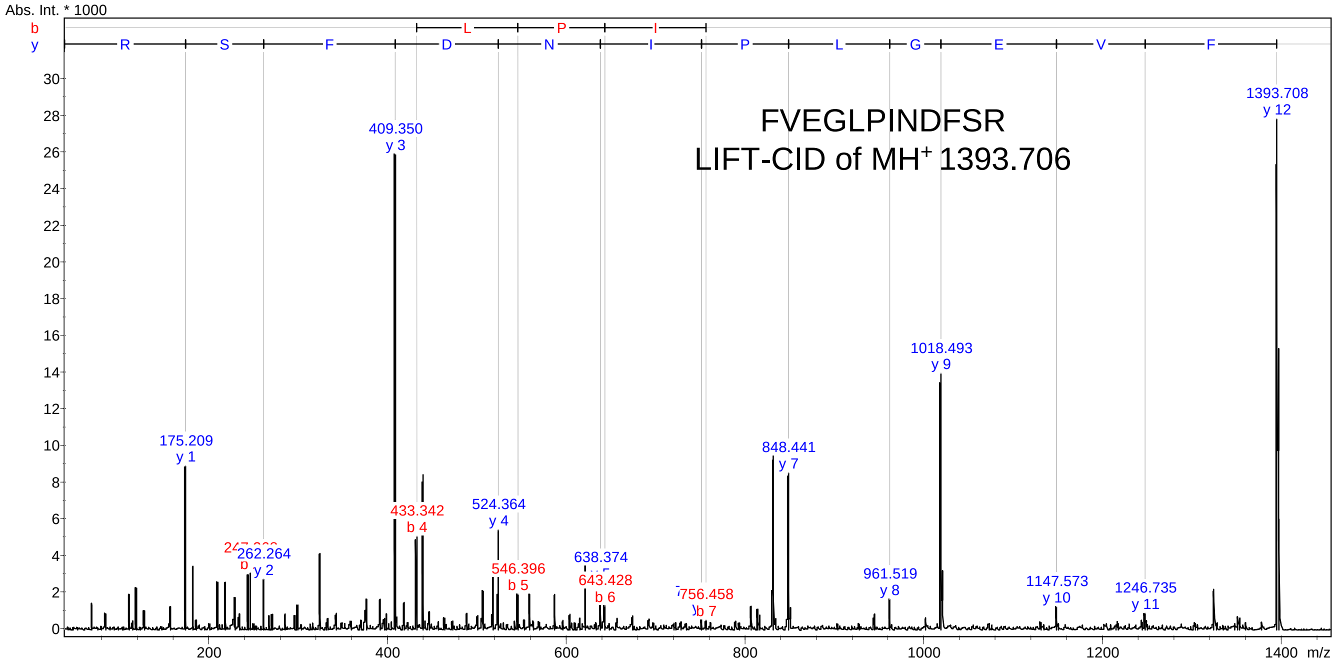

MSMS sequence analysis from the fragmentation of a precursor ion m/z 1393.706 by MALDI-ToF/ToF mass spectrometer

SPOT 49

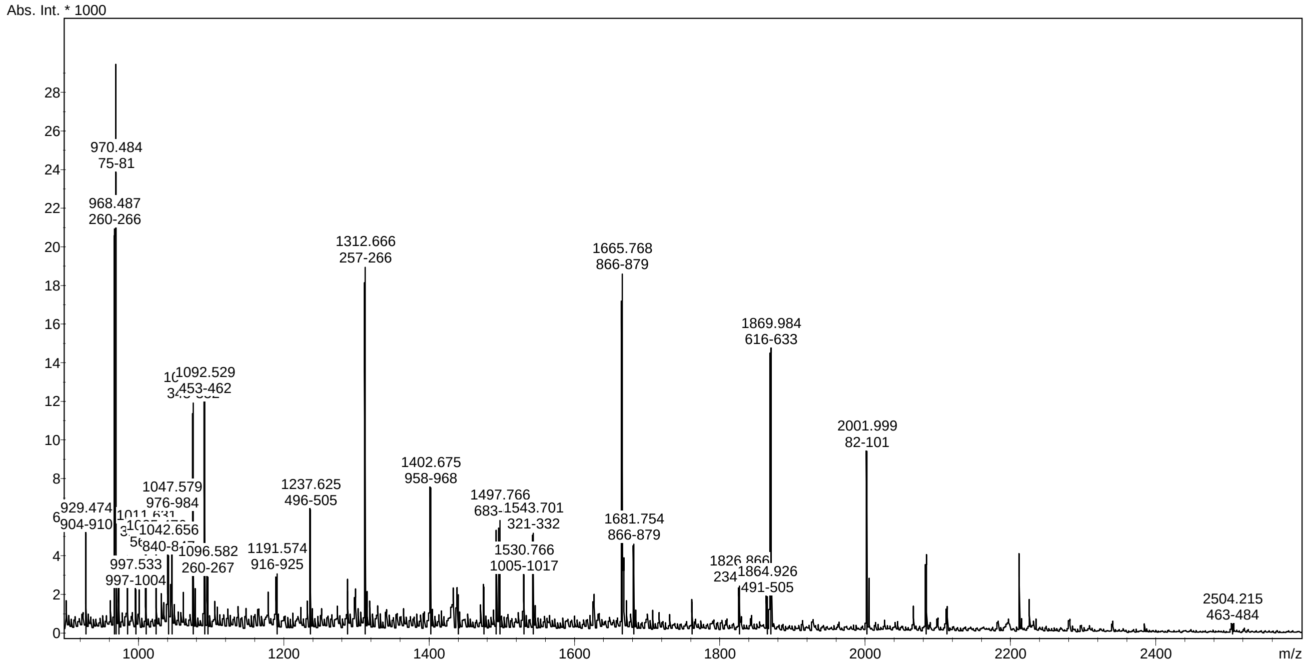

A representative MALDI-ToF PMF spectrum of spot 49

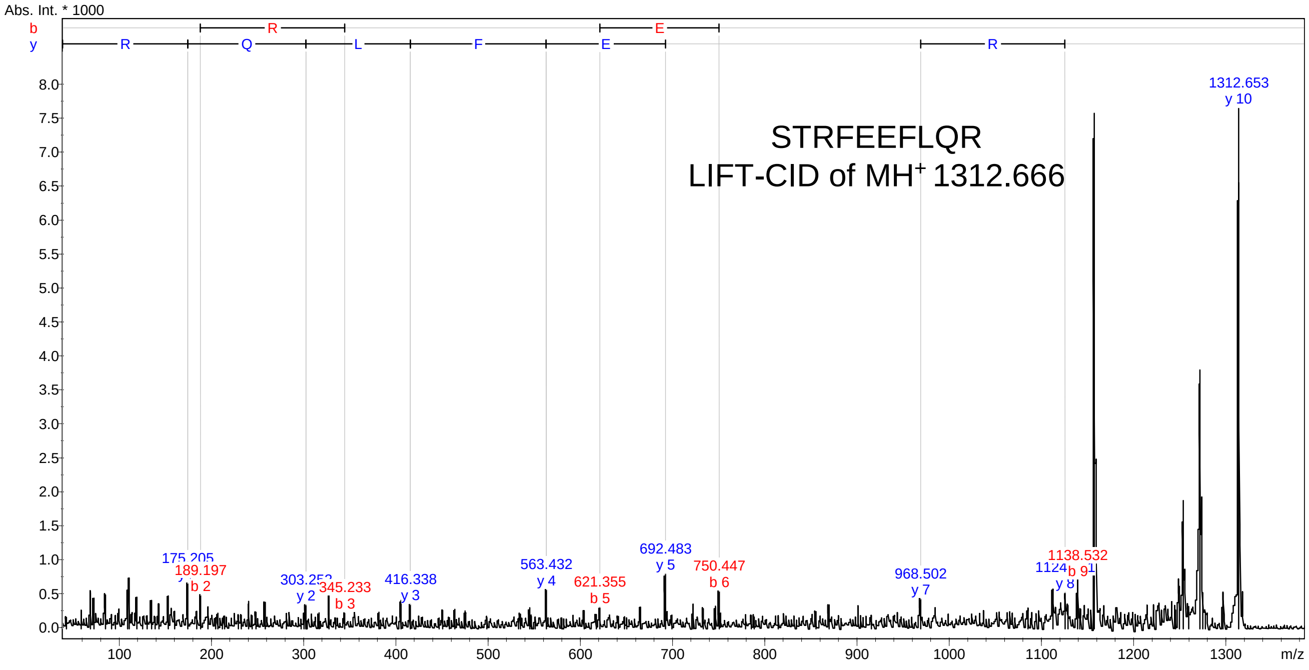

MSMS sequence analysis from the fragmentation of a precursor ion m/z 1312.666 by MALDI-ToF/ToF mass spectrometer

SPOT 50

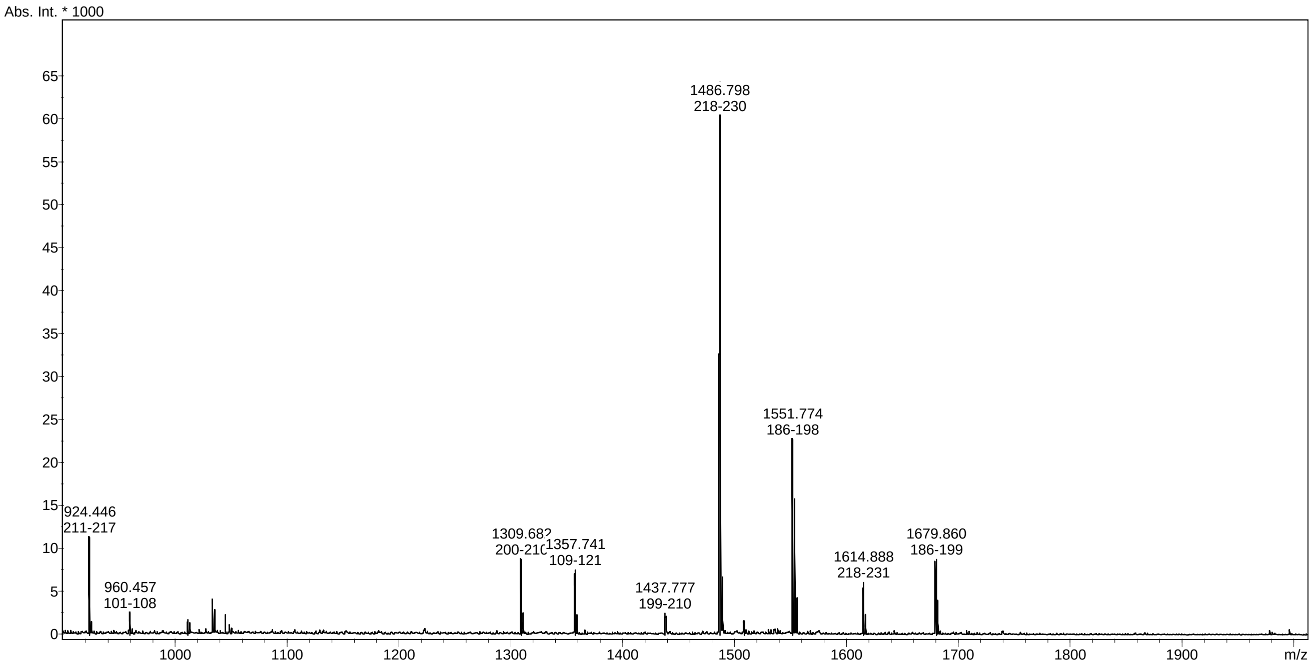

A representative MALDI-ToF PMF spectrum of spot 50

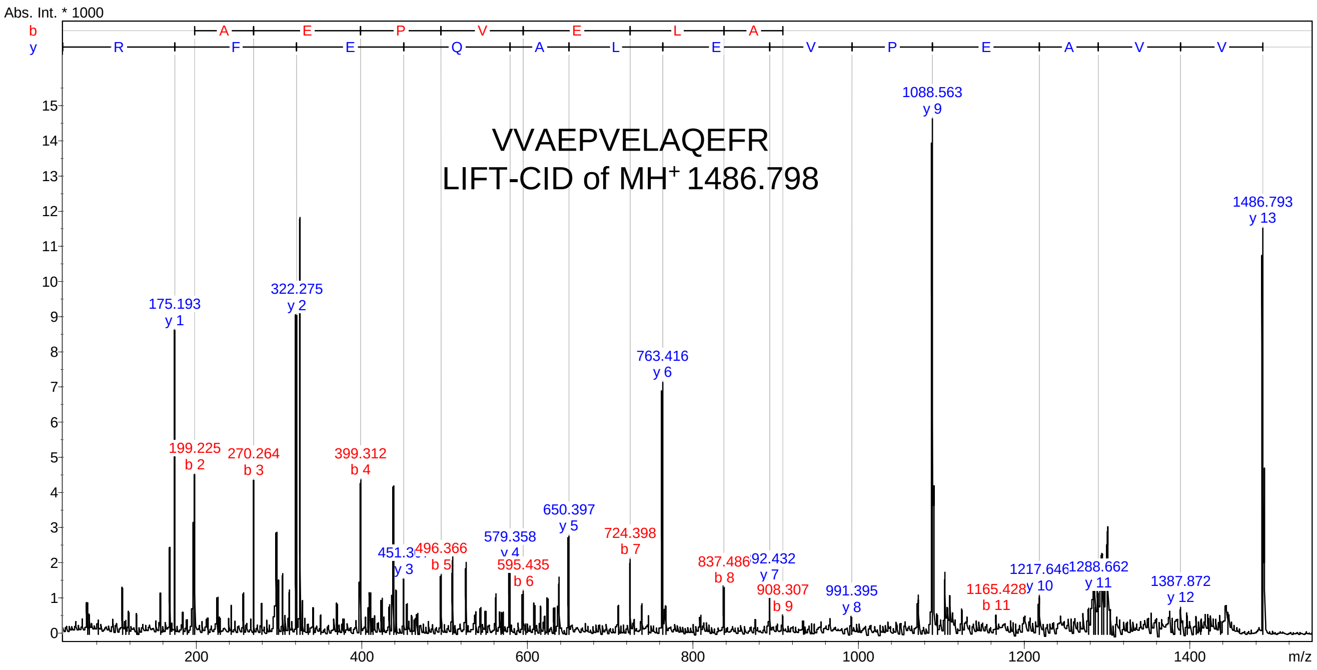

MSMS sequence analysis from the fragmentation of a precursor ion m/z 1486.798 by MALDI-ToF/ToF mass spectrometer

SPOT 51

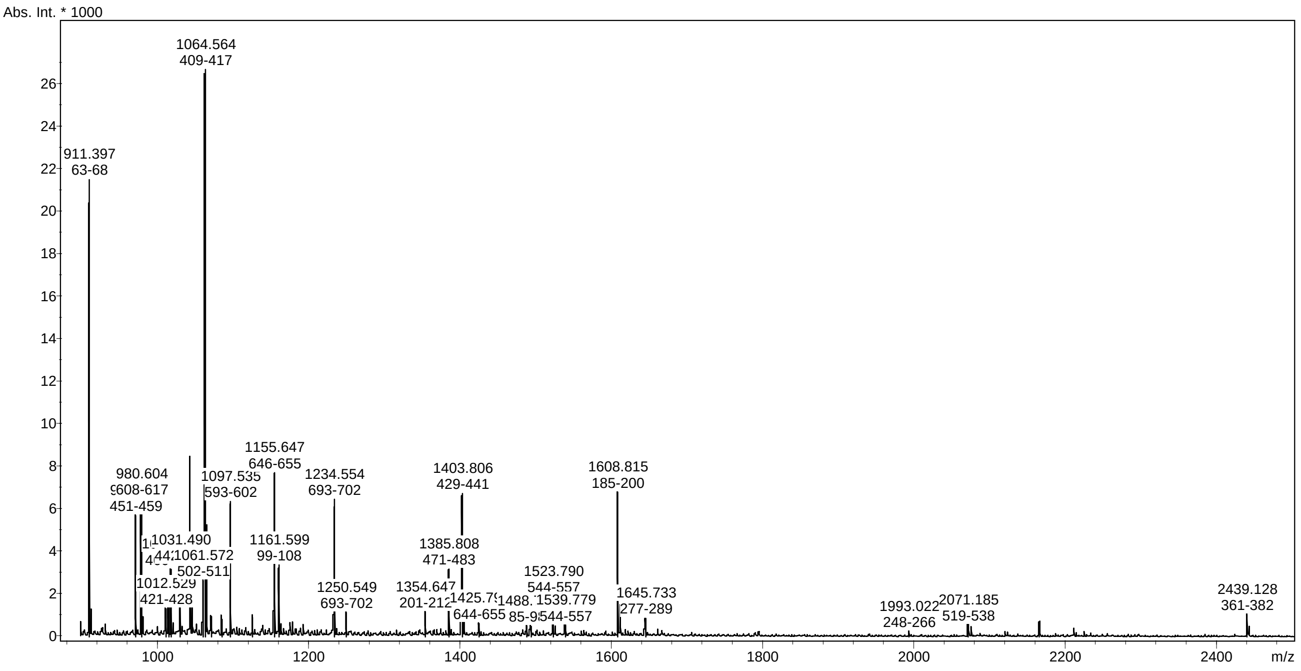

A representative MALDI-ToF PMF spectrum of spot 51

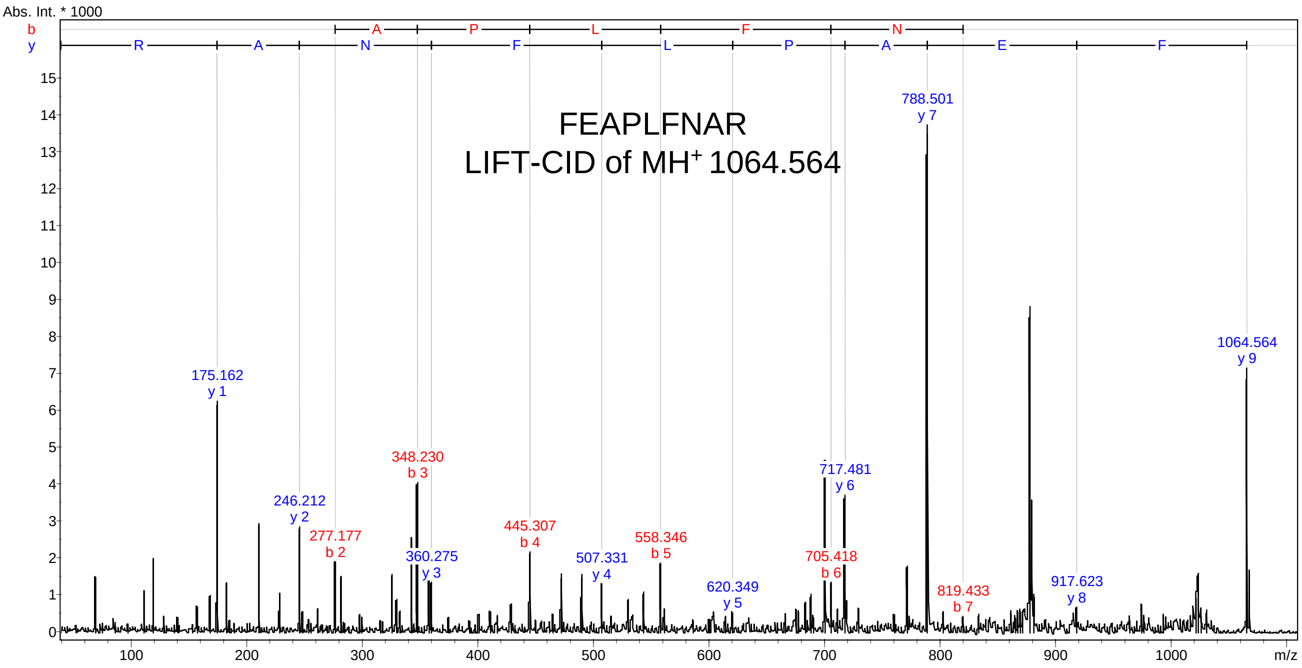

MSMS sequence analysis from the fragmentation of a precursor ion m/z 1064.564 by MALDI-ToF/ToF mass spectrometer

SPOT 52

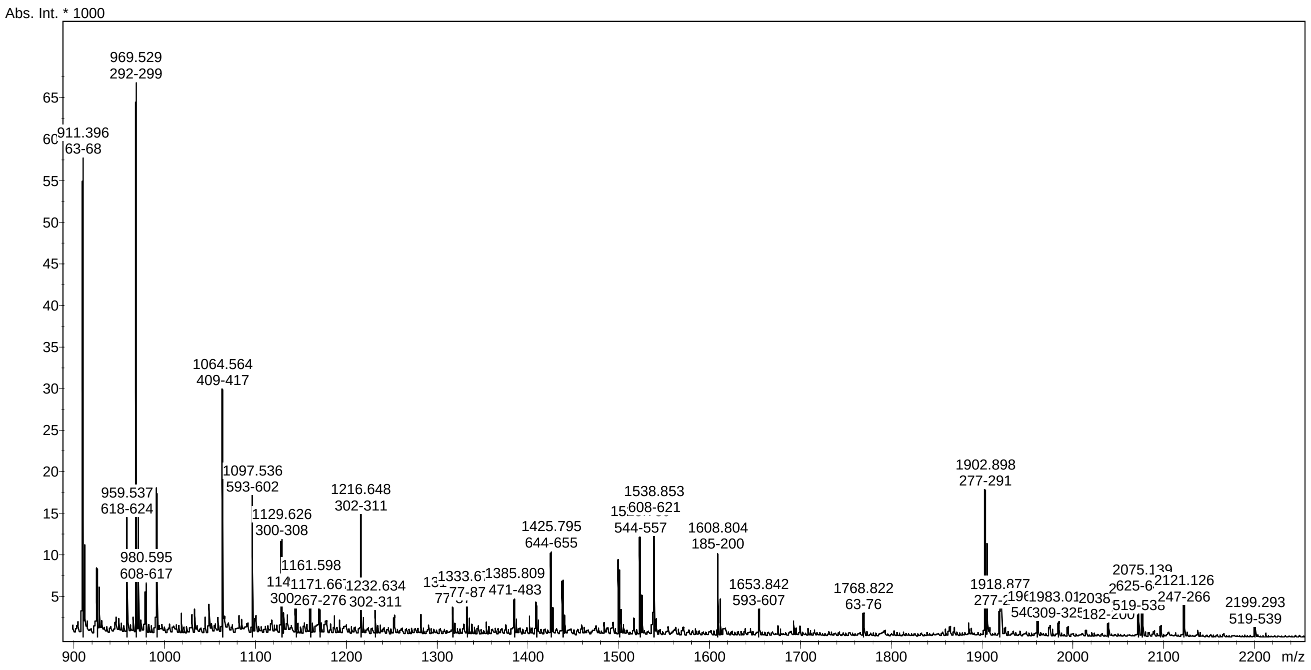

A representative MALDI-ToF PMF spectrum of spot 52

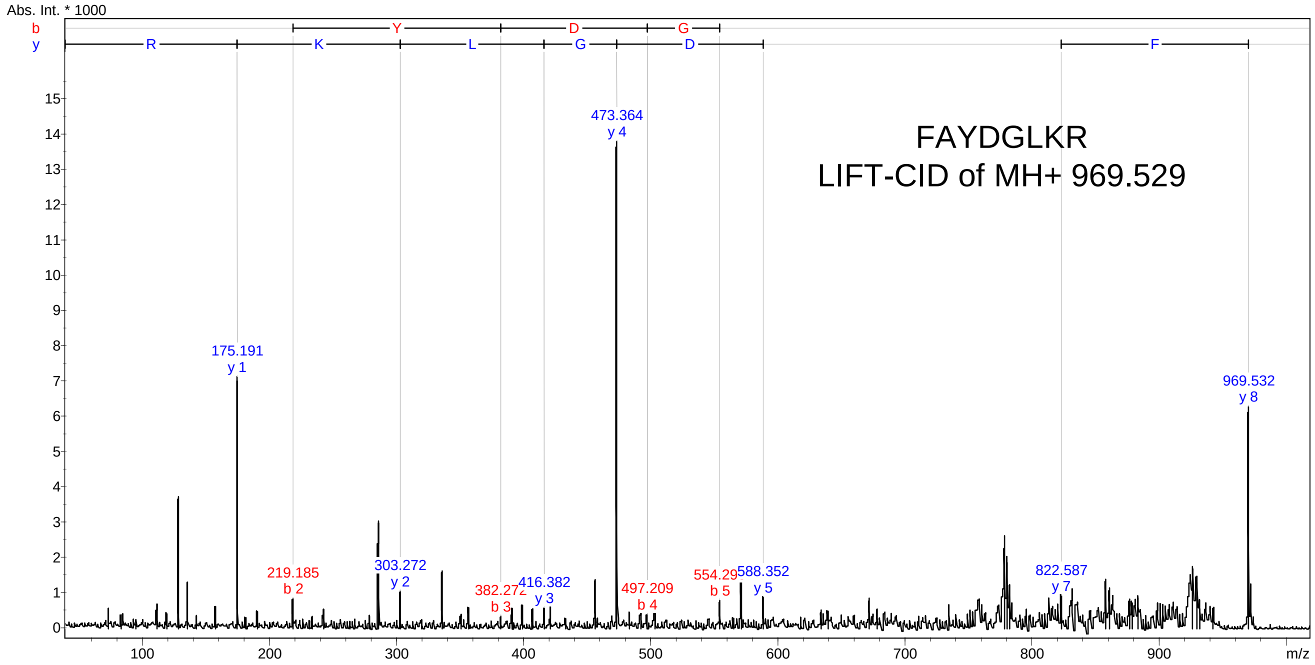

MSMS sequence analysis from the fragmentation of a precursor ion m/z 969.529 by MALDI-ToF/ToF mass spectrometer

SPOT 54

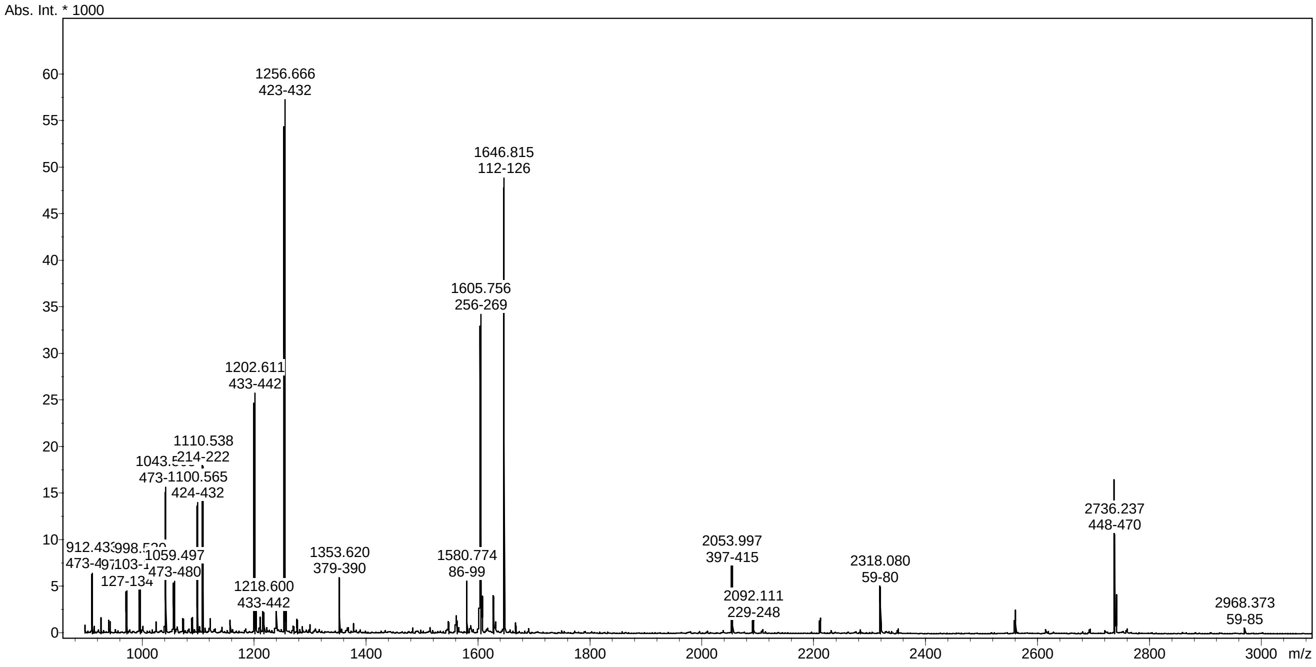

A representative MALDI-ToF PMF spectrum of spot 54

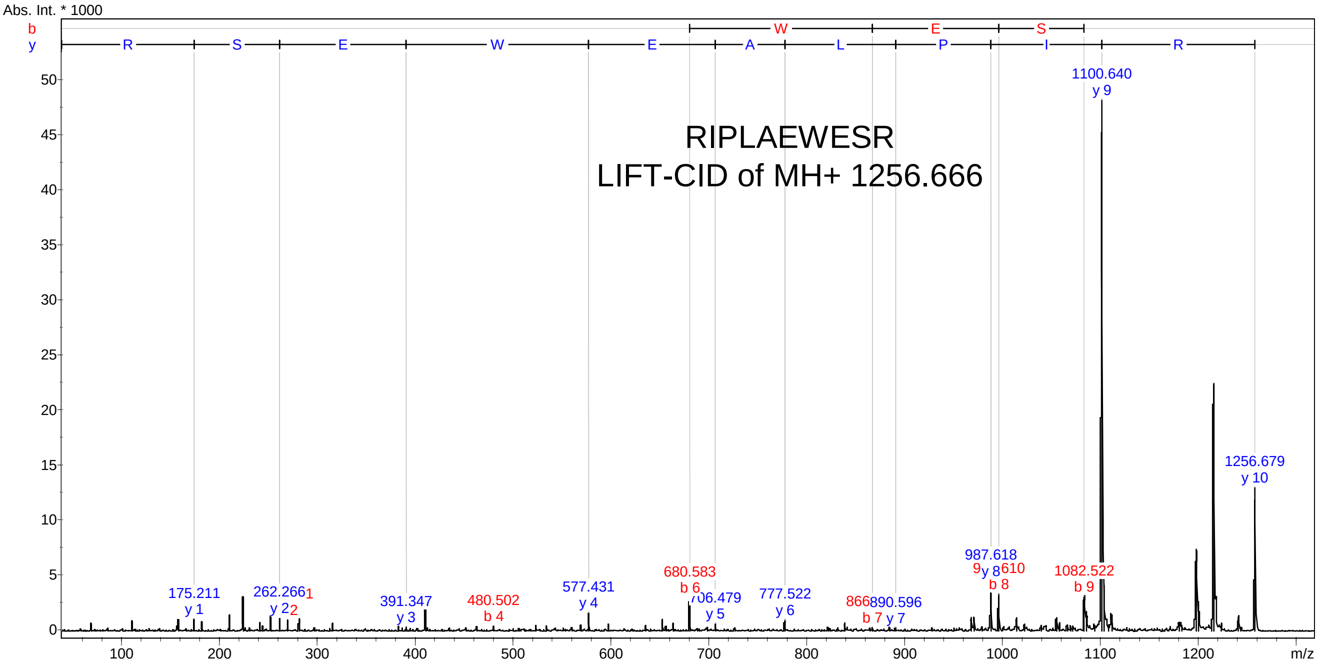

MSMS sequence analysis from the fragmentation of a precursor ion m/z 1256.666 by MALDI-ToF/ToF mass spectrometer

SPOT 55

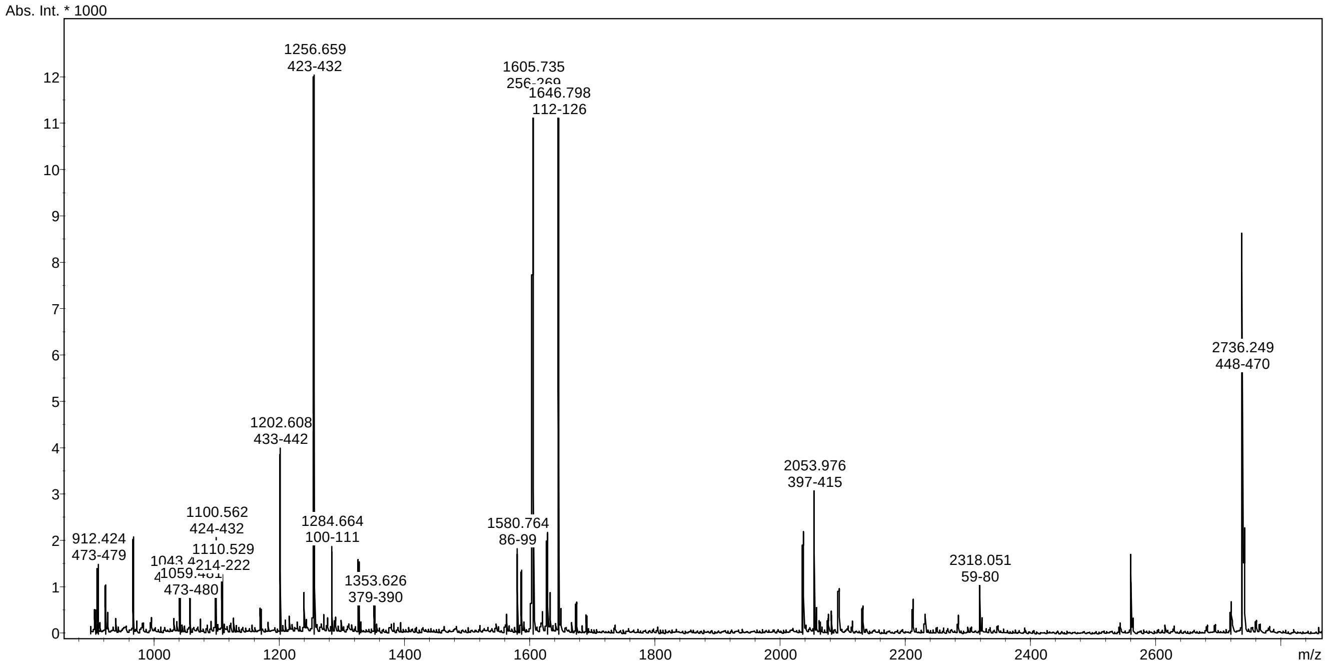

A representative MALDI-ToF PMF spectrum of spot 55

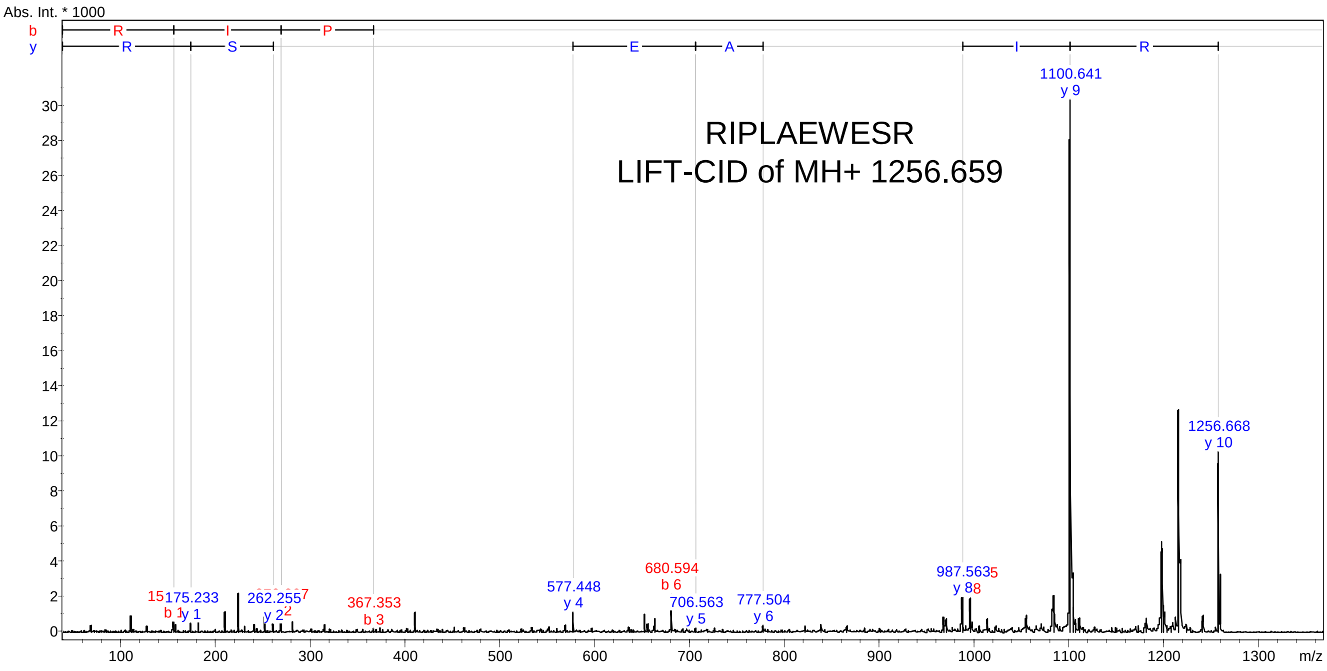

MSMS sequence analysis from the fragmentation of a precursor ion m/z 1713.810 by MALDI-ToF/ToF mass spectrometer

SPOT 56

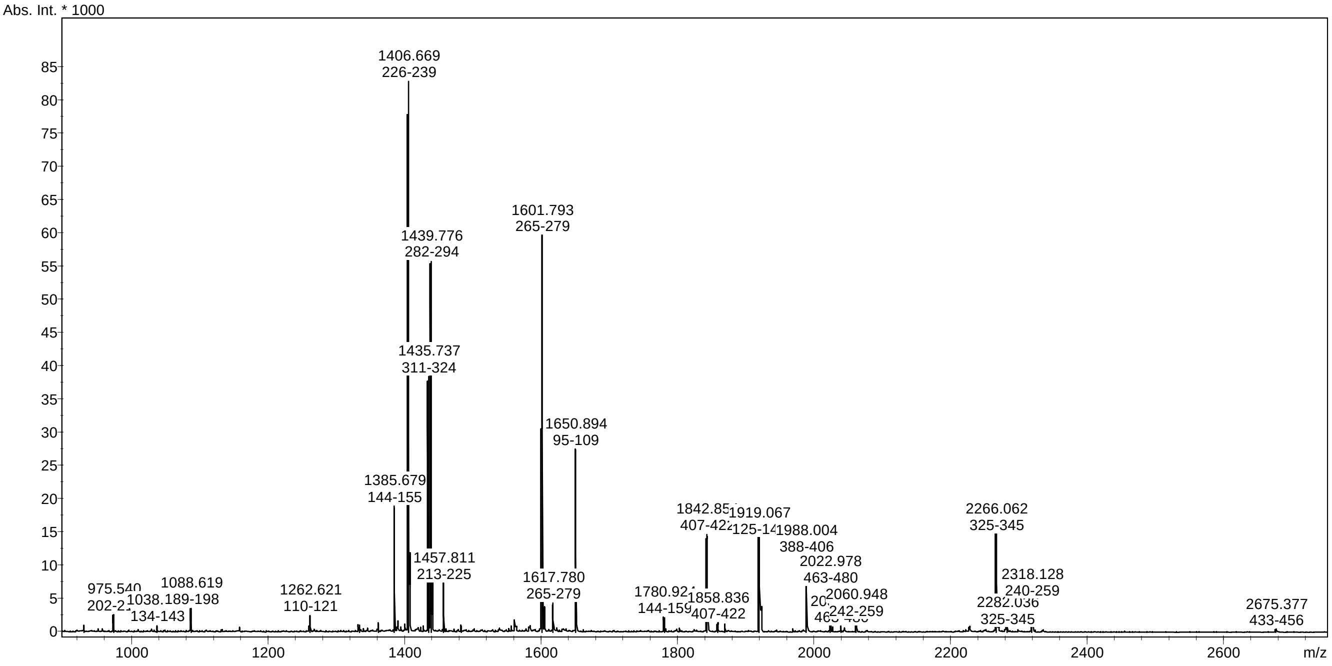

A representative MALDI-ToF PMF spectrum of spot 56

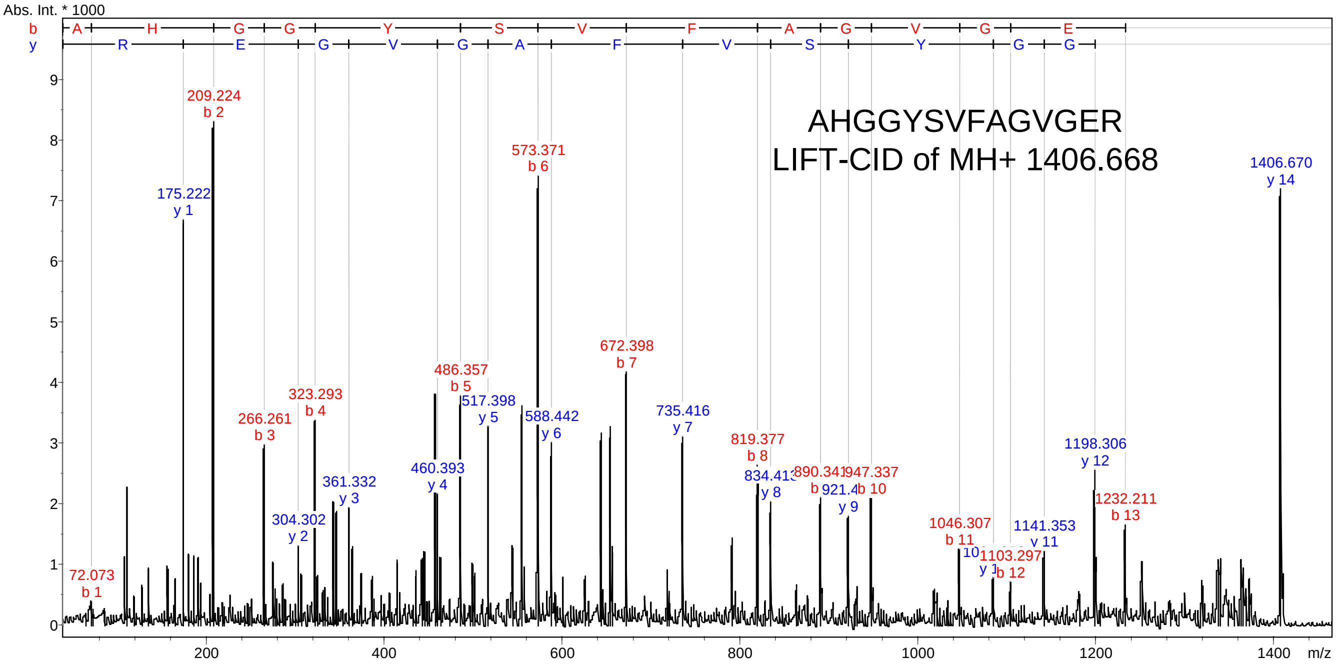

MSMS sequence analysis from the fragmentation of a precursor ion m/z 1406.668 by MALDI-ToF/ToF mass spectrometer

SPOT 57

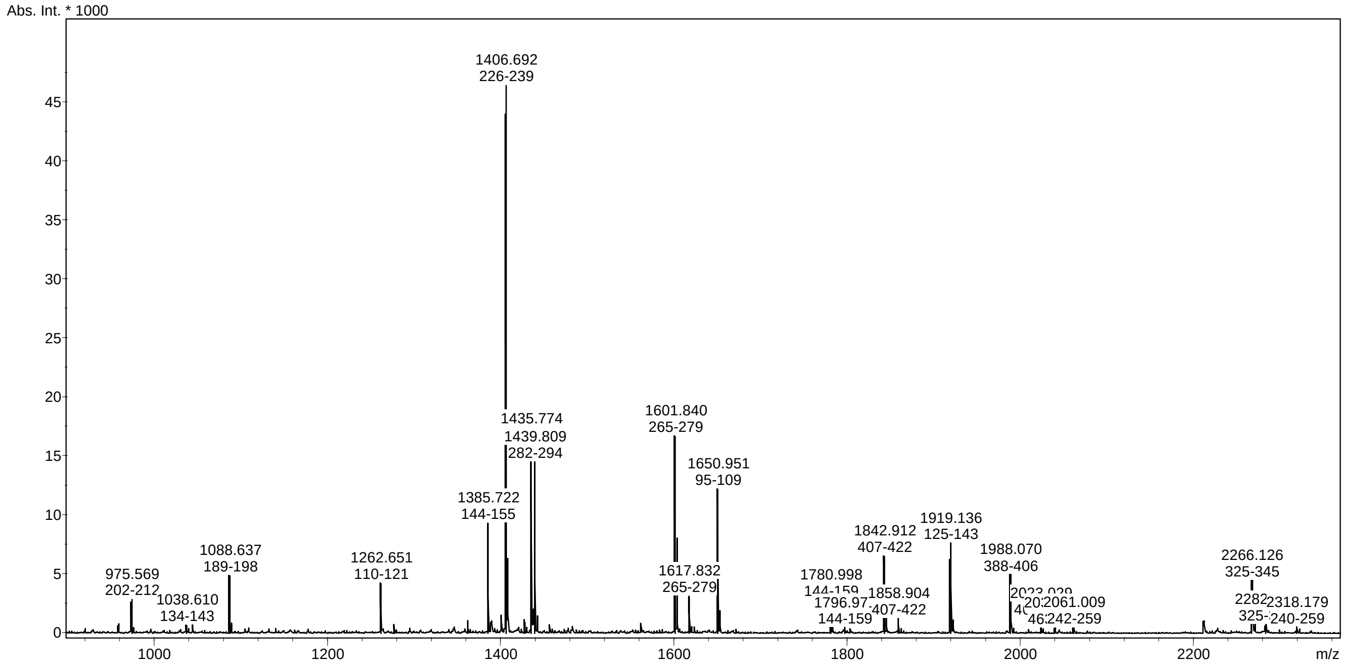

A representative MALDI-ToF PMF spectrum of spot 57

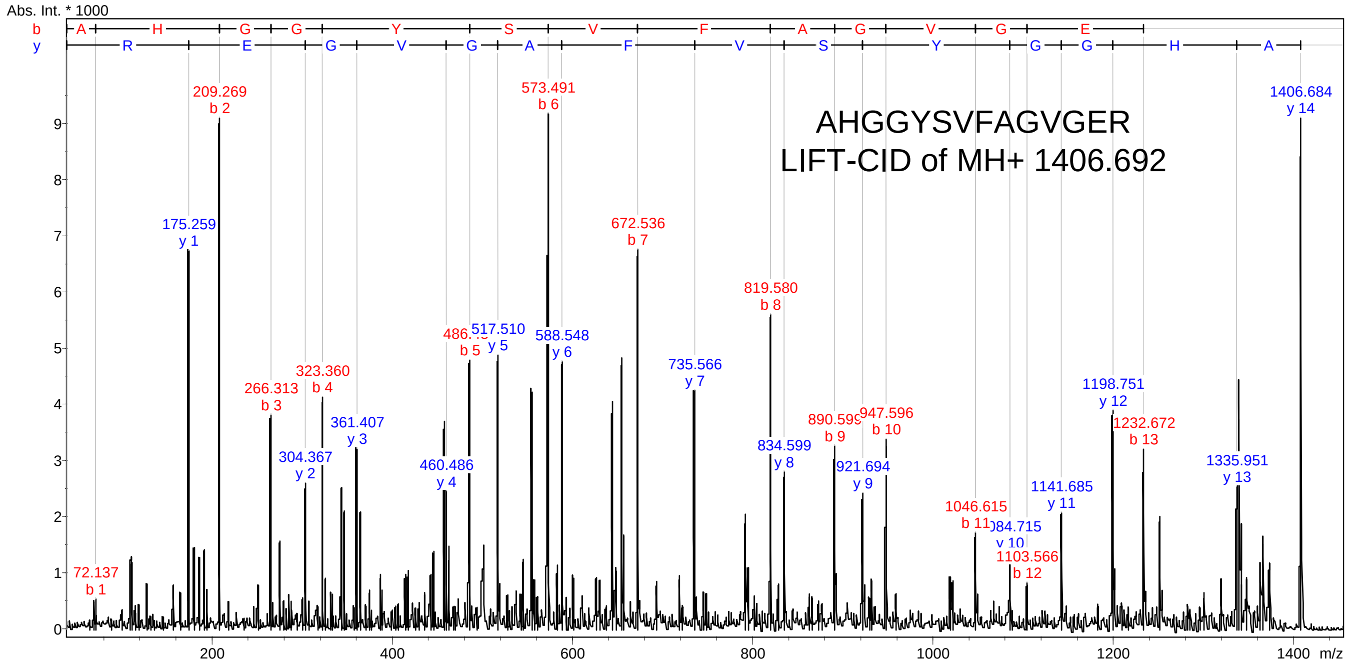

MSMS sequence analysis from the fragmentation of a precursor ion m/z 1406.692 by MALDI-ToF/ToF mass spectrometer

SPOT 58

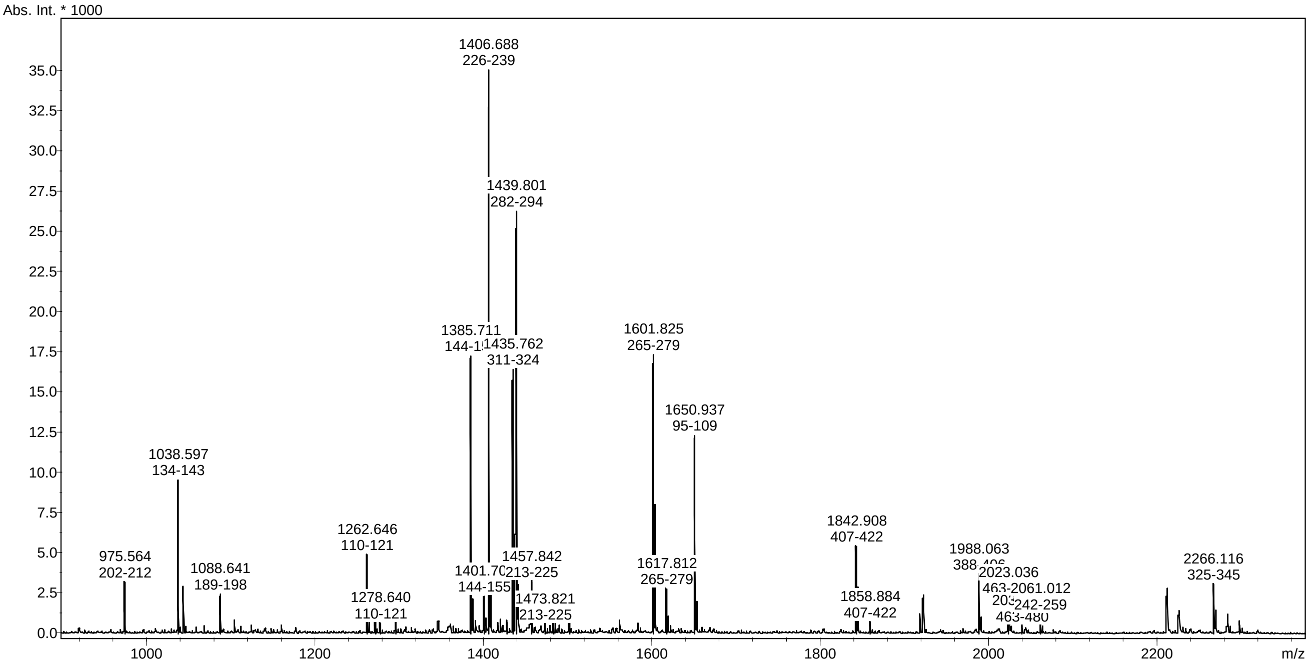

A representative MALDI-ToF PMF spectrum of spot 58

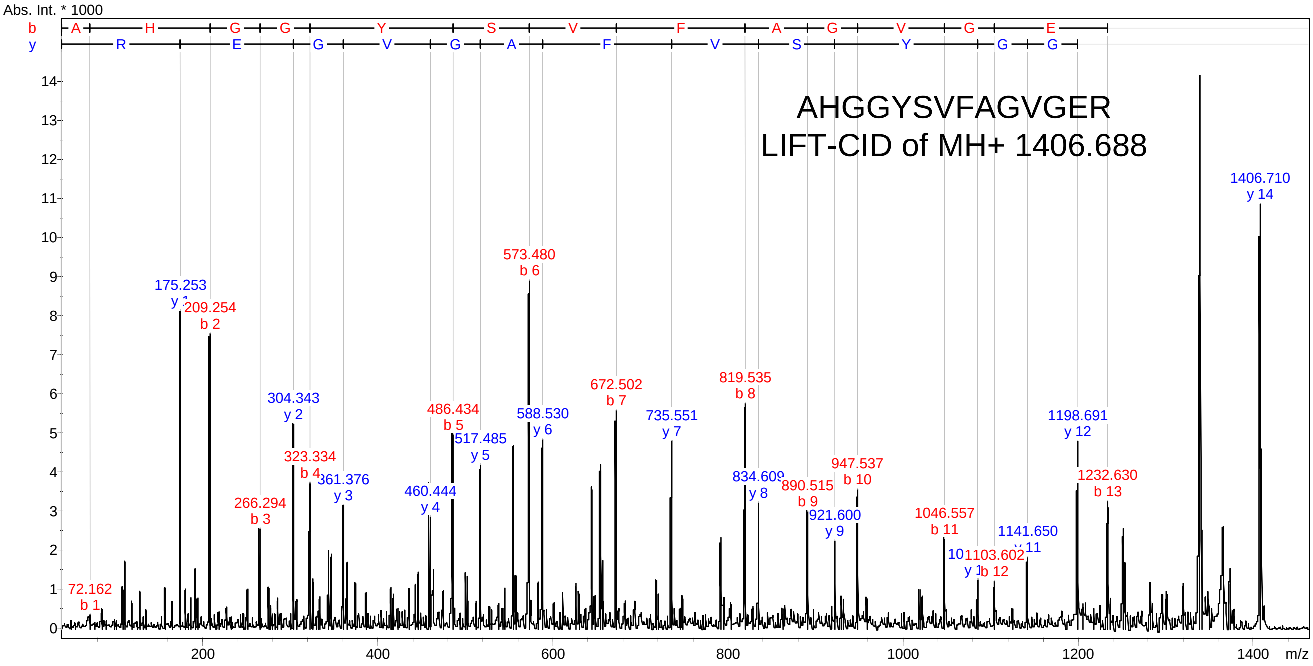

MSMS sequence analysis from the fragmentation of a precursor ion m/z 1406.688 by MALDI-ToF/ToF mass spectrometer

SPOT 59

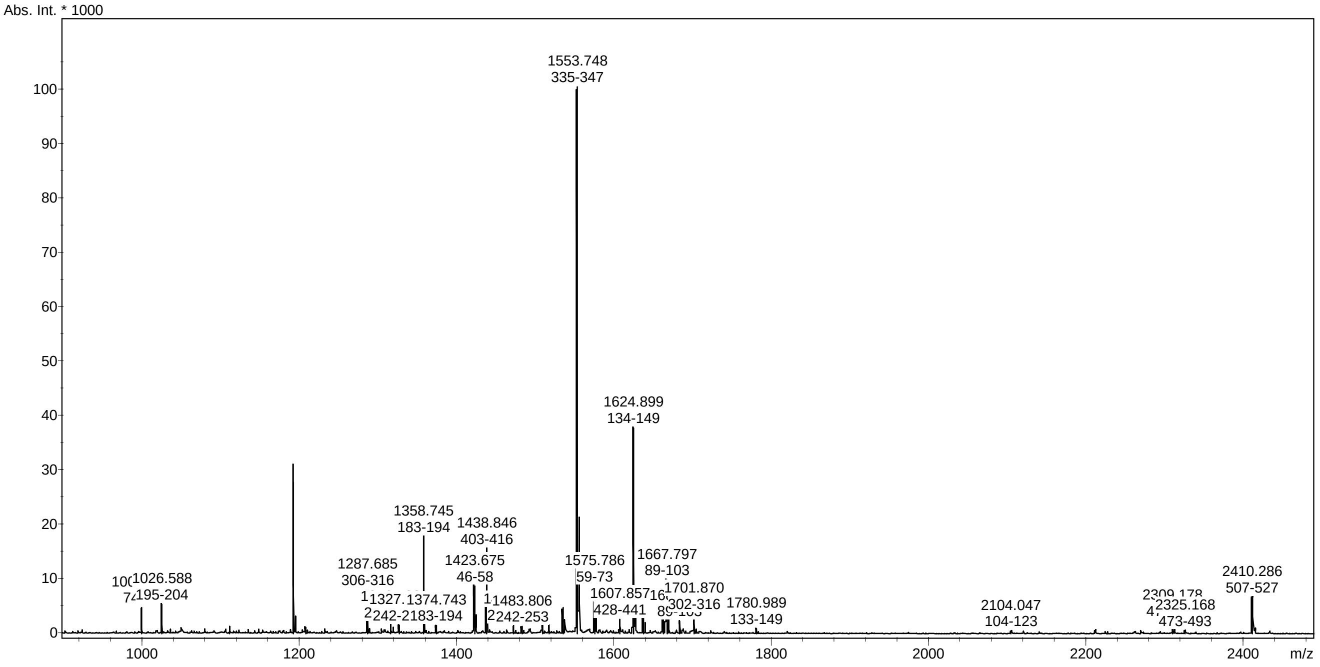

A representative MALDI-ToF PMF spectrum of spot 59

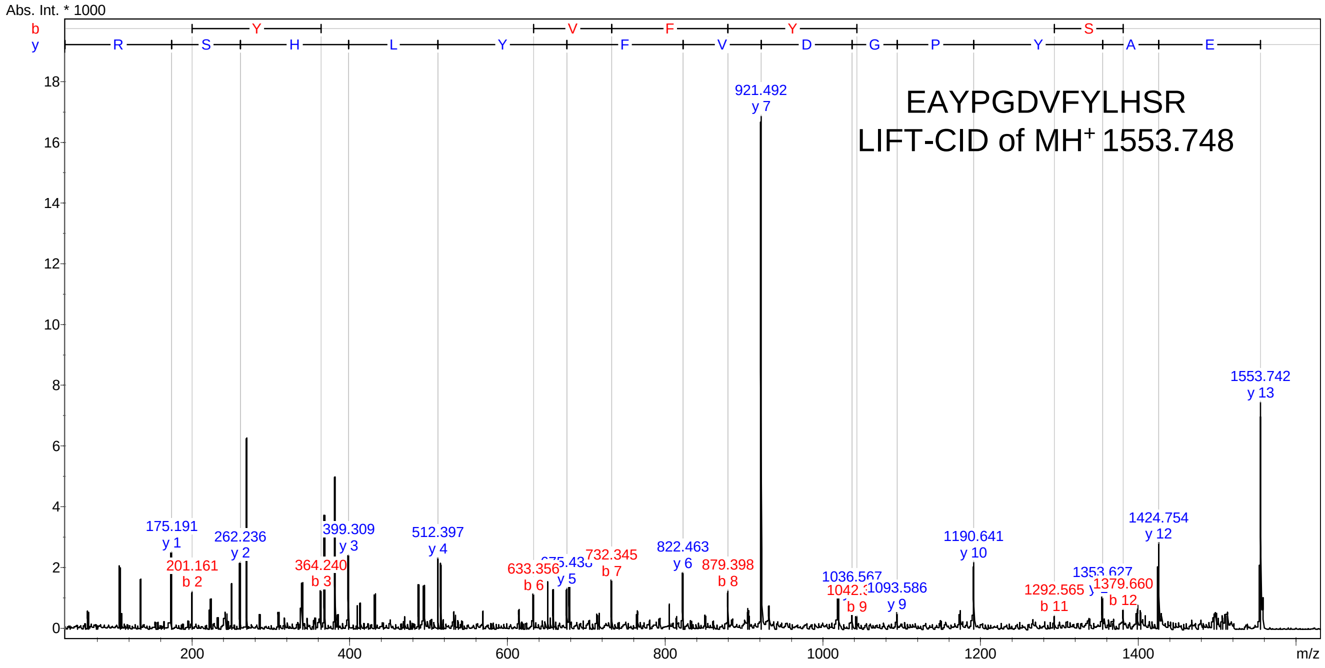

MSMS sequence analysis from the fragmentation of a precursor ion m/z 1553.748 by MALDI-ToF/ToF mass spectrometer

SPOT 60

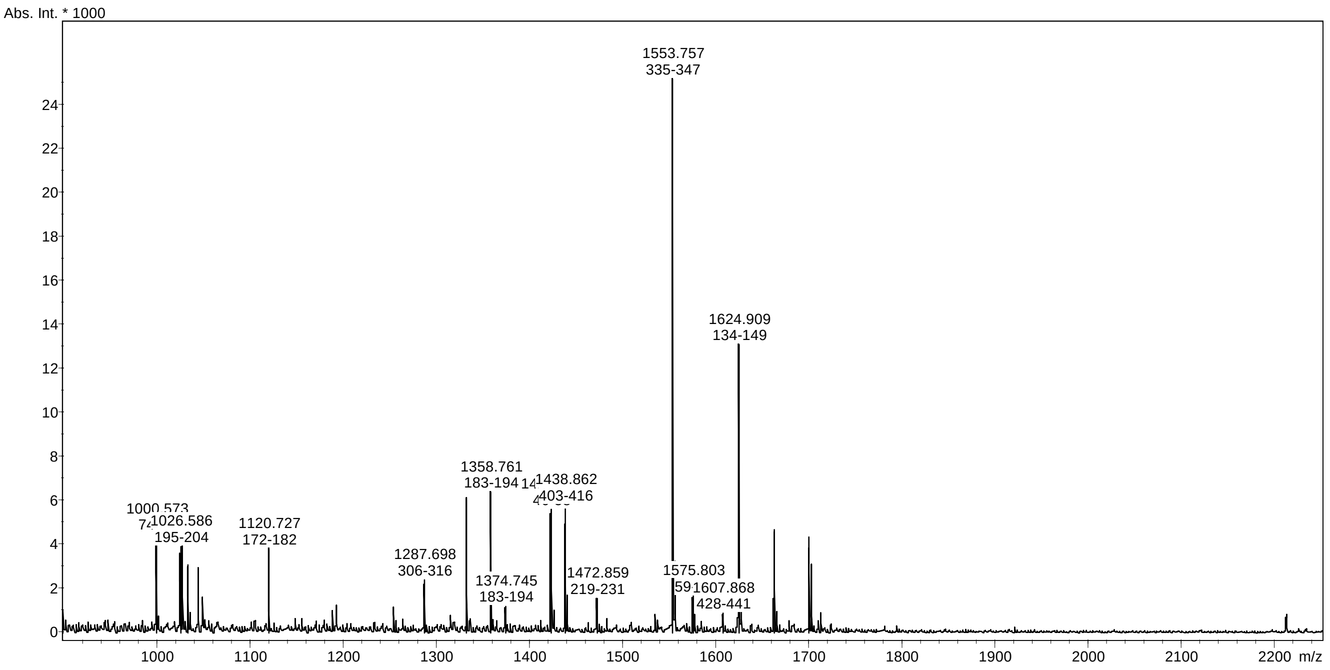

A representative MALDI-ToF PMF spectrum of spot 60

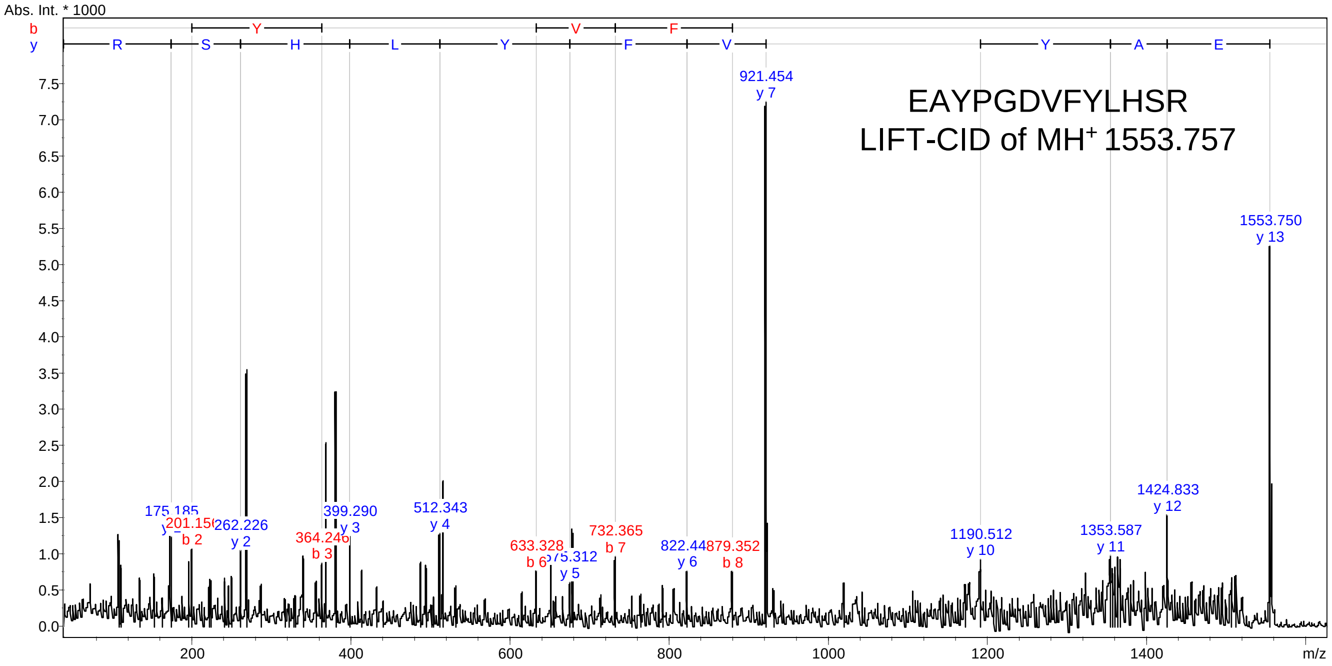

MSMS sequence analysis from the fragmentation of a precursor ion m/z 1553.757 by MALDI-ToF/ToF mass spectrometer

SPOT 61

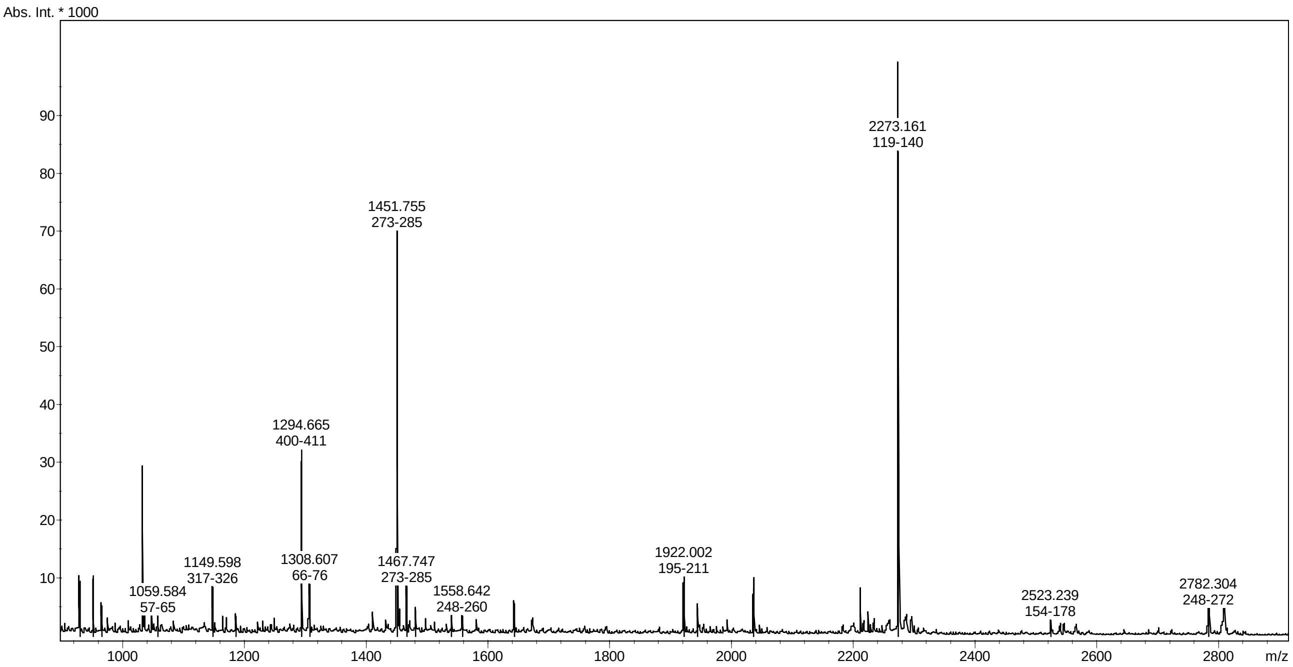

A representative MALDI-ToF PMF spectrum of spot 61

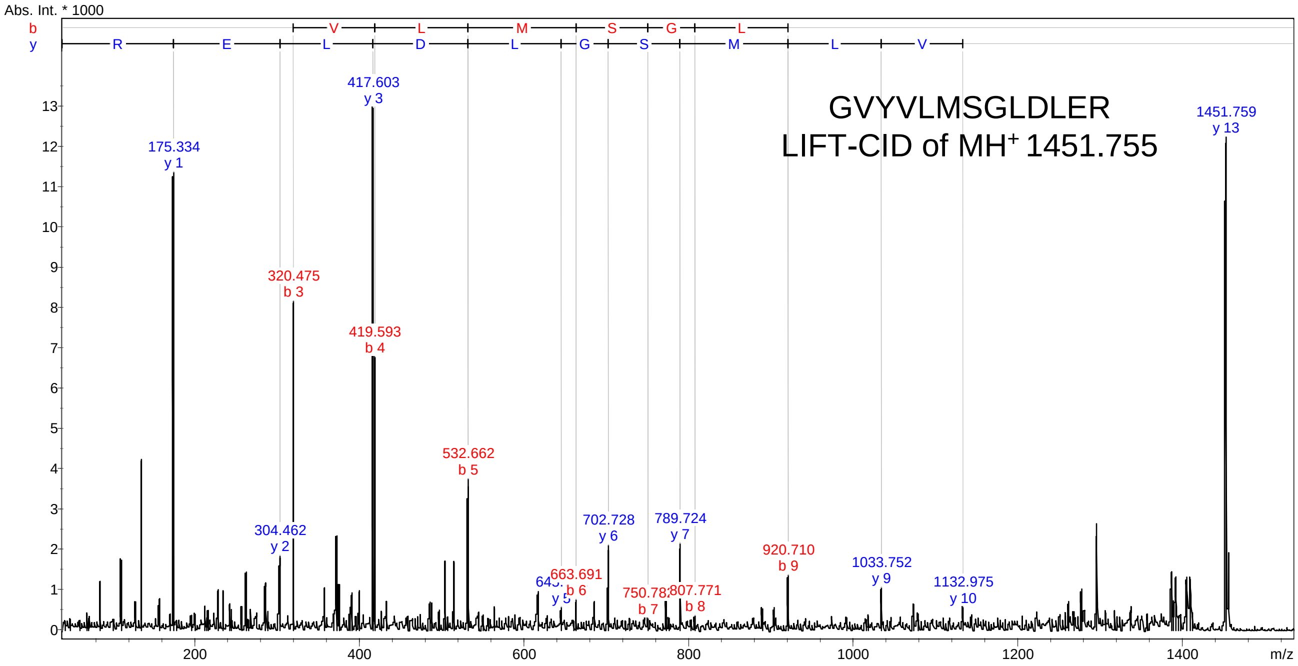

MSMS sequence analysis from the fragmentation of a precursor ion m/z 1451.755 by MALDI-ToF/ToF mass spectrometer

SPOT 62

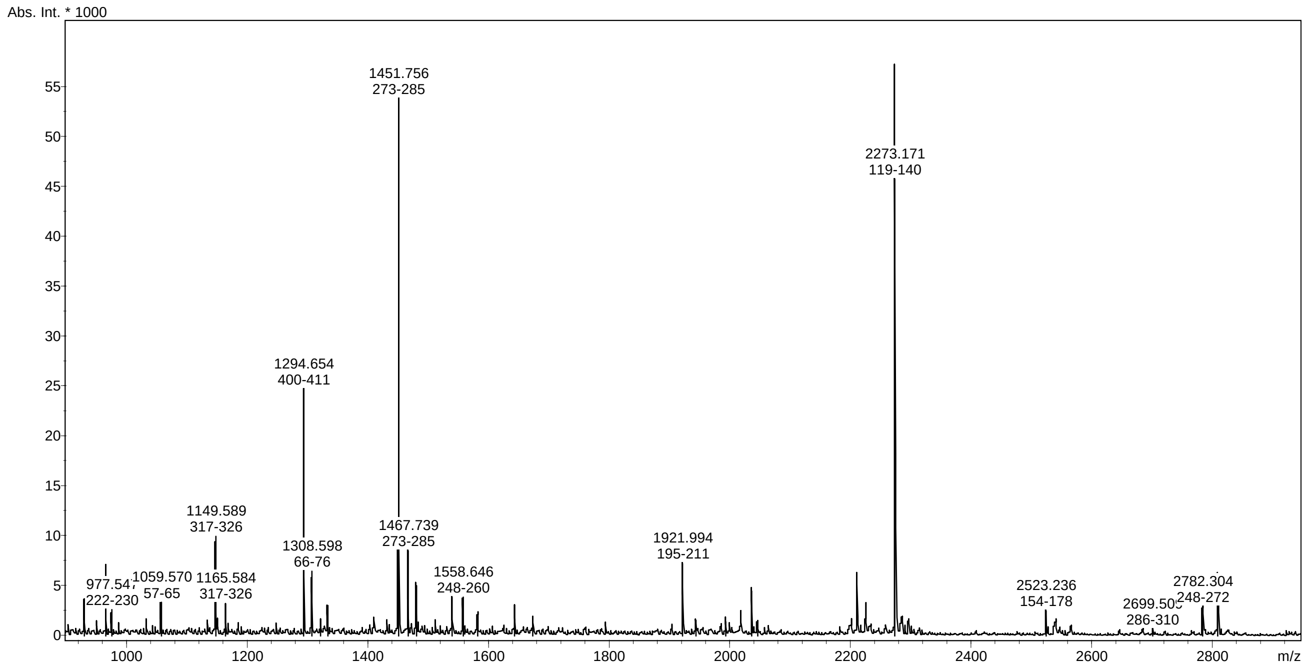

A representative MALDI-ToF PMF spectrum of spot 62

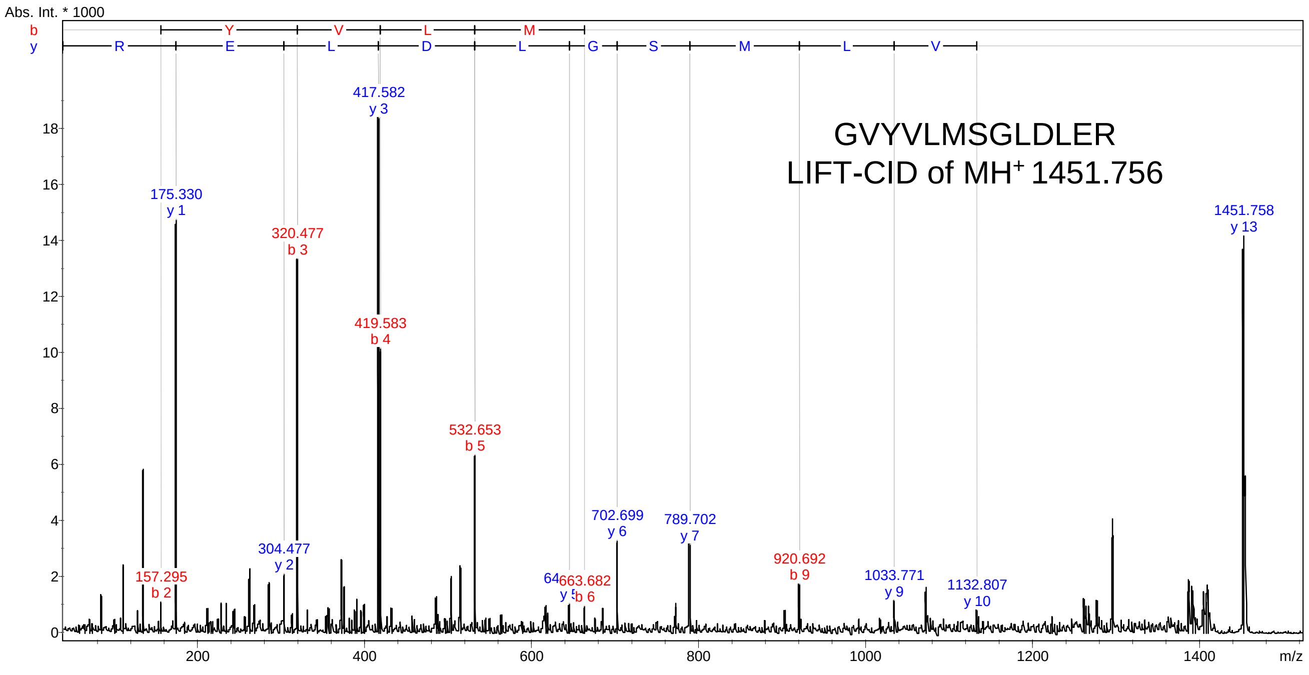

MSMS sequence analysis from the fragmentation of a precursor ion m/z 1451.756 by MALDI-ToF/ToF mass spectrometer

SPOT 63

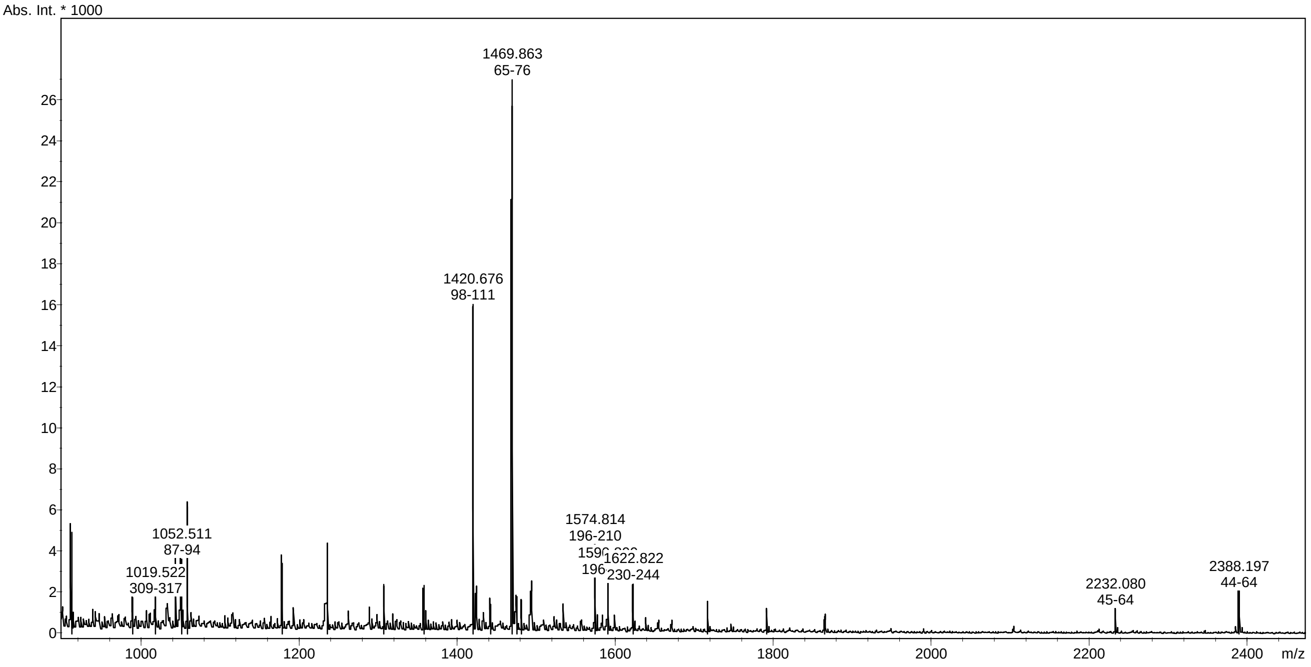

A representative MALDI-ToF PMF spectrum of spot 63

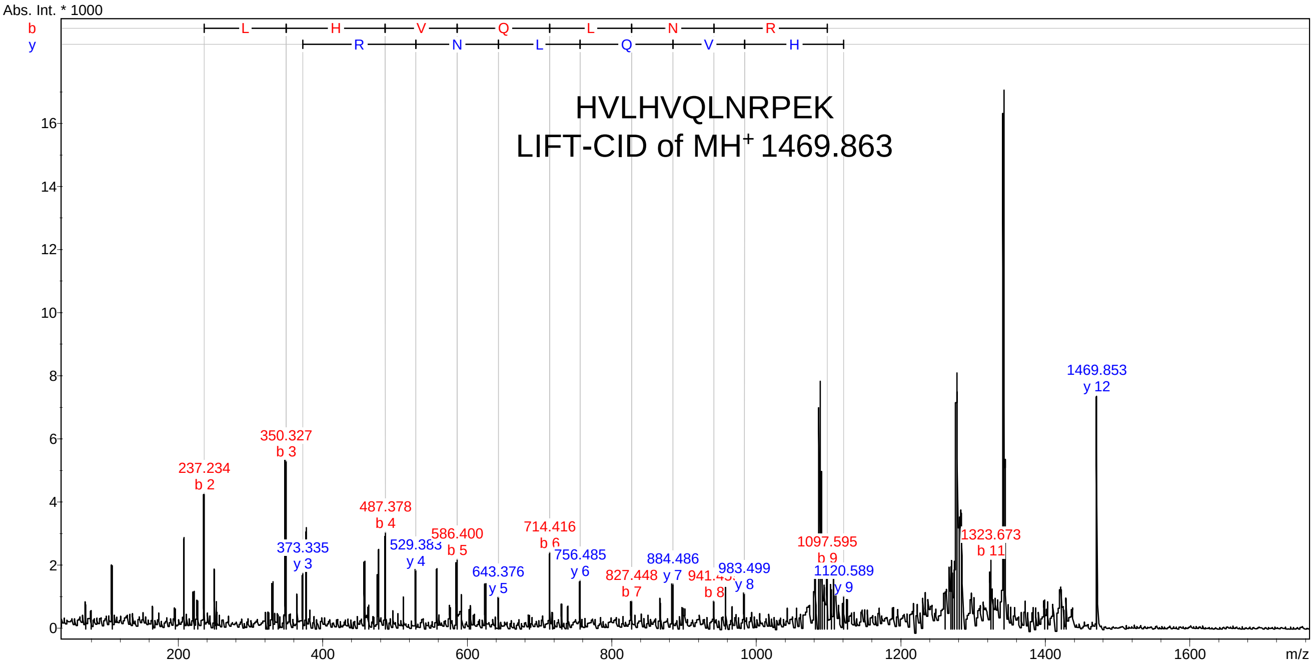

MSMS sequence analysis from the fragmentation of a precursor ion m/z 1469.863 by MALDI-ToF/ToF mass spectrometer

SPOT 65

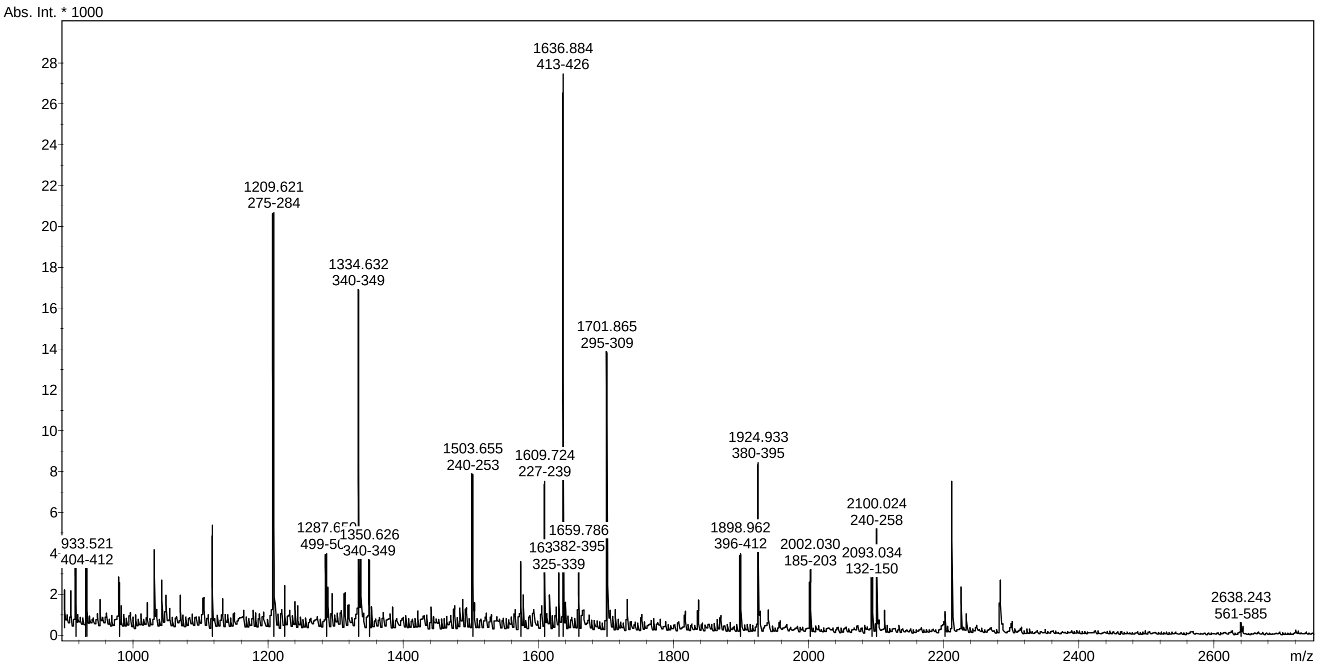

A representative MALDI-ToF PMF spectrum of spot 65

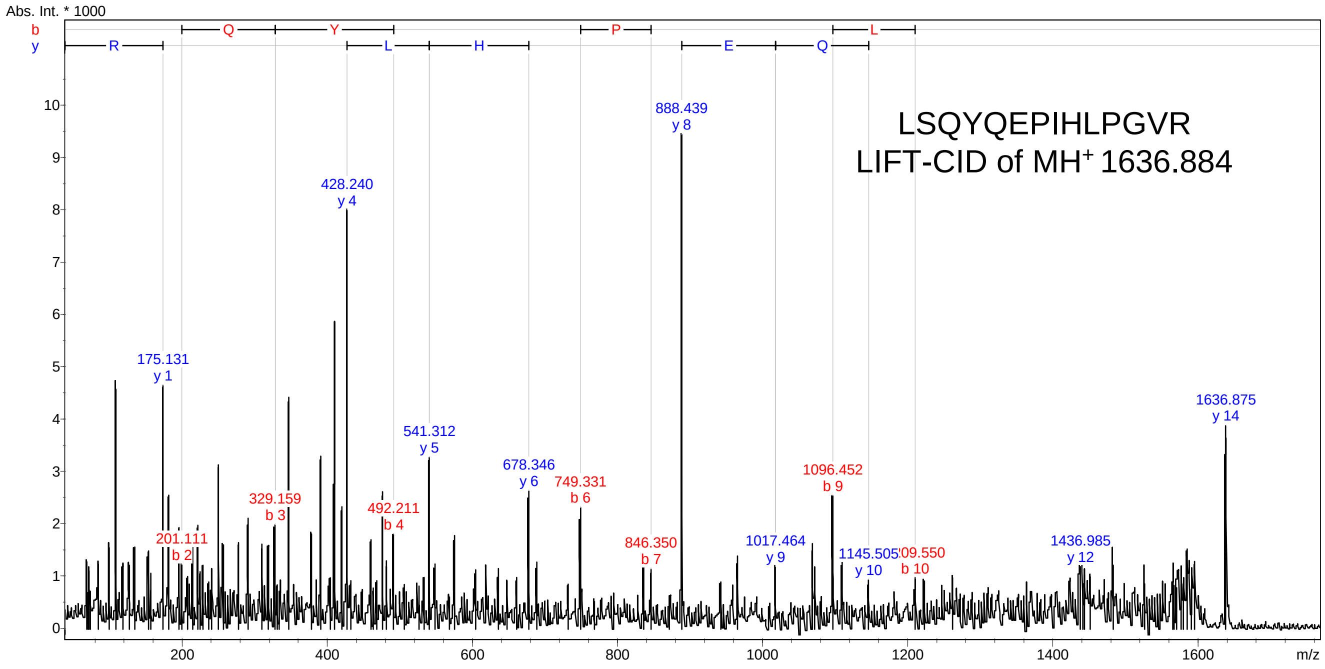

MSMS sequence analysis from the fragmentation of a precursor ion m/z 1636.884 by MALDI-ToF/ToF mass spectrometer

SPOT 66

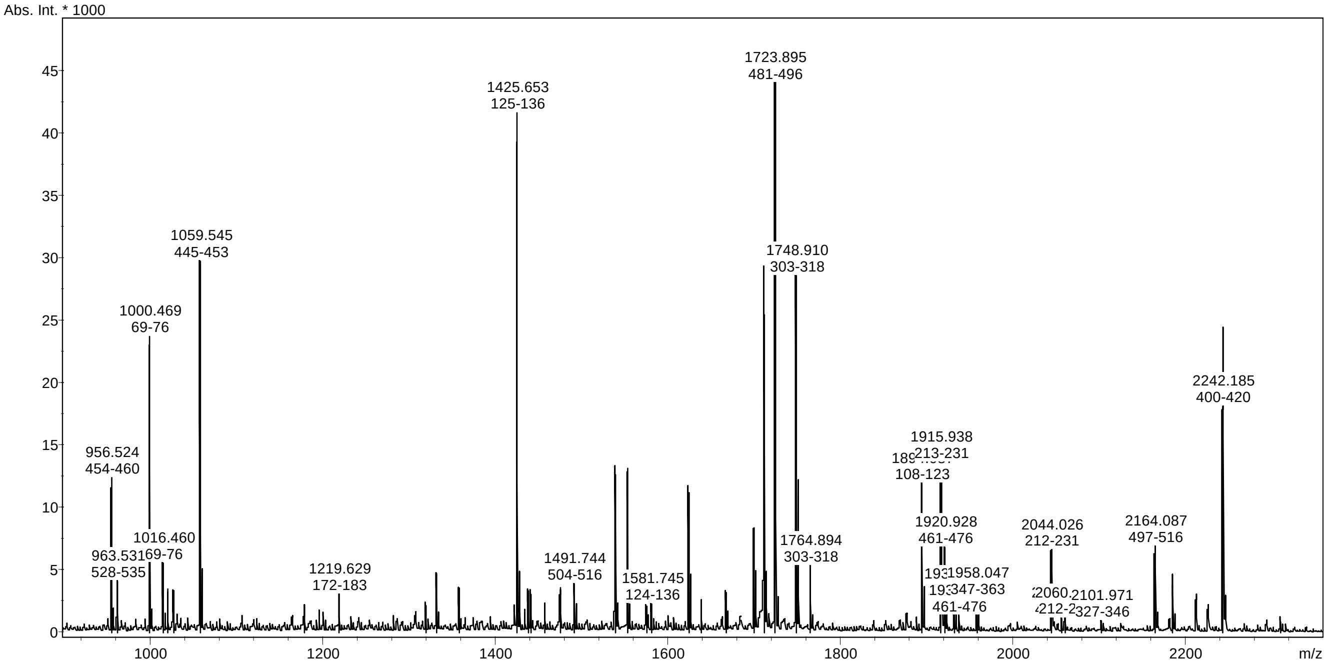

A representative MALDI-ToF PMF spectrum of spot 66

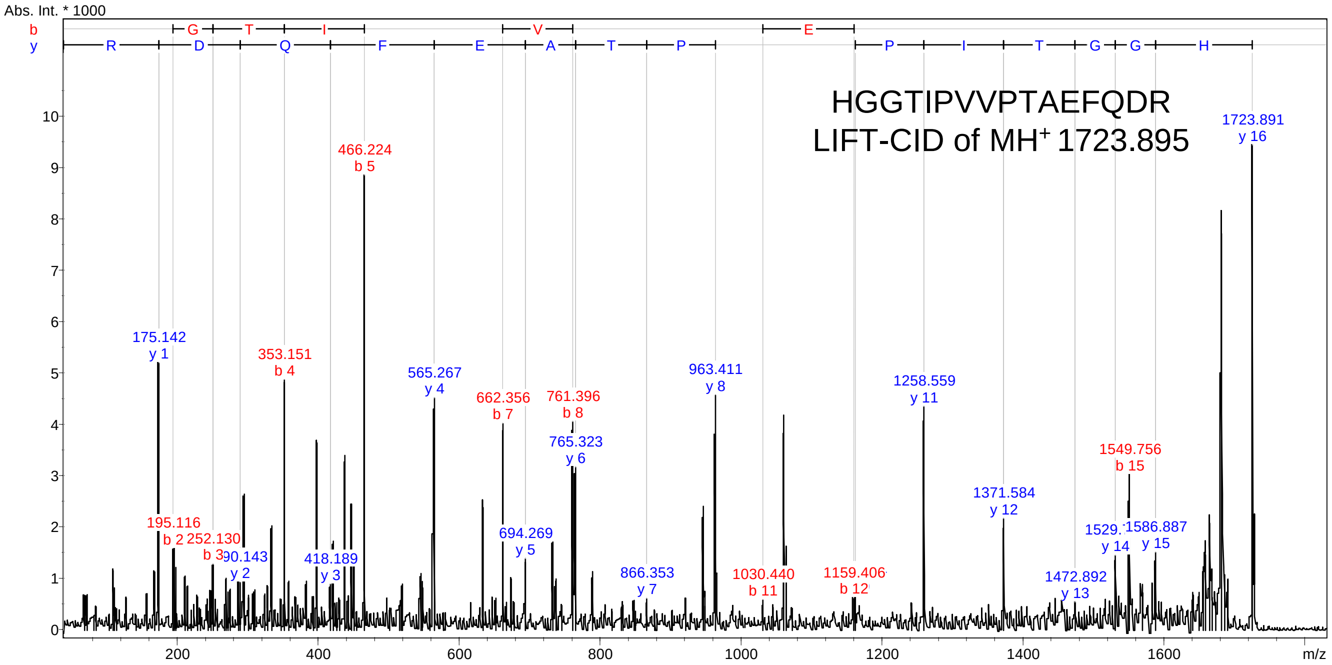

MSMS sequence analysis from the fragmentation of a precursor ion m/z 1723.895 by MALDI-ToF/ToF mass spectrometer

SPOT 67

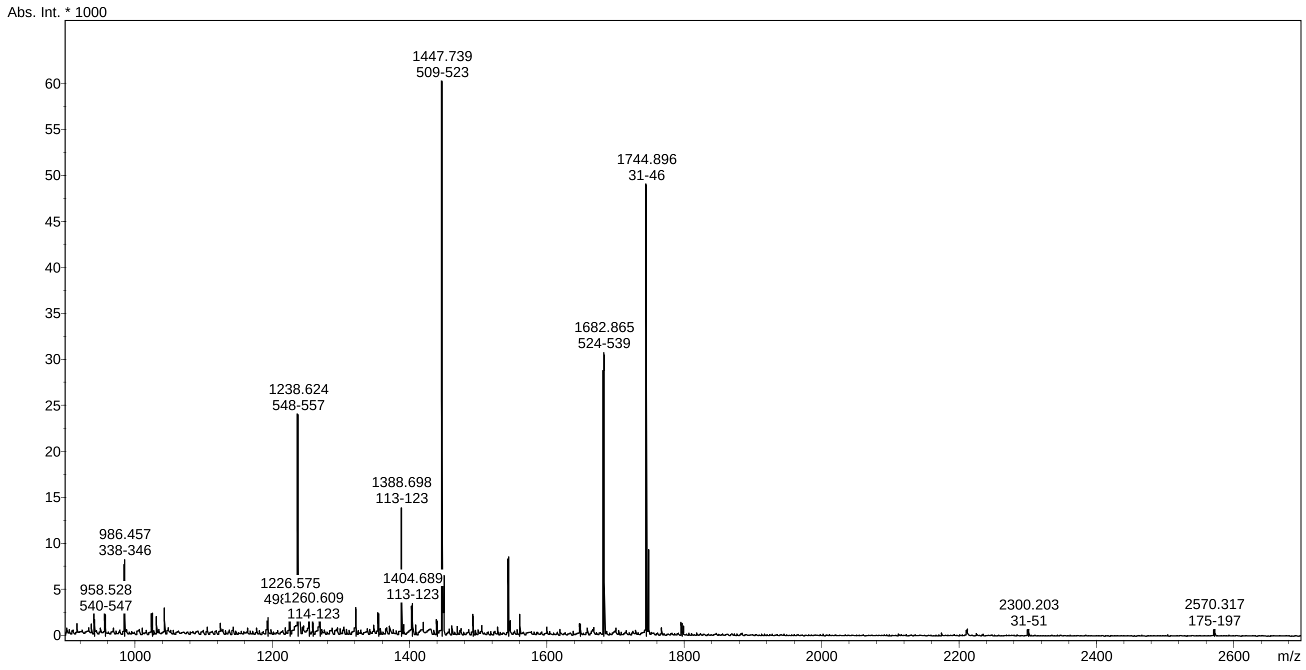

A representative MALDI-ToF PMF spectrum of spot 67

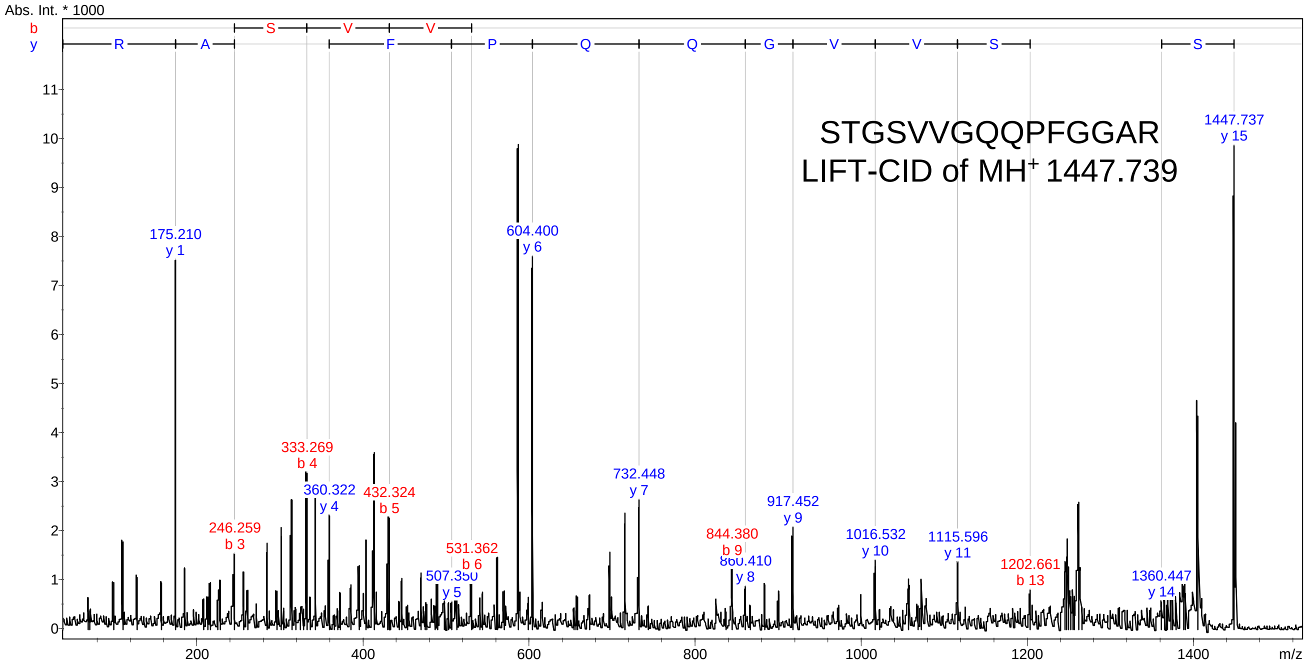

MSMS sequence analysis from the fragmentation of a precursor ion m/z 1447.739 by MALDI-ToF/ToF mass spectrometer

SPOT 68

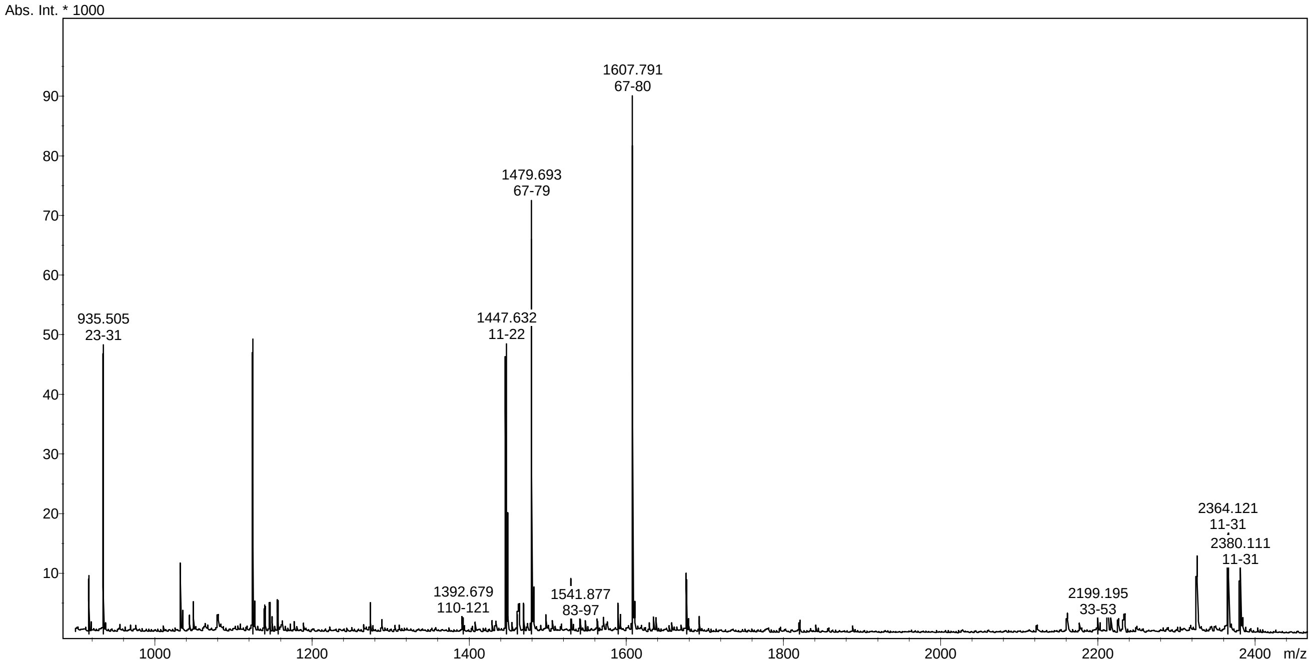

A representative MALDI-ToF PMF spectrum of spot 68

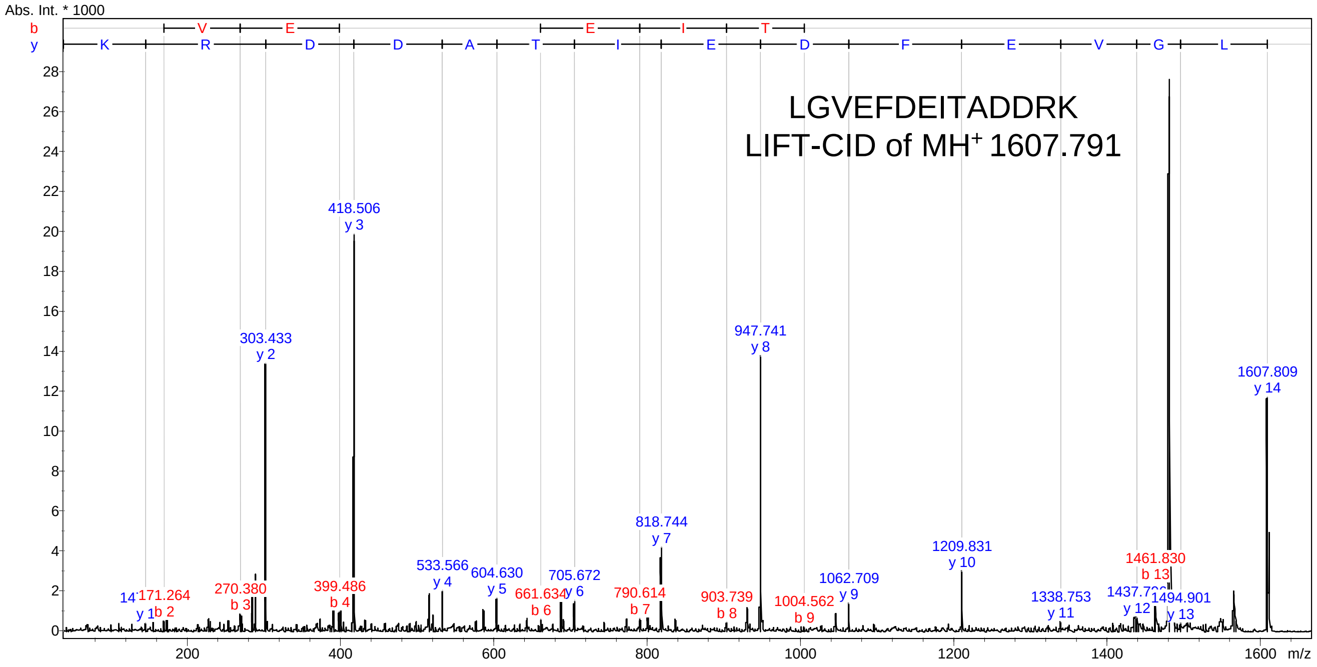

MSMS sequence analysis from the fragmentation of a precursor ion m/z 1607.791 by MALDI-ToF/ToF mass spectrometer

SPOT 69

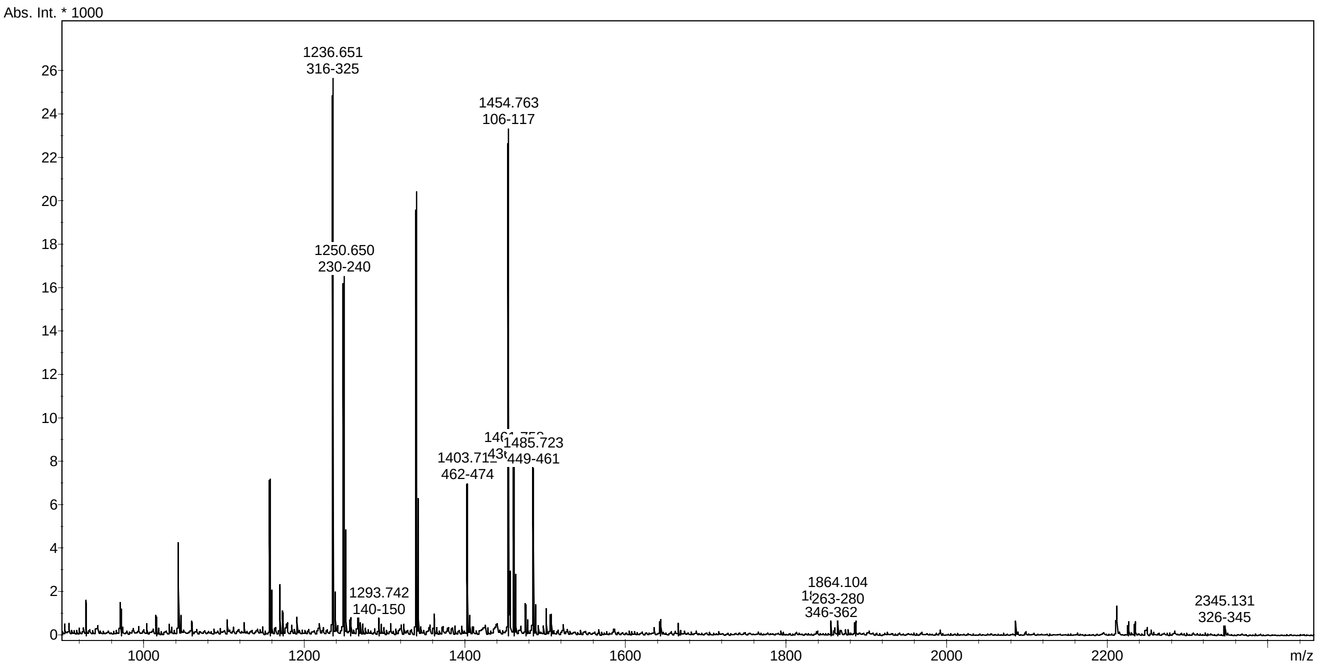

A representative MALDI-ToF PMF spectrum of spot 69

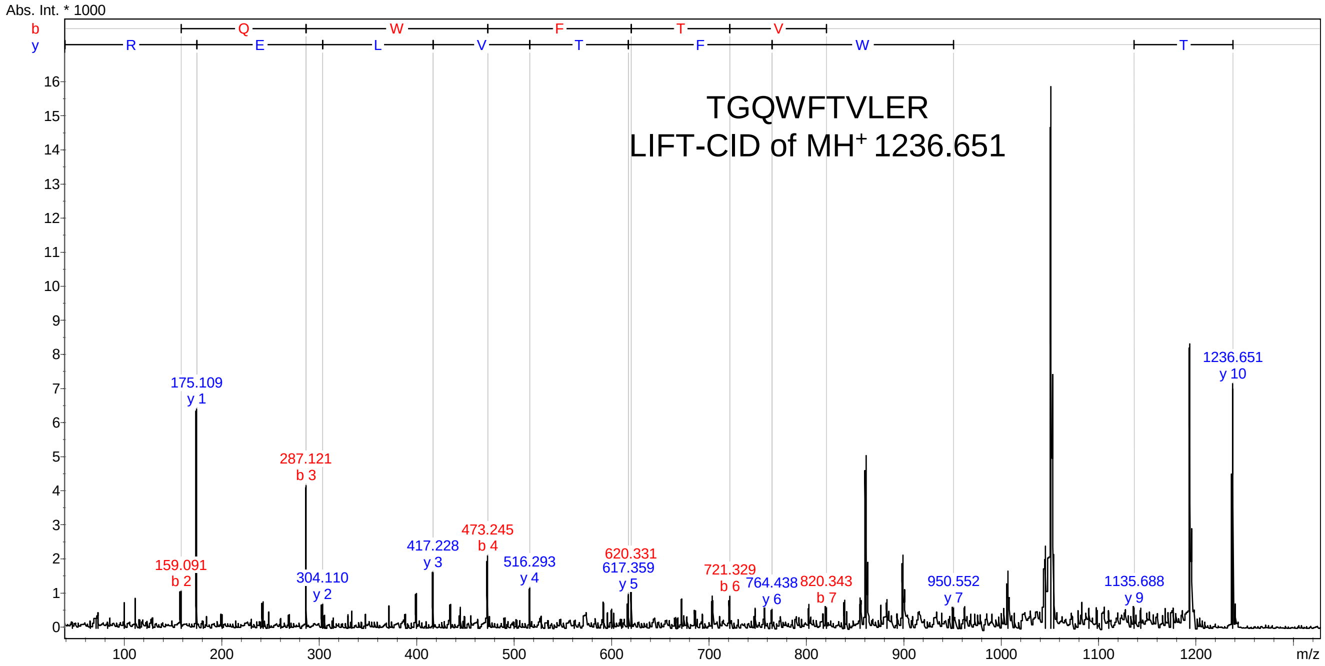

MSMS sequence analysis from the fragmentation of a precursor ion m/z 1236.651 by MALDI-ToF/ToF mass spectrometer

SPOT 70

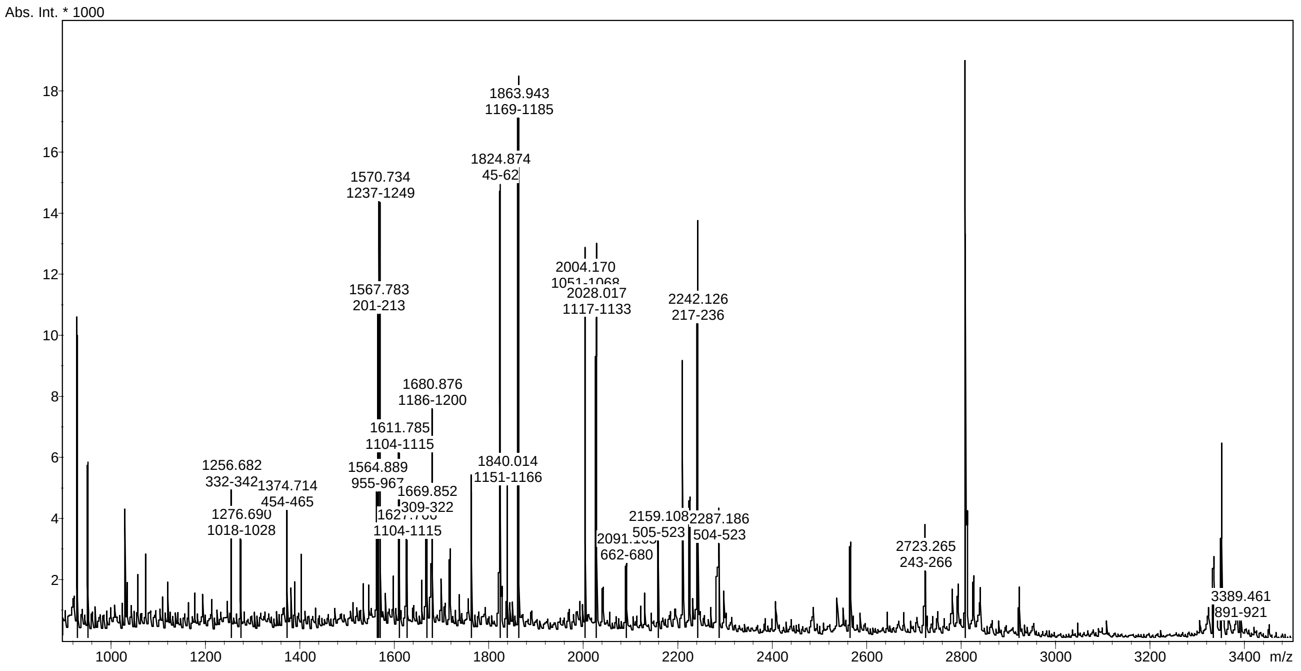

A representative MALDI-ToF PMF spectrum of spot 70

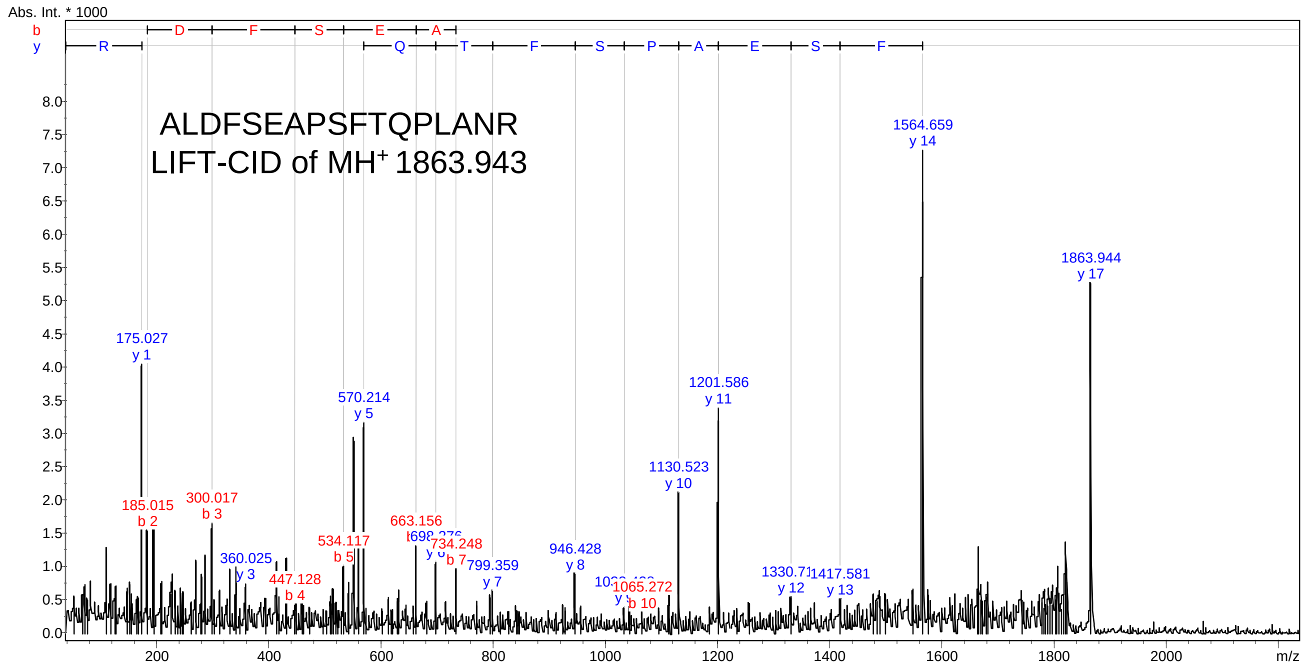

MSMS sequence analysis from the fragmentation of a precursor ion m/z 1863.943 by MALDI-ToF/ToF mass spectrometer

SPOT 71

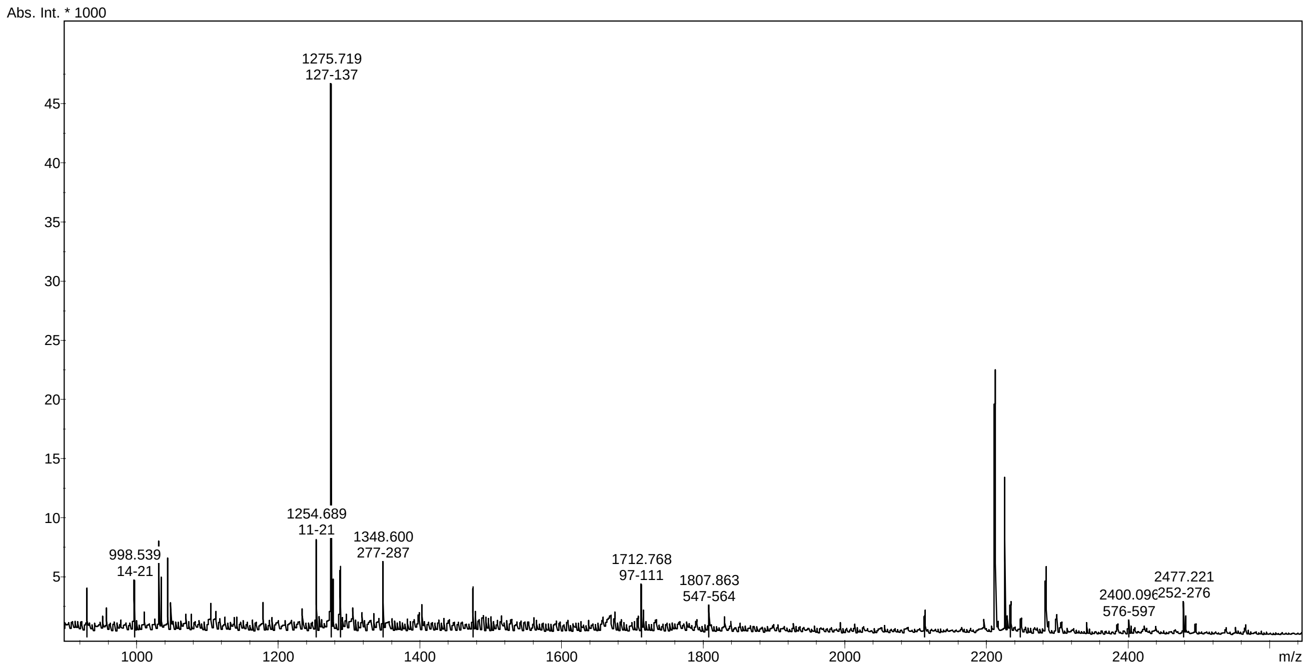

A representative MALDI-ToF PMF spectrum of spot 71

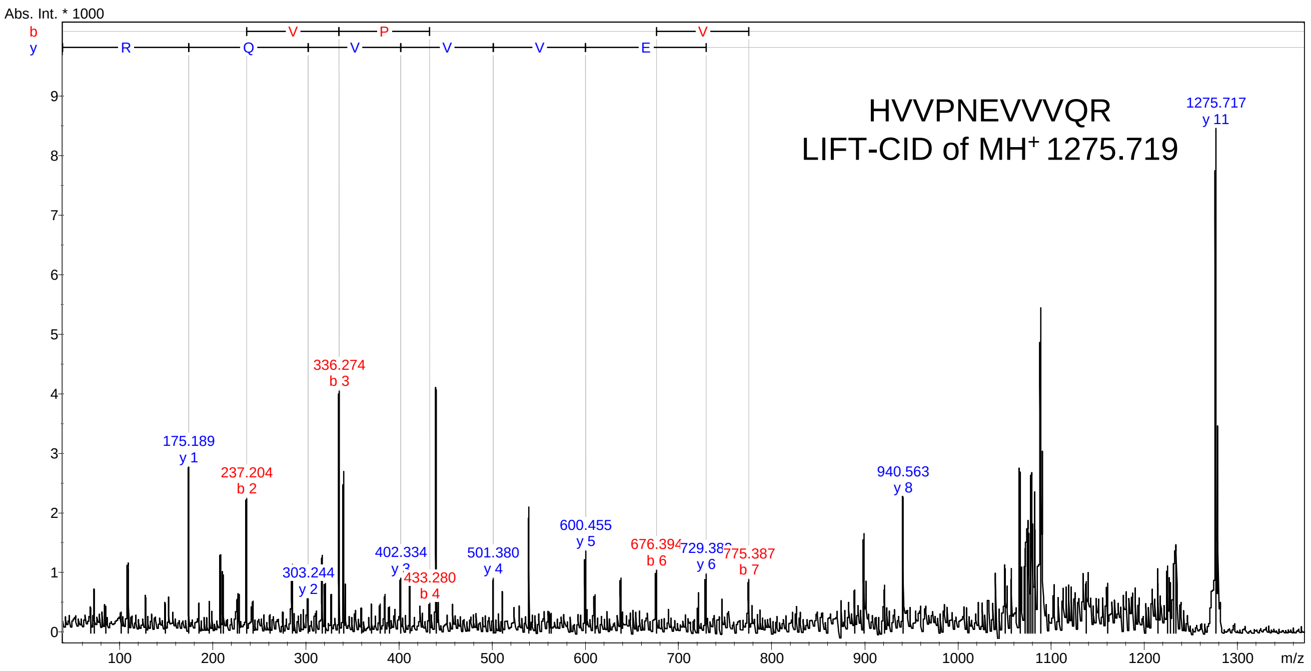

MSMS sequence analysis from the fragmentation of a precursor ion m/z 1275.719 by MALDI-ToF/ToF mass spectrometer

SPOT 72

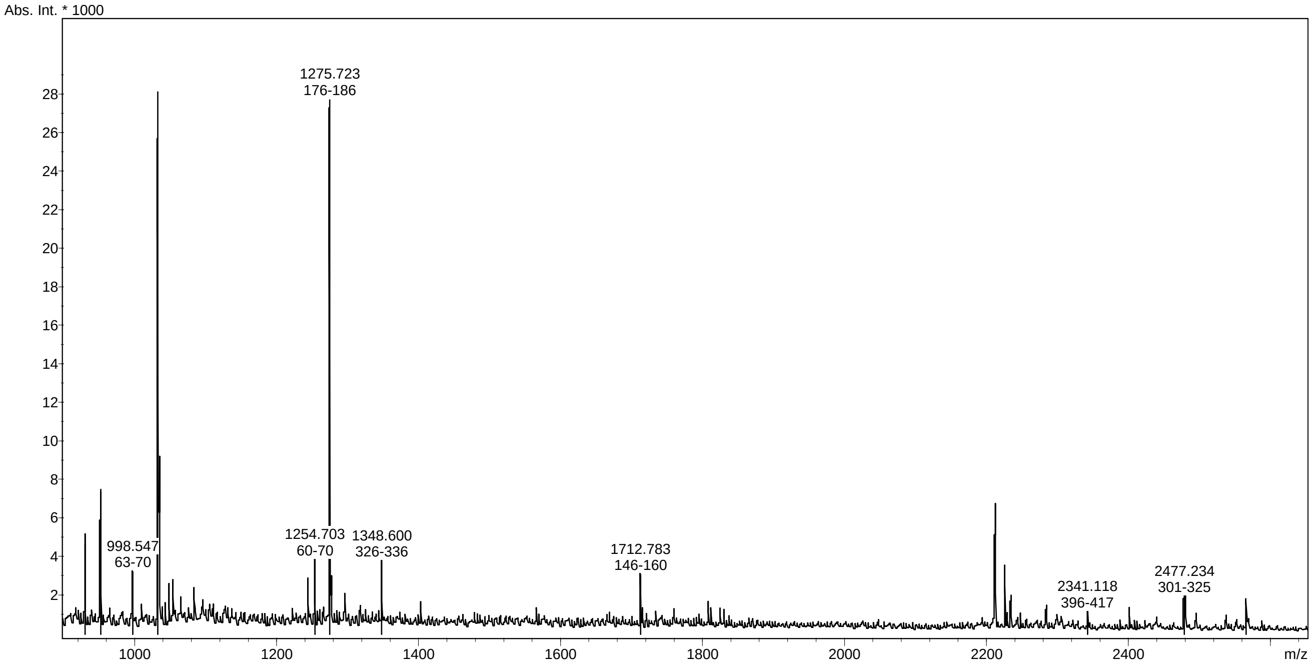

A representative MALDI-ToF PMF spectrum of spot 72

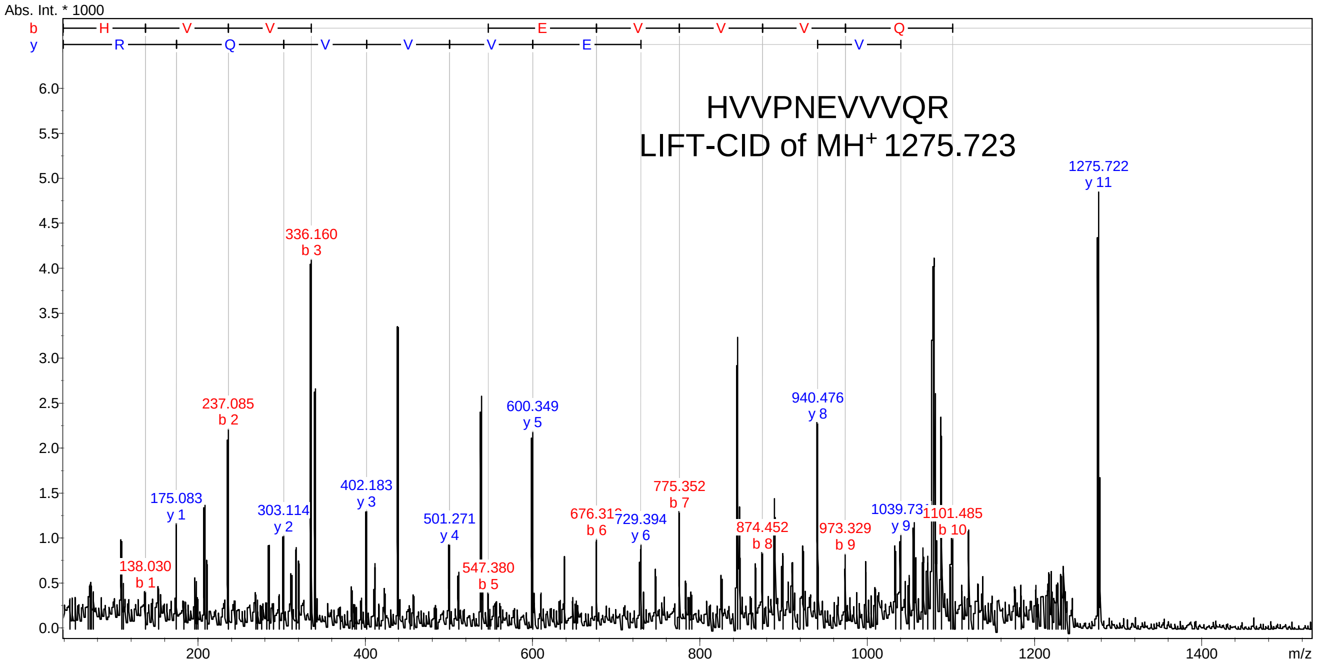

MSMS sequence analysis from the fragmentation of a precursor ion m/z 1275.723 by MALDI-ToF/ToF mass spectrometer

SPOT 73

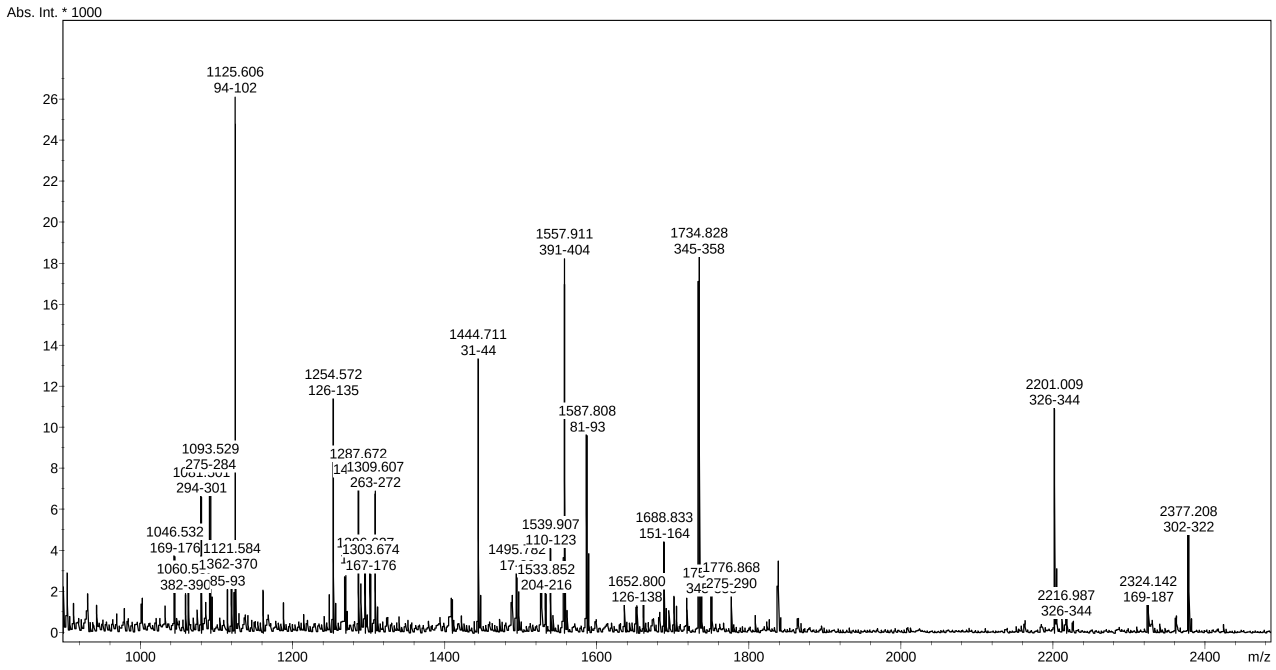

A representative MALDI-ToF PMF spectrum of spot 73

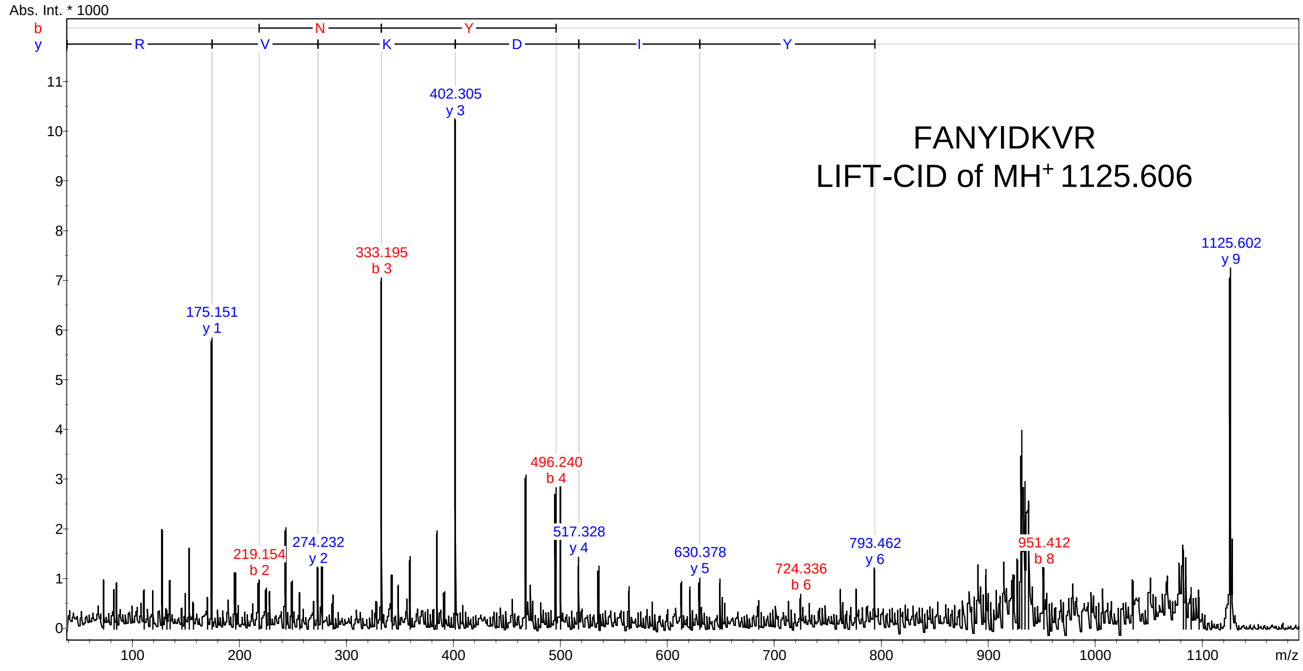

MSMS sequence analysis from the fragmentation of a precursor ion m/z 1125.606 by MALDI-ToF/ToF mass spectrometer

SPOT 74

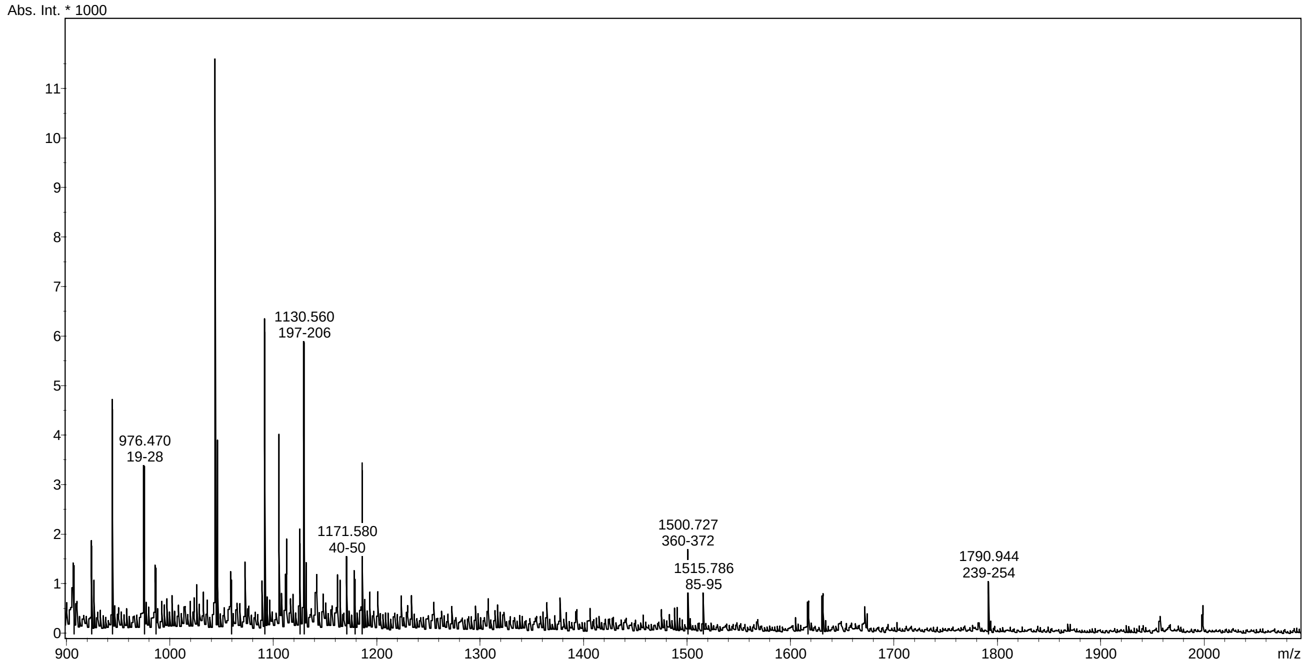

A representative MALDI-ToF PMF spectrum of spot 74

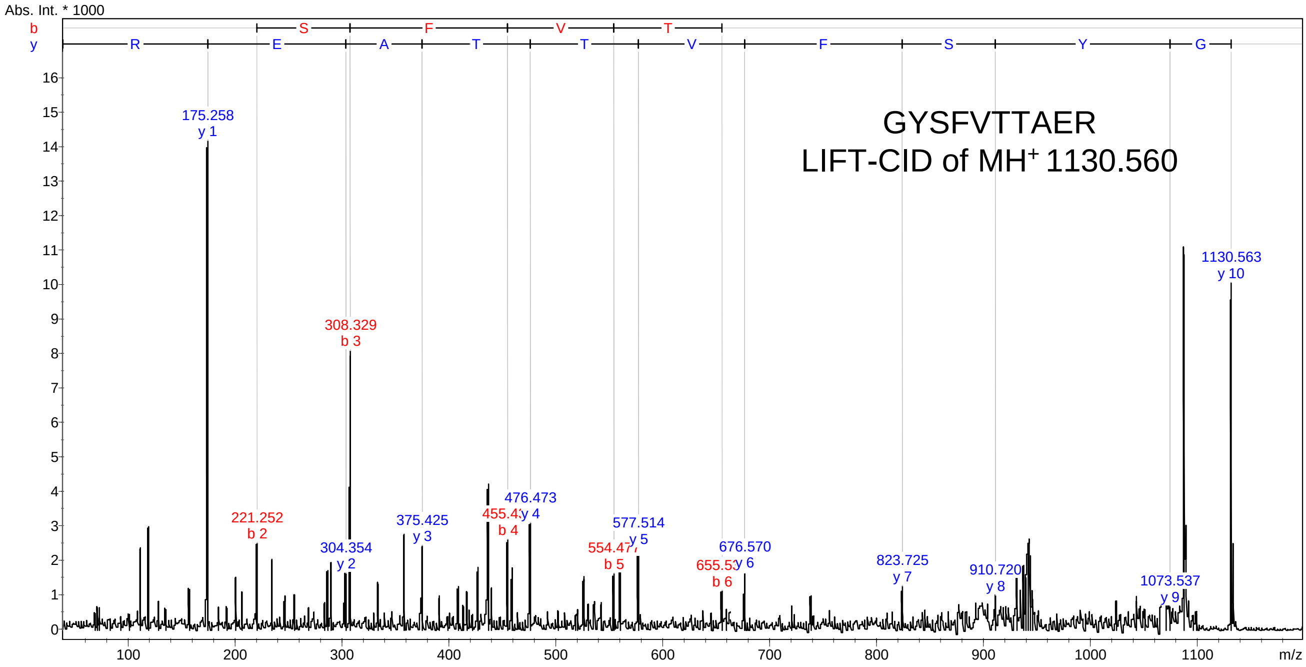

MSMS sequence analysis from the fragmentation of a precursor ion m/z 1130.560 by MALDI-ToF/ToF mass spectrometer

SPOT 75

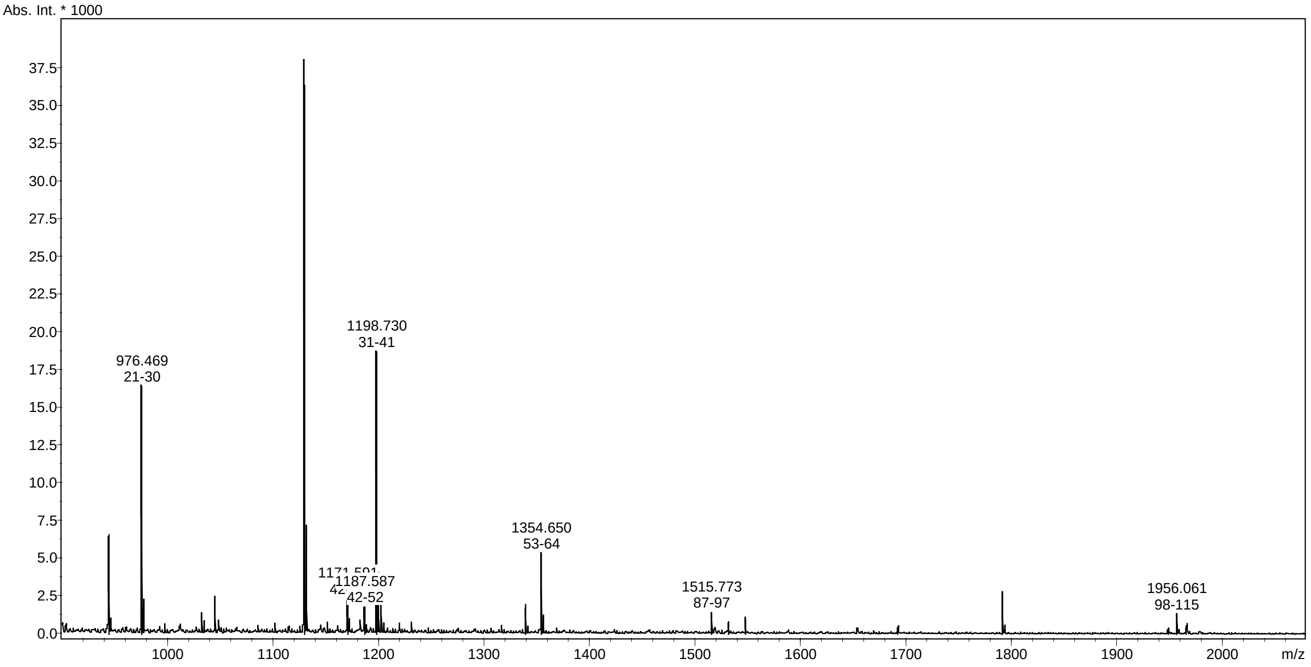

A representative MALDI-ToF PMF spectrum of spot 75

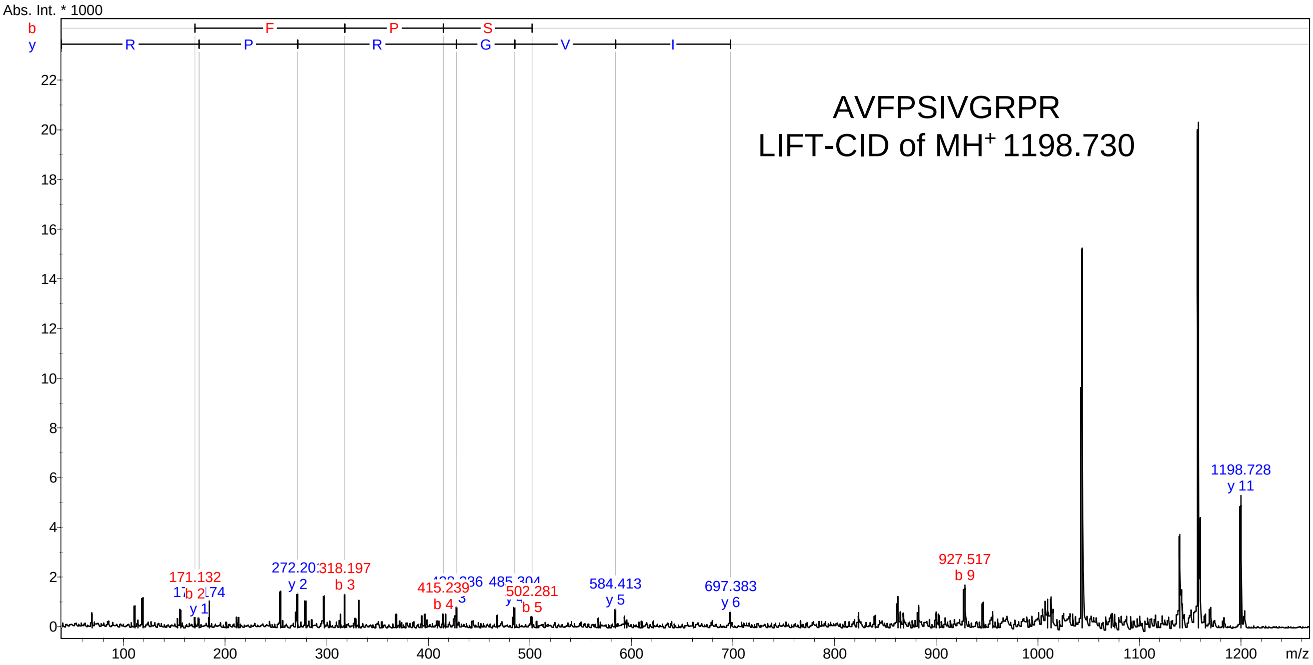

MSMS sequence analysis from the fragmentation of a precursor ion m/z 1198.730 by MALDI-ToF/ToF mass spectrometer

SPOT 76

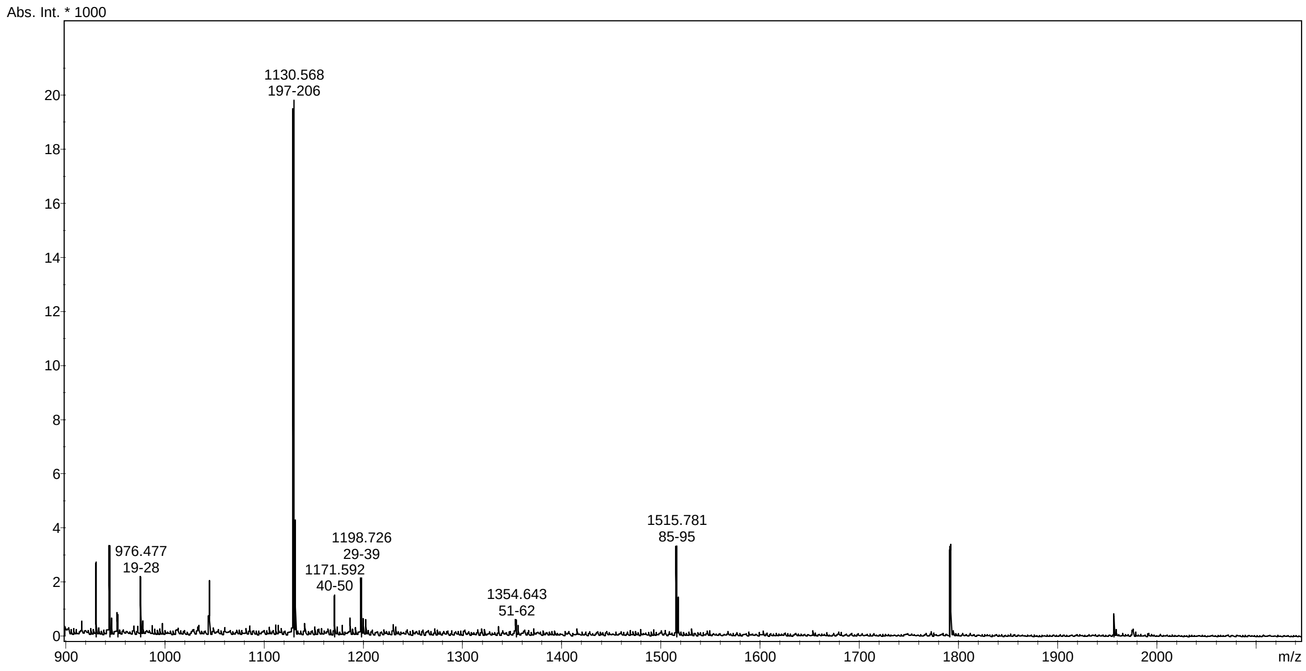

A representative MALDI-ToF PMF spectrum of spot 76

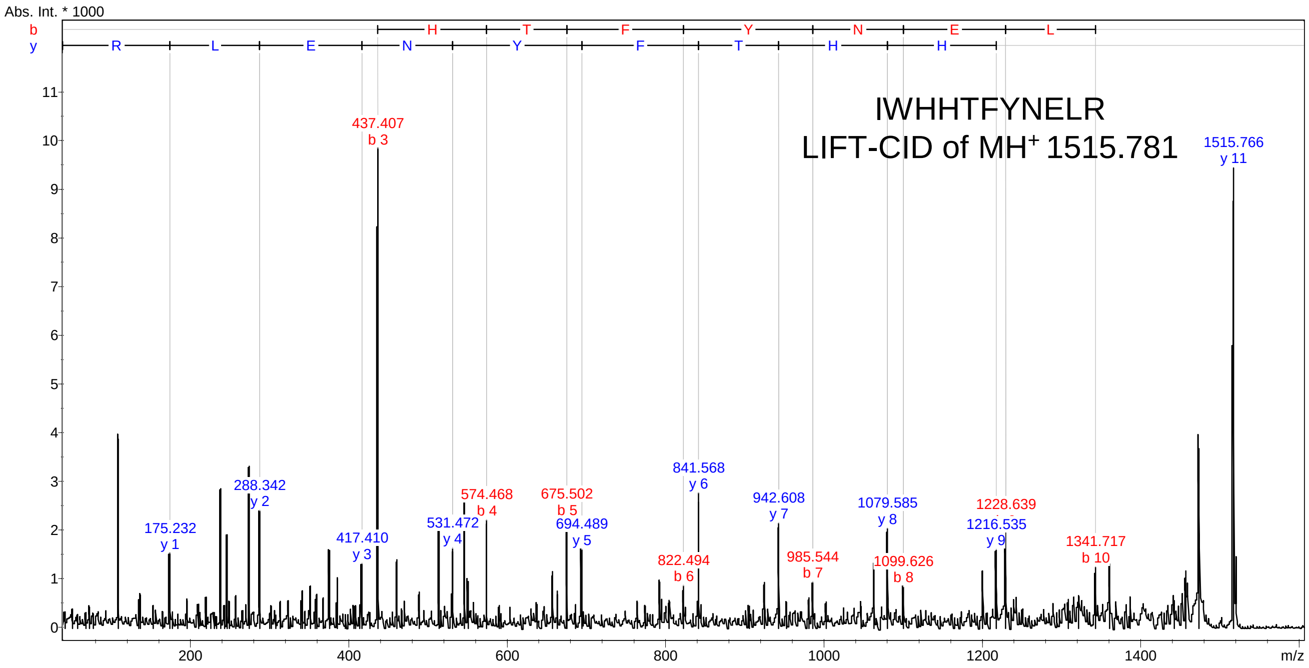

MSMS sequence analysis from the fragmentation of a precursor ion m/z 1515.781 by MALDI-ToF/ToF mass spectrometer

SPOT 77

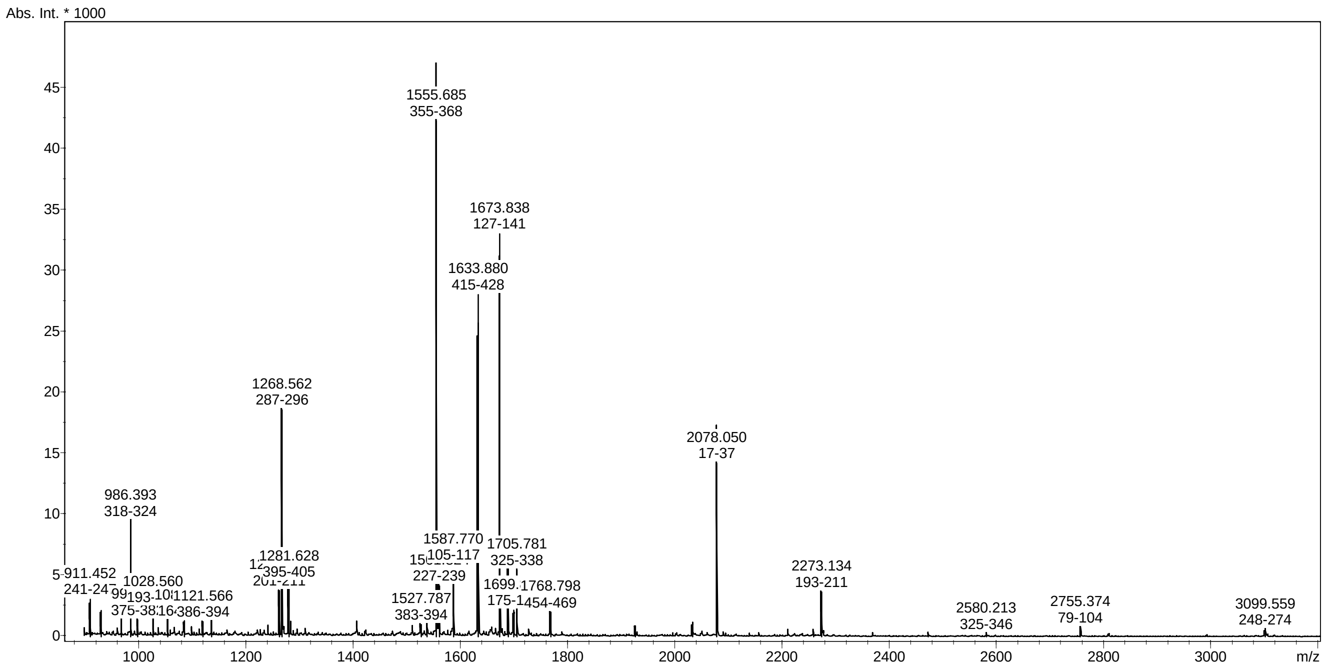

A representative MALDI-ToF PMF spectrum of spot 77

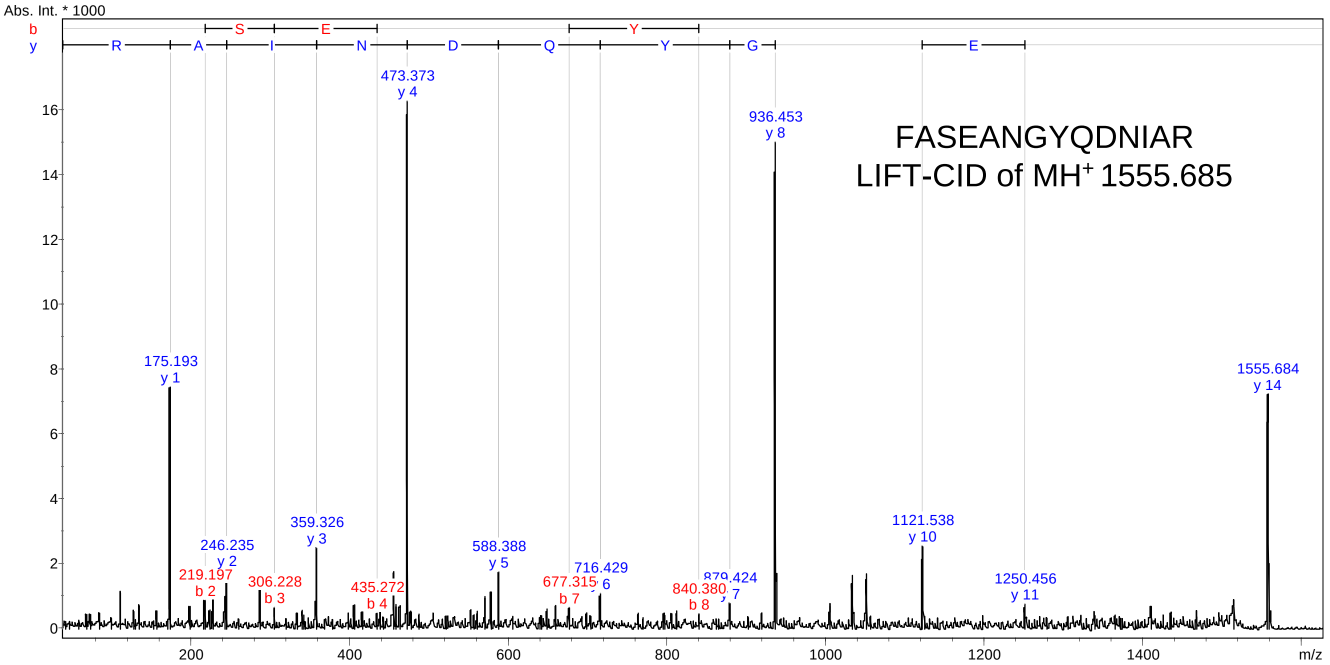

MSMS sequence analysis from the fragmentation of a precursor ion m/z 1555.685 by MALDI-ToF/ToF mass spectrometer

SPOT 78

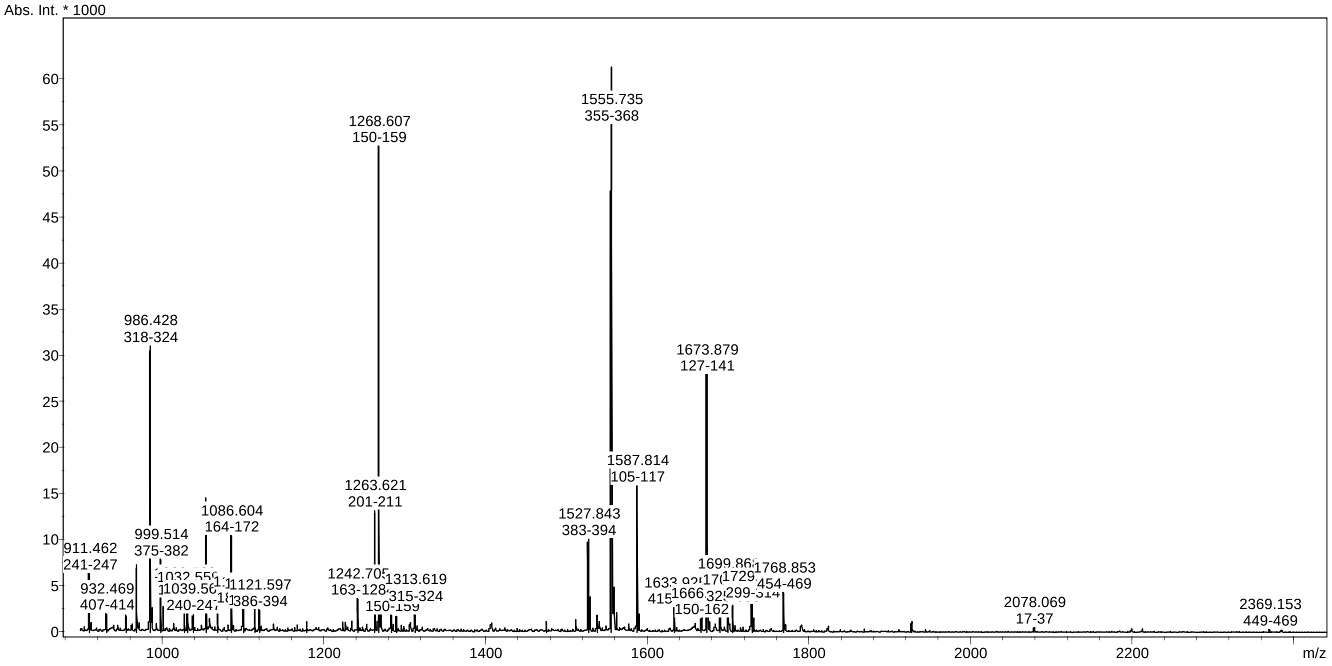

A representative MALDI-ToF PMF spectrum of spot 78

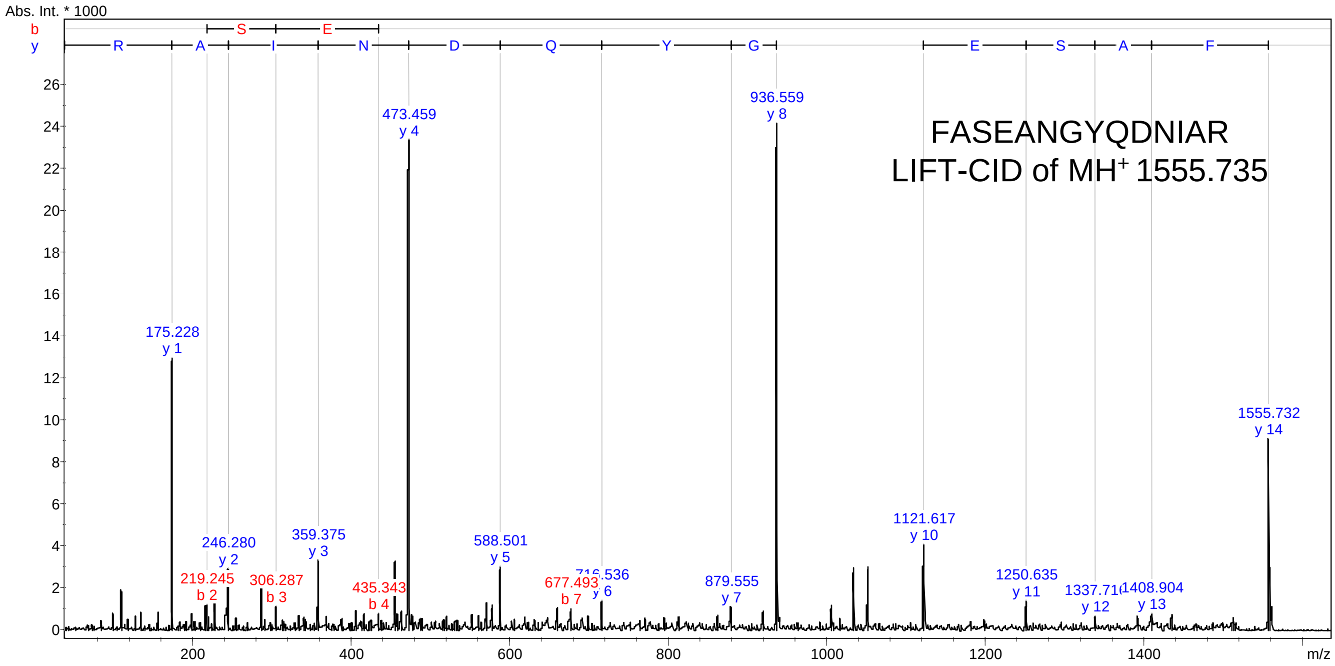

MSMS sequence analysis from the fragmentation of a precursor ion m/z 1555.735 by MALDI-ToF/ToF mass spectrometer

SPOT 79

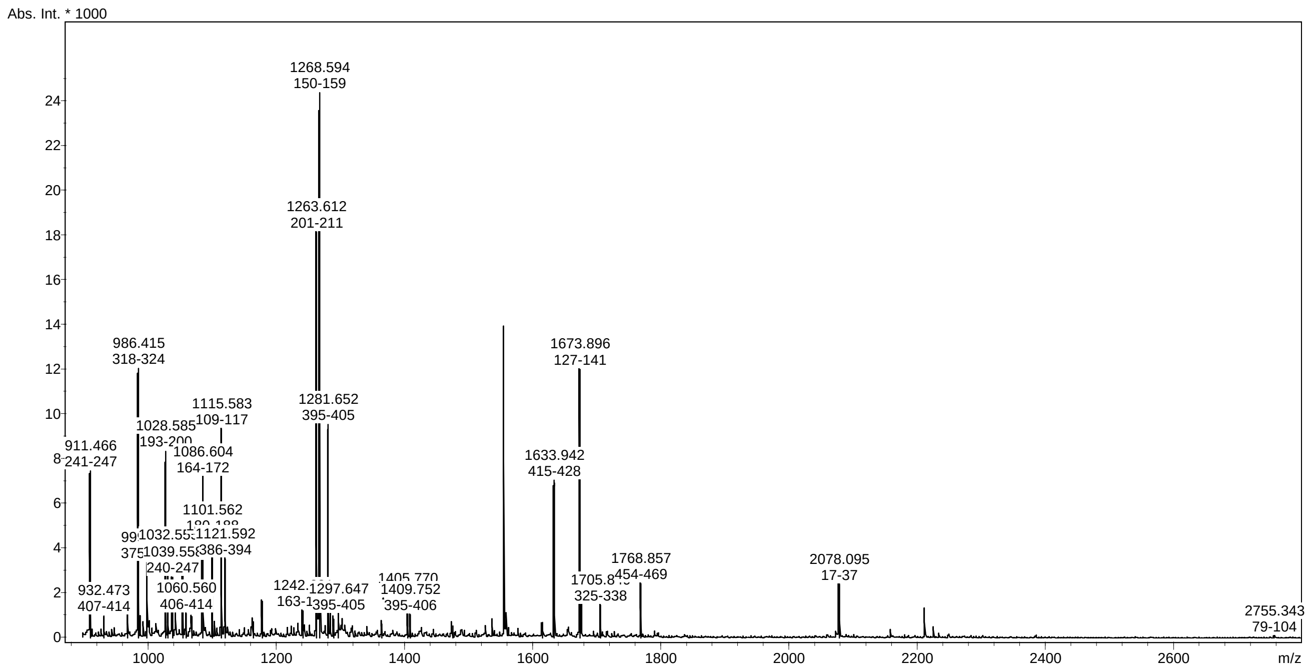

A representative MALDI-ToF PMF spectrum of spot 79

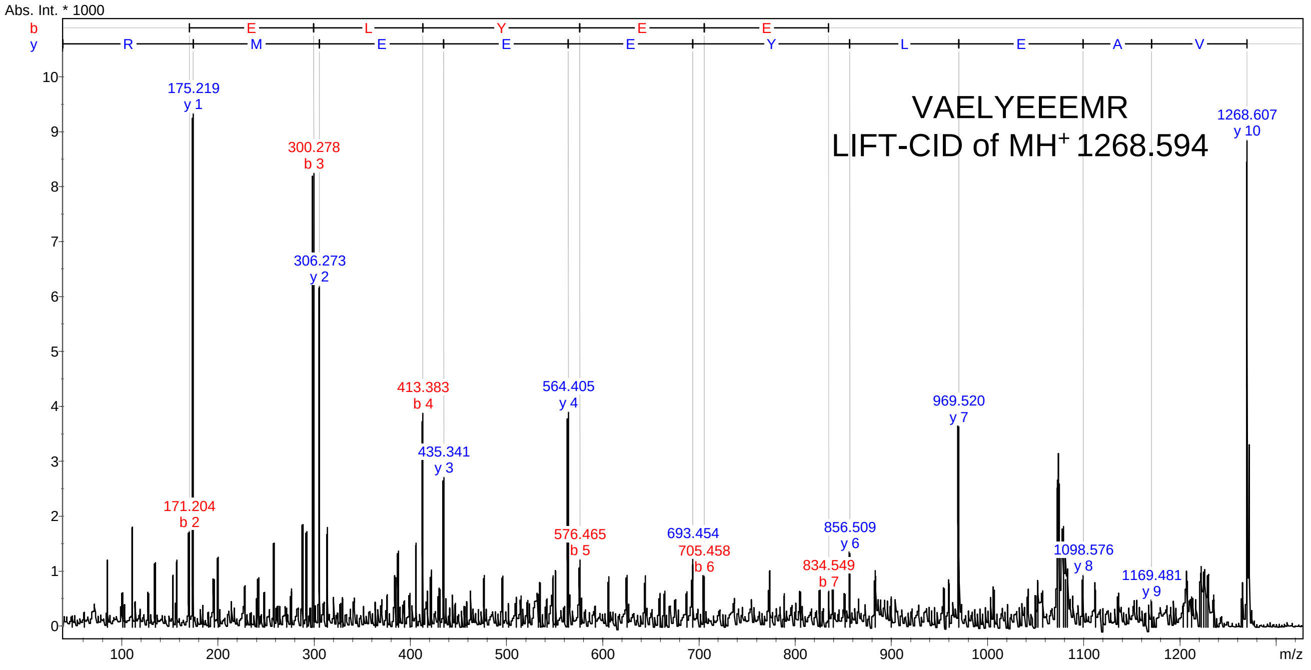

MSMS sequence analysis from the fragmentation of a precursor ion m/z 1268.594 by MALDI-ToF/ToF mass spectrometer

SPOT 81

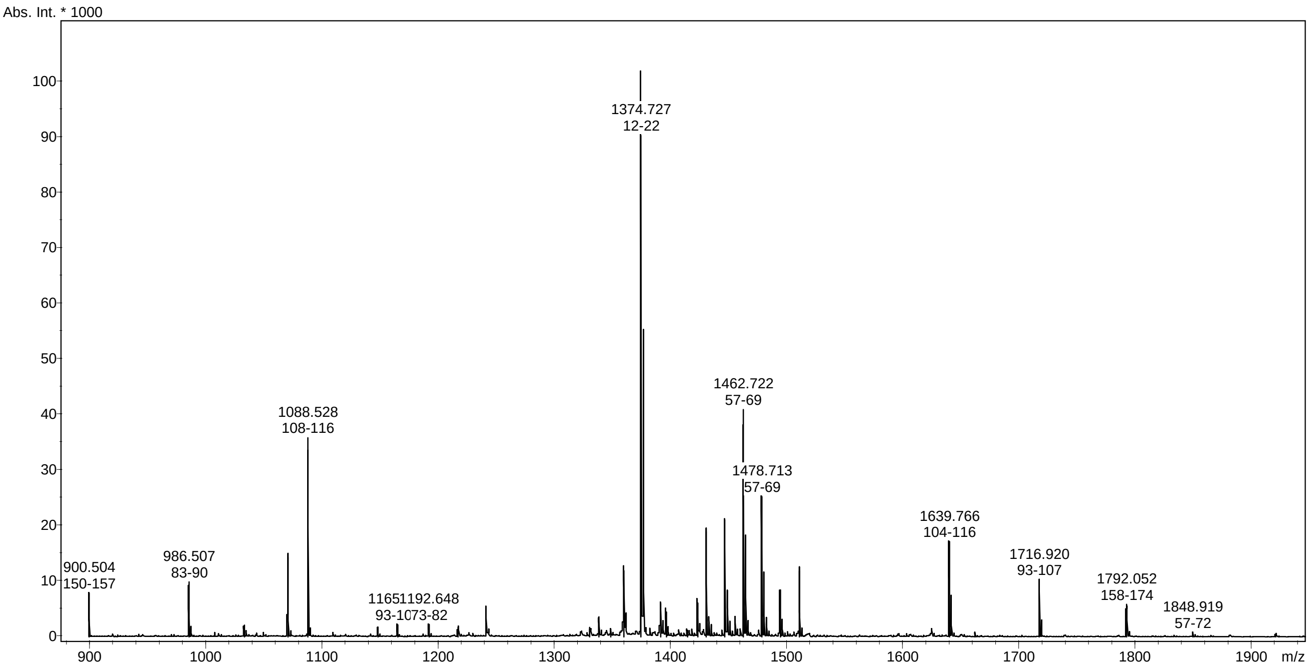

A representative MALDI-ToF PMF spectrum of spot 81

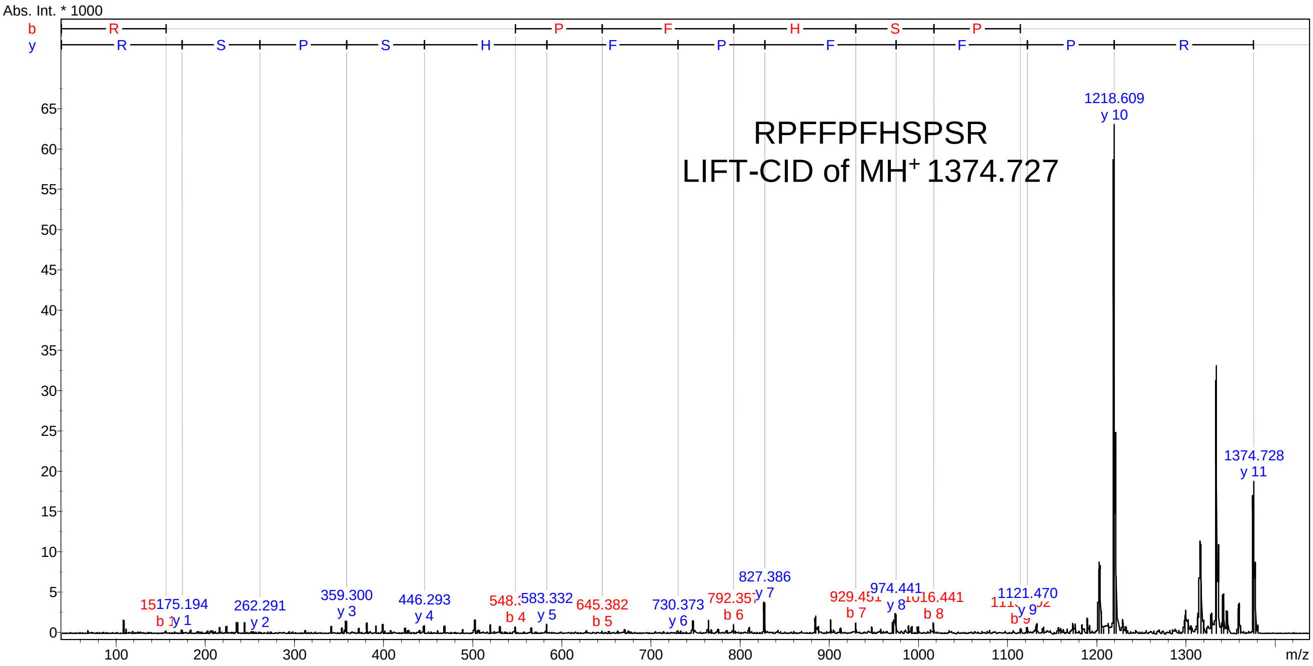

MSMS sequence analysis from the fragmentation of a precursor ion m/z 1374.727 by MALDI-ToF/ToF mass spectrometer

SPOT 83

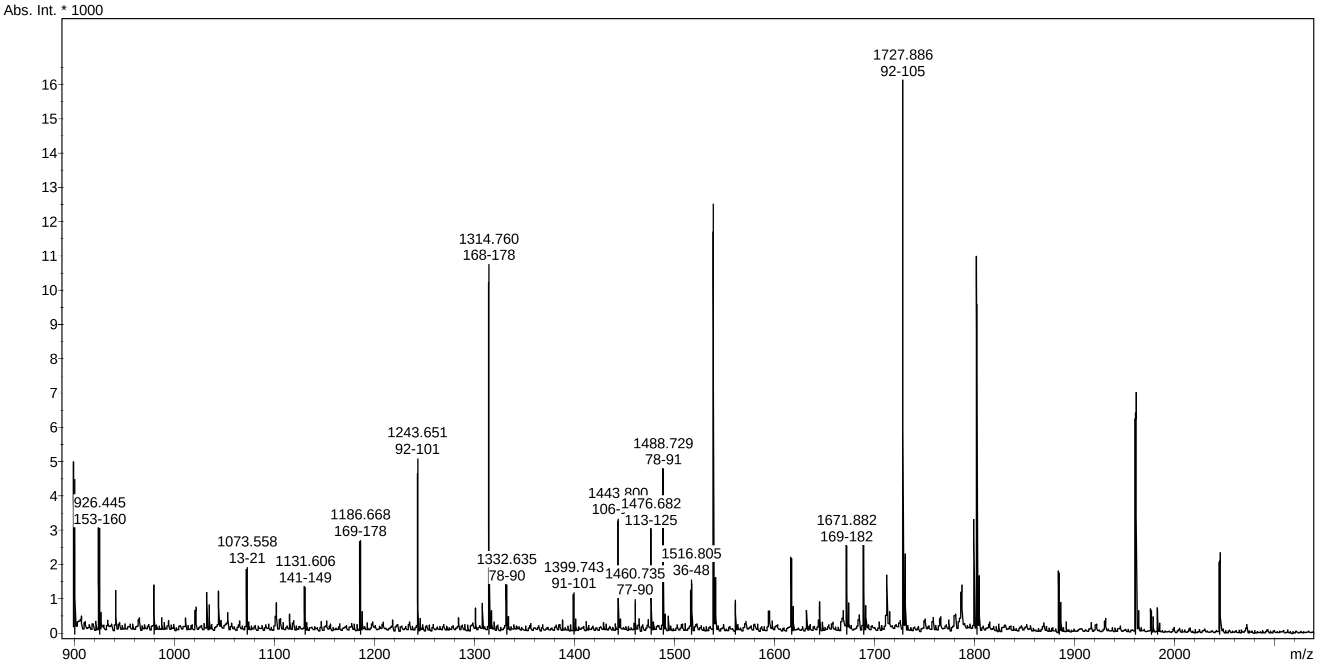

A representative MALDI-ToF PMF spectrum of spot 83

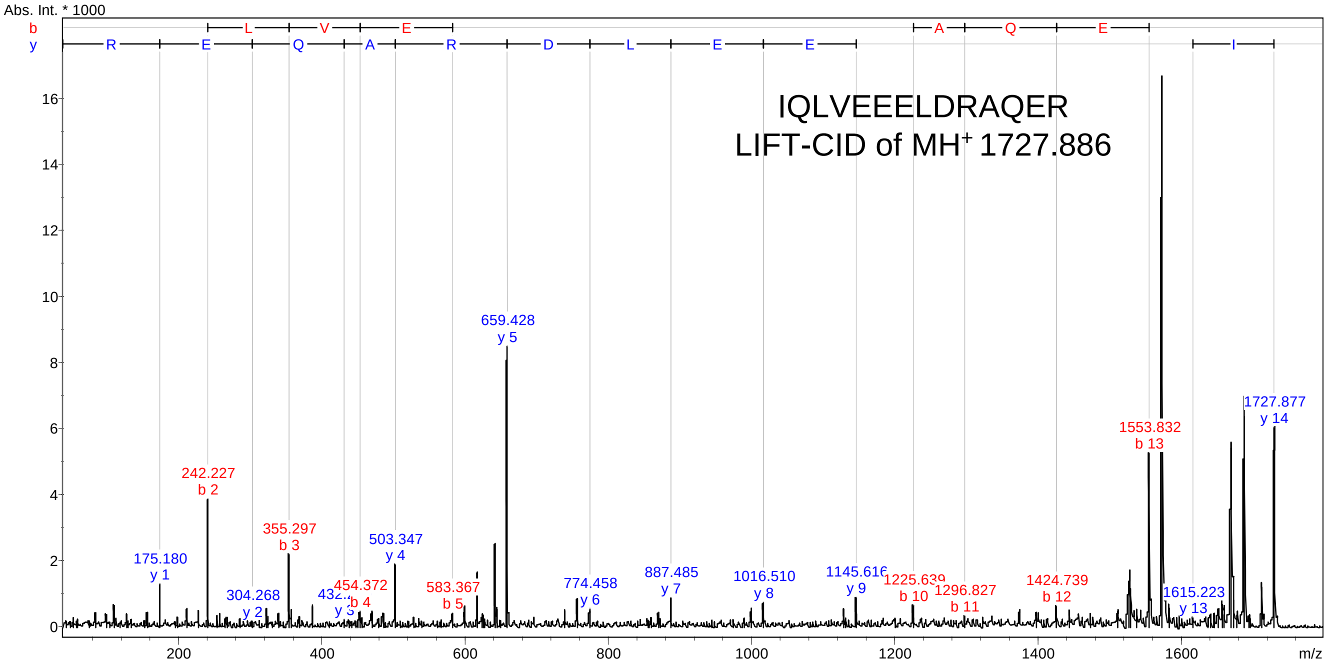

MSMS sequence analysis from the fragmentation of a precursor ion m/z 1727.886 by MALDI-ToF/ToF mass spectrometer

SPOT 84

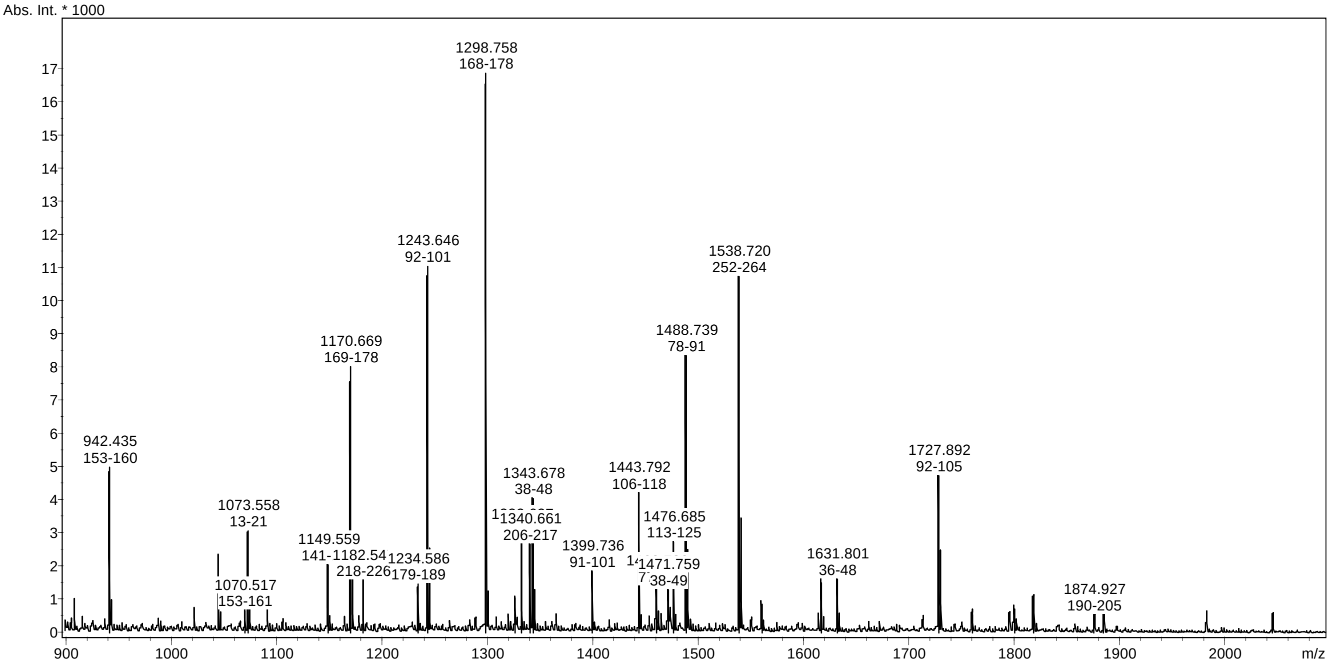

A representative MALDI-ToF PMF spectrum of spot 84

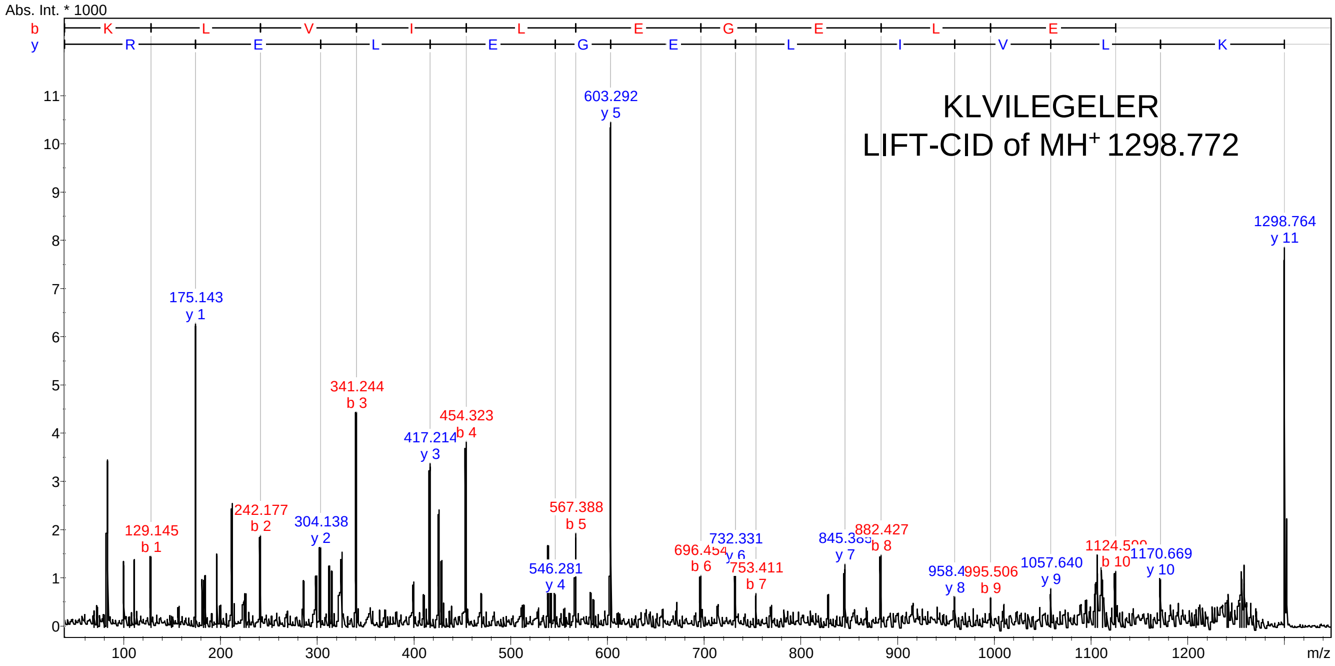

MSMS sequence analysis from the fragmentation of a precursor ion m/z 1298.772 by MALDI-ToF/ToF mass spectrometer

SPOT 85

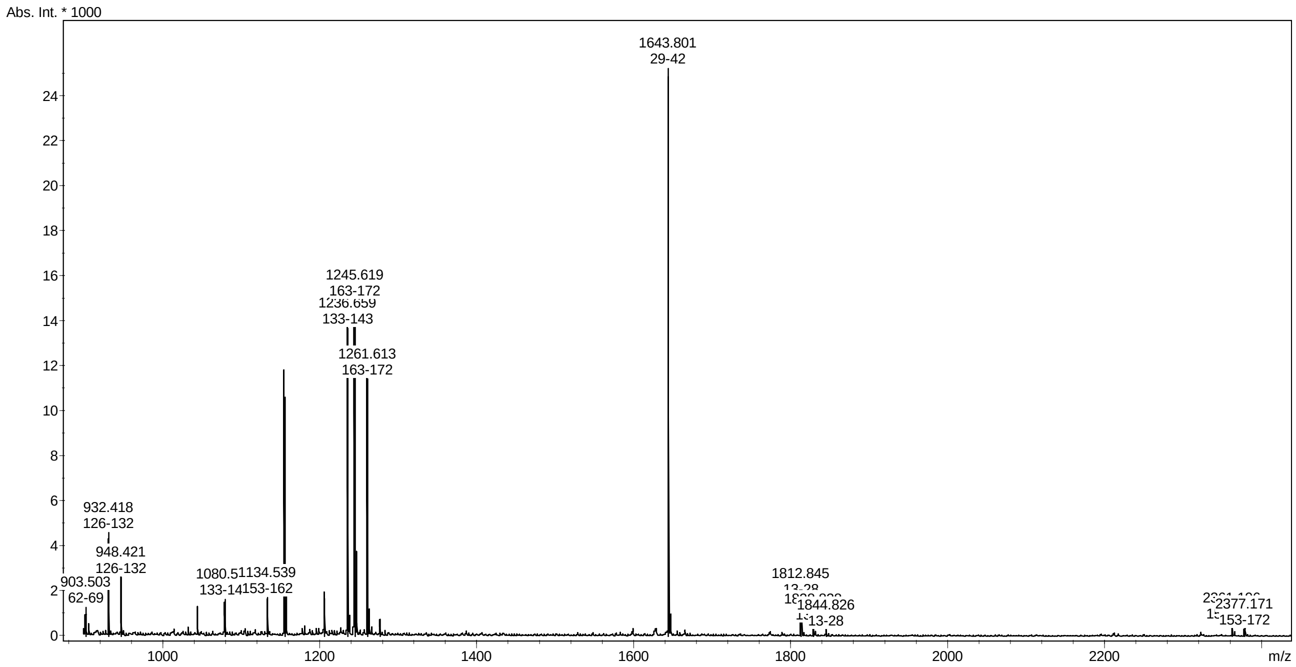

A representative MALDI-ToF PMF spectrum of spot 85

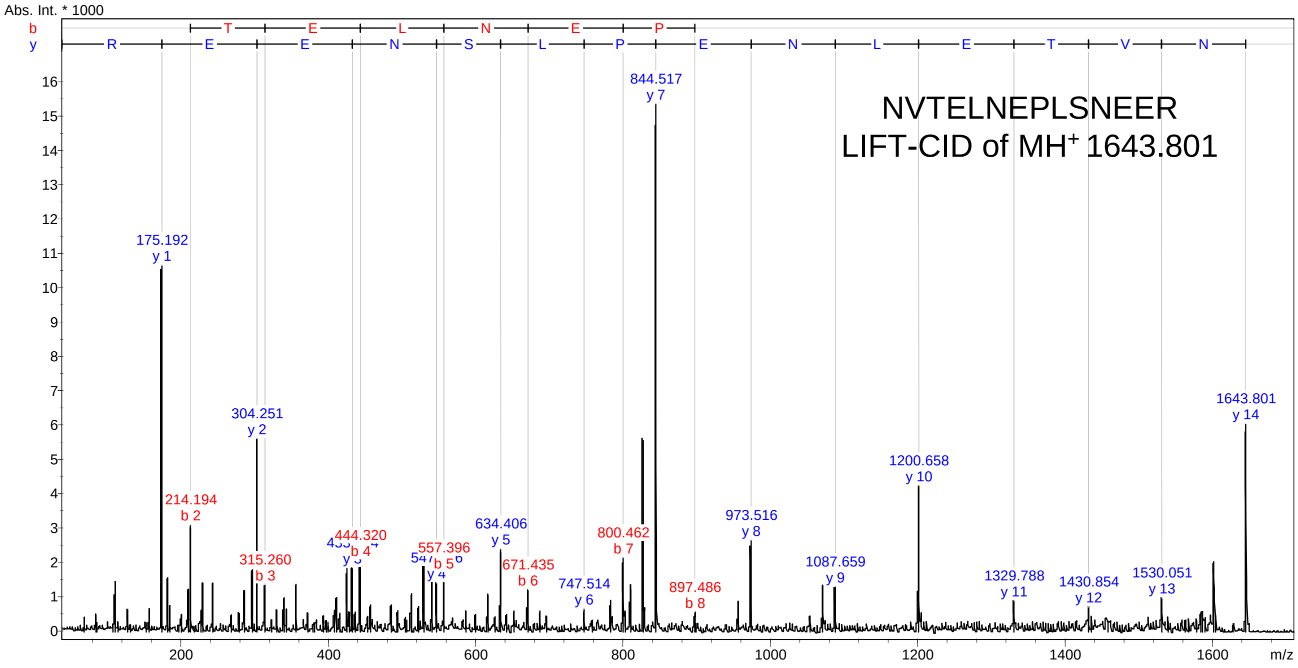

MSMS sequence analysis from the fragmentation of a precursor ion m/z 1643.801 by MALDI-ToF/ToF mass spectrometer

SPOT 86

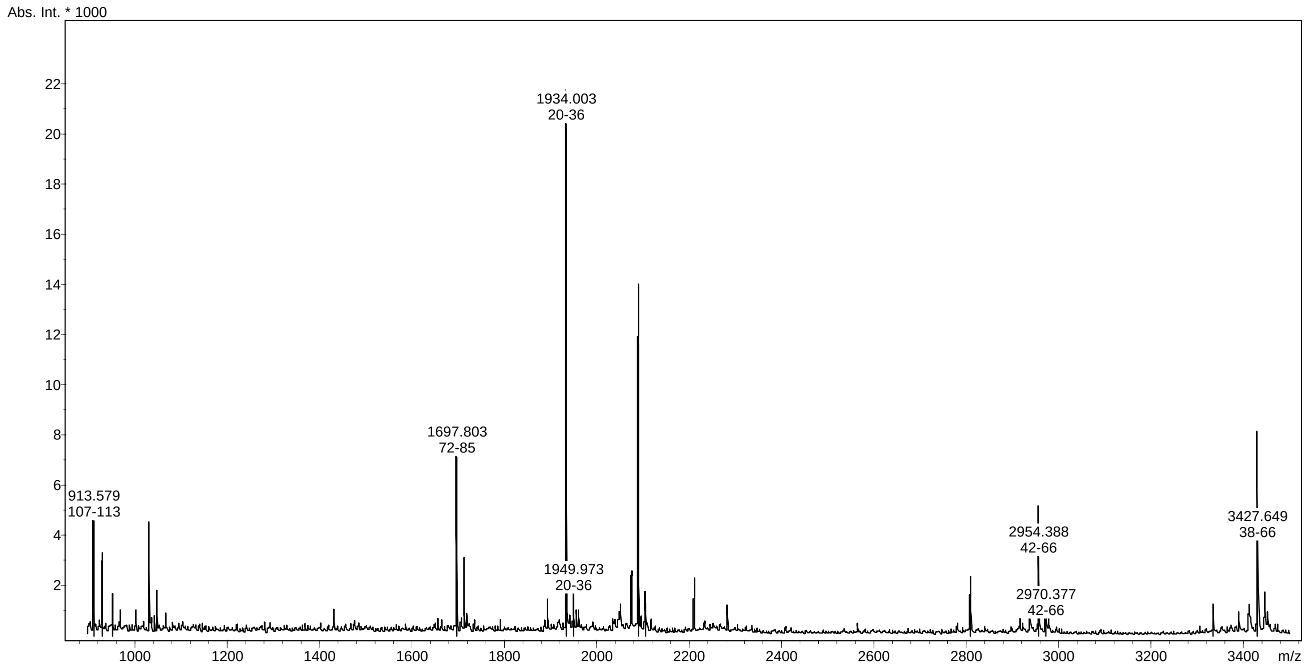

A representative MALDI-ToF PMF spectrum of spot 86

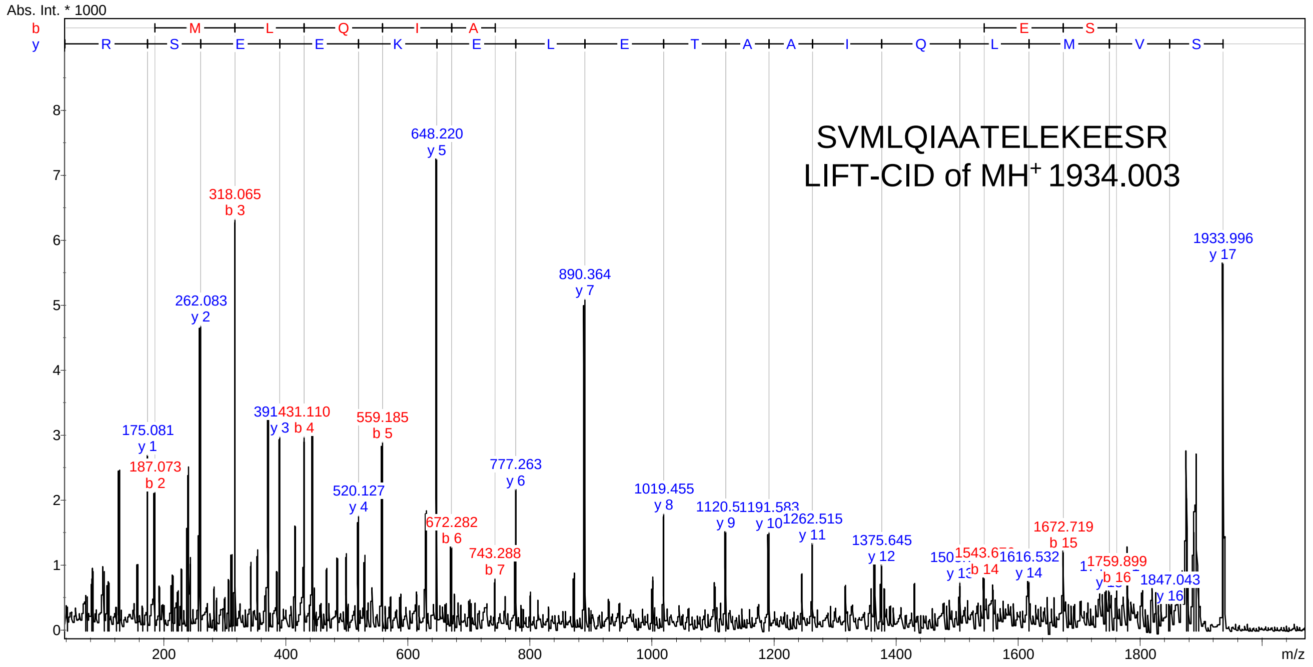

MSMS sequence analysis from the fragmentation of a precursor ion m/z 1934.003 by MALDI-ToF/ToF mass spectrometer

SPOT 87

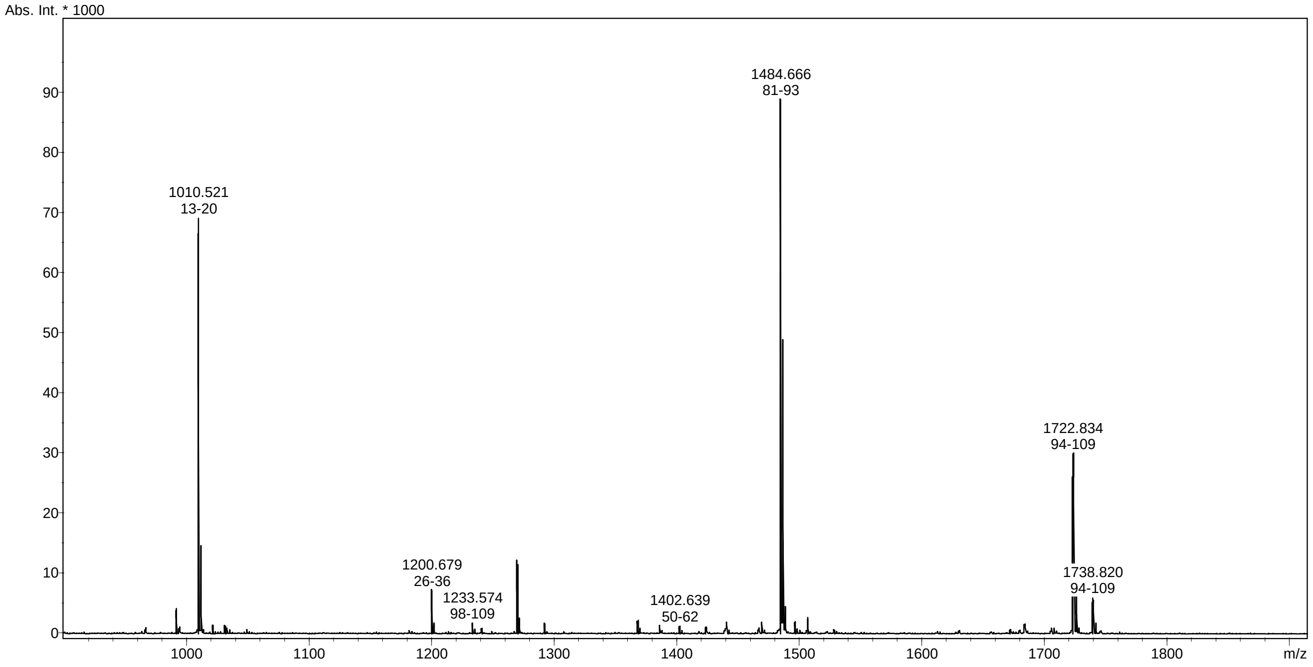

A representative MALDI-ToF PMF spectrum of spot 87

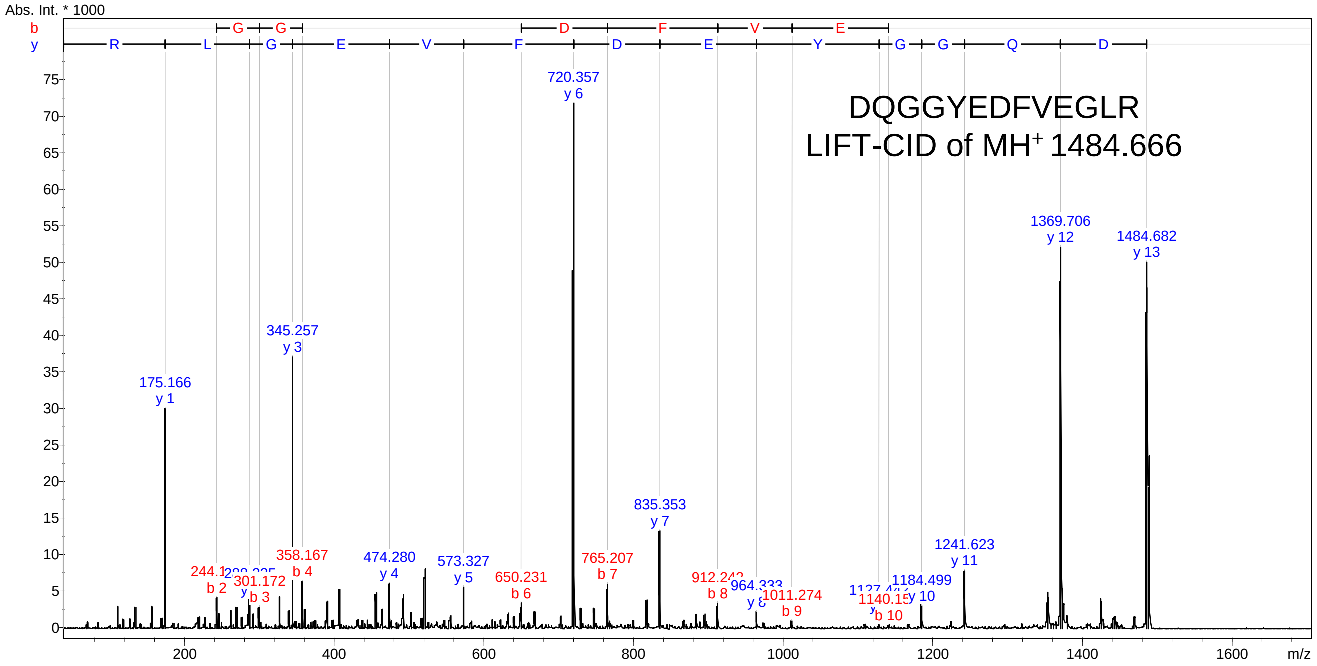

MSMS sequence analysis from the fragmentation of a precursor ion m/z 1484.666 by MALDI-ToF/ToF mass spectrometer

SPOT 88

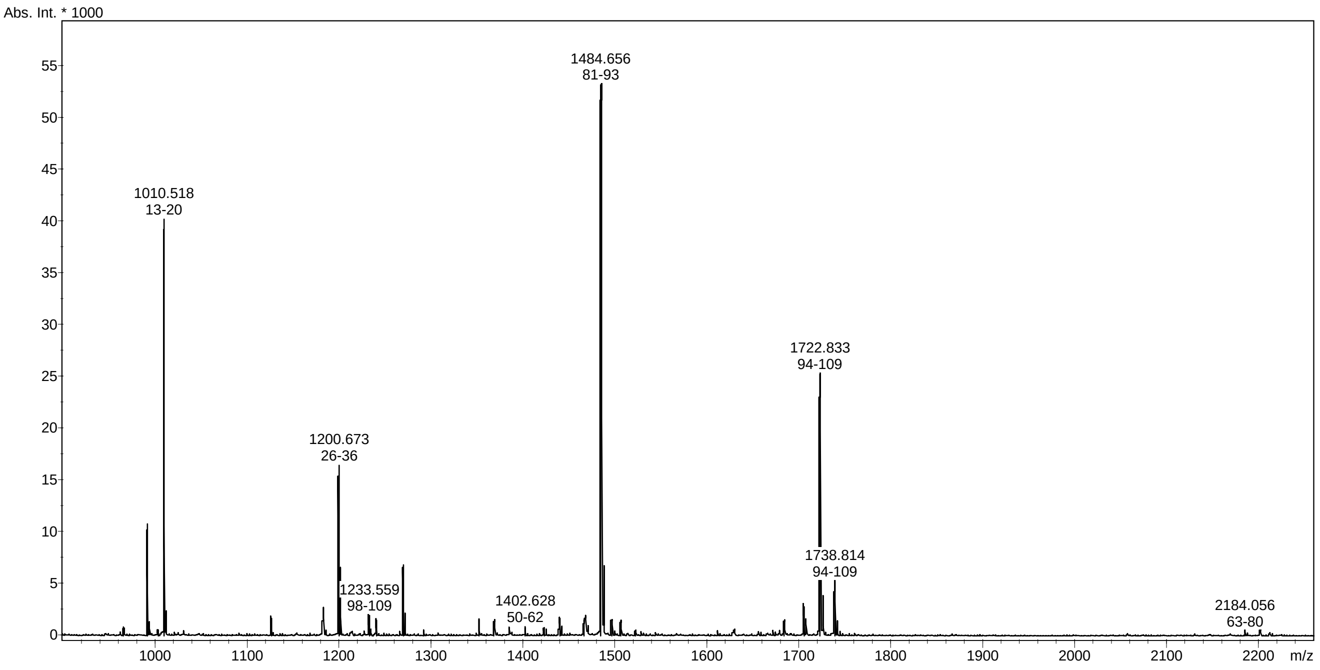

A representative MALDI-ToF PMF spectrum of spot 88

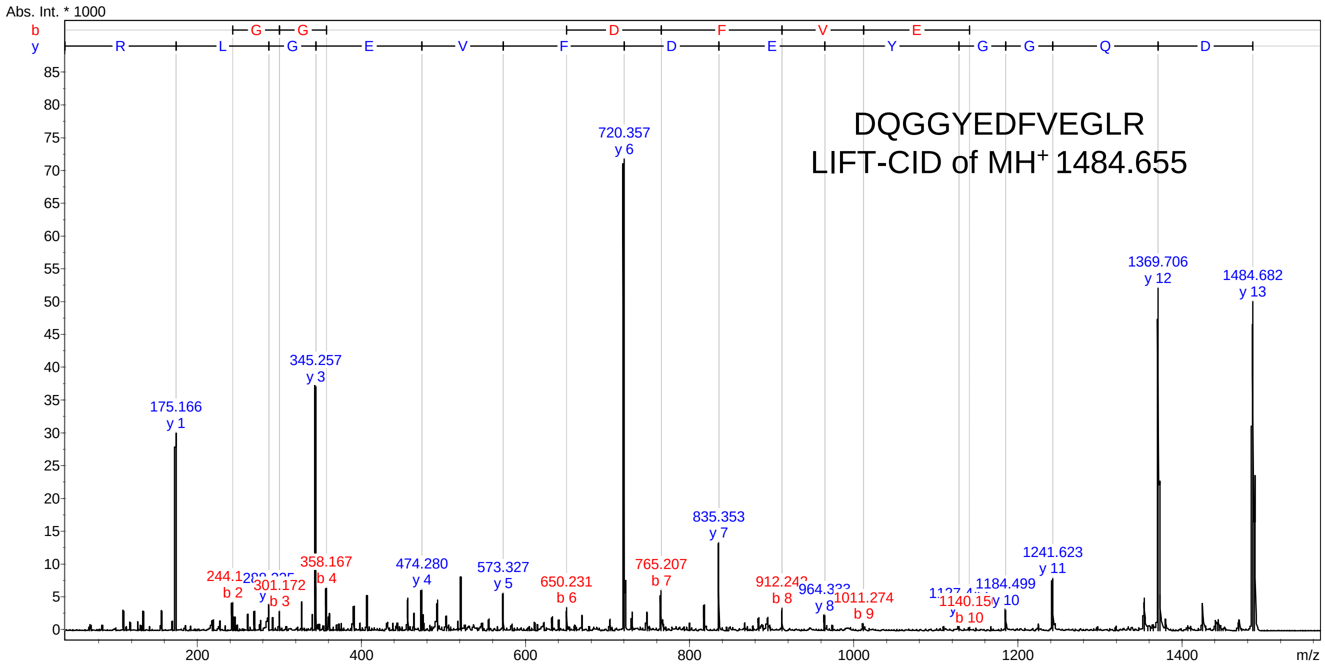

MSMS sequence analysis from the fragmentation of a precursor ion m/z 1484.655 by MALDI-ToF/ToF mass spectrometer

SPOT 89

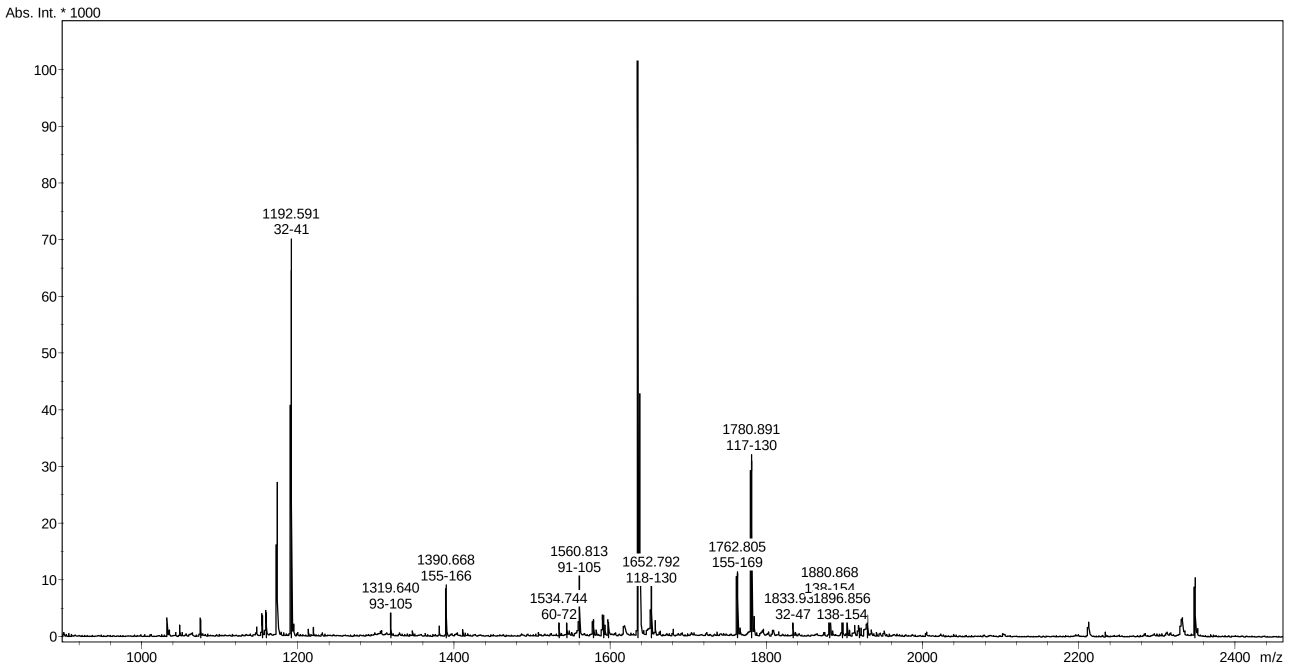

A representative MALDI-ToF PMF spectrum of spot 89

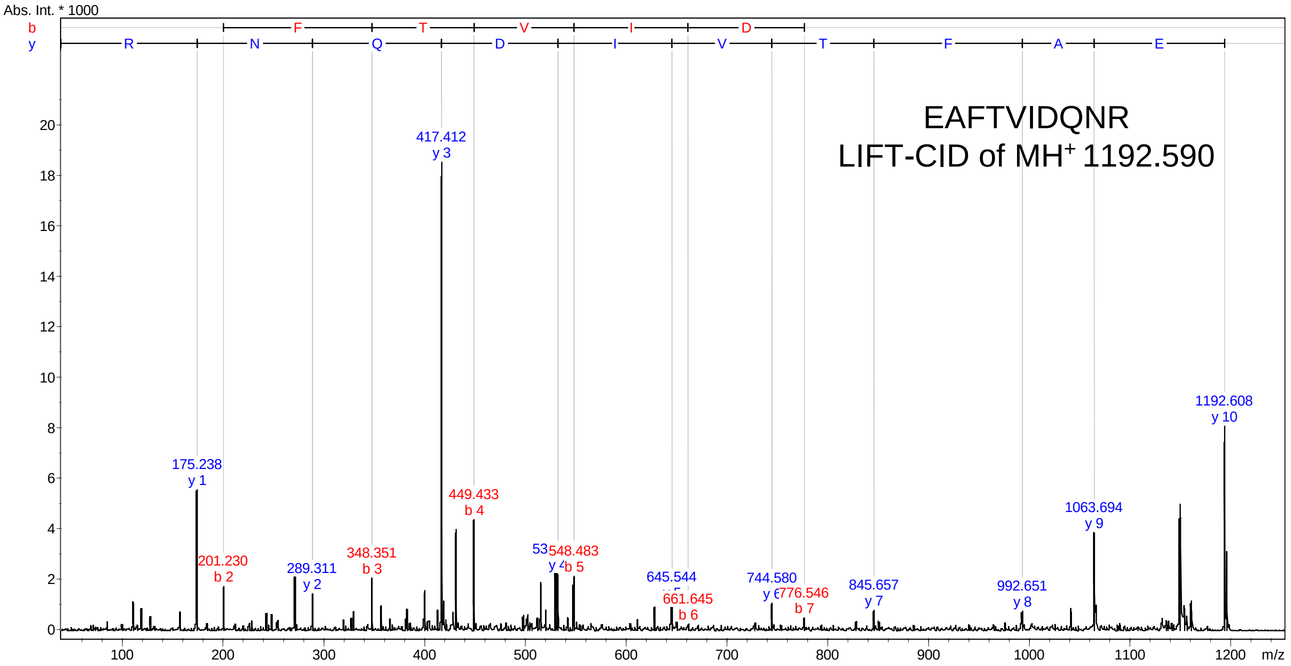

MSMS sequence analysis from the fragmentation of a precursor ion m/z 1192.590 by MALDI-ToF/ToF mass spectrometer

SPOT 90

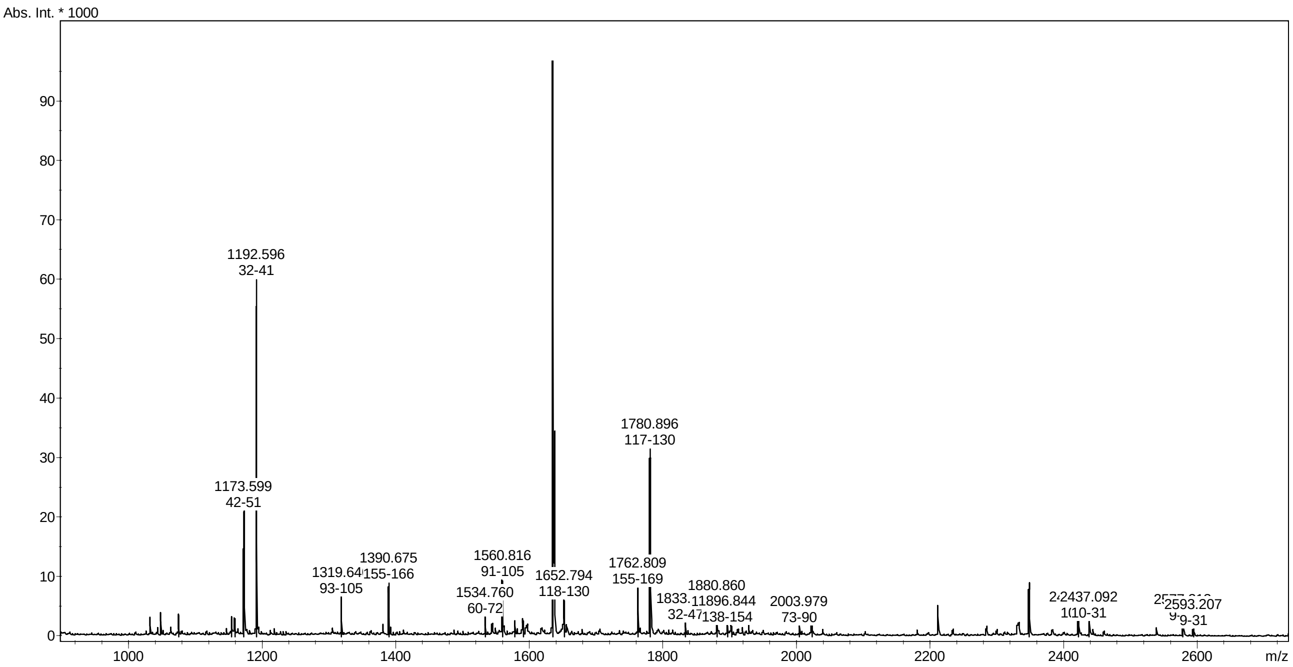

A representative MALDI-ToF PMF spectrum of spot 90

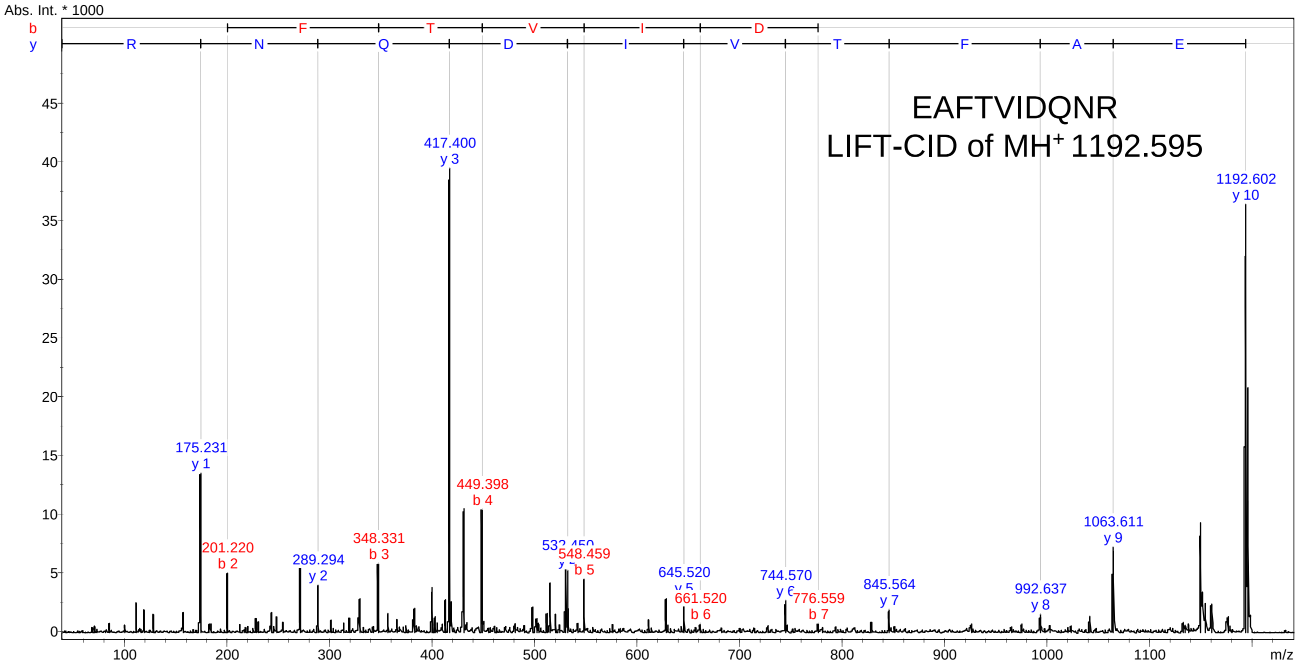

MSMS sequence analysis from the fragmentation of a precursor ion m/z 1192.595 by MALDI-ToF/ToF mass spectrometer

SPOT 91

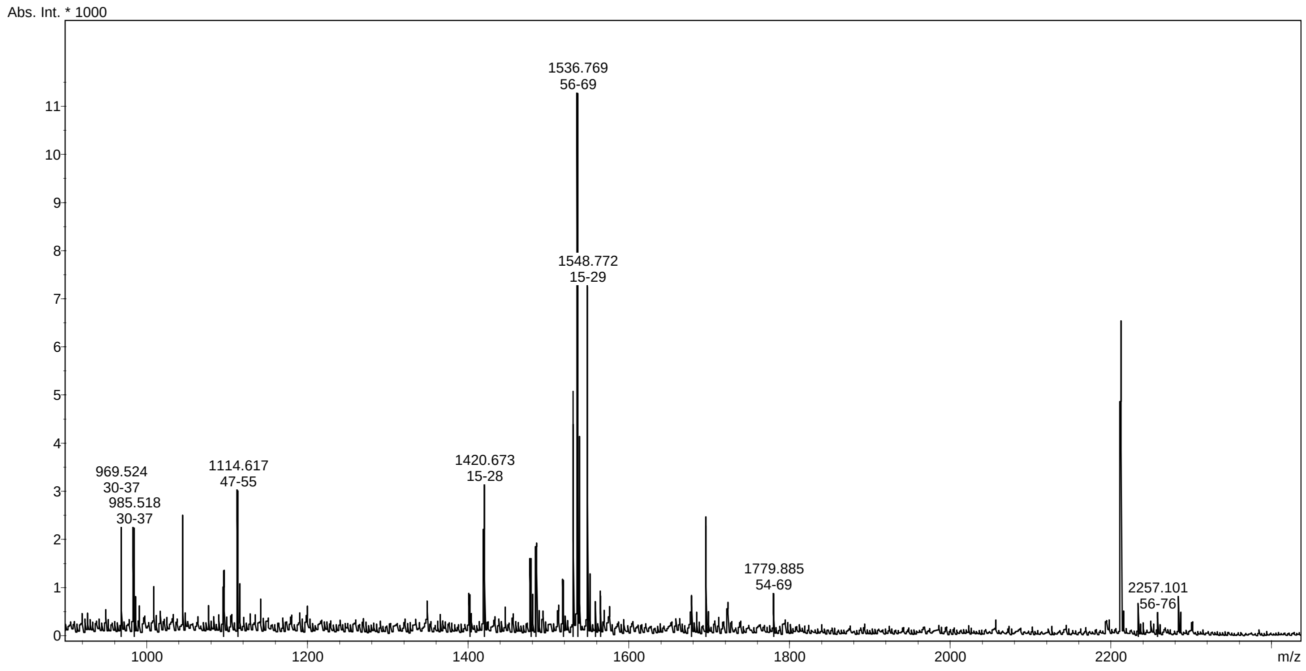

A representative MALDI-ToF PMF spectrum of spot 91

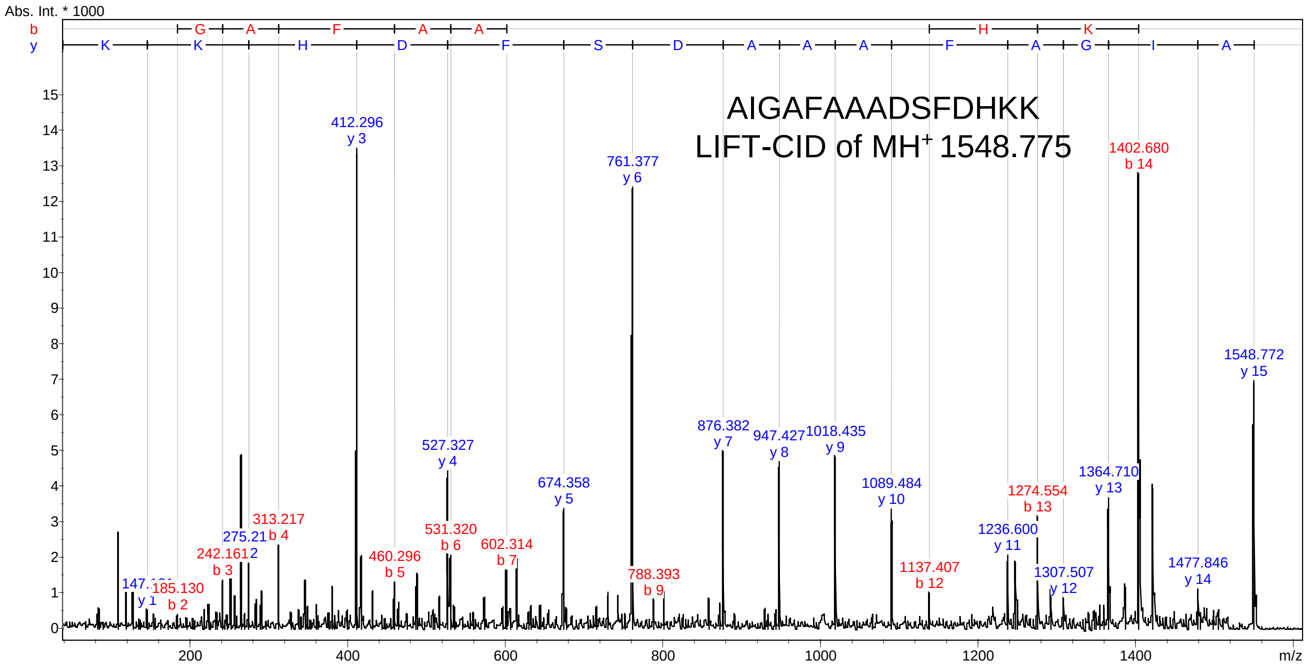

MSMS sequence analysis from the fragmentation of a precursor ion m/z 1548.775 by MALDI-ToF/ToF mass spectrometer

SPOT 92

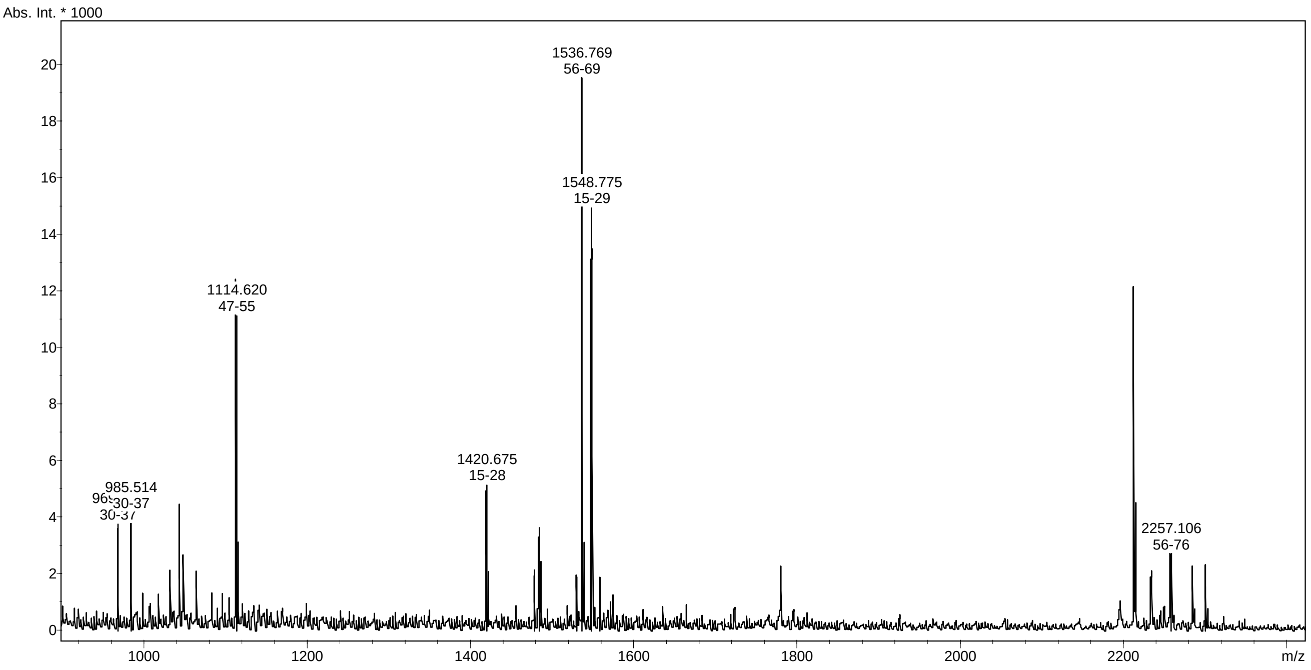

A representative MALDI-ToF PMF spectrum of spot 92

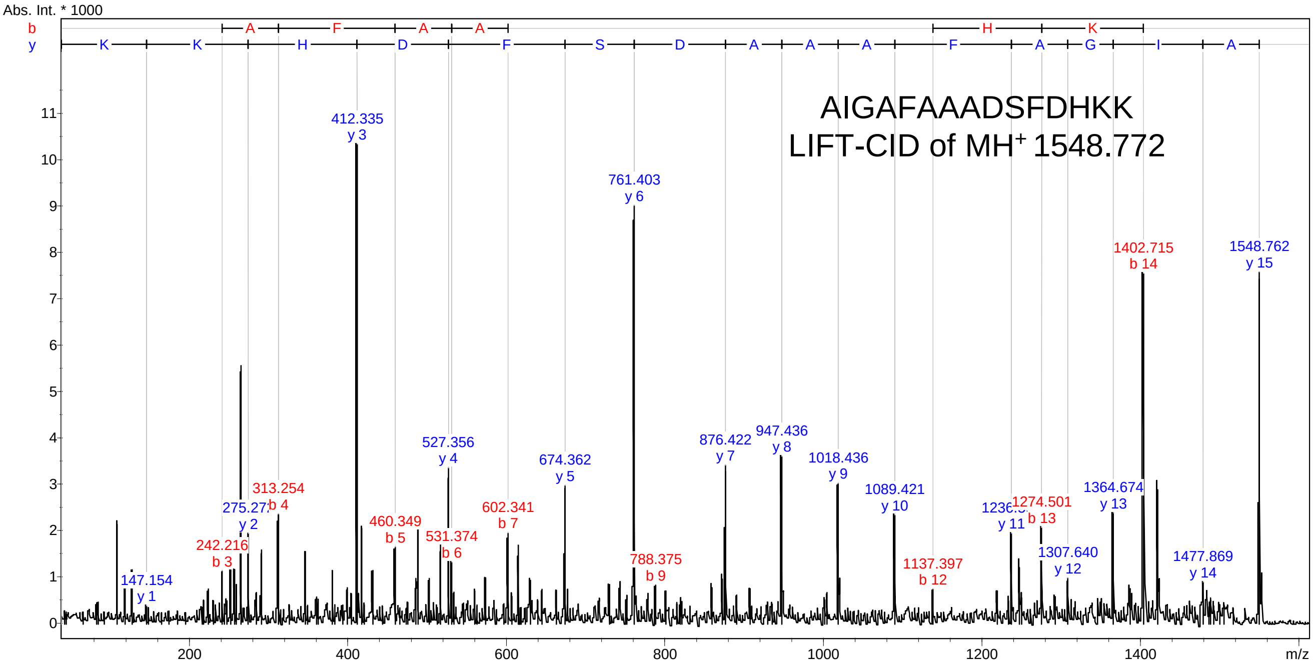

MSMS sequence analysis from the fragmentation of a precursor ion m/z 1548.772 by MALDI-ToF/ToF mass spectrometer

SPOT 94

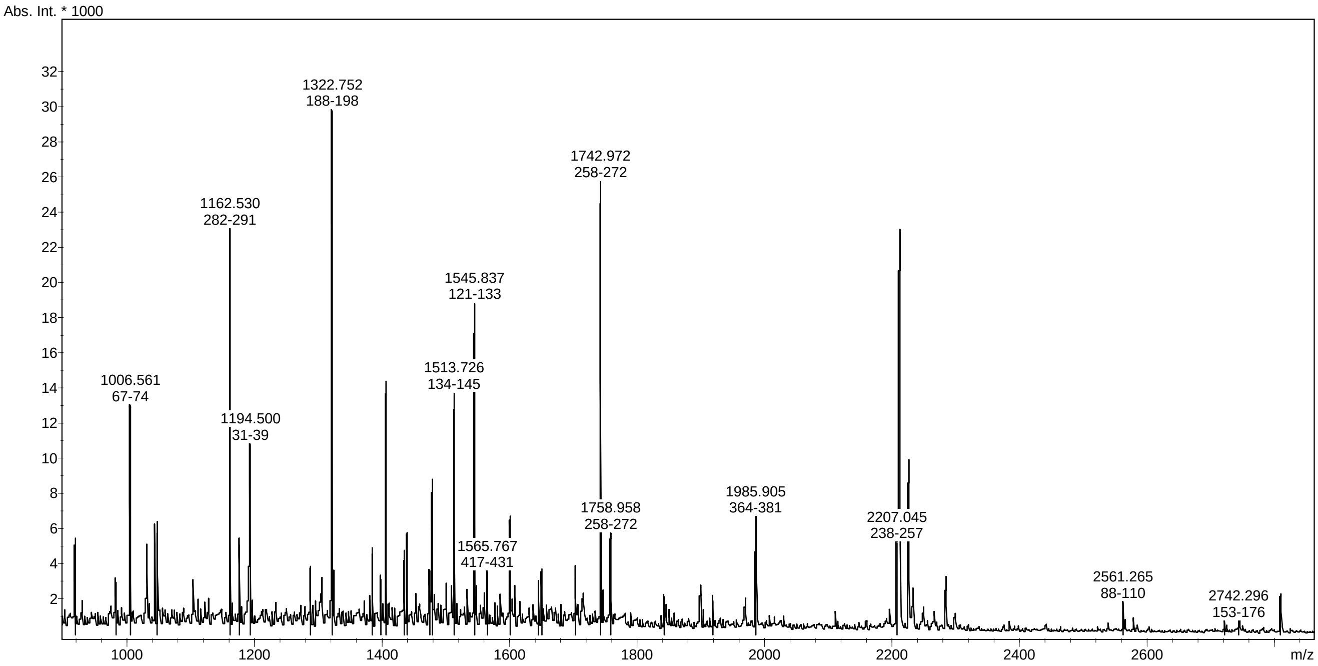

A representative MALDI-ToF PMF spectrum of spot 94

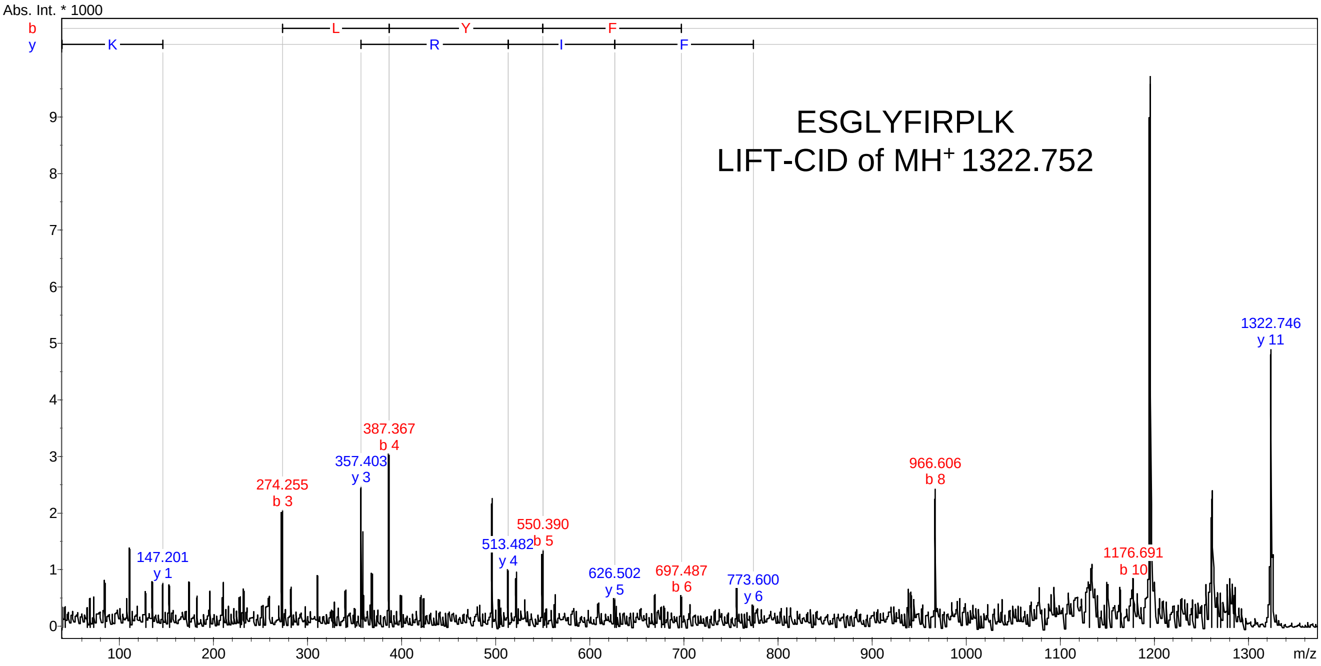

MSMS sequence analysis from the fragmentation of a precursor ion m/z 1322.752 by MALDI-ToF/ToF mass spectrometer

SPOT 95

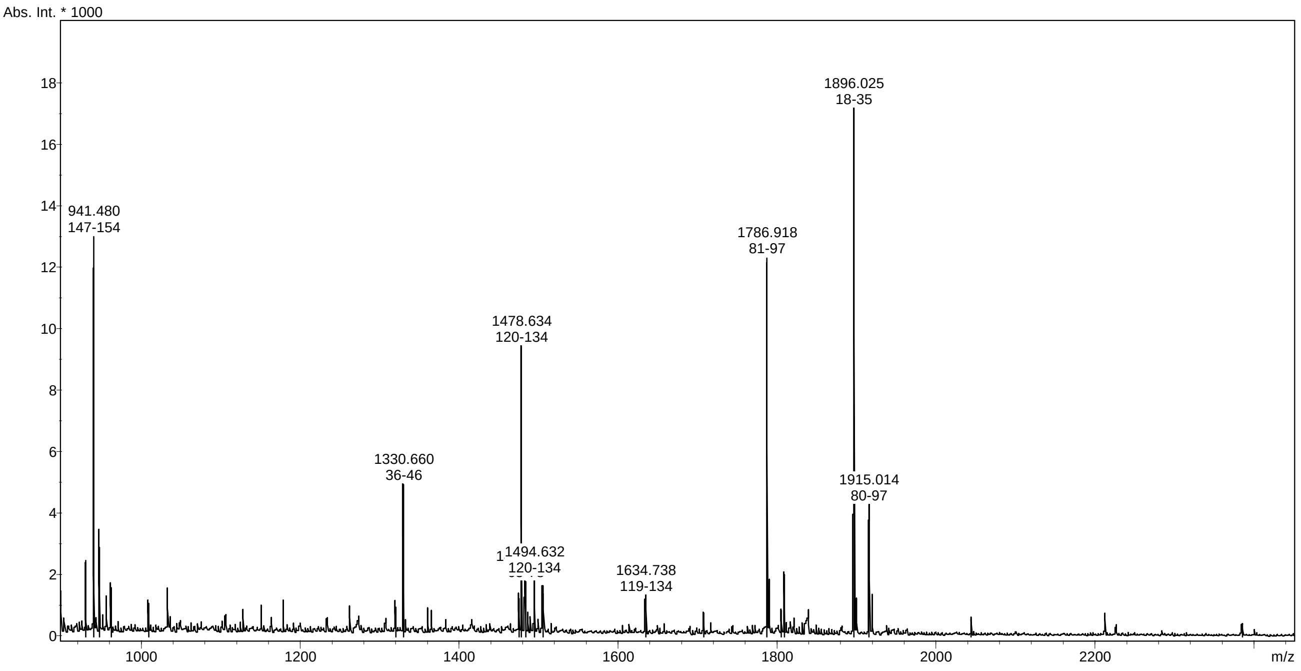

A representative MALDI-ToF PMF spectrum of spot 95

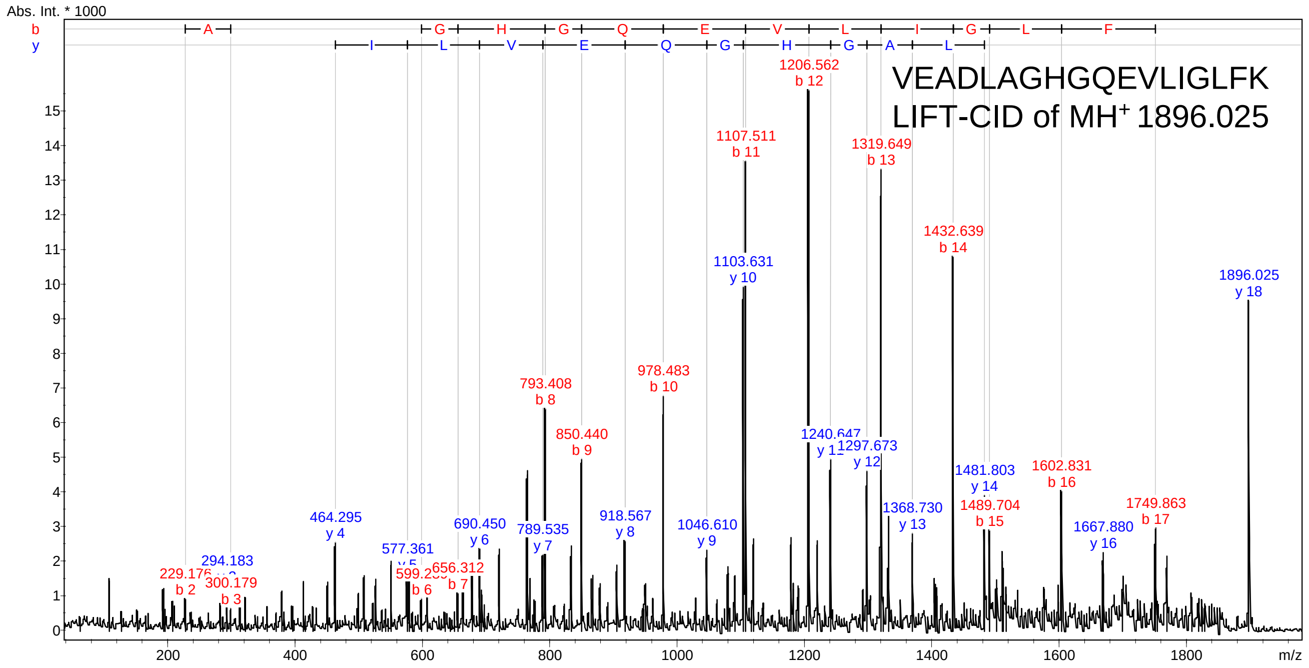

MSMS sequence analysis from the fragmentation of a precursor ion m/z 1896.025 by MALDI-ToF/ToF mass spectrometer

SPOT 96

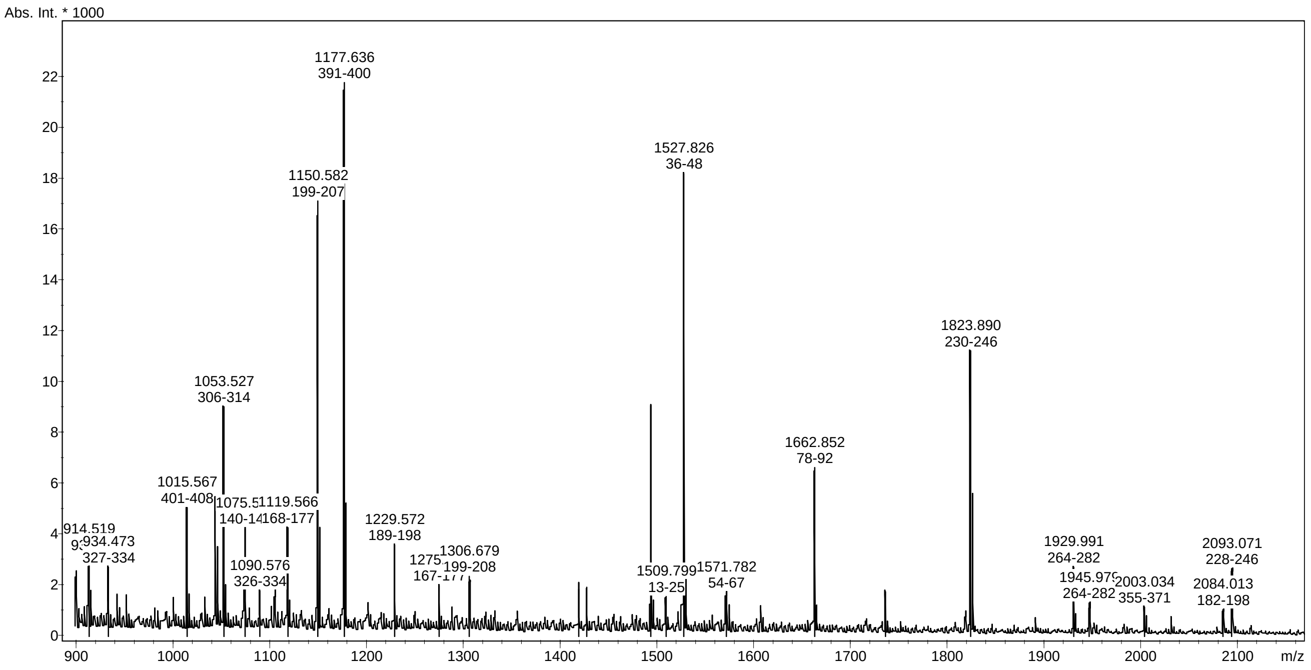

A representative MALDI-ToF PMF spectrum of spot 96

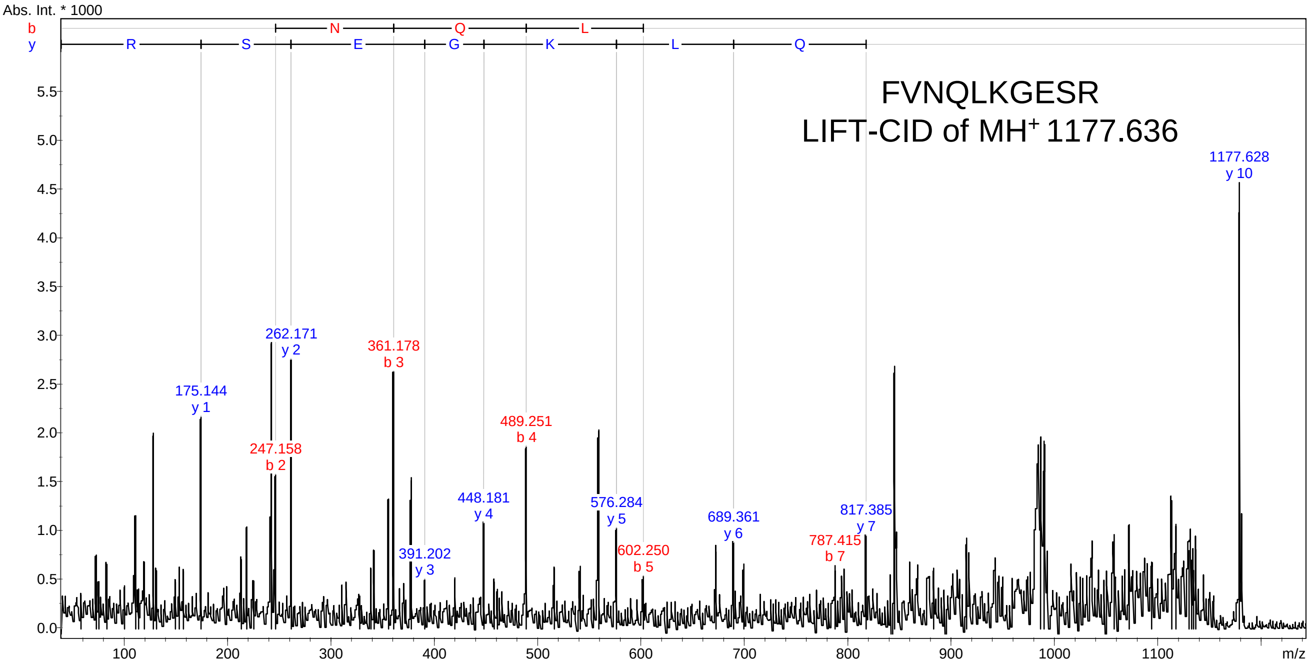

MSMS sequence analysis from the fragmentation of a precursor ion m/z 1177.636 by MALDI-ToF/ToF mass spectrometer

SPOT 97

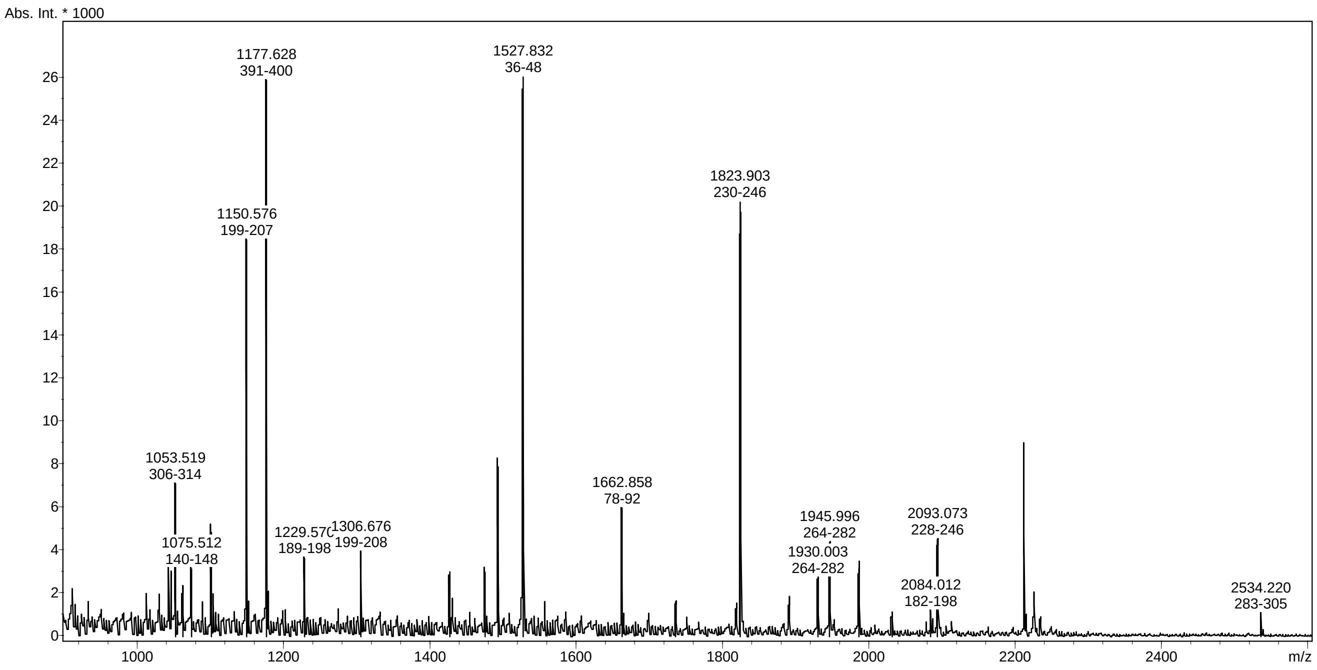

A representative MALDI-ToF PMF spectrum of spot 97

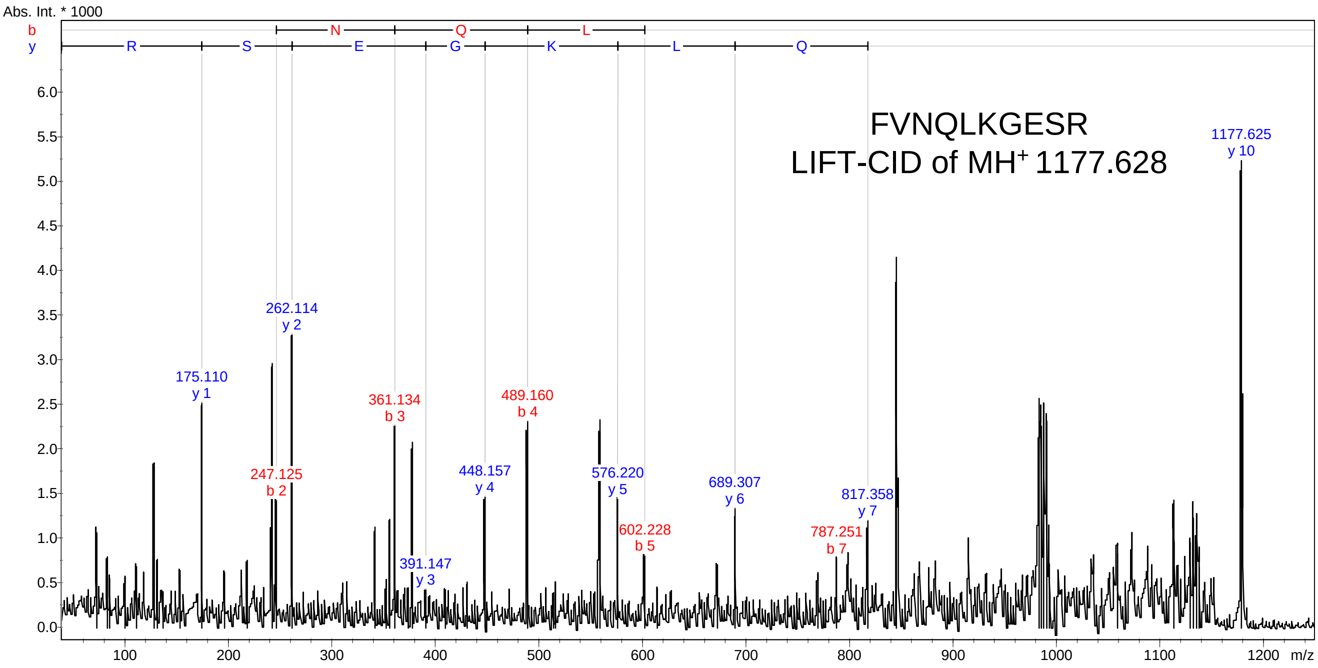

MSMS sequence analysis from the fragmentation of a precursor ion m/z 1177.628 by MALDI-ToF/ToF mass spectrometer

SPOT 98

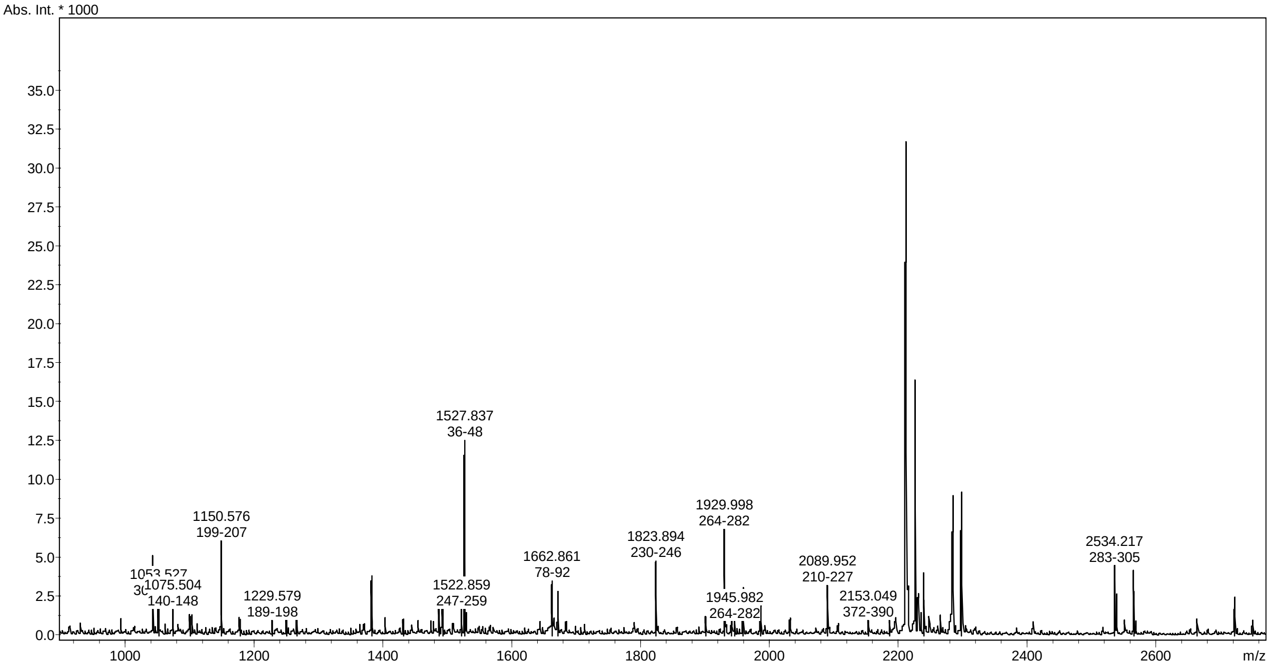

A representative MALDI-ToF PMF spectrum of spot 98

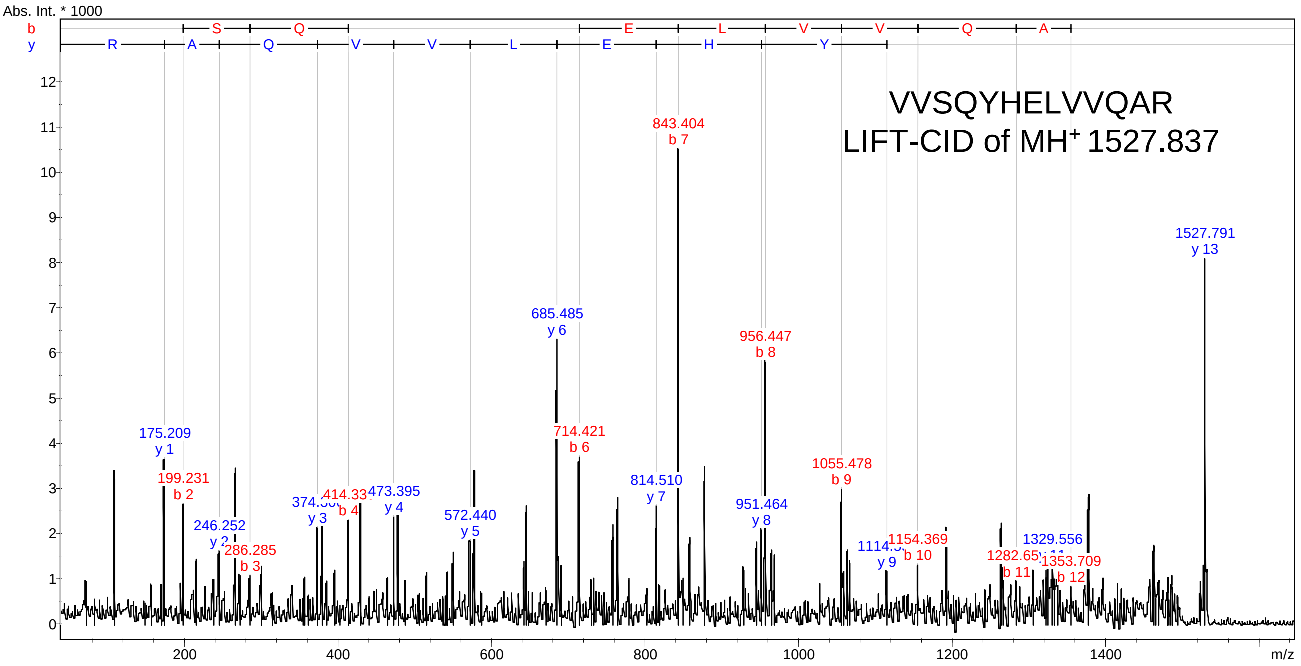

MSMS sequence analysis from the fragmentation of a precursor ion m/z 1527.837 by MALDI-ToF/ToF mass spectrometer

SPOT 99

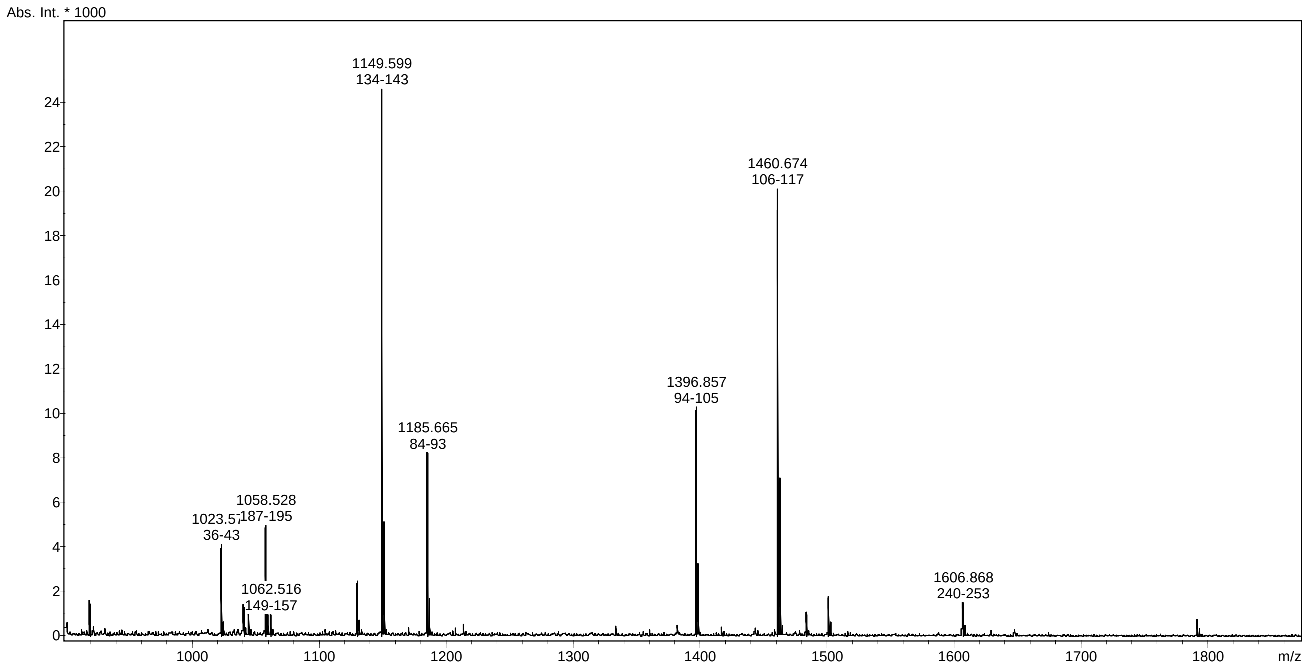

A representative MALDI-ToF PMF spectrum of spot 99

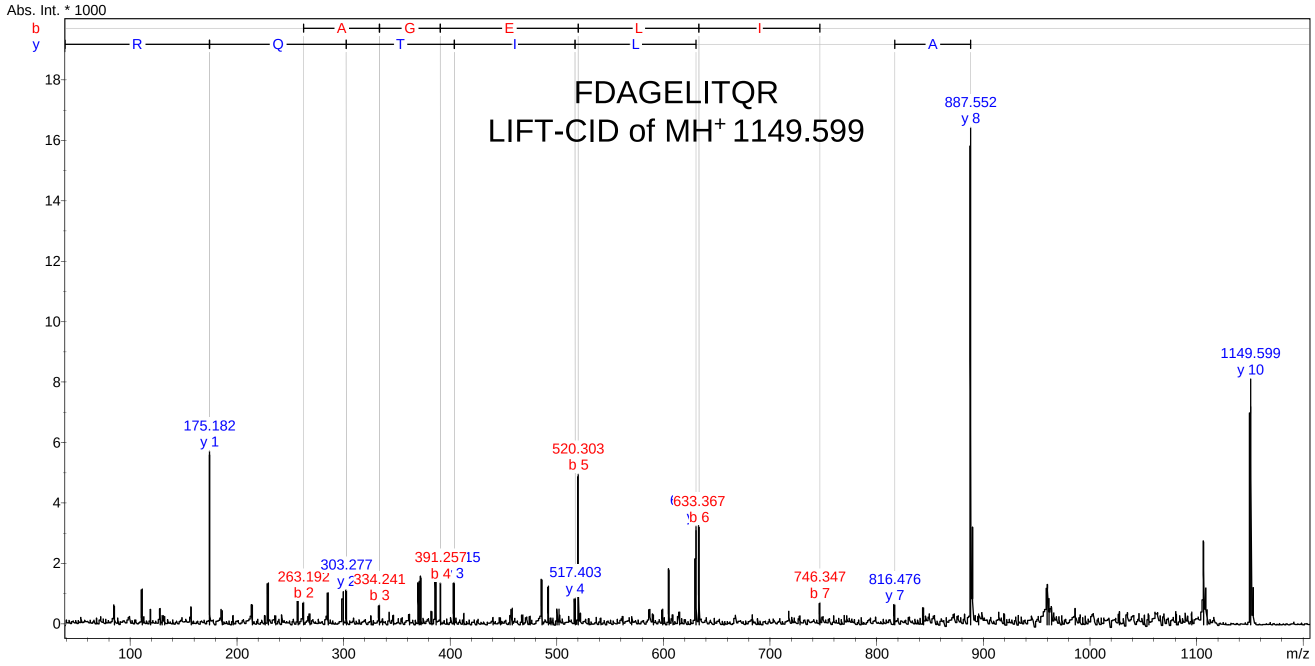

MSMS sequence analysis from the fragmentation of a precursor ion m/z 1149.599 by MALDI-ToF/ToF mass spectrometer

SPOT 100

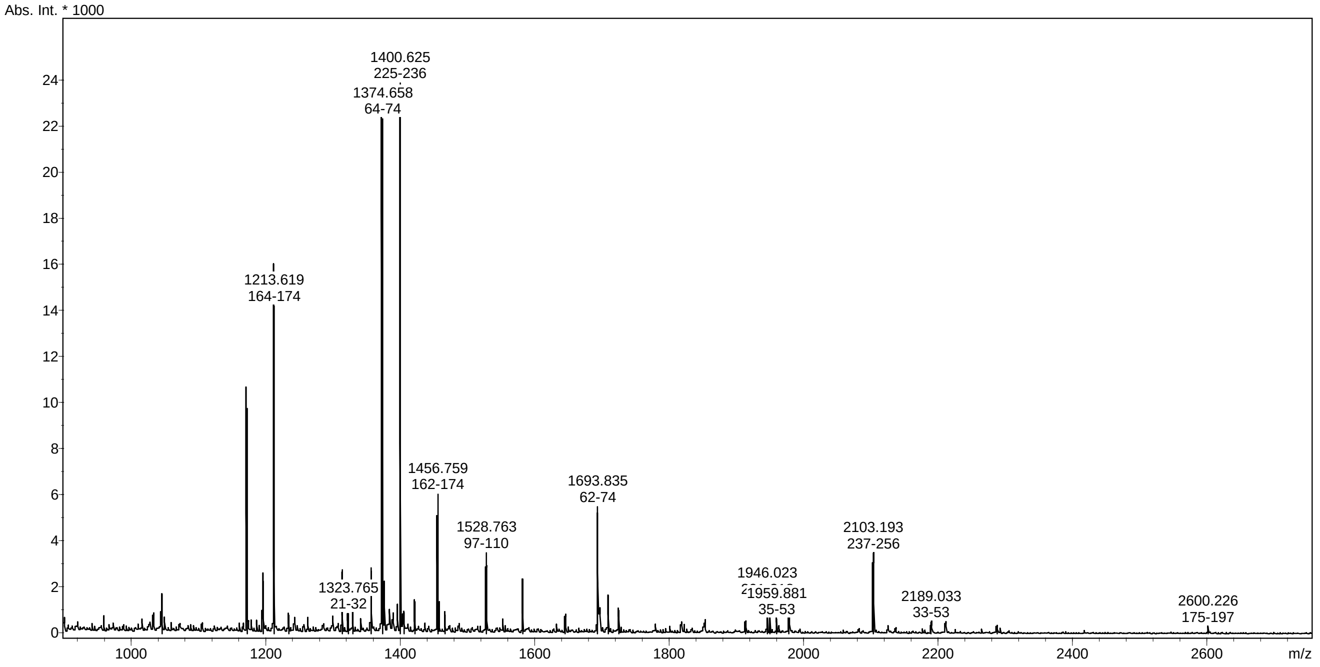

A representative MALDI-ToF PMF spectrum of spot 100

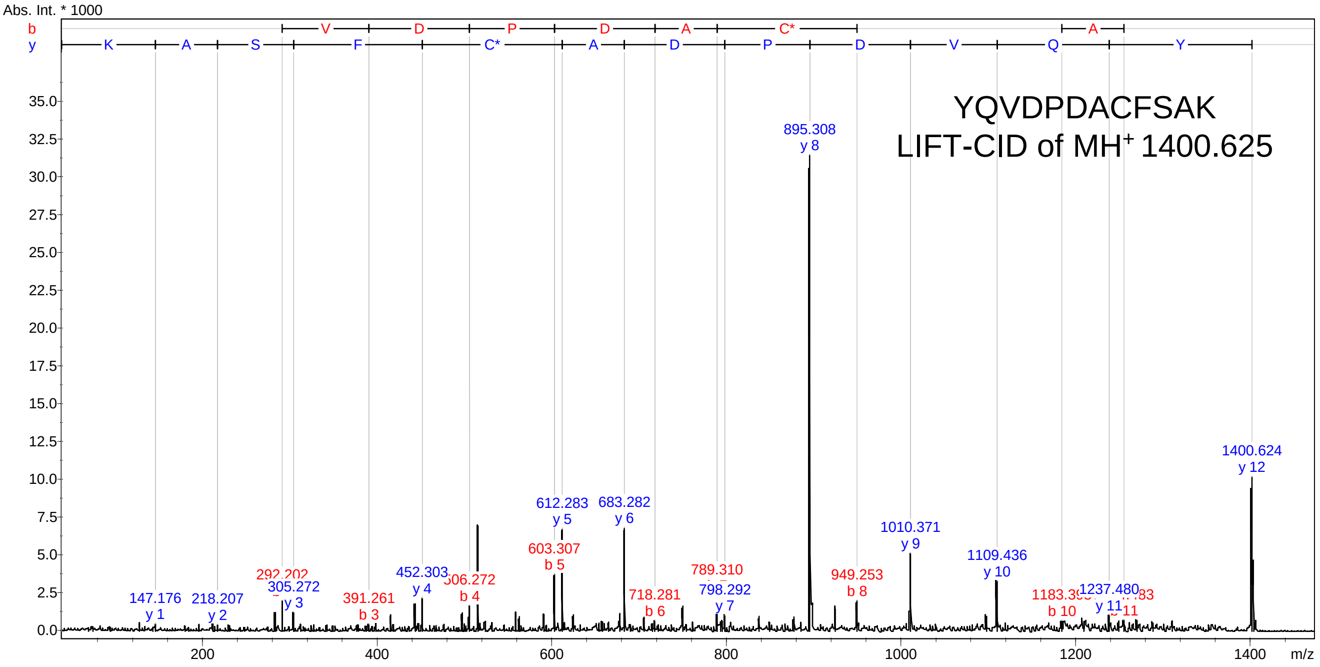

MSMS sequence analysis from the fragmentation of a precursor ion m/z 1400.625 by MALDI-ToF/ToF mass spectrometer

SPOT 101

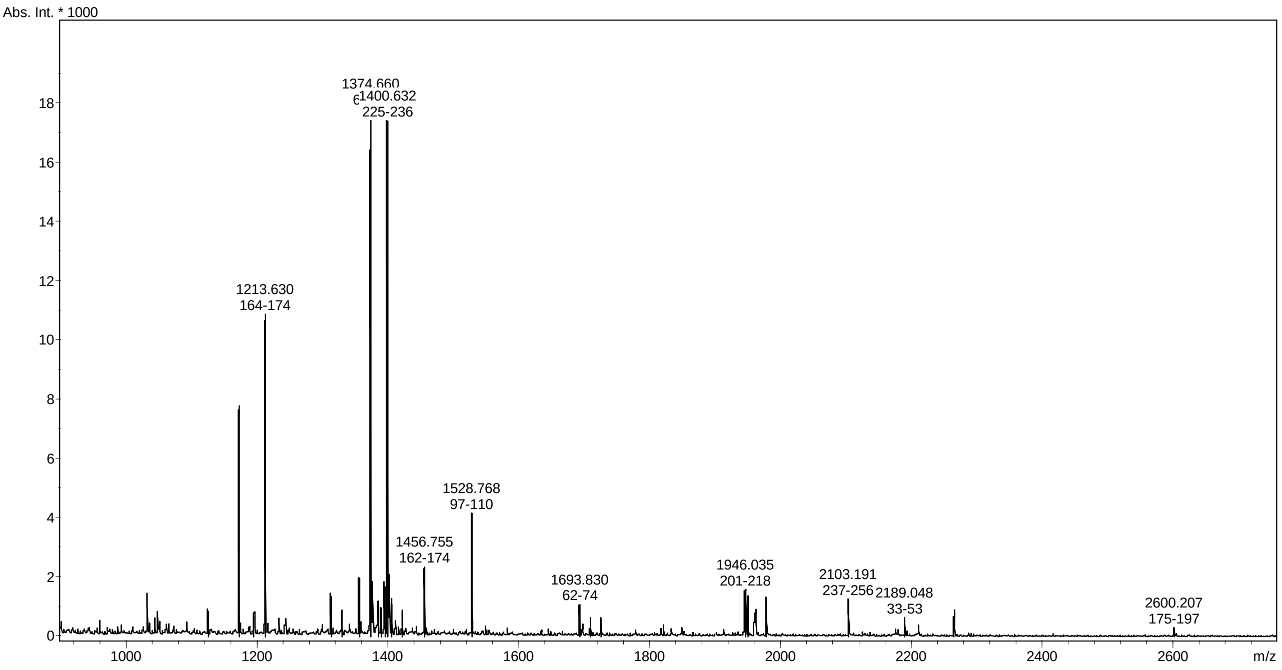

A representative MALDI-ToF PMF spectrum of spot 101

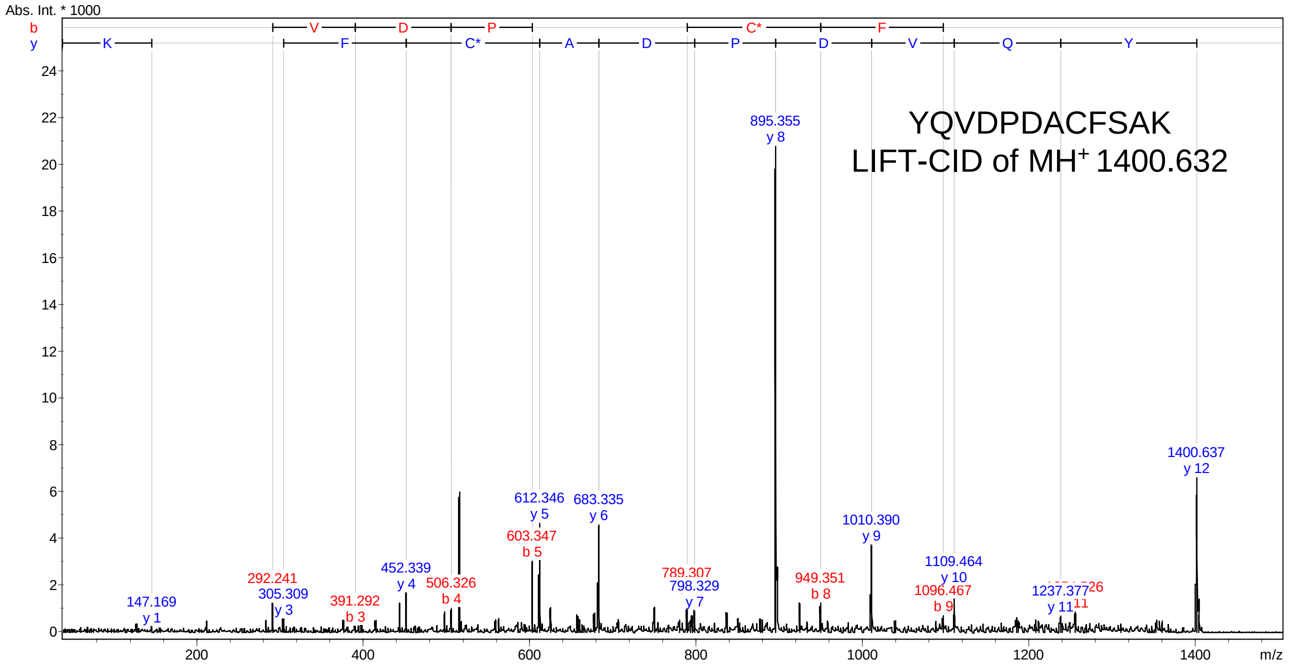

MSMS sequence analysis from the fragmentation of a precursor ion m/z 1400.632 by MALDI-ToF/ToF mass spectrometer

SPOT 102

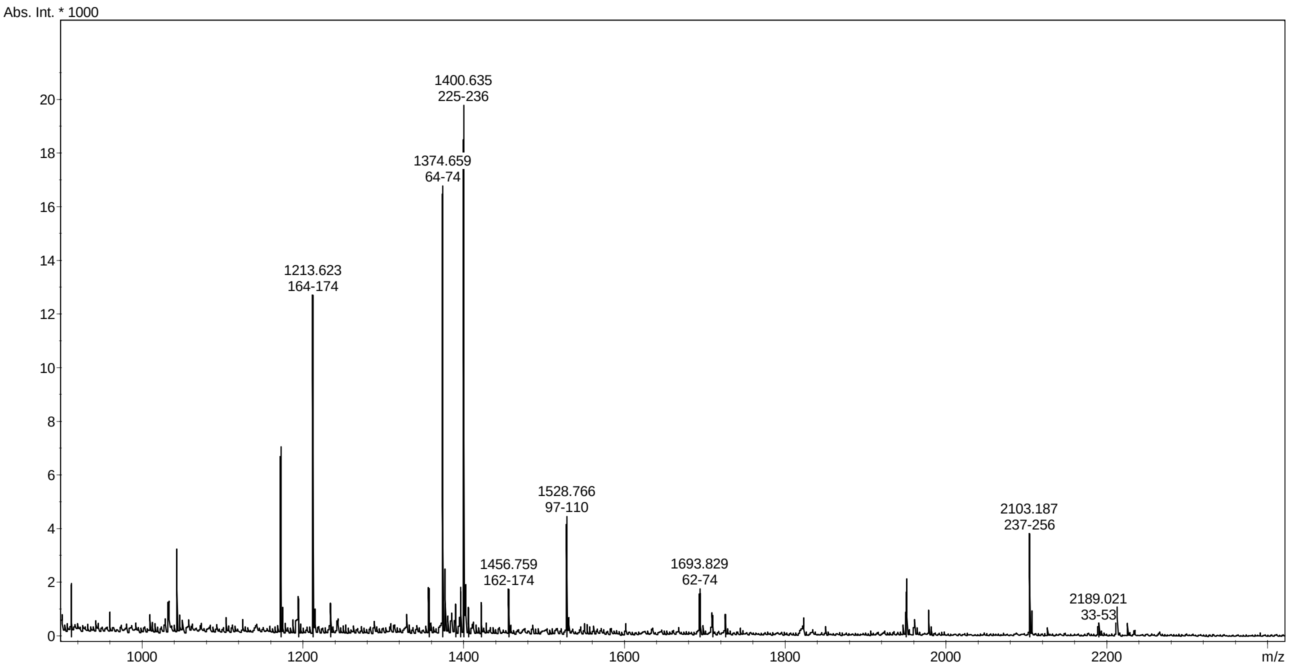

A representative MALDI-ToF PMF spectrum of spot 102

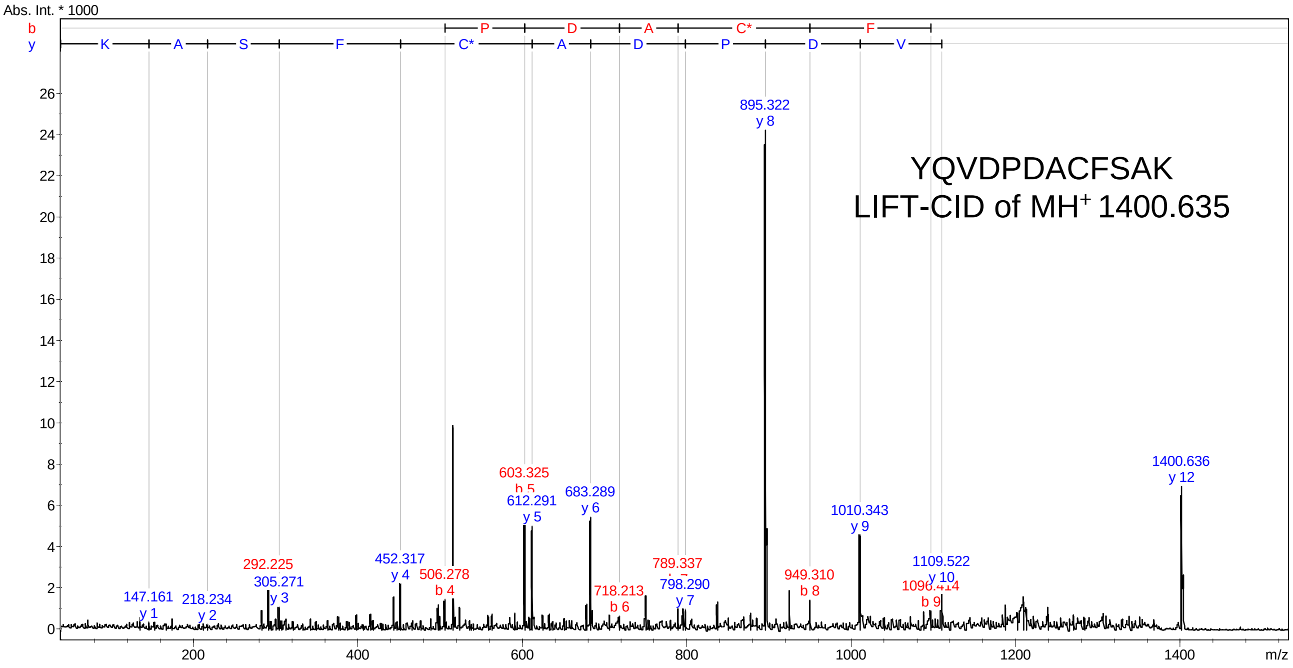

MSMS sequence analysis from the fragmentation of a precursor ion m/z 1400.635 by MALDI-ToF/ToF mass spectrometer

SPOT 104

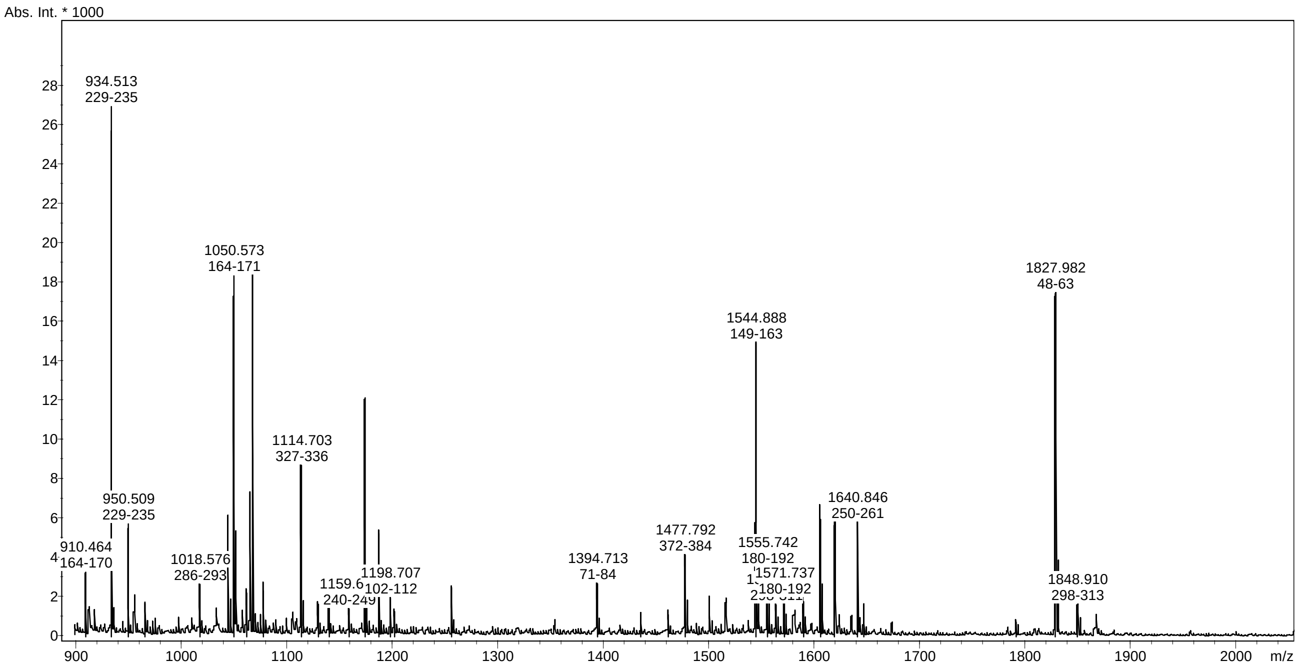

A representative MALDI-ToF PMF spectrum of spot 104

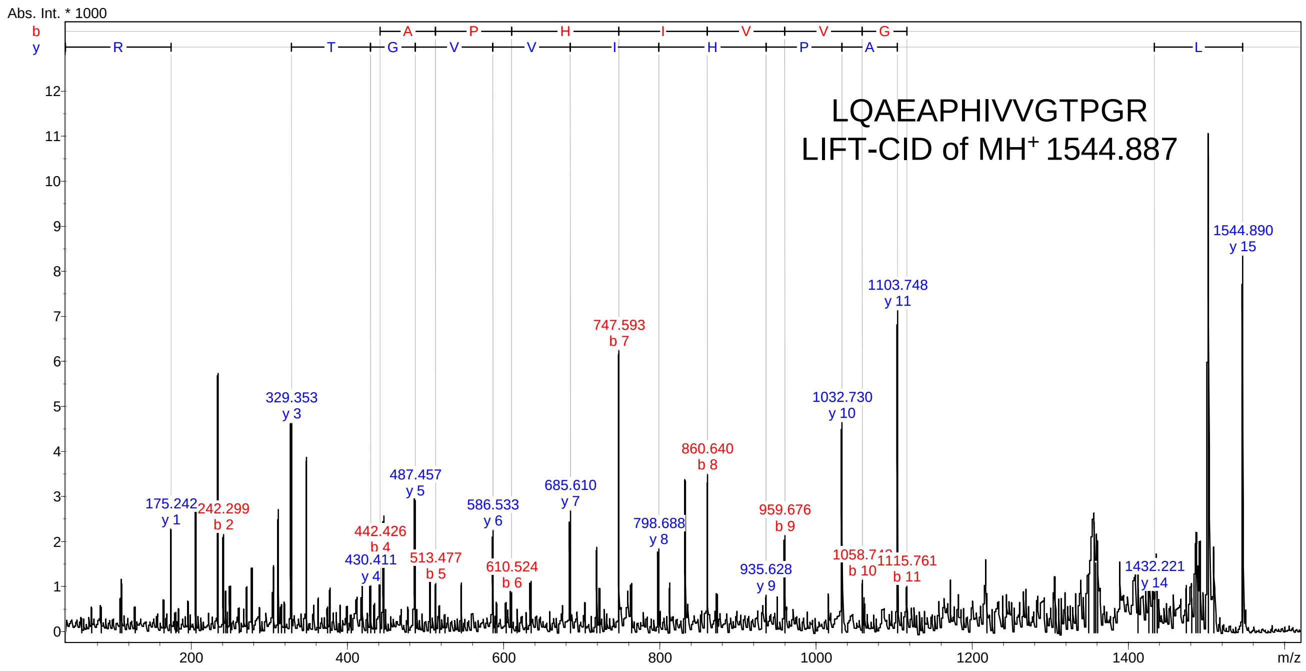

MSMS sequence analysis from the fragmentation of a precursor ion m/z 1544.887 by MALDI-ToF/ToF mass spectrometer

SPOT 106

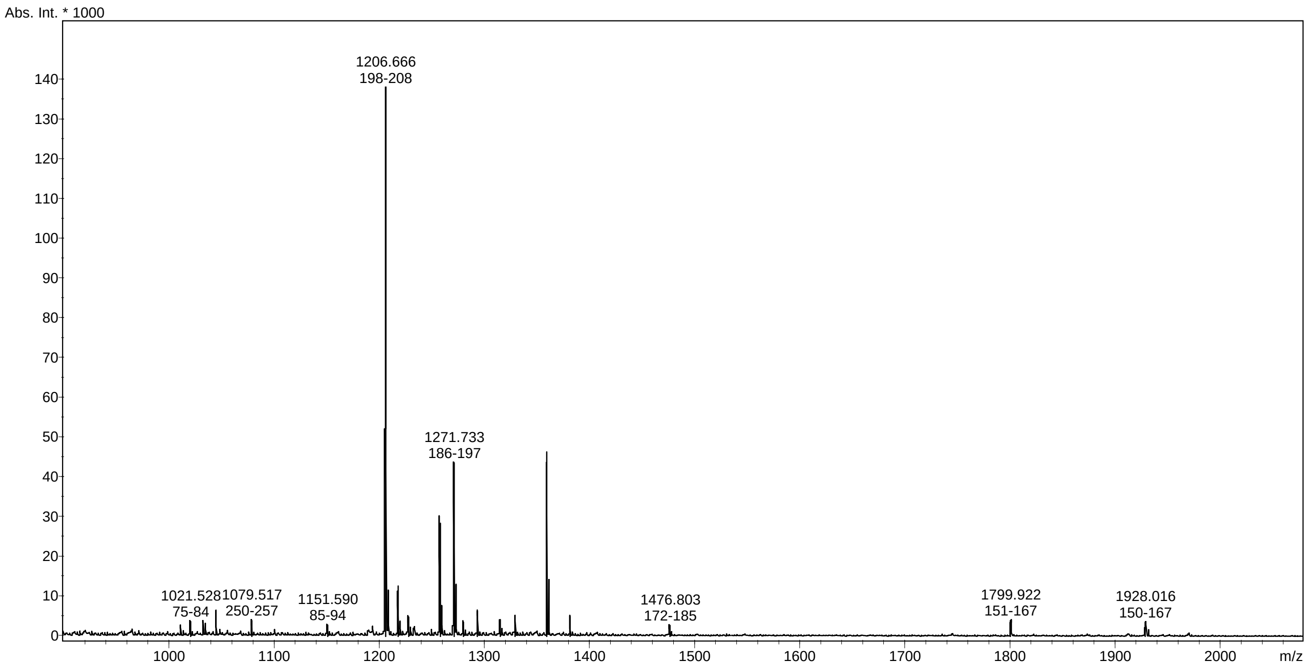

A representative MALDI-ToF PMF spectrum of spot 106

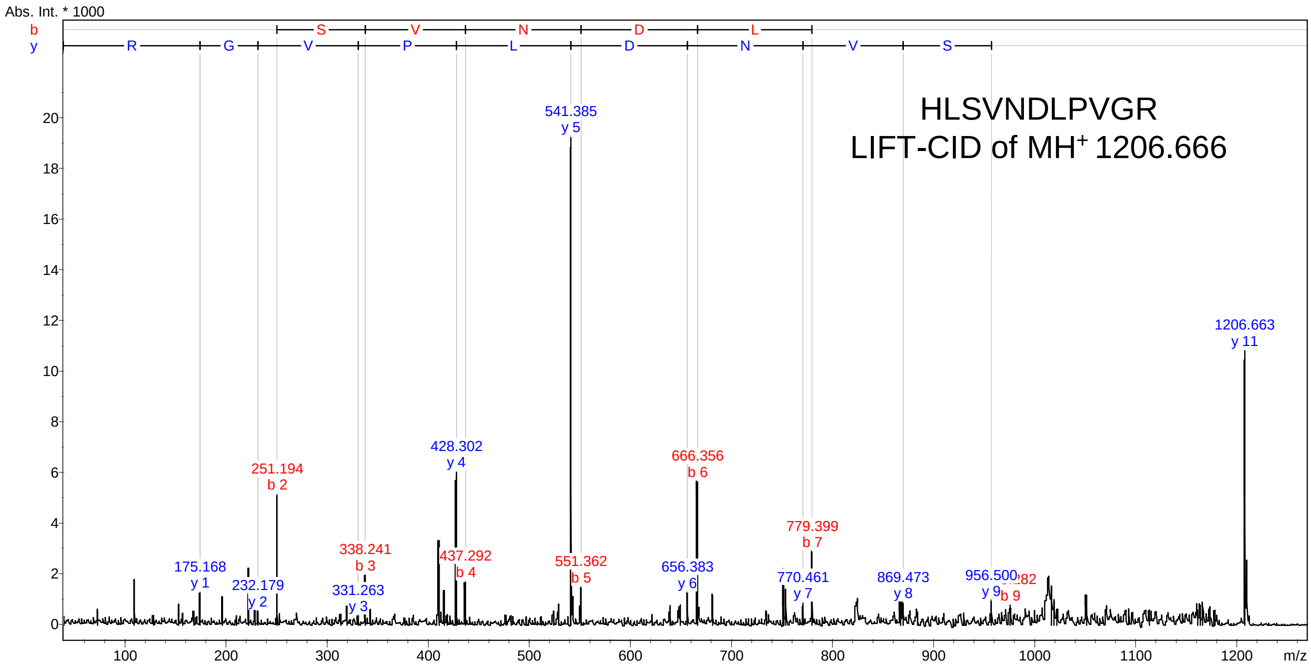

MSMS sequence analysis from the fragmentation of a precursor ion m/z 1206.666 by MALDI-ToF/ToF mass spectrometer

SPOT 107

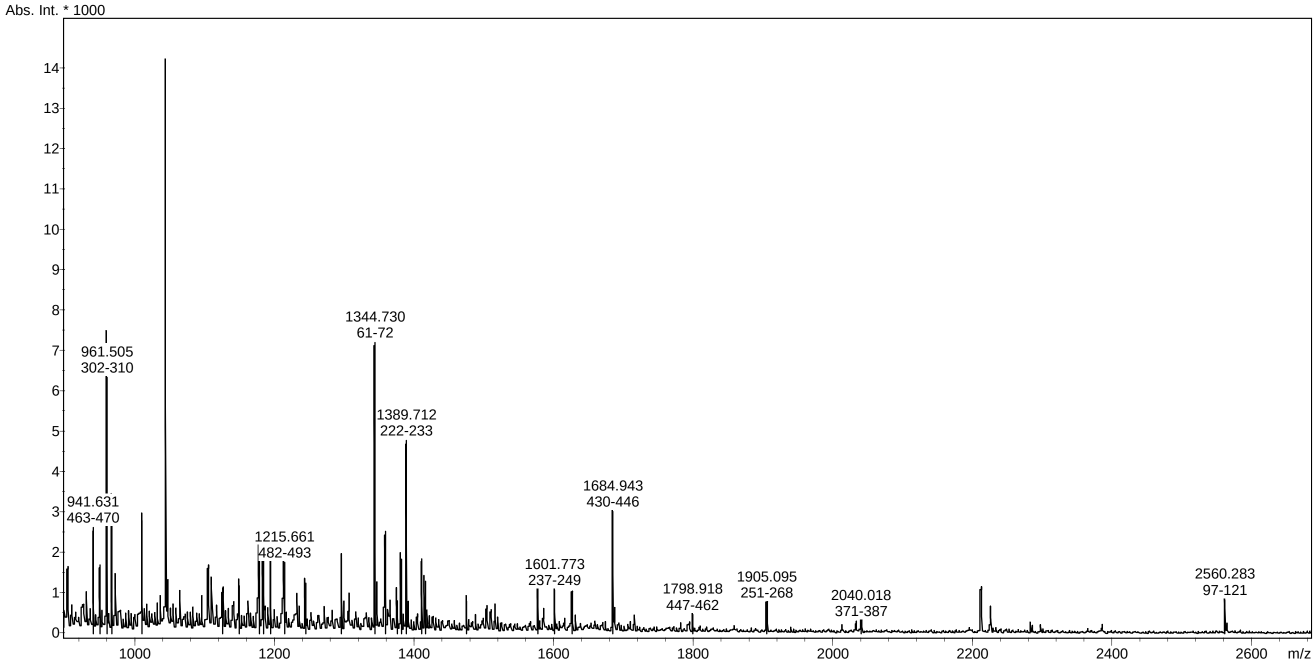

A representative MALDI-ToF PMF spectrum of spot 107

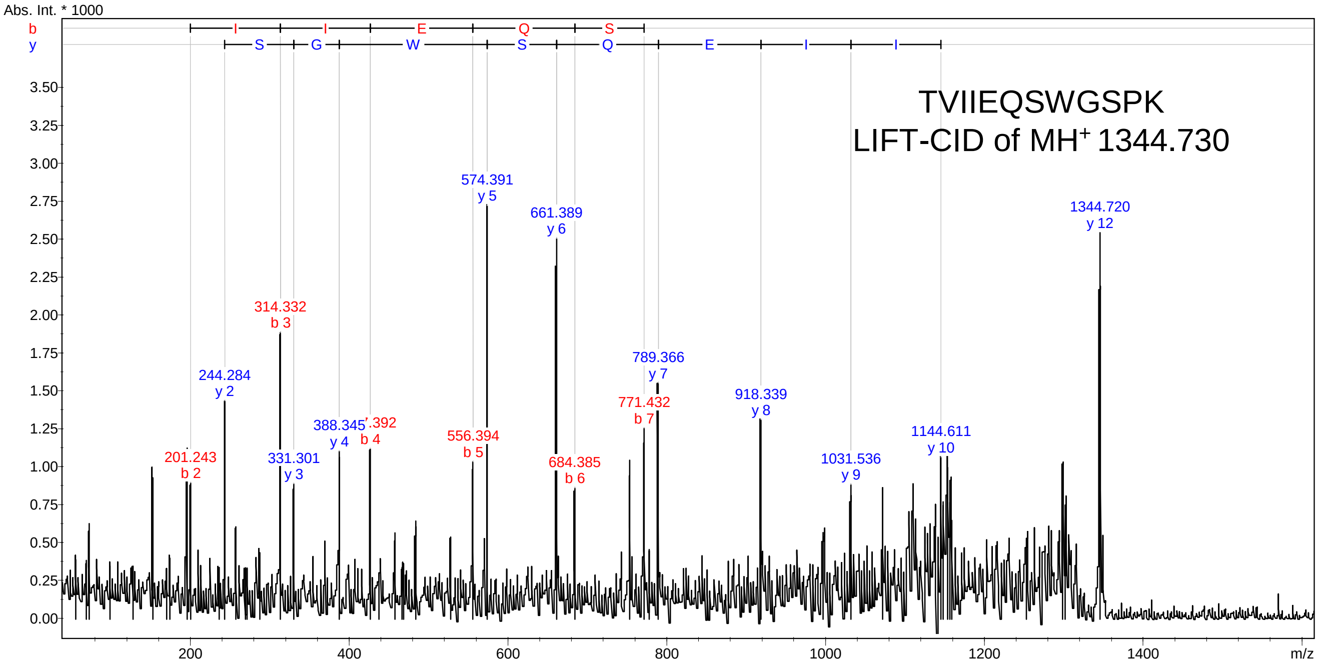

MSMS sequence analysis from the fragmentation of a precursor ion m/z 1344.730 by MALDI-ToF/ToF mass spectrometer

SPOT 108

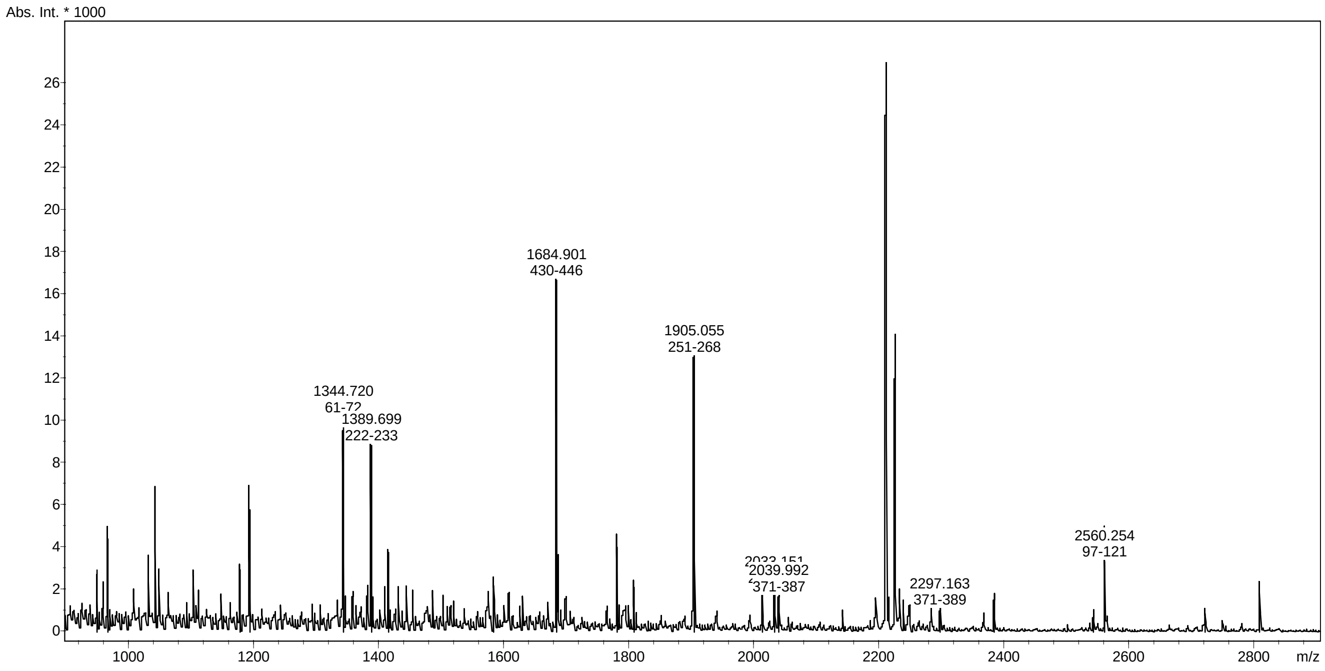

A representative MALDI-ToF PMF spectrum of spot 108

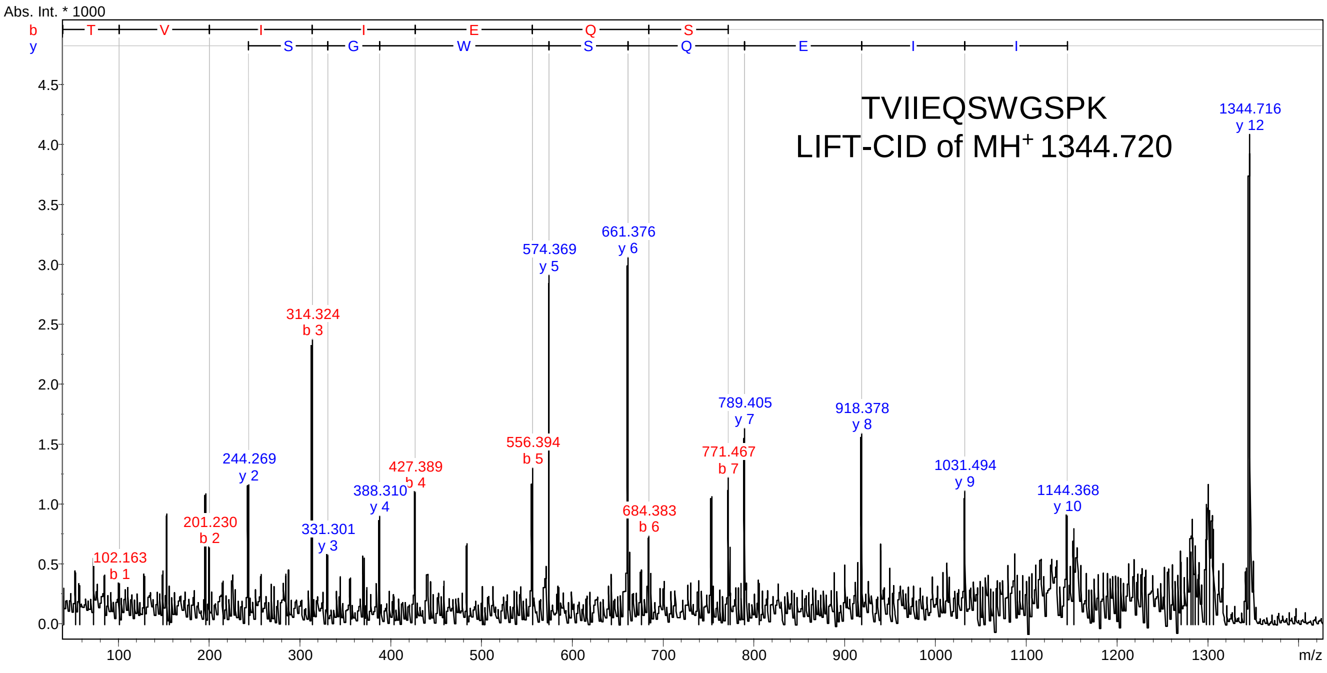

MSMS sequence analysis from the fragmentation of a precursor ion m/z 1344.720 by MALDI-ToF/ToF mass spectrometer

SPOT 109

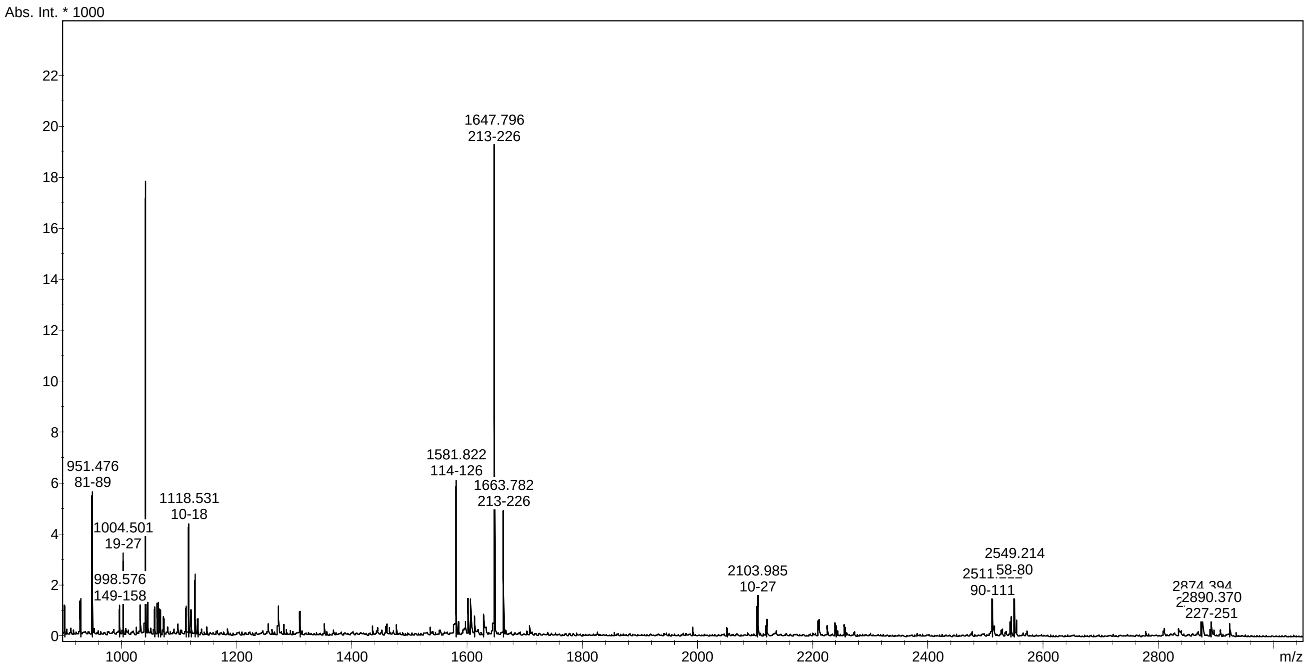

A representative MALDI-ToF PMF spectrum of spot 109

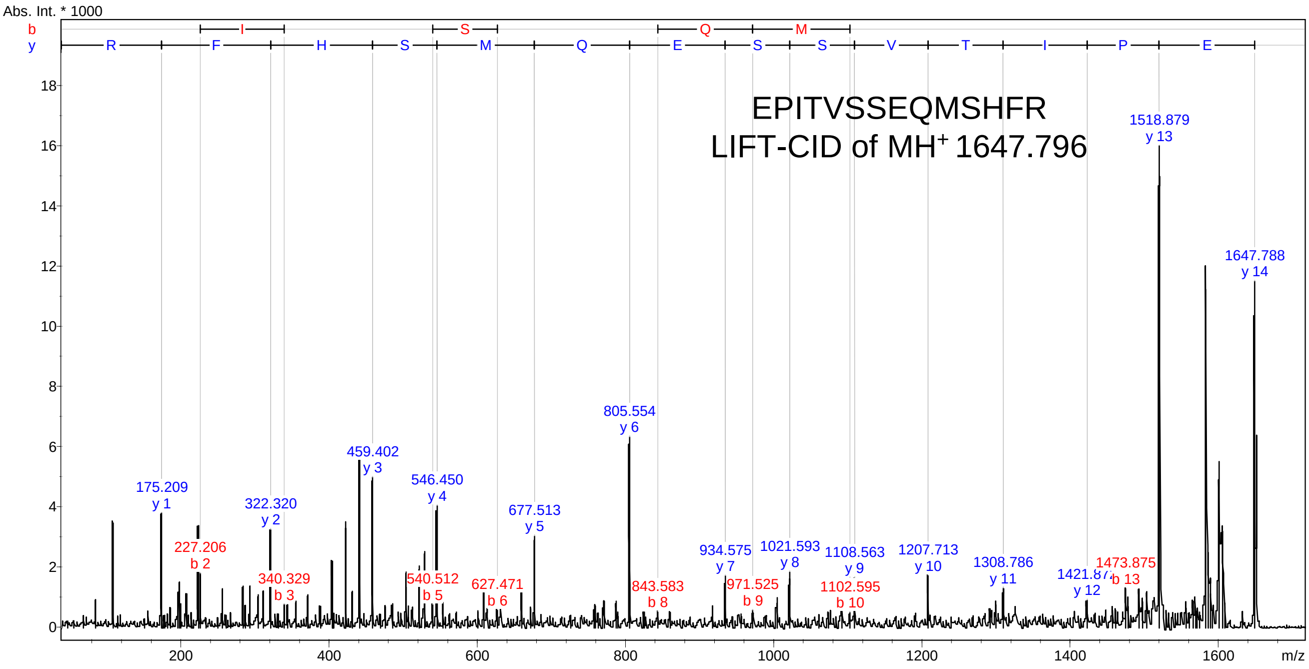

MSMS sequence analysis from the fragmentation of a precursor ion m/z 1647.796 by MALDI-ToF/ToF mass spectrometer

SPOT 110

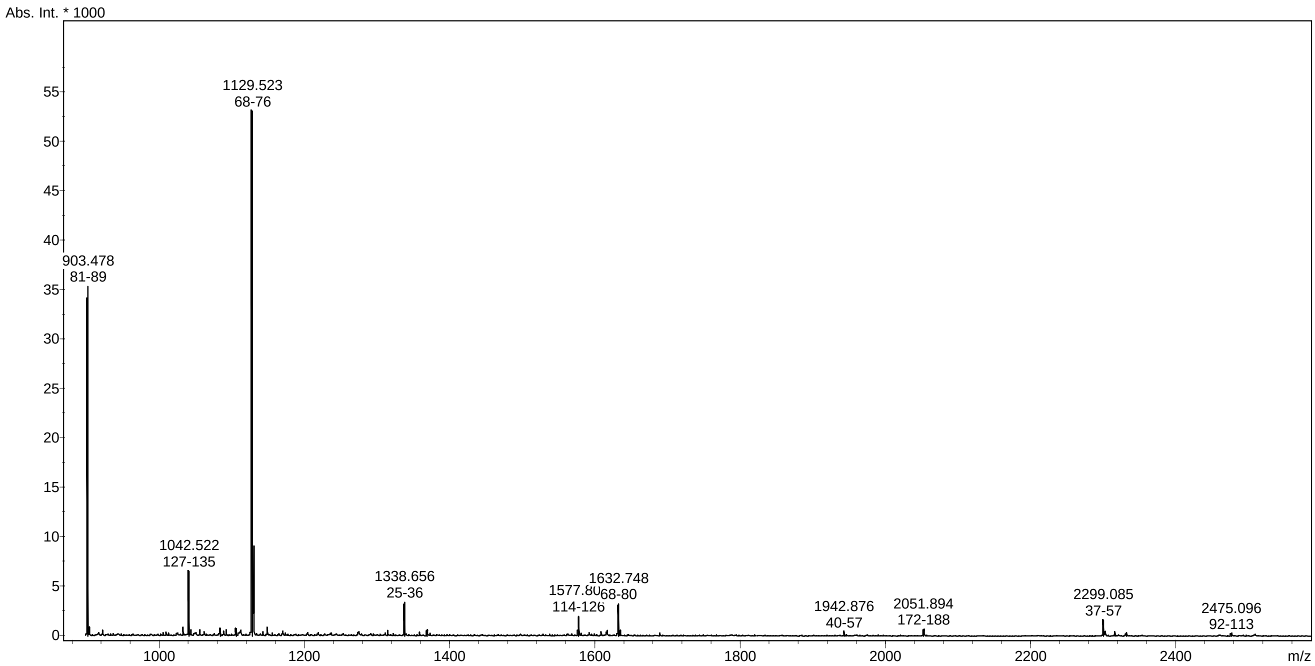

A representative MALDI-ToF PMF spectrum of spot 110

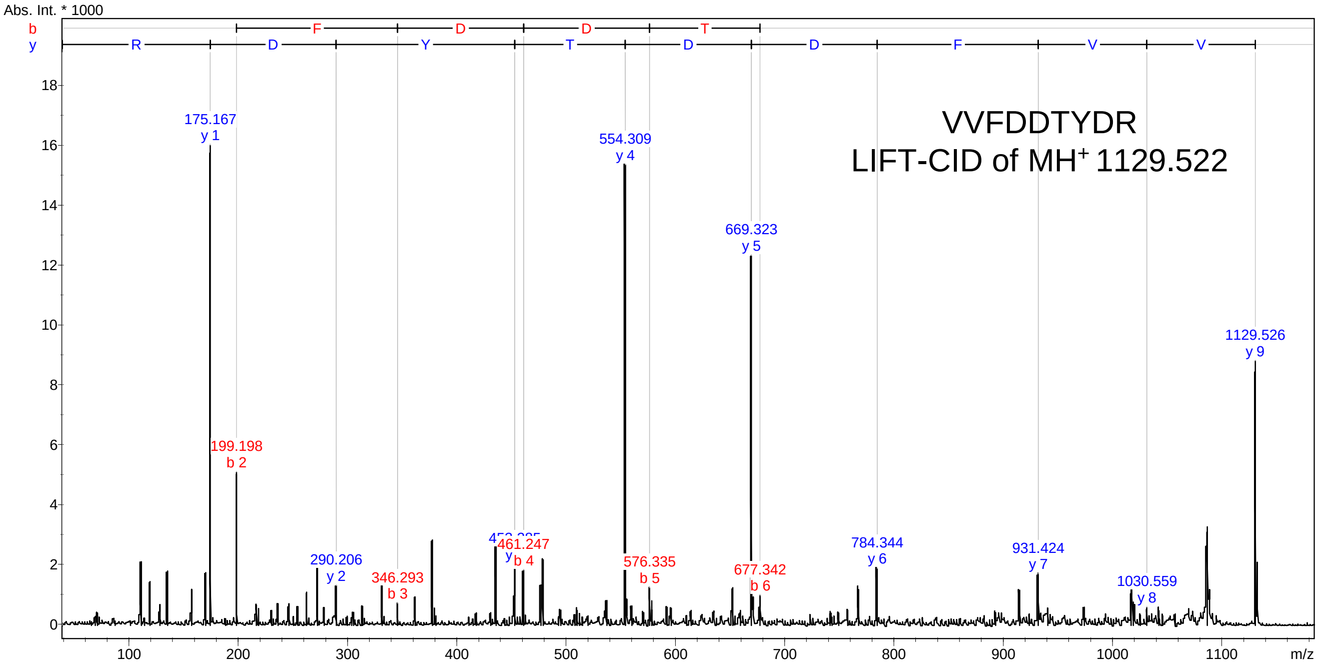

MSMS sequence analysis from the fragmentation of a precursor ion m/z 1129.522 by MALDI-ToF/ToF mass spectrometer

SPOT 111

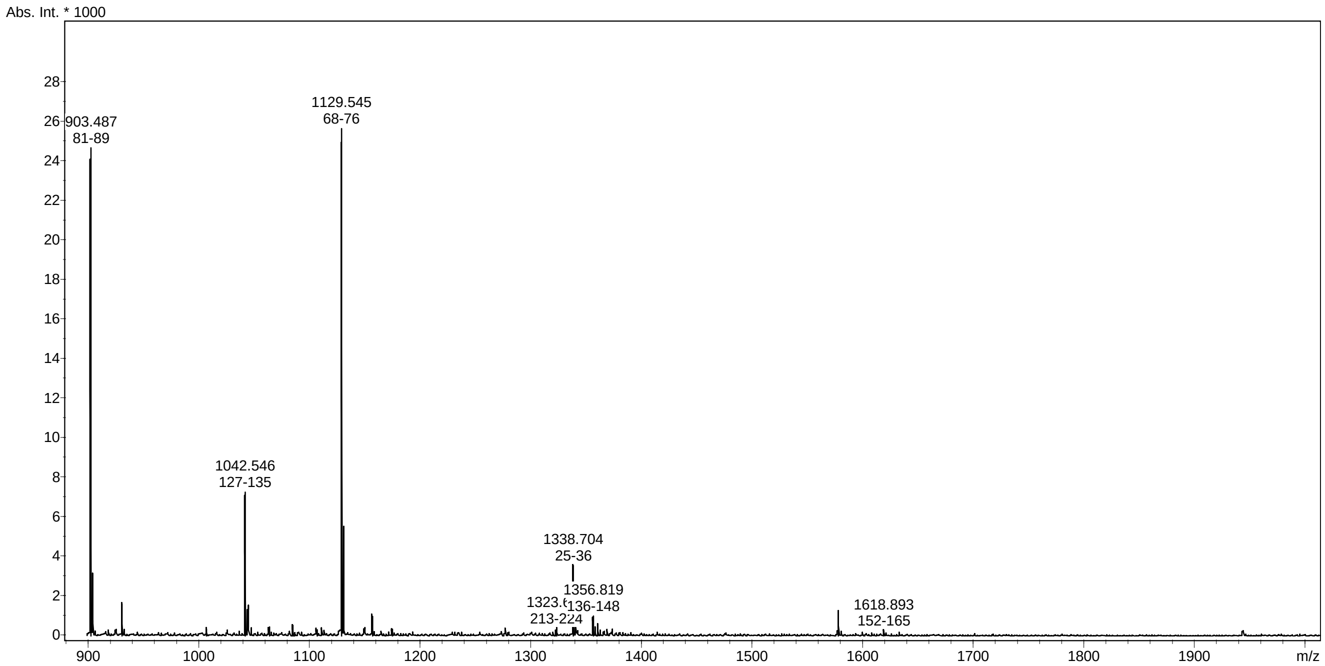

A representative MALDI-ToF PMF spectrum of spot 111

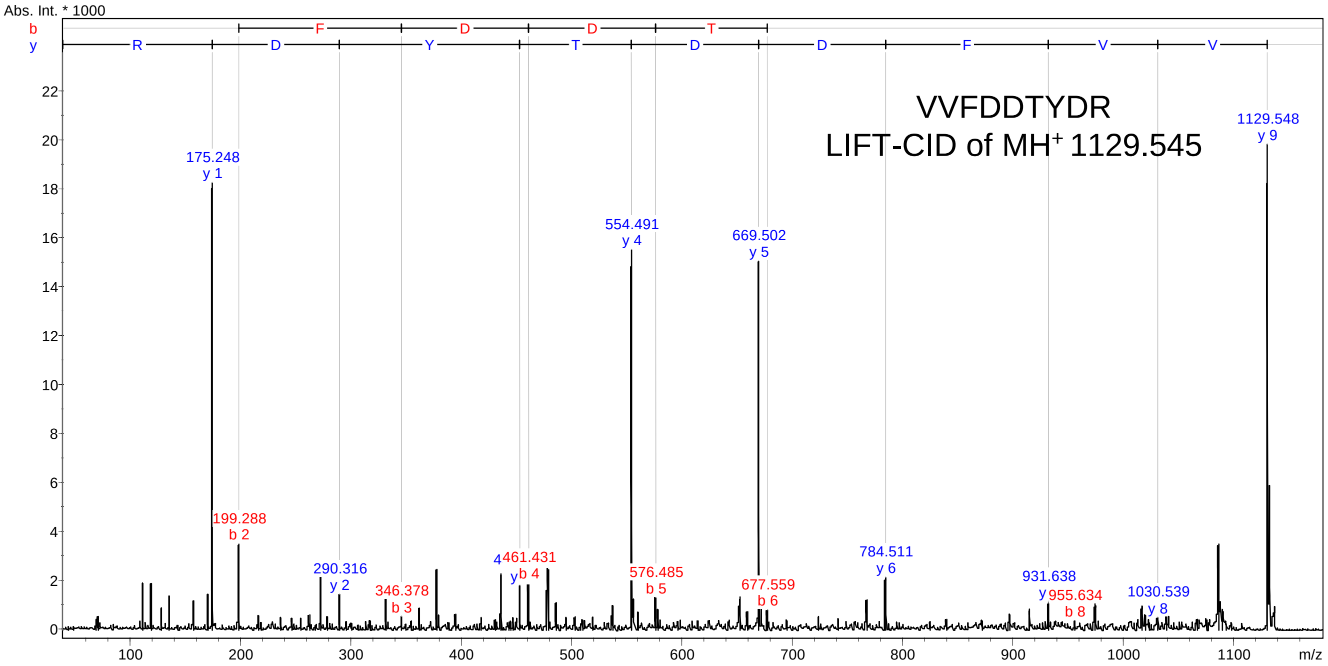

MSMS sequence analysis from the fragmentation of a precursor ion m/z 1129.545 by MALDI-ToF/ToF mass spectrometer

SPOT 112

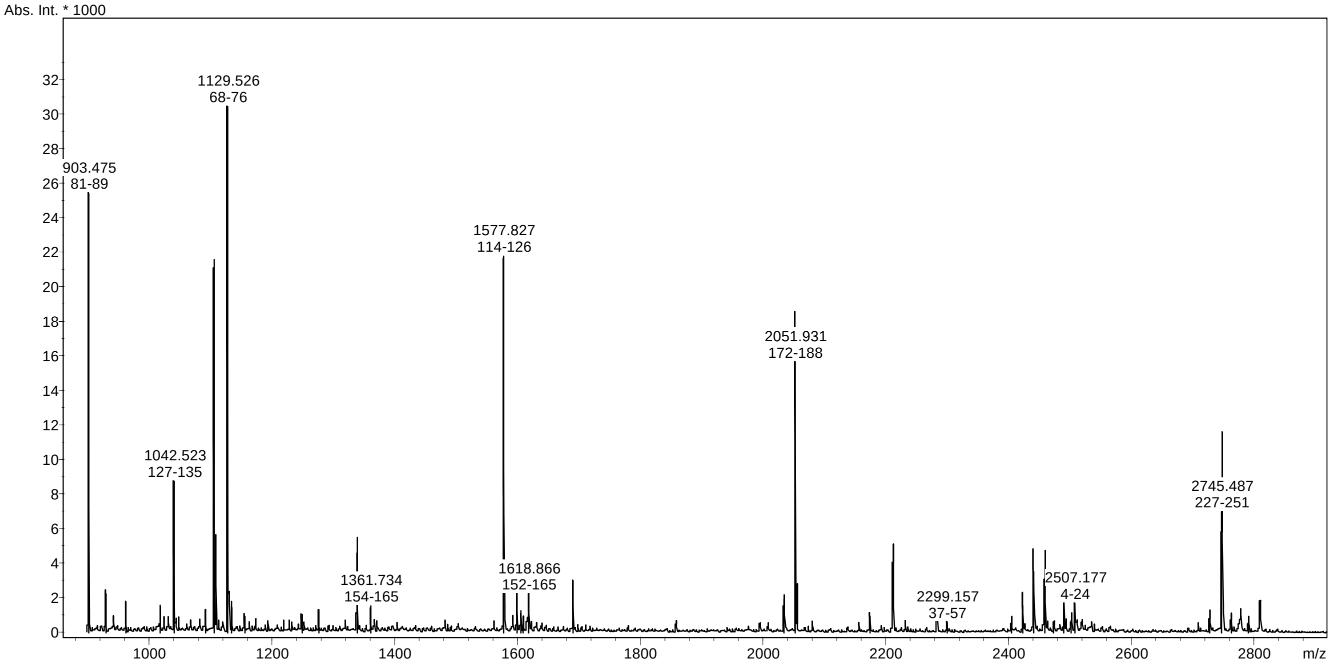

A representative MALDI-ToF PMF spectrum of spot 112

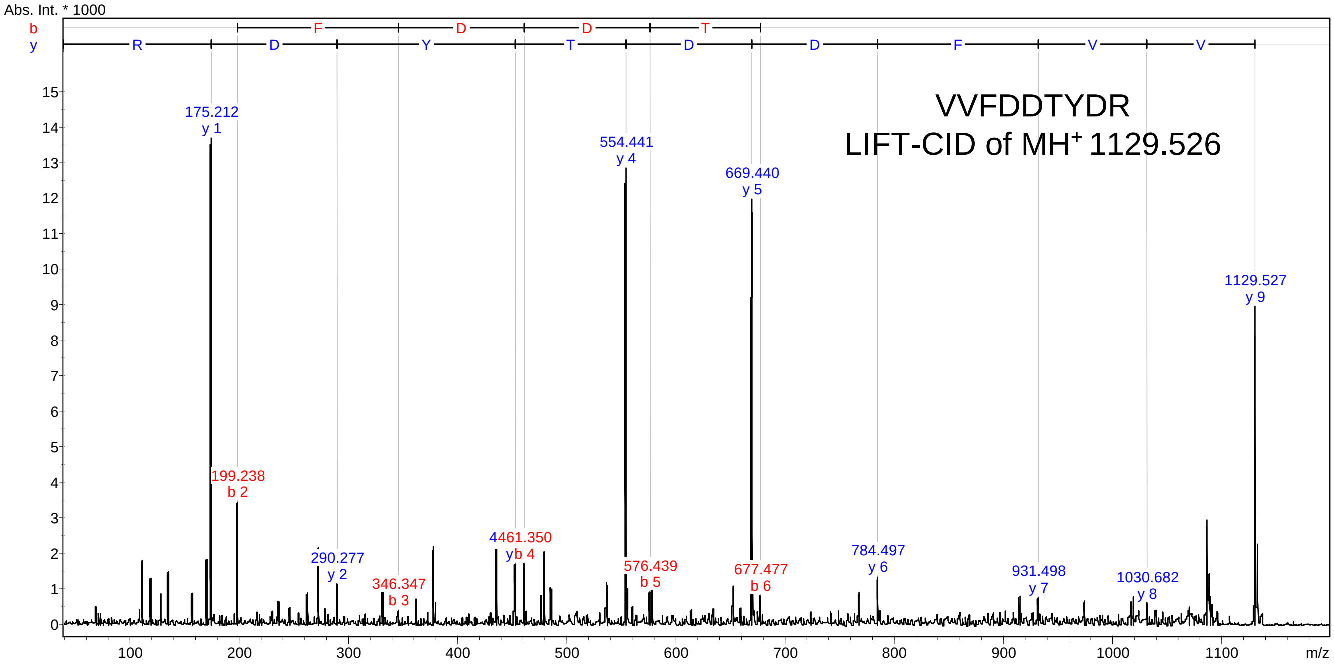

MSMS sequence analysis from the fragmentation of a precursor ion m/z 1129.526 by MALDI-ToF/ToF mass spectrometer

SPOT 113

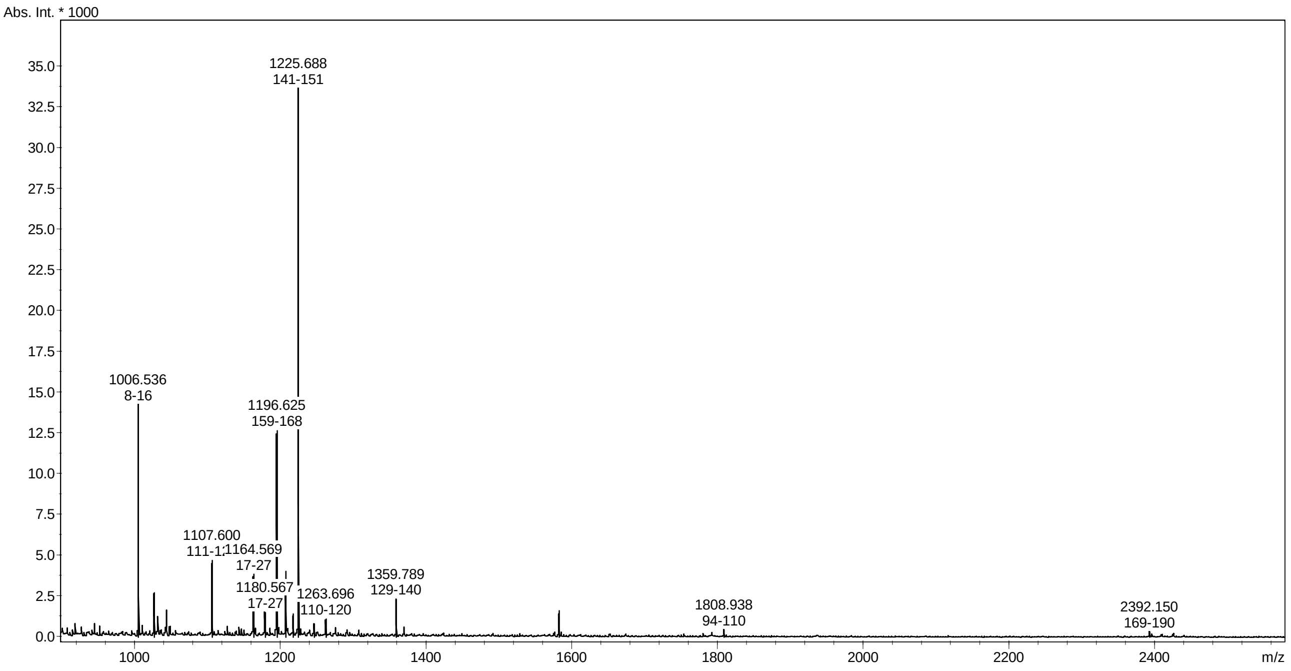

A representative MALDI-ToF PMF spectrum of spot 113

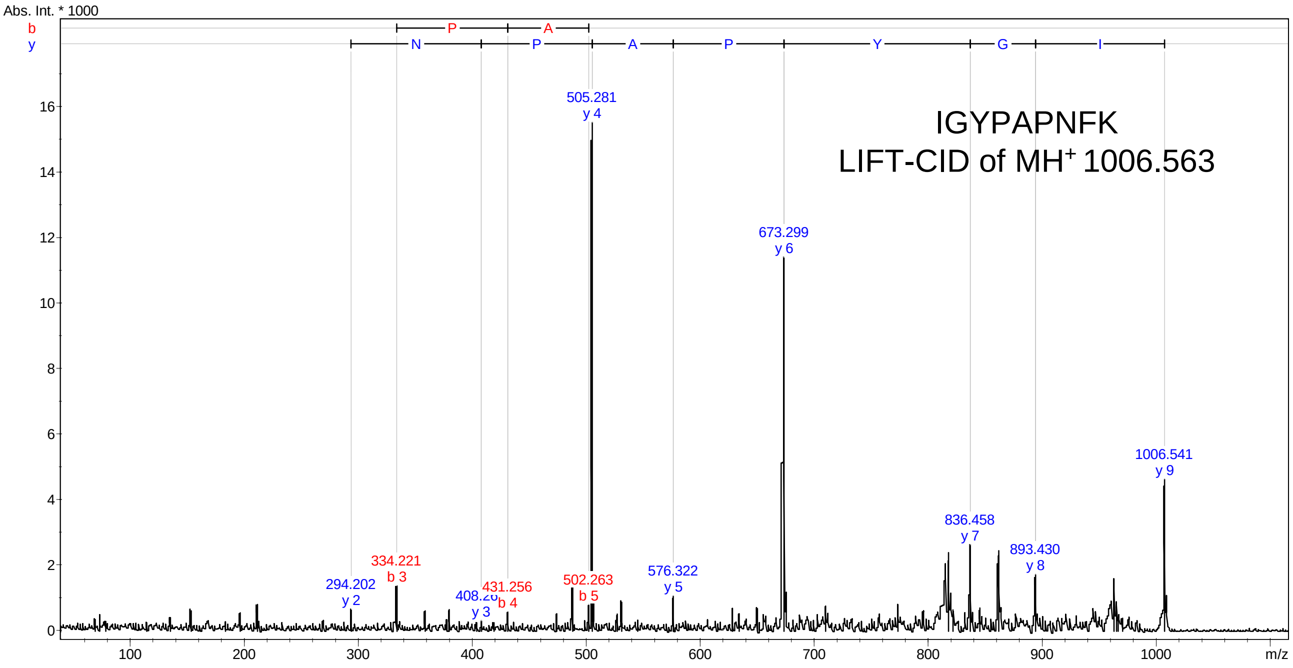

MSMS sequence analysis from the fragmentation of a precursor ion m/z 1006.563 by MALDI-ToF/ToF mass spectrometer

SPOT 114

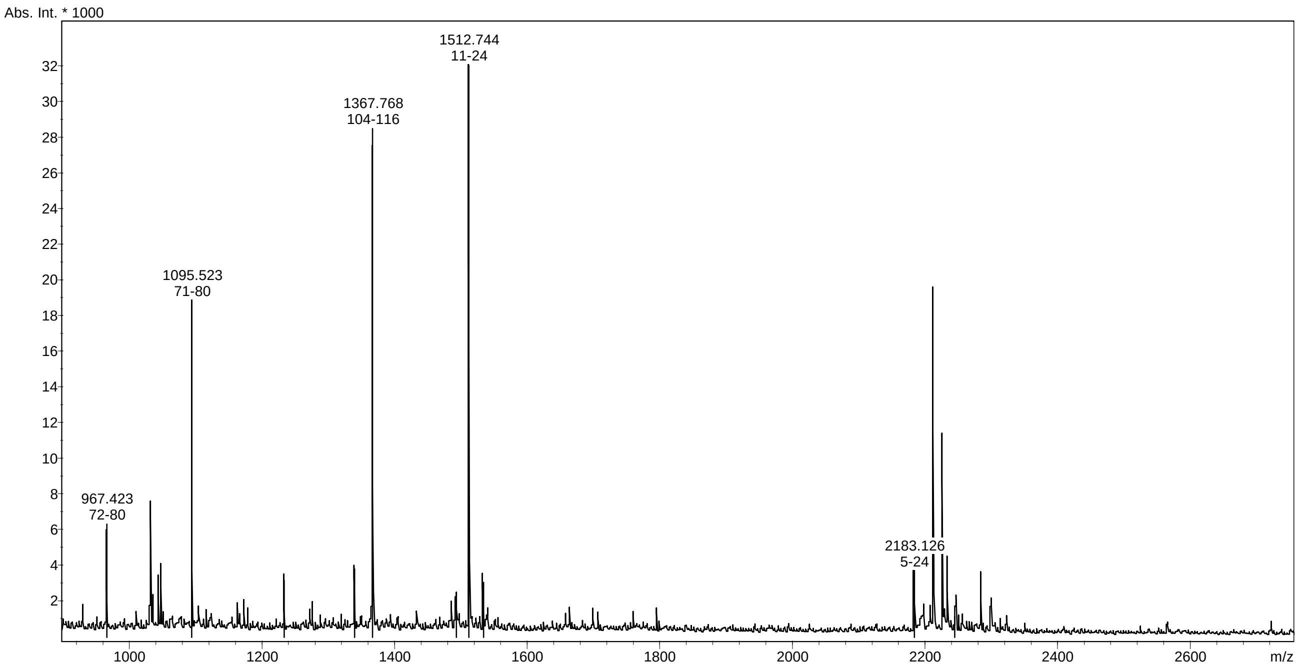

A representative MALDI-ToF PMF spectrum of spot 114

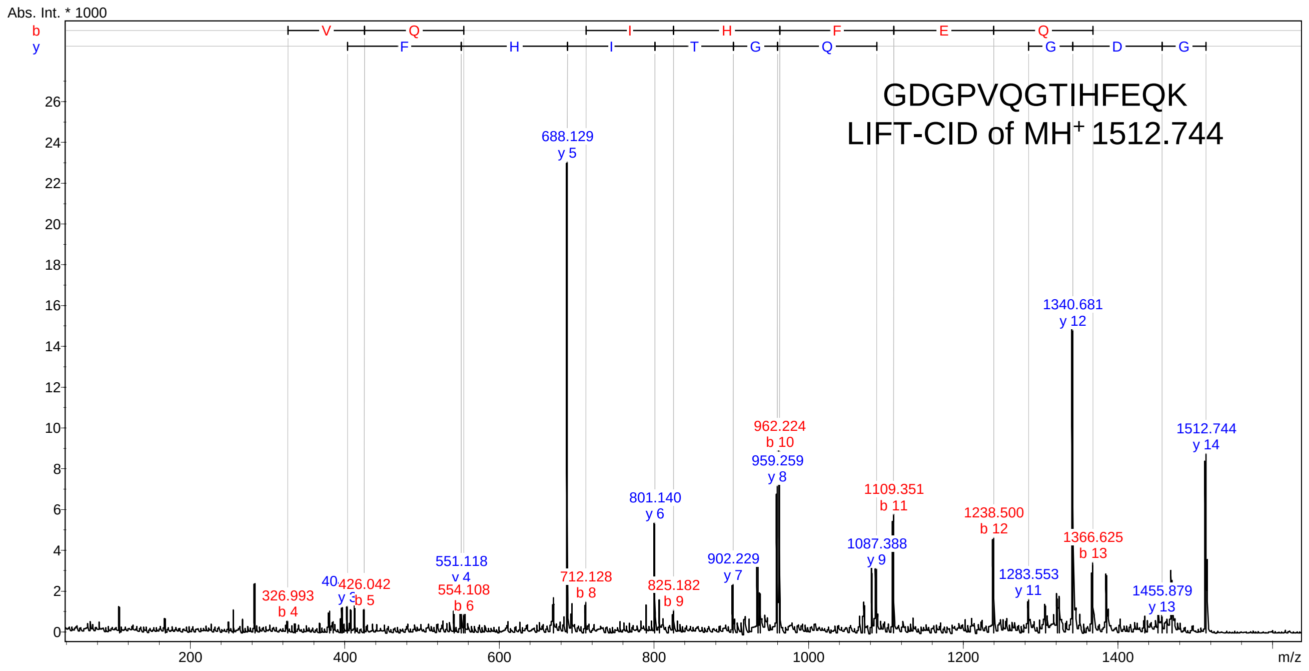

MSMS sequence analysis from the fragmentation of a precursor ion m/z 1512.744 by MALDI-ToF/ToF mass spectrometer

SPOT 115

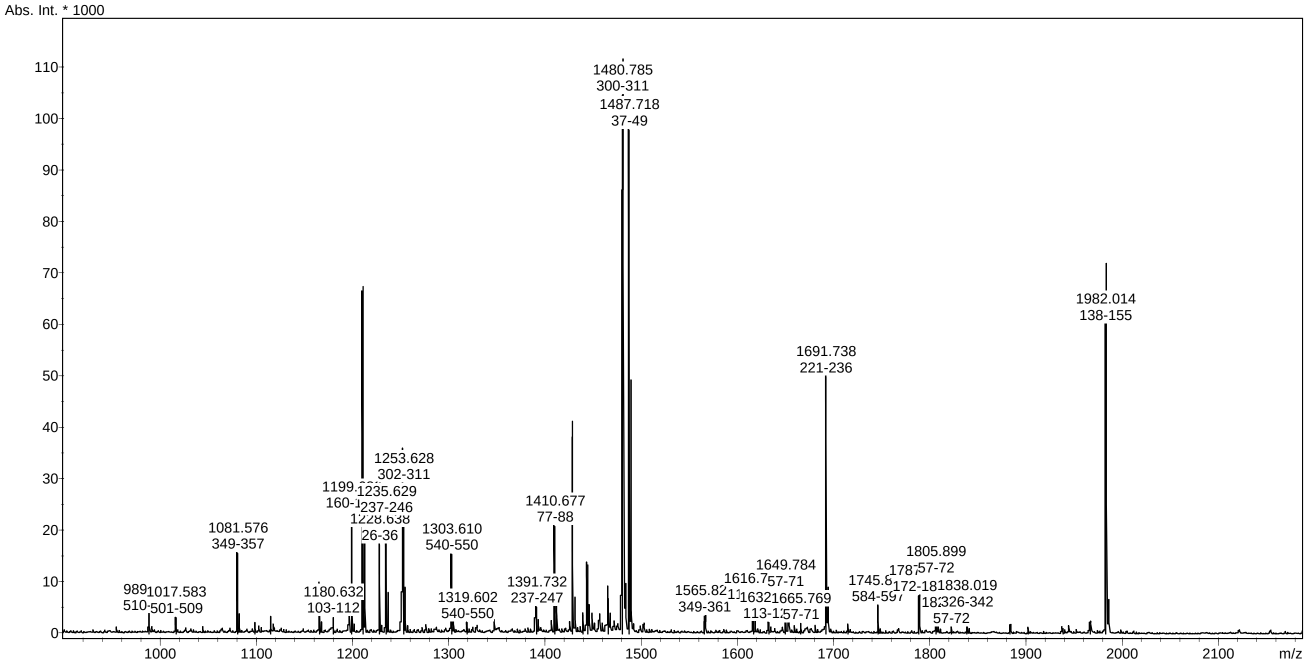

A representative MALDI-ToF PMF spectrum of spot 115

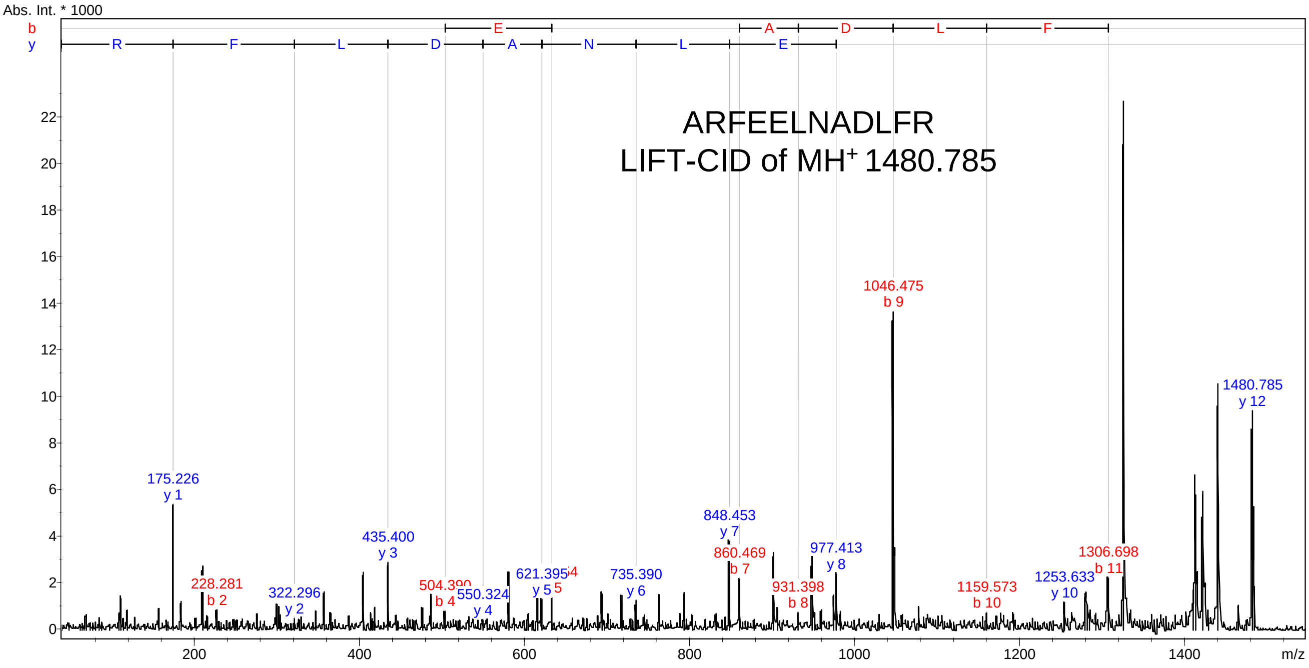

MSMS sequence analysis from the fragmentation of a precursor ion m/z 1480.785 by MALDI-ToF/ToF mass spectrometer

SPOT 116

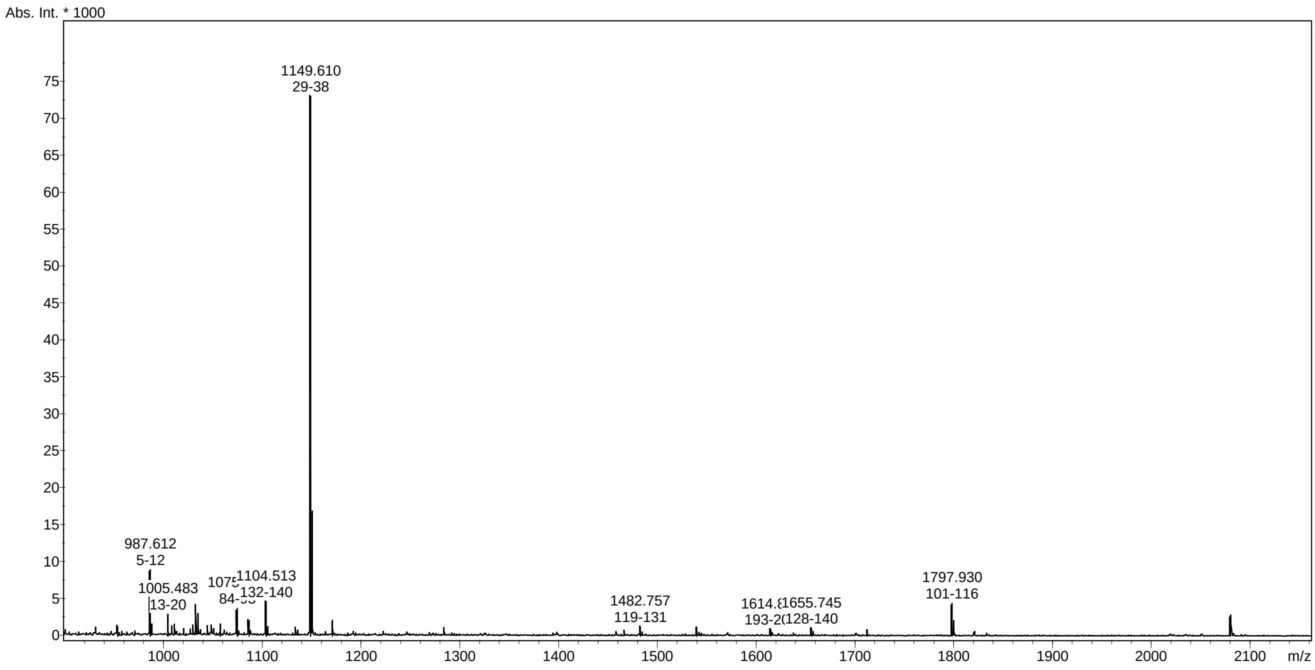

A representative MALDI-ToF PMF spectrum of spot 116

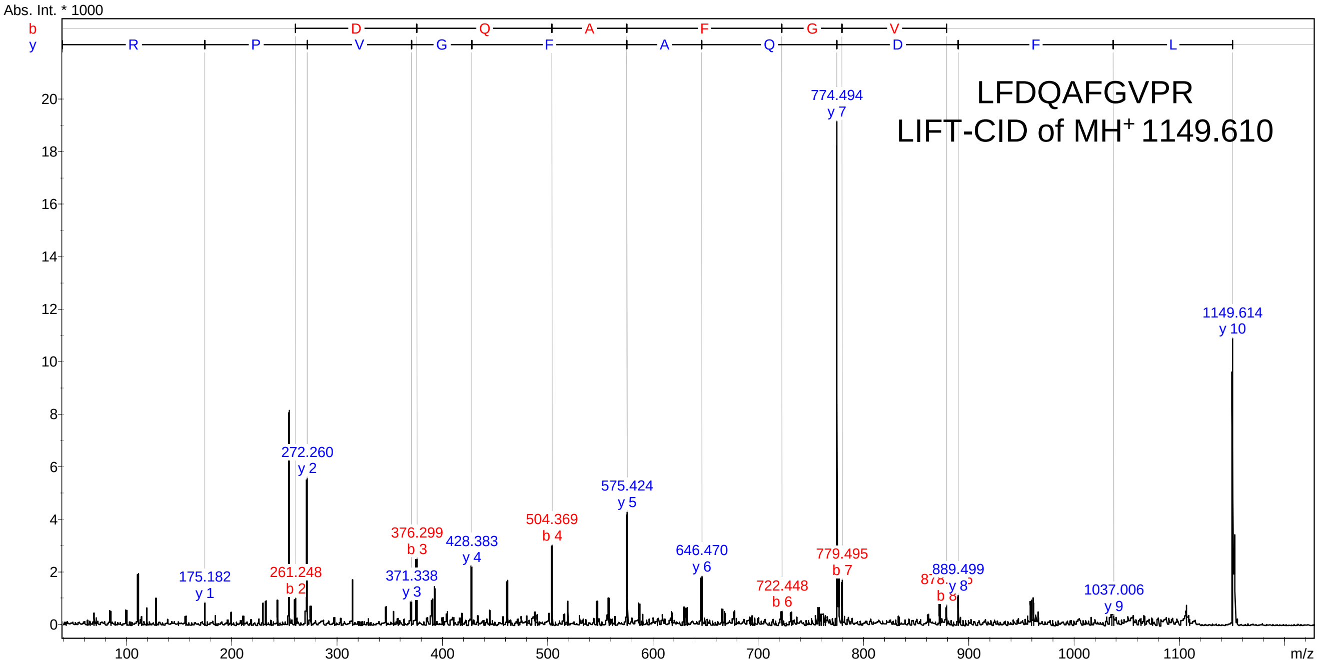

MSMS sequence analysis from the fragmentation of a precursor ion m/z 1149.610 by MALDI-ToF/ToF mass spectrometer

SPOT 117

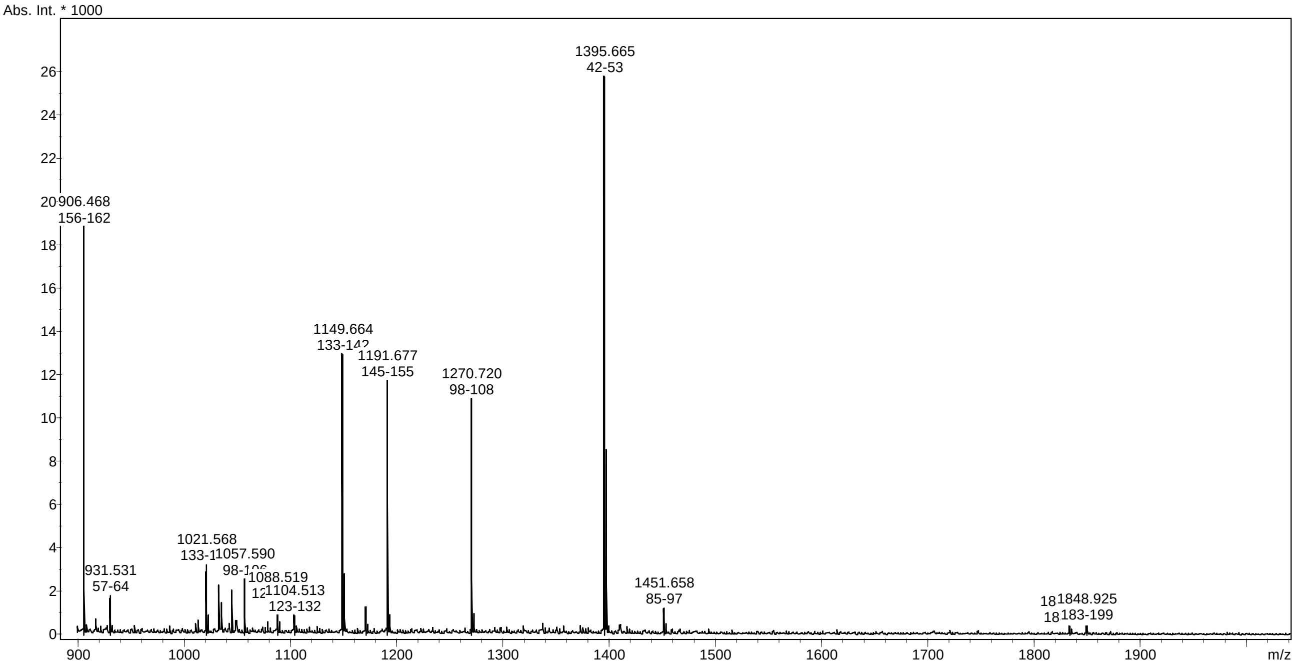

A representative MALDI-ToF PMF spectrum of spot 117

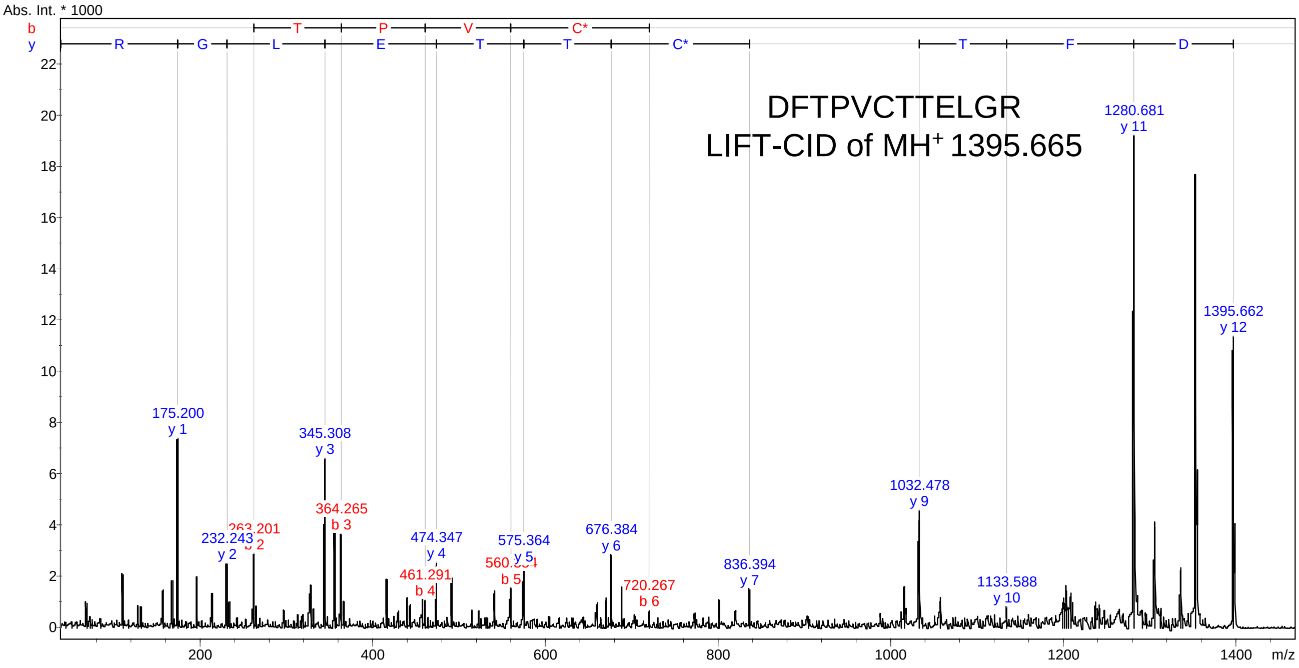

MSMS sequence analysis from the fragmentation of a precursor ion m/z 1130.606 by MALDI-ToF/ToF mass spectrometer
